# Combined Genome Scans for Body Stature in 6,602 European Twins: Evidence for Common Caucasian Loci

**DOI:** 10.1371/journal.pgen.0030097

**Published:** 2007-06-08

**Authors:** Markus Perola, Sampo Sammalisto, Tero Hiekkalinna, Nick G Martin, Peter M Visscher, Grant W Montgomery, Beben Benyamin, Jennifer R Harris, Dorret Boomsma, Gonneke Willemsen, Jouke-Jan Hottenga, Kaare Christensen, Kirsten Ohm Kyvik, Thorkild I. A Sørensen, Nancy L Pedersen, Patrik K. E Magnusson, Tim D Spector, Elisabeth Widen, Karri Silventoinen, Jaakko Kaprio, Aarno Palotie, Leena Peltonen, GenomEUtwin Project

**Affiliations:** 1 Department of Molecular Medicine, National Public Health Institute, Helsinki, Finland; 2 Faculty of Medicine, Department of Medical Genetics, University of Helsinki, Helsinki, Finland; 3 Queensland Institute of Medical Research, Brisbane, Australia; 4 Norwegian Institute of Public Health, Oslo, Norway; 5 Free University, Amsterdam, The Netherlands; 6 Department of Epidemiology, Institute of Public Health, University of Southern Denmark, Odense, Denmark; 7 The Institute of Preventive Medicine, Copenhagen, Denmark; 8 Karolinska Institutet, Stockholm, Sweden; 9 Kings College London, London, United Kingdom; 10 Finnish Genome Center, University of Helsinki, Helsinki, Finland; 11 Faculty of Medicine, Department of Public Health, University of Helsinki, Helsinki, Finland; 12 Department of Mental Health and Alcohol Research, National Public Health Institute, Helsinki, Finland; 13 The Broad Institute, Massachusetts Institute of Technology, Boston, Massachusetts, United States of America; University of Michigan, United States of America

## Abstract

Twin cohorts provide a unique advantage for investigations of the role of genetics and environment in the etiology of variation in common complex traits by reducing the variance due to environment, age, and cohort differences. The GenomEUtwin (http://www.genomeutwin.org) consortium consists of eight twin cohorts (Australian, Danish, Dutch, Finnish, Italian, Norwegian, Swedish, and United Kingdom) with the total resource of hundreds of thousands of twin pairs. We performed quantitative trait locus (QTL) analysis of one of the most heritable human complex traits, adult stature (body height) using genome-wide scans performed for 3,817 families (8,450 individuals) derived from twin cohorts from Australia, Denmark, Finland, Netherlands, Sweden, and United Kingdom with an approximate ten-centimorgan microsatellite marker map. The marker maps for different studies differed and they were combined and related to the sequence positions using software developed by us, which is publicly available (https://apps.bioinfo.helsinki.fi/software/cartographer.aspx). Variance component linkage analysis was performed with age, sex, and country of origin as covariates. The covariate adjusted heritability was 81% for stature in the pooled dataset. We found evidence for a major QTL for human stature on 8q21.3 (multipoint logarithm of the odds 3.28), and suggestive evidence for loci on Chromosomes X, 7, and 20. Some evidence of sex heterogeneity was found, however, no obvious female-specific QTLs emerged. Several cohorts contributed to the identified loci, suggesting an evolutionarily old genetic variant having effects on stature in European-based populations. To facilitate the genetic studies of stature we have also set up a website that lists all stature genome scans published and their most significant loci (http://www.genomeutwin.org/stature_gene_map.htm).

## Author Summary

Twin cohorts provide a unique advantage for research of the role of genetics and environment behind common complex traits by reducing the variance due to environment, age, and cohort differences. The GenomEUtwin consortium consists of eight twin cohorts with the total resource of hundreds of thousands of twin pairs (http://www.genomeutwin.org). We performed quantitative family-based genetic linkage analysis for one of the most heritable human complex traits, adult stature (body height), using genome-wide scans derived from twin cohorts from Australia, Denmark, Finland, Netherlands, Sweden, and United Kingdom. Age, sex, and country were adjusted for in the data analyses. Human stature was found to be very heritable across all the cohorts and in the combined dataset. We found evidence for a shared genetic locus accounting for human stature on Chromosome 8, and suggestive evidence for loci on Chromosomes X, 7, and 20. Since twins from several countries contributed to the identified loci, an evolutionarily old genetic variant must influence stature in European-based populations. To facilitate the research in the field we have also set up a website that lists all stature genome scans published and their most significant loci (http://www.genomeutwin.org/stature_gene_map.htm).

## Introduction

Human adult stature (body height) has been the target of numerous genetic quantitative trait linkage studies in the past few years [1–17]. Despite high heritability estimates for all populations, based on either twin comparison [18–21] or on actual genetic resemblance in siblings [22], the results have been disappointing and inconsistent, with reports of quantitative trait loci (QTLs) scattered across the genome and rarely replicated. Only loci on Chromosomes 3, 5, 6, and 7 have been implicated in more than one study [14]. It seems that the multifactorial and oligo- or polygenic nature of the trait renders identification of the genes involved quite a formidable task. It is likely that special strategies are needed to tackle the identification of the loci and genes underlying human height.

One obvious strategic decision in addressing a quantitative trait regulated by multiple QTLs, each potentially with a minor effect, is to maximize the sample size. Although this could introduce multiple challenges, including an increase in genetic and phenotypic heterogeneity, robust QTL analysis of large cohorts of families is the natural first choice. Based on a meta-analysis of linkage studies, the only factors independently associated with successful locus identification are an increase in the number of individuals studied and a study sample drawn from one ethnic group [23]. Our approach is based on the idea that in addition to maximizing the sample size, since environmental factors are likely to play a significant role in human stature, it would be desirable to minimize environmental noise by sampling relatives of similar age and shared environment, preferably dizygotic twins.

We performed QTL analysis of stature using data from genome-wide scans performed for 3,817 families (8,450 individuals, 3,301 twin pairs) collected within GenomEUtwin from Caucasian populations in six different countries: Australia, Denmark, Finland, Netherlands, Sweden, and United Kingdom. This study sample extracts its information from twin pairs sharing early environment throughout the critical period of human growth and although we are pooling sibpairs from various populations, they are all of European origin.

## Results

There were a total of 1,945 female–female dizygotic (DZ) twin pairs and 661 male–male DZ twin pairs and 695 opposite-sex twin pairs in the pooled material. Table 1 describes the statistical properties of height by cohort and for the pooled sample. Additional family members were recruited through the twin registries, as can be seen from the family sizes (Table 1). Altogether, there were 8,450 individuals with pheno- and genotype data, including the non-twin members of the families, from Australia (*n* = 2,609), Denmark (*n* = 628), Finland (*n* = 851), Netherlands (*n* = 1,086), Sweden (*n* = 1,064), and United Kingdom (*n* = 2,212). The United Kingdom, Danish, and Swedish cohorts consisted of only DZ twin pairs with no additional family members. The covariate-adjusted heritability of adult height was 82% in the pooled sample, being 93% for the males and 98% for the females when analyzed separately, while it was considerable lower, 80%, for the opposite-sex pairs. The average map density was <10 cM.

**Table 1:**
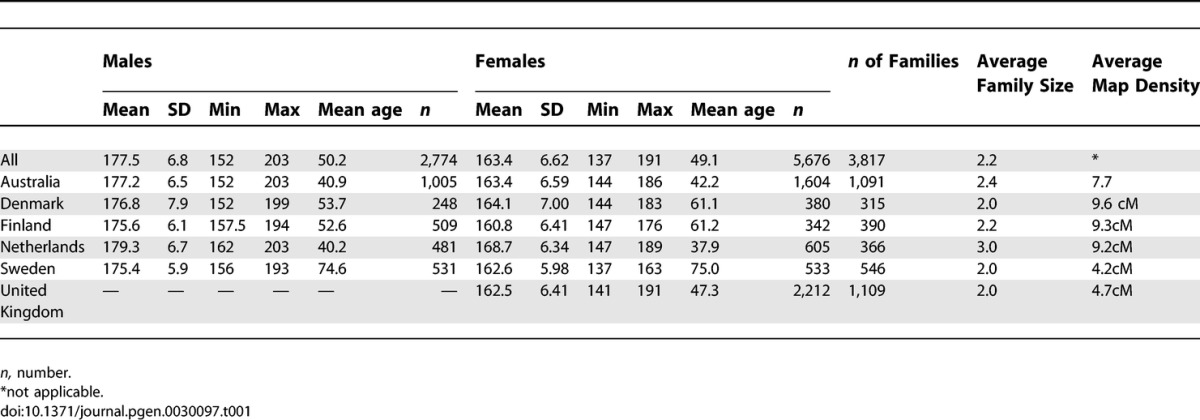
Descriptive Statistics of Stature by Sex and Country in the Study Populations

The overview of the primary results from the pooled genome-wide linkage scan for stature can be seen in Figure 1, while Figure 2 shows the result when analyses were restricted to DZ twin pairs only (excluding additional family members phenotypes from the analyses). Table 2 shows the identified loci with a logarithm of the odds (LOD) score > 2 in the total cohort, and in sex/twin specific analyses. Actual numeric LOD scores and marker location multipoint LOD scores can be found in Table S1. Because of several rounds of testing (the pooled dataset, male–male and female–female pairs and seven subcohorts), we discuss here only the LOD scores >2 or the ones supported by several cohorts and/or previous findings. To limit multiple testing, we refrained from analyzing the subcohorts in a sex-specific manner. The LOD scores are presented unadjusted for the analyses on these six subcohorts (total cohort, DZ twins, and the sex-specific analyses).

**Figure 1:**
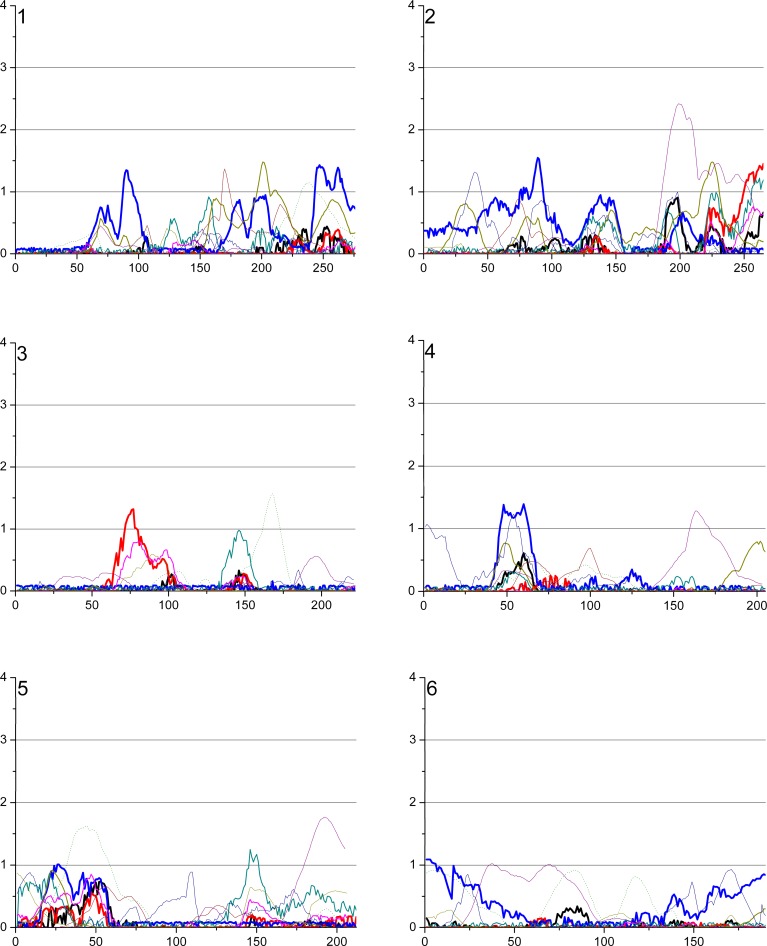
Multipoint LOD Score Curves for Different Study Subcohorts

**Figure 1:**
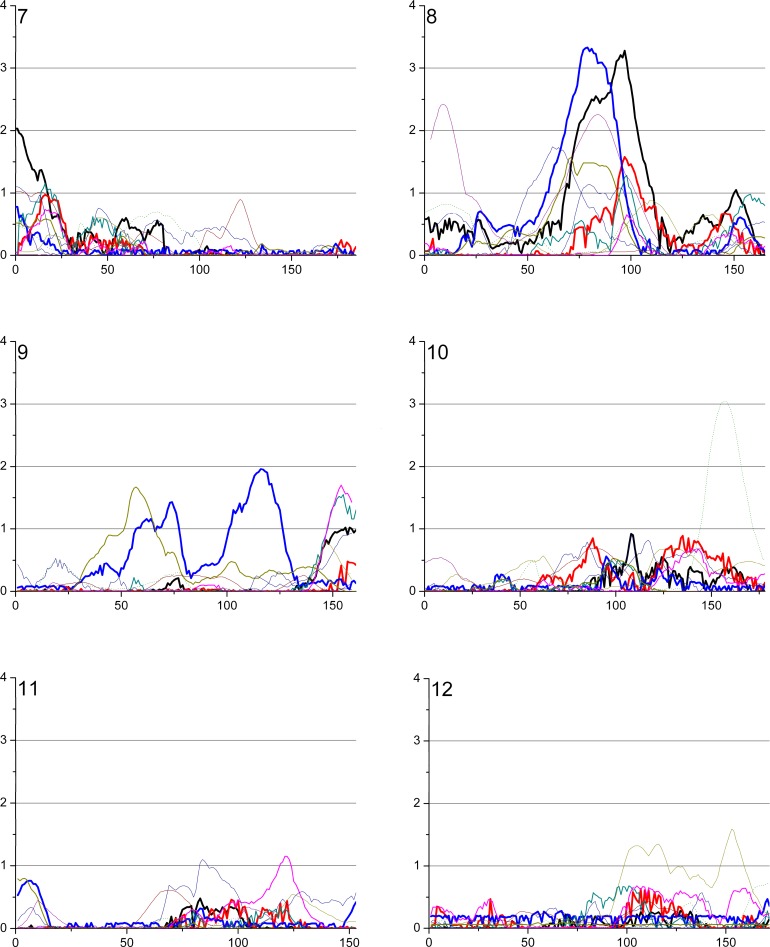
Continued.

**Figure 1:**
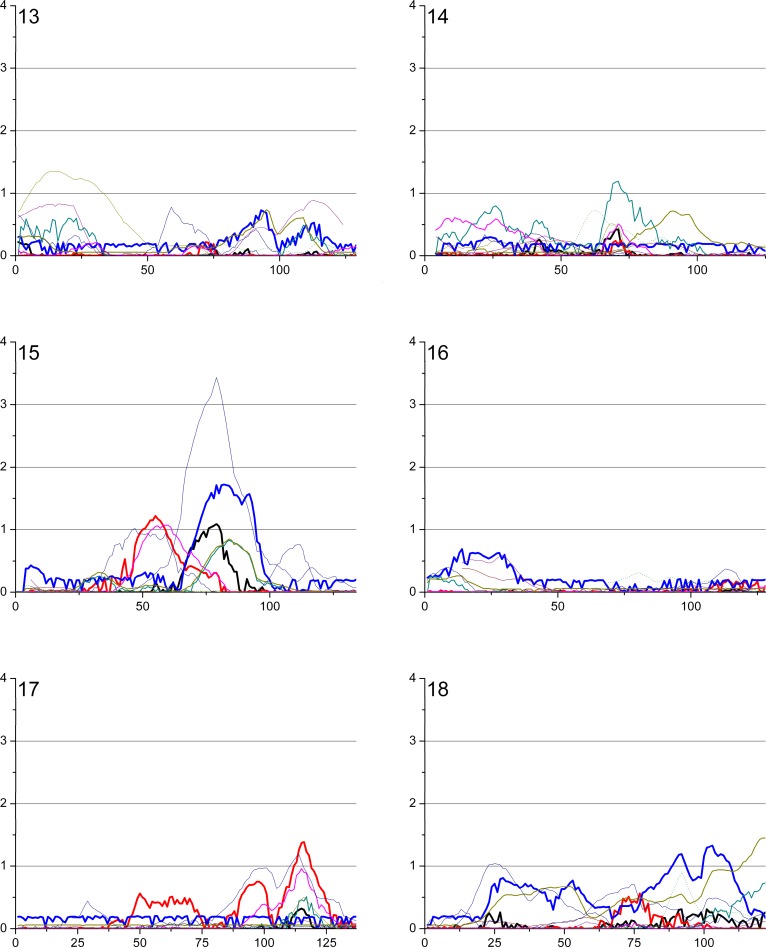
Continued.

**Figure 1:**
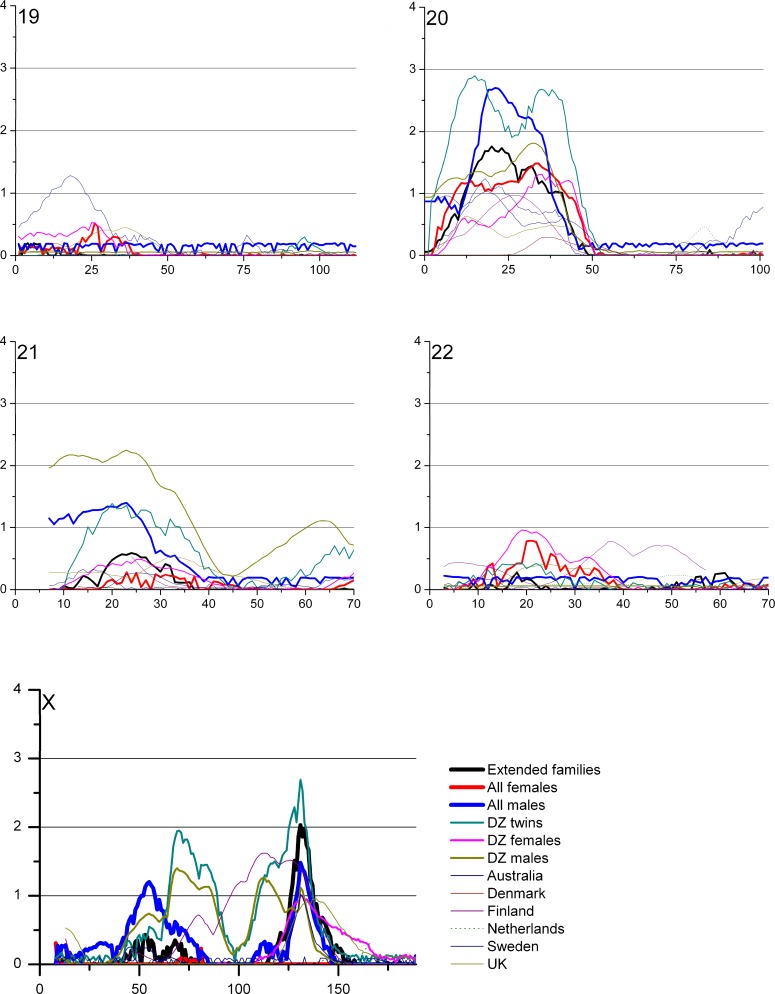
Continued.

**Figure 2:**
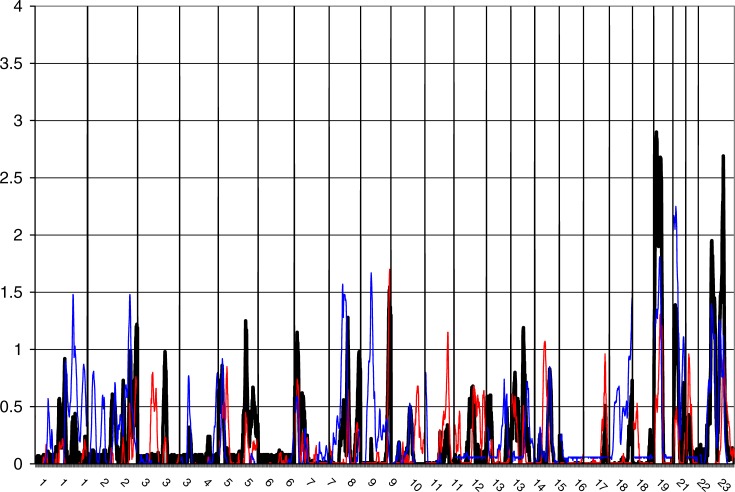
Multipoint LOD Score Curves for the Sample Restricted to DZ Twins Only

**Table 2:**
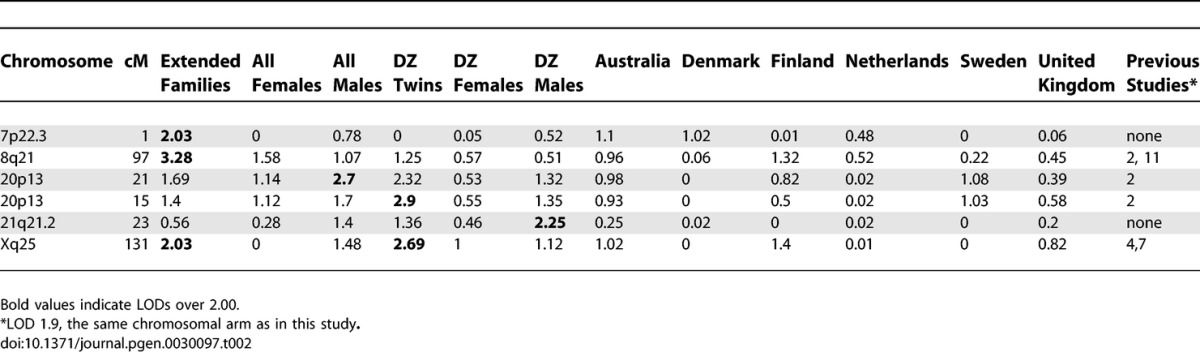
Loci with LOD Scores >2 in the Current Study in the Total Cohort, Sex- and DZ Twin-specific Analyses, by Comparison with Previous Studies

The best evidence for a locus contributing to body stature was achieved by analyzing the complete study sample including cohorts from all six countries. A multipoint LOD score of 3.28 (empirical *p*-value from 100 replications = 0.08, Wilson's 95% confidence interval 0.04–0.15) was observed at 97 cM on 8q21.3. This result was mainly contributed by three cohorts, Australia, Finland, and Sweden (Figure 1). In the sex-specific analysis (see below) the males seemed to contribute most of the signal in this region, albeit the peak for the males only was in a position some 10cM centromeric from the peak obtained for the complete study sample. Interestingly, this locus overlaps with a previously reported locus on 8q in a family sample from Finland [19]. No other loci with LOD score >3 were seen in the pooled study sample. The second best signal of (LOD 2.03) was observed on the telomere region of 7p; this particular area has not previously been reported to be linked to adult stature.

To test if sex-specific loci could be identified in the complete study sample the linkage analysis was performed separately in female–female and male–male pairs. Interestingly, although two-thirds of the total sample (67%) consisted of females, the females-only analyses revealed no LOD scores >2 in the pooled sample. The highest female-only signal was observed on the 8q21.3 region at 98 cM, with an MLOD (multipoint logarithm of the odds) value of 1.50. The highest male-specific signal was also observed at 8q21 with an MLOD 3.33 at 79 cM; these two sex-specific peaks on Chromosome 8 nicely overlapped each other, suggesting that this locus is not sex-specific. The second-highest male signal was observed at 20q21.2 at 21 cM with a suggestive MLOD of 2.70. The same region was previously linked with stature in a study sample of both Finnish and Australian families [15,19].

To minimize environmental heterogeneity, we stratified the sample by excluding all other family members except twins from the variance components analyses; however, all available genotypes were retained for phase information. This stratified sample consisted of 3,301 pairs with pheno- and genotype data. No genome region provided a signal of genome-wide significance in the DZ twin-only sample, probably because of decreased power due to smaller sample of individuals with phenotypes, in statistical analyses. However, in the DZ twins–only sample, a LOD score of 2.54 was identified on Xq25 at 127 cM, slightly distal to a previously linked region in a United States sample [19]. Both the data from male–male and female–female DZ pairs seemed to contribute to the linkage signal for this locus (Figure 1). Suggestive linkage was also observed on 20q (MLOD 2.47), the same region implicated in the males-only analysis of the pooled sample. Lastly, the male DZ twins showed some evidence for linkage (LOD 1.40) in exactly the same location as that seen in a previous Finnish study on 1p21 [14]. In twins-only analysis the locus on 8q21 produced the LOD score of 1.28.

A proper cohort-specific analysis is hampered by the different sample sizes and was thus not performed. However, two relatively significant linkage signals were observed that were mostly contributed by a single cohort; one with the Dutch sample of 366 families (representing 13% of the study individuals) on 10q (MLOD 3.04) and one on 15q24.1 with the Australian cohort of 1,091 families (representing 31 % of the study individuals, MLOD 3.43). Interestingly, these loci have not previously been reported to be linked to adult stature and did not provide significant evidence in the pooled sample.

### Stature Loci and Genes (http://www.genomeutwin.org/stature_gene_map.htm)

**Figure 3:**
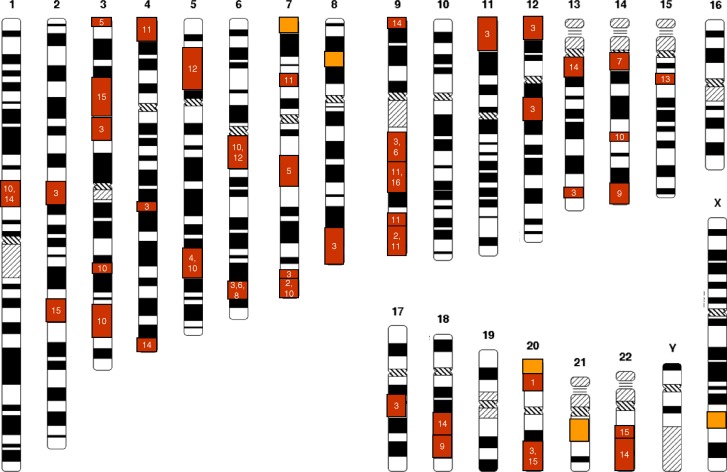
Overview of Loci from Genome-Wide QTL Screens for Stature Where Multipoint LOD Scores over 2.0 Have Been Reported The red boxes represent previous studies and the numbers in them refer to the original publications, while the orange boxes represent loci identified in this study.

To date, about 20 genome scans have been published that investigate human adult body height [1–19]. Since height and various phenotypic characters are recorded in most studies, it is expected that this field will expand even more in the future. To make access more tractable for researchers in the field, we have set up a Web site that lists all genome scans published and their most significant loci. We searched the MEDLINE and PubMed databases (http://www.ncbi.nih.gov) and bibliography reviews (as of December 2006) to identify all eligible studies and have provided the essential data for them as well as the references on the website. The criteria for the eligibility for inclusion on this web site are: (1) the study is a family-based study utilizing genome-wide markers and quantitative linkage analysis on the adult human height and (2) the families are unselected for adult height. Figure 3 illustrates all loci from the studies fulfilling the above criteria with LOD score ≥ 2.

## Discussion

To our knowledge, this is by far the largest genetic linkage study performed regarding human height and on any trait in DZ twins. The nuclear families originated from six different countries of European origin. The method of data pooling used here includes the assumption of some locus homogeneity across the study samples. Silventoinen et al. [19] have shown that there are only minor differences in the genetic architecture of height between these twin cohorts, especially among men. We can see this from our data as well; we obtained evidence for linkage on Chromosomes 8, 20, and X, contributed by both sexes and from several cohorts. Since three identified loci, on 8q, 20p, and Xq25, represent the genome segments earlier identified in family-based studies for stature [19], they probably are of significance for stature generally. That said, the 7q arm that has been linked to stature in at least three different studies [19] did not show any evidence for linkage on the q arm but rather to 7p, which could of course reflect the same signal, but due to long genetic distance between these loci (>100cM) we consider this unlikely.

Growth in twins differs to some degree from that in singletons, especially during fetal life. Dizygotic twins share the same intrauterine conditions, albeit with separate placenta and fetal membranes. Twins grow at the same rate as singletons during the first half of pregnancy, but exhibit slowing growth rates in the third trimester of pregnancy, primarily due to space restriction in utero. This results in lower birth weights than in singletons on average, mostly due to shorter gestation time [24]. Twins show a rapid catch-up growth in infancy, exhibit the same general pattern of growth and onset of puberty as singletons, and do not differ from general population in attained height [25]. However, the initially larger twin (both monozygotic and DZ) at birth tends to remain larger even at adulthood [26]. This shows that the influence of intrauterine conditions for the adult height is of importance and may introduce some “environmental noise” even into this unique human study sample, which is, to a large extent, harmonized for major environmental effects between sibs. Moreover, DZ twins share not only the pre- but also post-birth environment (in most cases). Here, we also formed a hypothesis that by restricting our analyses to DZ twins only, who share all the early life events, the potential environmental (nongenetic) noise affecting growth would be reduced. This would then allow genetic loci components influencing adult stature to be more easily detected, such as the peak (DZ males, LOD 1.47) on a previously linked locus to male stature on 1q21 [19]. It is of interest that the previous study by Sammalisto et al. [19] was performed on a genetically and culturally homogenous population of Finns, and that the linked locus was seen in males only, thus representing some analogy to the subcohort showing some evidence for linkage here. The loci on Chromosomes 20 and 21 also show some evidence for linkage seen only in the cohort restricted to DZ twins; however, the results are only suggestive.

Although the most evident height differences among humans appear between the sexes, and nutrition and infections are critical factors for final adult height across the global populations [27], there is ample evidence that autosomal loci also contribute to human growth and adult height. For example, in Turner's syndrome patients, who possess an abnormal number of X chromosomes, correlations for stature are similar to those for non-Turner mothers and daughters [28]. Also, most of the previously suggested QTLs for stature are located on autosomes [14]; however, the main reason for this is that most of the previous studies report only autosomal data. Our most significant finding on Chromosome 8 is not surprising, since suggestive linkage for stature to 8q24 was previously reported by Hirschorn et al. [3]. Interestingly, the 8q21 area contains the gene for Nijmegen breakage syndrome, which is characterized by growth retardation. After birth the growth rate of these children is appropriate, but individuals remain small for their age.

However, in light of the large difference in stature between males and females, it would be surprising to find no evidence at all for QTL on a sex chromosome in such a large dataset. The potential role of the X-chromosomal locus was identified through analyses based on data from several cohorts, and this locus would not have been apparent in any stand-alone cohort analysis. This lends support to the pooling strategy used here to identify such minor-to-moderate QTLs. This X-chromosomal locus, Xq25, has previously shown suggestive linkage to stature in pedigrees of European origin [29] and it harbors several candidate genes for growth. Telomerically from the peak marker, DXS1047, lies the gene associated with Borjeson-Forssman-Lehmann syndrome, X-linked mental retardation of which one characteristic is moderately short stature [30,31]. Two genes associated with the syndrome, the widely expressed plant homeodomain–like finger gene, *PHF6,* and fibroblast growth factor 13, *FGF13,* [29] lie within 9 Mb of DXS1047. Other potential candidate genes on the area are the skeletal muscle LIM protein 1, *SLIM 1,* which shows elevated expression levels in skeletal muscle during postnatal growth, and glypican 3, *GPC3.* Variants in *GPC3* cause Simpson-Golabi-Behmel syndrome, a condition characterized by pre- and postnatal overgrowth (gigantism) with visceral and skeletal anomalies [32]. *GPC3* seems to be involved in the suppression/modulation of growth in the mainly mesodermal tissues and organs and also may interact with the insulin-like growth factor II *(IGF2),* thus regulating growth [33].

There are no previous QTLs linked to stature on Chromosome 15 [14]. Here, the only subcohort linked to that locus was the largest, the Australian cohort. This cohort constitutes almost one third of the total sample (30.8%). The linked families were not characteristically different from others in the Australian cohort (unpublished data); there were no outliers for stature values and rigorous quality control of genotypes did not expose obvious errors in data produced. The Australian cohort is mostly European based, as are the other cohorts, and was earlier found to be comparable to a European population in regard to microsatellite allele [34]. Thus, although other cohorts and the pooled analyses did not lend significant support for linkage to this region, we find it potentially interesting, while being aware that replication is needed to confirm the potential QTL for stature on 15q. The same applies to the locus on 10q for the Dutch sample—this locus has not previously been reported to be linked to stature in other populations, nor was there any evidence for linkage in other subgroups in this study. Part of the Dutch cohort was included in an earlier study on stature, in which the Chromosome 10 peak was also seen [14]. Here, however, the Dutch cohort includes more people and not all the loci in the earlier study [14] are as evident as the one on Chromosome 10. Also, the Finnish cohort showed some evidence of linkage on previously linked loci for stature in Finnish studies, such as 2q, 4q [14], and 9q [14]. Obviously, differing information content of the genotyped cohorts could potentially explain these population-specific peaks; however, this is not evident from our data. The genotyping success rates and map densities did not show variations on the above-mentioned population-specific loci (unpublished data).

We identified several other potential QTLs, which were not statistically significant, but nevertheless triggered some interest. For example, the loci for which data from many cohorts seem to contribute to and increase the overall LOD score (20p and Xq) might mirror minor loci either needing larger sampling or some kind of dissection of the sample to produce a more homogeneous sample in relation to the locus in question. Loci that have previously been reported to be linked to stature, such as on Chromosomes 8q, 20q, 21p, and Xq25, might well present a real QTL, but due to multiple testing of genome-wide scans here we cannot apply the more relaxed linkage thresholds that are often used in replication studies, and, thus, they remain suggestive. Several previous loci were not replicated in this study, maybe because stature is a very heterogeneous phenotype, or possibly due to type I or II errors in this or earlier studies. We think that our findings support the essential argument of quantitative genetics, that of the infinitesimal model. From the results we can deduce that, most probably for stature, the ultimate causation of variance components is segregating alleles at many underlying loci, each of which has a very small individual impact on the character in question. One of Fisher's several important contributions to evolutionary theory was to provide a statistical outline for such a complex inheritance model in a Mendelian framework that could account for continuous variation [35], giving rise to the field of quantitative genetics [36,37]. Here, we have combined genome scans using multiallelic markers from a total of 8,450 individuals, including 3,301 DZ twin pairs, the largest published twin QTL genome scan, with microsatellite genome-wide scan data, and produced evidence for several potential stature QTLs in this Caucasian study sample in a manner confirmed to minimize the nongenetic noise of early environment. However, given the relatively low LOD scores observed in even this sizable study sample, it is quite evident that, although highly heritable, human stature is quite polygenic, probably determined by several minor QTLs, interacting with environment and sex differences and adding up to the phenomenon we know as variation in stature.

## Materials and Methods

GenomEUtwin is a research consortium of 12 partners, including eight twin cohorts from Europe and Australia (http://www.genomeutwin.org), formed to explore genetic influences on common traits using large population and twin cohorts. For these analyses, genome-wide microsatellite scan data were available from six twin cohorts: The Australian Twin Registry [38], The Danish Twin Registry [39], The Finnish Twin Cohort [40], The Netherlands Twin Register [41], The Swedish Twin Registry [42], and United Kingdom St. Thomas' UK Adult Twin Registry [43]. While the Australian twin cohort is geographically quite distinct from the others, it does have a mostly European-based population background. The Australian twin registry has self-reported ancestry information on a random subset that is representative of the entire sample. From this subset (897 families), 99.7% are Caucasian. From that, 92.5% are from the United Kingdom. The rest are from Europe and the United States.

While the twin studies served as the recruitment basis for the participants, additional siblings and/or parents/descendants of the twins were also recruited in some cohorts and included in the analysis set here where available. The total number of families included in the analysis was 3,817, and the number of full DZ sib pairs with genome-wide marker and phenotype data was 3,301. A federated database with open-source code has been created to share the data across the study partners [44,45]. Genotypes for the Australian, Dutch, and United Kingdom cohorts, as well as 102 Swedish twins, were produced as described earlier [12,46,47]. Dutch samples were genotyped by the Mammalian Genotyping Service in Marshfield, and the Molecular Epidemiology Section in Leiden University Medical Centre (The Netherlands). The Finnish subjects, as well as the Danes, were genotyped at the Finnish Genome Center (using 96 capillary Megabase 1000 sequencers [Amersham Biosciences, http://www1.gelifesciences.com]) and partly at Uppsala Rudbeck laboratory (using Applied Biosystems automated DNA sequencing system, http://www.appliedbiosystems.com). A total of 487 Swedish twin pairs were genotyped by deCODE Genetics (http://www.decode.com), using the Applied Biosystems automated DNA sequencing system (1000 marker, 4-cM map).

The program GRR, (Graphical Representation of Relationships, http://www.sph.umich.edu/csg/abecasis/GRR/index.html) [48] was used to screen for inconsistencies in familial relationships as well as excluding potential monozygotic twin pairs. Between-group phenotypic comparisons were conducted using the program SAS 8.2 (SAS Institute, http://www.sas.com). Measured height was available from the Dutch and United Kingdom cohorts as well as from large parts of the Australian and Danish cohort, while the Swedish and Finnish height data was acquired from questionnaires. Genotypes were checked for Mendelian inconsistencies using the PedCheck program [49] and the program Merlin's [50] genotyping-error option was used for identifying problematic-yet-Mendelian genotypes, which were then excluded from the analyses using Merlin's Pedwipe-program.

Having access to raw data of all the genome-wide scans performed, we decided to pool all raw data (genotypes and phenotypes) instead of applying meta-analytic strategy. Since the original genome scans differed in their selection of genetic markers, we first harmonized the genetic marker maps using the in-house-developed Cartographer program [14]. Cartographer retrieves the physical location of the markers from the University of California Santa Cruz (UCSC) database and orders the markers based on the sequence information. The genetic location of each marker is defined using the published deCODE genetic map [51], which are also stored in the UCSC database. For markers that were not included in the deCODE genetic map, we used linear interpolation for obtaining estimates of the genetic locations of these markers by using the physical and the genetic locations of the immediately flanking deCODE markers. Also, if the physical location from the UCSC database and the genetic location from the deCODE genetic map for a given marker were in disagreement, we obtained an estimate of the genetic location via interpolation using the nearest flanking deCODE markers that are in agreement with the sequence information. For those markers that were not found in the UCSC database, we retrieved PCR primers from UniSTS and used these to map the marker using UCSC in-silico PCR, and performed the linear interpolation to obtain an estimate of the genetic location as described previously [14].

After harmonization of the maps across the cohorts studied, the cohorts were pooled together such that all identical markers that were genotyped in several populations were renamed for all of them and these renamed markers were located at 0.001 recombination fractions from each other. A total of 253 markers were genotyped in all six populations (90 in five populations, 50 in four, 142 in three and 238 in two, while 2,939 markers were genotyped in only one population). To circumvent the problems produced by this partial overlap of markers among the cohorts when interpreting two-point LOD scores, only multipoint linkage results are shown here. All data were then analyzed together using the variance component method in the program Merlin [50]. Genome-wide scan was automized by using the program AUTOGSCAN [52]. People differing in height by more than four standard deviations from the population-and sex-specific mean were excluded from the phenotypic analyses, but their genotypes were included for phase-determination purposes.

Age, sex, and country of origin were used as covariates in the variance component analysis. The distribution of height was not notably skewed or kurtotic, so no transformations of any of the variables were necessary. Sexes were also analyzed separately with age and country of origin as covariates. The LOD scores are presented unadjusted for the analyses on these six subcohorts (total cohort, DZ twins, and the sex-specific analyses). No monozygotic twin pairs were identified in the analyses.

To estimate empirical *p*-values for the obtained results, a total of 100 replicates was created and analyzed using Merlin's simulate option. The same analyses that were conducted with the actual data were repeated for each of the simulated genomes. Since any evidence of linkage found in the simulated genomes is due to chance, these simulations allowed us to evaluate the false-positive rate. We determined the empirical genome-wide significance of a given LOD score as the fraction of simulated genome scans in which this LOD score was reached or exceeded. The Wilson confidence intervals were estimated as described in [53].

## Supporting Information

Table S1 Click here for additional data file. **Marker Location of Multipoint LOD Scores**
(2.4 MB PDF)

### Accession Numbers

The Online Mendelian Inheritance in Man (OMIM) database accession numbers for the syndromes discussed in this paper are Borjeson-Forssman-Lehmann syndrome, 301900; Nijmegen breakage syndrome, 251260; and Simpson-Golabi-Behmel syndrome, 312870.

## Abbreviations

DZ: dizygotic
LOD: logarithm of the odds
MLOD: multipoint logarithm of the odds
QTL: quantitative trait locus
UCSC: University of California Santa Cruz

## References

[pgen-0030097-b001] Thompson DB, Ossowski V, Janssen RC, Knowler WC, Bogardus C (1995). Linkage between stature and a region on Chromosome 20 and analysis of a candidate gene, bone morphogenetic protein 2. Am J Med Genet.

[pgen-0030097-b002] Perola M, Ohman M, Hiekkalinna T, Leppavuori J, Pajukanta P (2001). Quantitative-trait-locus analysis of body-mass index and of stature, by combined analysis of genome scans of five Finnish study groups. Am J Hum Genet.

[pgen-0030097-b003] Hirschhorn JN, Lindgren CM, Daly MJ, Kirby A, Schaffner SF (2001). Genomewide linkage analysis of stature in multiple populations reveals several regions with evidence of linkage to adult height. Am J Hum Genet.

[pgen-0030097-b004] Deng HW, Xu FH, Liu YZ, Shen H, Deng H (2002). A whole-genome linkage scan suggests several genomic regions potentially containing QTLs underlying the variation of stature. Am J Med Genet.

[pgen-0030097-b005] Wiltshire S, Frayling TM, Hattersley AT, Hitman GA, Walker M (2002). Evidence for linkage of stature to Chromosome 3p26 in a large U.K. Family data set ascertained for type 2 diabetes. Am J Hum Genet.

[pgen-0030097-b006] Xu J, Bleecker ER, Jongepier H, Howard TD, Koppelman GH (2002). Major recessive gene(s) with considerable residual polygenic effect regulating adult height: confirmation of genomewide scan results for Chromosomes 6, 9, and 12. Am J Hum Genet.

[pgen-0030097-b007] Beck SR, Brown WM, Williams AH, Pierce J, Rich SS (2003). Age-stratified QTL genome scan analyses for anthropometric measures. BMC Genet.

[pgen-0030097-b008] Geller F, Dempfle A, Gorg T (2003). Genome scan for body mass index and height in the Framingham Heart Study. BMC Genet.

[pgen-0030097-b009] Mukhopadhyay N, Weeks DE (2003). Linkage analysis of adult height with parent-of-origin effects in the Framingham Heart Study. BMC Genet.

[pgen-0030097-b010] Wu X, Cooper RS, Boerwinkle E, Turner ST, Hunt S (2003). Combined analysis of genomewide scans for adult height: results from the NHLBI Family Blood Pressure Program. Eur J Hum Genet.

[pgen-0030097-b011] Liu YZ, Xu FH, Shen H, Liu YJ, Zhao LJ (2004). Genetic dissection of human stature in a large sample of multiplex pedigrees. Ann Hum Genet.

[pgen-0030097-b012] Willemsen G, Boomsma DI, Beem AL, Vink JM, Slagboom PE (2004). QTLs for height: Results of a full genome scan in Dutch sibling pairs. Eur J Hum Genet.

[pgen-0030097-b013] Sale MM, Freedman BI, Hicks PJ, Williams AH, Langefeld CD (2005). Loci contributing to adult height and body mass index in African American families ascertained for type 2 diabetes. Ann Hum Genet.

[pgen-0030097-b014] Sammalisto SA, Hiekkalinna T, Suviolahti E, Sood K, Metzidis A (2005). A male-specific QTL on 1p21 controlling human stature. J Med Genet.

[pgen-0030097-b015] Ellis JA, Scurrah KJ, Duncan AE, Lamantia A, Byrnes GB (2006). Comprehensive multi-stage linkage analyses identify a locus for adult height on Chromosome 3p in a healthy Caucasian population. Hum Genet.

[pgen-0030097-b016] Liu YZ, Xiao P, Guo YF, Xiong DH, Zhao LJ (2006). Genetic linkage of human height is confirmed to 9q22 and Xq24. Hum Genet.

[pgen-0030097-b017] Shmulewitz D, Heath SC, Blundell ML, Han Z, Sharma R (2006). Linkage analysis of quantitative traits for obesity, diabetes, hypertension, and dyslipidemia on the island of Kosrae, Federated States of Micronesia. Proc Natl Acad Sci U S A.

[pgen-0030097-b018] Healey SC, Kirk KM, Hyland VJ, Munns CF, Henders AK (2001). Height discordance in monozygotic females is not attributable to discordant inactivation of X-linked stature determining genes. Twin Res.

[pgen-0030097-b019] Silventoinen K, Sammalisto S, Perola M, Boomsma DI, Cornes BK (2003). Heritability of adult body height: A comparative study of twin cohorts in eight countries. Twin Res.

[pgen-0030097-b020] Macgregor S, Cornes BK, Martin NG, Visscher PM (2006). Bias, precision and heritability of self-reported and clinically measured height in Australian twins. Hum Genet.

[pgen-0030097-b021] Silventoinen K (2003). Determinants of variation in adult body height. J Biosoc Sci.

[pgen-0030097-b022] Visscher PM, Medland SE, Ferreira MA, Morley KI, Zhu G (2006). Assumption-free estimation of heritability from genome-wide identity-by-descent sharing between full siblings. PLoS Genet.

[pgen-0030097-b023] Altmuller J, Palmer LJ, Fischer G, Scherb H, Wjst M (2001). Genomewide scans of complex human diseases: True linkage is hard to find. Am J Hum Genet.

[pgen-0030097-b024] Blickstein I, Blickstein I, Keith LG (2005). Intrauterine growth. Multiple pregnancy: Epidemiology, gestation & perinatal outcome. 2nd edition.

[pgen-0030097-b025] Wilson R, Falkner F, Tanner JM (1986). Growth and development of human twins. Human growth: A comprehensive treatise volume 3: Methodology; ecological, genetic, and nutritional effects on growth.

[pgen-0030097-b026] Pietilainen KH, Kaprio J, Rasanen M, Rissanen A, Rose RJ (2002). Genetic and environmental influences on the tracking of body size from birth to early adulthood. Obes Res.

[pgen-0030097-b027] Eveleth PB TJ (1990). Worldwide variation in human growth.

[pgen-0030097-b028] Brook CG (1974). Proceedings: Growth in children with 45XO Turner's syndrome. Arch Dis Child.

[pgen-0030097-b029] Gecz J, Baker E, Donnelly A, Ming JE, McDonald-McGinn DM (1999). Fibroblast growth factor homologous factor 2 (FHF2): gene structure, expression and mapping to the Borjeson-Forssman-Lehmann syndrome region in Xq26 delineated by a duplication breakpoint in a BFLS-like patient. Hum Genet.

[pgen-0030097-b030] Lower KM, Turner G, Kerr BA, Mathews KD, Shaw MA (2002). Mutations in PHF6 are associated with Borjeson-Forssman-Lehmann syndrome. Nat Genet.

[pgen-0030097-b031] Turner G, Lower KM, White SM, Delatycki M, Lampe AK (2004). The clinical picture of the Borjeson-Forssman-Lehmann syndrome in males and heterozygous females with PHF6 mutations. Clin Genet.

[pgen-0030097-b032] Pilia G, Hughes-Benzie RM, MacKenzie A, Baybayan P, Chen EY (1996). Mutations in GPC3, a glypican gene, cause the Simpson-Golabi-Behmel overgrowth syndrome. Nat Genet.

[pgen-0030097-b033] Weksberg R, Squire JA (1996). Molecular biology of Beckwith-Wiedemann syndrome. Med Pediatr Oncol.

[pgen-0030097-b034] Sullivan PF, Montgomery GW, Hottenga JJ, Wray NR, Boomsma DI, Martin NG (2006). Empirical evaluation of the genetic similarity of samples from twin registries in Australia and the Netherlands using 359 STRP markers. Twin Res Hum Genet.

[pgen-0030097-b035] Fisher R (1918). The correlation between relatives on the supposition of Mendelian inheritance. Trans R Soc Edinb Earth Sci.

[pgen-0030097-b036] Bulmer M (1980). The mathematical theory of quantitative genetics.

[pgen-0030097-b037] Falconer D, Mackay T (1996). Introduction to quantitative genetics.

[pgen-0030097-b038] Hopper JL (2002). The Australian Twin Registry. Twin Res.

[pgen-0030097-b039] Skytthe A, Kyvik K, Holm NV, Vaupel JW, Christensen K (2002). The Danish Twin Registry: 127 birth cohorts of twins. Twin Res.

[pgen-0030097-b040] Kaprio J, Koskenvuo M (2002). Genetic and environmental factors in complex diseases: The older Finnish Twin Cohort. Twin Res.

[pgen-0030097-b041] Boomsma DI, Vink JM, van Beijsterveldt TC, de Geus EJ, Beem AL (2002). Netherlands Twin Register: A focus on longitudinal research. Twin Res.

[pgen-0030097-b042] Pedersen NL, Lichtenstein P, Svedberg P (2002). The Swedish Twin Registry in the third millennium. Twin Res.

[pgen-0030097-b043] Spector TD, MacGregor AJ (2002). The St. Thomas' UK Adult Twin Registry. Twin Res.

[pgen-0030097-b044] Litton JE, Muilu J, Bjorklund A, Leinonen A, Pedersen NL (2003). Data modeling and data communication in GenomEUtwin. Twin Res.

[pgen-0030097-b045] Muilu J, Peltonen L, Litton JE (2007). The federated database - a basis for biobank-based post-genome studies, integrating phenome and genome data from 600 000 twin pairs in Europe. Eur J Hum Genet.

[pgen-0030097-b046] Wilson SG, Reed PW, Bansal A, Chiano M, Lindersson M (2003). Comparison of genome screens for two independent cohorts provides replication of suggestive linkage of bone mineral density to 3p21 and 1p36. Am J Hum Genet.

[pgen-0030097-b047] Cornes BK, Medland SE, Ferreira MA, Morley KI, Duffy DL (2005). Sex-limited genome-wide linkage scan for body mass index in an unselected sample of 933 Australian twin families. Twin Res Hum Genet.

[pgen-0030097-b048] Abecasis GR, Cherny SS, Cookson WO, Cardon LR (2001). GRR: graphical representation of relationship errors. Bioinformatics.

[pgen-0030097-b049] O'Connell JR, Weeks DE (1998). PedCheck: A program for identification of genotype incompatibilities in linkage analysis. Am J Hum Genet.

[pgen-0030097-b050] Abecasis GR, Cherny SS, Cookson WO, Cardon LR (2002). Merlin–rapid analysis of dense genetic maps using sparse gene flow trees. Nat Genet.

[pgen-0030097-b051] Kong A, Gudbjartsson DF, Sainz J, Jonsdottir GM, Gudjonsson SA (2002). A high-resolution recombination map of the human genome. Nat Genet.

[pgen-0030097-b052] Hiekkalinna T, Terwilliger JD, Sammalisto S, Peltonen L, Perola M (2005). AUTOGSCAN: Powerful tools for automated genome-wide linkage and linkage disequilibrium analysis. Twin Res Hum Genet.

[pgen-0030097-b053] Brown LD, Cai TT, DasGupta A (2001). Interval estimation for a binomial proportion. Stat Sci.

