# Supplementary material for: Combined Genome Scans for Body Stature in 6,602 European Twins: Evidence for Common Caucasian Loci
Source: PLoS Genet. 2007 Jun 8;3(6):e97. doi: 10.1371/journal.pgen.0030097 (PMC1892350; doi:10.1371/journal.pgen.0030097)
Supplement: Supplementary file 1 [file pgen.0030097.st001.pdf]

		Extended Families									DZ Twins Only		
Chromosome	cM	Both sexes	Females	Males	Australia*	Denmark*	Finland*	Netherlands*	Sweden*	UK*	Both sexes	Females	Males
1	0	0.00	0.00	0.00	0.00	0.00	0.00	0.00	0.00	0.00	0.00	0.00	0.00
1	1	0.00	0.00	0.07	0.00	0.00	0.00	0.00	0.00	0.00	0.00	0.00	0.00
1	2	0.00	0.00	0.08	0.00	0.00	0.00	0.00	0.00	0.00	0.00	0.00	0.00
1	3	0.02	0.00	0.07	0.00	0.00	0.00	0.00	0.00	0.00	0.00	0.00	0.00
1	4	0.00	0.00	0.08	0.00	0.00	0.00	0.02	0.00	0.01	0.00	0.00	0.00
1	5	0.00	0.00	0.09	0.00	0.00	0.00	0.02	0.00	0.01	0.00	0.00	0.00
1	6	0.00	0.00	0.07	0.00	0.00	0.00	0.02	0.00	0.01	0.04	0.00	0.00
1	7	0.00	0.00	0.09	0.00	0.00	0.00	0.02	0.00	0.01	0.01	0.00	0.00
1	8	0.00	0.00	0.07	0.00	0.00	0.00	0.02	0.00	0.01	0.00	0.00	0.00
1	9	0.01	0.00	0.07	0.00	0.00	0.00	0.02	0.00	0.01	0.06	0.00	0.00
1	10	0.01	0.00	0.06	0.00	0.00	0.00	0.02	0.00	0.01	0.00	0.00	0.00
1	11	0.00	0.00	0.09	0.00	0.00	0.00	0.02	0.00	0.01	0.00	0.00	0.00
1	12	0.00	0.00	0.04	0.00	0.00	0.00	0.02	0.00	0.00	0.00	0.00	0.00
1	13	0.00	0.00	0.10	0.00	0.00	0.00	0.02	0.00	0.01	0.07	0.00	0.00
1	14	0.02	0.00	0.02	0.00	0.00	0.00	0.02	0.03	0.01	0.04	0.00	0.00
1	15	0.00	0.00	0.10	0.00	0.00	0.00	0.02	0.04	0.00	0.00	0.00	0.00
1	16	0.00	0.02	0.06	0.00	0.00	0.00	0.02	0.04	0.00	0.00	0.00	0.00
1	17	0.00	0.01	0.08	0.00	0.00	0.00	0.02	0.03	0.00	0.00	0.00	0.00
1	18	0.00	0.00	0.08	0.00	0.00	0.00	0.02	0.00	0.00	0.01	0.00	0.00
1	19	0.01	0.02	0.07	0.00	0.00	0.00	0.02	0.00	0.01	0.07	0.00	0.00
1	20	0.00	0.03	0.09	0.00	0.00	0.00	0.02	0.00	0.01	0.00	0.00	0.00
1	21	0.00	0.00	0.08	0.00	0.00	0.00	0.02	0.04	0.00	0.07	0.00	0.00
1	22	0.01	0.02	0.00	0.00	0.00	0.00	0.02	0.00	0.00	0.00	0.00	0.00
1	23	0.00	0.00	0.05	0.00	0.00	0.00	0.02	0.04	0.01	0.06	0.00	0.00
1	24	0.00	0.00	0.09	0.00	0.00	0.00	0.03	0.04	0.00	0.00	0.00	0.00
1	25	0.00	0.00	0.03	0.00	0.00	0.00	0.03	0.00	0.01	0.06	0.00	0.00
1	26	0.00	0.00	0.06	0.00	0.00	0.00	0.04	0.00	0.00	0.04	0.00	0.00
1	27	0.00	0.03	0.07	0.00	0.01	0.00	0.05	0.00	0.01	0.06	0.00	0.00
1	28	0.00	0.03	0.00	0.00	0.01	0.00	0.06	0.00	0.01	0.05	0.00	0.00
1	29	0.00	0.00	0.08	0.00	0.02	0.00	0.07	0.00	0.01	0.00	0.00	0.00
1	30	0.00	0.03	0.01	0.00	0.03	0.00	0.09	0.04	0.01	0.00	0.00	0.00
1	31	0.01	0.00	0.05	0.00	0.04	0.00	0.10	0.00	0.01	0.00	0.00	0.00
1	32	0.00	0.00	0.08	0.00	0.04	0.00	0.10	0.00	0.00	0.05	0.00	0.00
1	33	0.00	0.02	0.08	0.00	0.05	0.00	0.12	0.00	0.00	0.06	0.00	0.00

1	34	0.00	0.01	0.03	0.00	0.05	0.00	0.12	0.00	0.01	0.05	0.00	0.00
1	35	0.00	0.00	0.07	0.00	0.05	0.00	0.13	0.00	0.00	0.04	0.00	0.00
1	36	0.00	0.00	0.08	0.00	0.05	0.00	0.13	0.00	0.00	0.04	0.00	0.00
1	37	0.00	0.01	0.00	0.00	0.05	0.00	0.14	0.00	0.00	0.00	0.00	0.00
1	38	0.00	0.00	0.00	0.00	0.05	0.00	0.14	0.00	0.01	0.00	0.00	0.00
1	39	0.00	0.01	0.08	0.00	0.05	0.00	0.14	0.03	0.00	0.06	0.00	0.00
1	40	0.00	0.00	0.08	0.00	0.05	0.00	0.15	0.03	0.01	0.00	0.00	0.00
1	41	0.00	0.00	0.08	0.00	0.06	0.00	0.17	0.04	0.00	0.06	0.01	0.00
1	42	0.00	0.00	0.08	0.00	0.06	0.00	0.17	0.02	0.01	0.07	0.00	0.00
1	43	0.00	0.00	0.08	0.00	0.06	0.00	0.18	0.01	0.00	0.04	0.04	0.00
1	44	0.00	0.00	0.07	0.00	0.05	0.00	0.19	0.04	0.00	0.01	0.05	0.00
1	45	0.00	0.04	0.00	0.00	0.05	0.00	0.19	0.00	0.00	0.08	0.06	0.00
1	46	0.00	0.00	0.09	0.00	0.05	0.00	0.20	0.04	0.01	0.07	0.04	0.00
1	47	0.00	0.03	0.00	0.00	0.05	0.00	0.20	0.03	0.01	0.08	0.01	0.00
1	48	0.00	0.03	0.11	0.00	0.05	0.00	0.19	0.02	0.01	0.00	0.04	0.00
1	49	0.00	0.01	0.10	0.00	0.05	0.00	0.20	0.01	0.01	0.07	0.02	0.00
1	50	0.00	0.01	0.11	0.00	0.04	0.02	0.21	0.02	0.00	0.02	0.03	0.00
1	51	0.00	0.02	0.11	0.00	0.04	0.03	0.22	0.02	0.01	0.06	0.03	0.01
1	52	0.00	0.02	0.11	0.00	0.04	0.05	0.22	0.00	0.00	0.00	0.01	0.02
1	53	0.00	0.02	0.07	0.00	0.03	0.07	0.24	0.00	0.00	0.00	0.03	0.03
1	54	0.01	0.02	0.13	0.00	0.03	0.09	0.25	0.00	0.00	0.06	0.06	0.03
1	55	0.00	0.03	0.03	0.00	0.02	0.10	0.25	0.00	0.02	0.08	0.09	0.04
1	56	0.00	0.03	0.13	0.00	0.02	0.12	0.26	0.00	0.04	0.02	0.07	0.04
1	57	0.00	0.05	0.06	0.00	0.01	0.13	0.27	0.00	0.08	0.07	0.13	0.05
1	58	0.02	0.00	0.18	0.00	0.01	0.14	0.28	0.00	0.11	0.04	0.17	0.05
1	59	0.00	0.06	0.11	0.00	0.01	0.15	0.30	0.00	0.13	0.05	0.14	0.04
1	60	0.00	0.03	0.24	0.00	0.01	0.18	0.31	0.00	0.12	0.07	0.15	0.08
1	61	0.00	0.07	0.26	0.00	0.00	0.21	0.31	0.04	0.12	0.00	0.14	0.10
1	62	0.00	0.08	0.32	0.00	0.00	0.24	0.32	0.03	0.10	0.00	0.08	0.13
1	63	0.00	0.00	0.36	0.00	0.00	0.27	0.32	0.03	0.07	0.09	0.09	0.17
1	64	0.00	0.01	0.42	0.00	0.00	0.30	0.32	0.00	0.05	0.00	0.01	0.21
1	65	0.04	0.00	0.47	0.00	0.00	0.33	0.32	0.01	0.03	0.00	0.05	0.25
1	66	0.00	0.00	0.53	0.00	0.00	0.36	0.31	0.00	0.01	0.09	0.02	0.29
1	67	0.04	0.00	0.62	0.00	0.00	0.39	0.25	0.00	0.00	0.00	0.00	0.40
1	68	0.00	0.00	0.69	0.00	0.00	0.41	0.20	0.04	0.01	0.10	0.00	0.47
1	69	0.02	0.00	0.75	0.00	0.00	0.45	0.15	0.00	0.00	0.11	0.00	0.57

1	70	0.01	0.01	0.74	0.00	0.00	0.43	0.10	0.00	0.01	0.05	0.00	0.55
1	71	0.00	0.00	0.66	0.00	0.00	0.41	0.06	0.00	0.00	0.02	0.00	0.51
1	72	0.00	0.00	0.58	0.00	0.00	0.39	0.06	0.00	0.00	0.00	0.00	0.46
1	73	0.00	0.00	0.50	0.00	0.00	0.36	0.06	0.00	0.00	0.03	0.00	0.45
1	74	0.00	0.00	0.65	0.00	0.00	0.33	0.05	0.00	0.00	0.05	0.00	0.43
1	75	0.00	0.00	0.78	0.00	0.00	0.30	0.05	0.04	0.01	0.09	0.00	0.41
1	76	0.02	0.00	0.65	0.01	0.00	0.27	0.05	0.04	0.01	0.06	0.00	0.36
1	77	0.00	0.02	0.53	0.00	0.00	0.24	0.04	0.00	0.01	0.00	0.00	0.31
1	78	0.00	0.00	0.54	0.00	0.00	0.21	0.03	0.03	0.00	0.03	0.00	0.27
1	79	0.00	0.00	0.48	0.00	0.00	0.19	0.03	0.00	0.01	0.00	0.00	0.23
1	80	0.00	0.02	0.40	0.00	0.00	0.16	0.02	0.00	0.01	0.06	0.00	0.19
1	81	0.00	0.00	0.32	0.00	0.00	0.14	0.02	0.00	0.02	0.00	0.00	0.15
1	82	0.00	0.02	0.32	0.00	0.00	0.14	0.02	0.00	0.02	0.06	0.00	0.14
1	83	0.00	0.00	0.24	0.00	0.00	0.14	0.02	0.04	0.02	0.05	0.00	0.14
1	84	0.00	0.00	0.33	0.00	0.00	0.13	0.02	0.00	0.03	0.06	0.00	0.13
1	85	0.00	0.00	0.45	0.00	0.01	0.13	0.01	0.00	0.03	0.07	0.00	0.15
1	86	0.00	0.00	0.68	0.00	0.02	0.12	0.01	0.02	0.03	0.00	0.00	0.20
1	87	0.02	0.00	0.92	0.00	0.02	0.11	0.02	0.02	0.02	0.03	0.00	0.26
1	88	0.00	0.00	1.17	0.00	0.03	0.11	0.01	0.02	0.01	0.07	0.00	0.30
1	89	0.01	0.00	1.30	0.00	0.04	0.12	0.02	0.00	0.01	0.08	0.00	0.28
1	90	0.00	0.00	1.35	0.00	0.06	0.12	0.04	0.00	0.01	0.05	0.00	0.28
1	91	0.06	0.01	1.34	0.00	0.06	0.13	0.06	0.03	0.02	0.06	0.00	0.23
1	92	0.01	0.00	1.20	0.00	0.08	0.13	0.08	0.00	0.02	0.07	0.00	0.17
1	93	0.00	0.00	1.17	0.00	0.10	0.13	0.09	0.00	0.02	0.00	0.00	0.11
1	94	0.04	0.02	1.14	0.00	0.12	0.13	0.11	0.00	0.02	0.08	0.00	0.08
1	95	0.00	0.00	1.07	0.00	0.14	0.13	0.14	0.00	0.02	0.00	0.00	0.04
1	96	0.05	0.00	0.94	0.00	0.16	0.13	0.16	0.00	0.02	0.00	0.00	0.02
1	97	0.00	0.00	0.92	0.00	0.18	0.13	0.18	0.00	0.03	0.01	0.00	0.01
1	98	0.03	0.03	0.86	0.00	0.20	0.12	0.21	0.00	0.03	0.05	0.00	0.00
1	99	0.07	0.00	0.81	0.00	0.22	0.11	0.23	0.00	0.03	0.07	0.00	0.00
1	100	0.09	0.00	0.66	0.00	0.24	0.11	0.25	0.00	0.03	0.00	0.00	0.00
1	101	0.10	0.00	0.63	0.01	0.25	0.09	0.28	0.00	0.03	0.00	0.00	0.00
1	102	0.00	0.00	0.52	0.02	0.27	0.09	0.29	0.00	0.03	0.00	0.00	0.00
1	103	0.01	0.00	0.30	0.03	0.28	0.08	0.32	0.00	0.03	0.00	0.00	0.00
1	104	0.16	0.00	0.35	0.04	0.30	0.07	0.35	0.03	0.03	0.08	0.00	0.00
1	105	0.20	0.00	0.22	0.04	0.36	0.07	0.37	0.04	0.02	0.04	0.00	0.00

1	106	0.23	0.00	0.28	0.05	0.41	0.08	0.40	0.00	0.02	0.00	0.01	0.00
1	107	0.26	0.00	0.17	0.05	0.46	0.08	0.37	0.00	0.02	0.00	0.00	0.00
1	108	0.16	0.00	0.09	0.02	0.40	0.04	0.24	0.00	0.02	0.10	0.04	0.00
1	109	0.00	0.00	0.09	0.01	0.32	0.02	0.18	0.03	0.03	0.00	0.00	0.00
1	110	0.00	0.00	0.04	0.00	0.24	0.00	0.13	0.01	0.03	0.15	0.00	0.00
1	111	0.00	0.01	0.07	0.00	0.17	0.00	0.08	0.01	0.04	0.14	0.00	0.00
1	112	0.00	0.00	0.08	0.00	0.11	0.00	0.04	0.03	0.04	0.00	0.09	0.00
1	113	0.00	0.00	0.09	0.00	0.06	0.00	0.01	0.03	0.05	0.15	0.08	0.00
1	114	0.01	0.02	0.09	0.00	0.02	0.00	0.02	0.04	0.05	0.15	0.05	0.00
1	115	0.00	0.00	0.08	0.00	0.00	0.00	0.02	0.07	0.06	0.01	0.04	0.00
1	116	0.00	0.00	0.06	0.00	0.00	0.00	0.02	0.06	0.07	0.13	0.01	0.00
1	117	0.01	0.01	0.09	0.00	0.00	0.00	0.02	0.00	0.09	0.09	0.00	0.00
1	118	0.00	0.00	0.00	0.00	0.00	0.00	0.02	0.03	0.15	0.11	0.00	0.00
1	119	0.00	0.00	0.09	0.00	0.00	0.00	0.02	0.05	0.21	0.11	0.00	0.00
1	120	0.00	0.00	0.08	0.00	0.00	0.00	0.02	0.06	0.25	0.08	0.00	0.00
1	121	0.00	0.00	0.09	0.00	0.00	0.00	0.02	0.00	0.30	0.18	0.00	0.00
1	122	0.00	0.00	0.09	0.00	0.00	0.00	0.01	0.01	0.36	0.26	0.00	0.00
1	123	0.00	0.00	0.09	0.00	0.00	0.00	0.02	0.04	0.41	0.28	0.00	0.00
1	124	0.00	0.00	0.08	0.00	0.00	0.00	0.02	0.03	0.38	0.33	0.00	0.00
1	125	0.00	0.00	0.08	0.00	0.00	0.00	0.02	0.02	0.38	0.39	0.09	0.00
1	126	0.00	0.00	0.08	0.00	0.00	0.00	0.02	0.05	0.38	0.56	0.00	0.00
1	127	0.00	0.00	0.02	0.00	0.00	0.00	0.02	0.03	0.37	0.48	0.05	0.00
1	128	0.00	0.02	0.00	0.00	0.00	0.00	0.02	0.11	0.35	0.54	0.17	0.00
1	129	0.00	0.01	0.08	0.00	0.00	0.00	0.02	0.01	0.34	0.57	0.17	0.00
1	130	0.00	0.03	0.09	0.01	0.00	0.00	0.01	0.03	0.32	0.52	0.18	0.00
1	131	0.00	0.01	0.05	0.00	0.00	0.00	0.02	0.09	0.26	0.47	0.19	0.00
1	132	0.01	0.00	0.07	0.02	0.00	0.00	0.02	0.08	0.20	0.38	0.13	0.00
1	133	0.00	0.03	0.08	0.01	0.00	0.00	0.02	0.01	0.16	0.32	0.16	0.00
1	134	0.01	0.01	0.08	0.00	0.00	0.00	0.02	0.05	0.12	0.23	0.14	0.00
1	135	0.02	0.02	0.07	0.01	0.01	0.00	0.02	0.10	0.09	0.16	0.14	0.00
1	136	0.00	0.02	0.08	0.00	0.03	0.00	0.02	0.14	0.05	0.23	0.14	0.00
1	137	0.00	0.00	0.07	0.00	0.04	0.00	0.02	0.13	0.04	0.14	0.15	0.00
1	138	0.02	0.00	0.08	0.00	0.05	0.00	0.02	0.10	0.02	0.21	0.14	0.00
1	139	0.00	0.00	0.00	0.00	0.07	0.00	0.02	0.19	0.01	0.19	0.14	0.00
1	140	0.00	0.00	0.07	0.00	0.07	0.00	0.02	0.19	0.00	0.24	0.15	0.00
1	141	0.00	0.00	0.00	0.00	0.09	0.00	0.02	0.13	0.00	0.26	0.16	0.00

1	142	0.00	0.00	0.08	0.01	0.09	0.00	0.02	0.13	0.00	0.27	0.16	0.00
1	143	0.02	0.02	0.08	0.04	0.10	0.00	0.02	0.18	0.01	0.14	0.20	0.00
1	144	0.02	0.02	0.08	0.12	0.11	0.00	0.02	0.28	0.01	0.32	0.21	0.00
1	145	0.06	0.03	0.09	0.21	0.12	0.00	0.02	0.29	0.01	0.30	0.22	0.00
1	146	0.08	0.02	0.07	0.26	0.14	0.00	0.02	0.23	0.00	0.36	0.21	0.00
1	147	0.12	0.00	0.06	0.27	0.17	0.00	0.02	0.18	0.01	0.30	0.20	0.00
1	148	0.06	0.00	0.04	0.30	0.20	0.00	0.02	0.22	0.00	0.30	0.18	0.00
1	149	0.12	0.00	0.00	0.35	0.23	0.00	0.02	0.13	0.01	0.48	0.16	0.00
1	150	0.06	0.03	0.08	0.35	0.27	0.00	0.02	0.06	0.01	0.53	0.15	0.00
1	151	0.14	0.02	0.07	0.38	0.30	0.00	0.02	0.11	0.01	0.51	0.13	0.00
1	152	0.11	0.02	0.08	0.40	0.34	0.00	0.02	0.05	0.01	0.54	0.09	0.04
1	153	0.11	0.01	0.08	0.31	0.38	0.00	0.02	0.16	0.01	0.58	0.08	0.09
1	154	0.00	0.00	0.08	0.33	0.42	0.00	0.02	0.17	0.01	0.70	0.05	0.16
1	155	0.02	0.00	0.06	0.29	0.44	0.00	0.02	0.21	0.01	0.71	0.05	0.24
1	156	0.03	0.00	0.00	0.33	0.47	0.00	0.02	0.24	0.00	0.84	0.05	0.34
1	157	0.00	0.00	0.07	0.29	0.49	0.00	0.02	0.16	0.01	0.92	0.03	0.48
1	158	0.00	0.00	0.08	0.24	0.52	0.00	0.02	0.33	0.01	0.88	0.02	0.61
1	159	0.00	0.00	0.03	0.23	0.56	0.02	0.02	0.32	0.01	0.84	0.00	0.71
1	160	0.00	0.00	0.01	0.18	0.59	0.05	0.01	0.32	0.01	0.70	0.00	0.80
1	161	0.00	0.00	0.06	0.15	0.60	0.08	0.02	0.31	0.01	0.45	0.00	0.88
1	162	0.00	0.00	0.00	0.08	0.60	0.11	0.02	0.31	0.01	0.37	0.00	0.86
1	163	0.00	0.01	0.00	0.09	0.58	0.17	0.02	0.33	0.00	0.28	0.00	0.90
1	164	0.00	0.00	0.06	0.11	0.56	0.22	0.01	0.33	0.00	0.19	0.00	0.87
1	165	0.00	0.03	0.07	0.13	0.53	0.26	0.02	0.33	0.01	0.12	0.00	0.84
1	166	0.00	0.01	0.09	0.13	0.55	0.28	0.02	0.31	0.01	0.06	0.00	0.81
1	167	0.02	0.02	0.22	0.17	0.80	0.20	0.02	0.28	0.01	0.05	0.00	0.79
1	168	0.02	0.00	0.17	0.12	1.06	0.11	0.02	0.23	0.01	0.06	0.00	0.79
1	169	0.02	0.02	0.17	0.10	1.31	0.06	0.02	0.23	0.00	0.11	0.00	0.78
1	170	0.00	0.02	0.22	0.08	1.37	0.05	0.02	0.23	0.01	0.00	0.00	0.69
1	171	0.00	0.01	0.25	0.04	1.30	0.05	0.02	0.23	0.01	0.00	0.00	0.62
1	172	0.00	0.02	0.29	0.00	1.21	0.04	0.02	0.22	0.01	0.00	0.00	0.58
1	173	0.00	0.02	0.34	0.00	1.10	0.06	0.02	0.19	0.01	0.00	0.00	0.55
1	174	0.01	0.00	0.39	0.01	1.00	0.07	0.02	0.22	0.01	0.03	0.00	0.54
1	175	0.00	0.00	0.46	0.03	0.90	0.08	0.02	0.19	0.01	0.00	0.00	0.52
1	176	0.00	0.01	0.64	0.03	0.79	0.09	0.02	0.21	0.01	0.00	0.00	0.49
1	177	0.00	0.00	0.69	0.03	0.74	0.09	0.02	0.14	0.01	0.00	0.00	0.47

1	178	0.00	0.00	0.75	0.00	0.72	0.09	0.01	0.17	0.01	0.00	0.00	0.45
1	179	0.00	0.00	0.78	0.00	0.69	0.09	0.02	0.16	0.01	0.00	0.00	0.43
1	180	0.00	0.00	0.84	0.00	0.66	0.08	0.02	0.11	0.01	0.00	0.00	0.42
1	181	0.00	0.03	0.82	0.00	0.62	0.08	0.01	0.12	0.00	0.00	0.00	0.41
1	182	0.01	0.00	0.87	0.00	0.60	0.09	0.02	0.10	0.00	0.05	0.00	0.43
1	183	0.01	0.03	0.76	0.00	0.56	0.08	0.02	0.08	0.01	0.03	0.00	0.46
1	184	0.00	0.00	0.68	0.00	0.53	0.08	0.02	0.06	0.01	0.02	0.00	0.49
1	185	0.00	0.00	0.55	0.00	0.49	0.07	0.02	0.07	0.00	0.06	0.00	0.51
1	186	0.00	0.00	0.48	0.00	0.46	0.07	0.02	0.08	0.01	0.00	0.00	0.53
1	187	0.00	0.00	0.39	0.00	0.43	0.06	0.03	0.07	0.01	0.00	0.00	0.54
1	188	0.00	0.00	0.27	0.00	0.40	0.05	0.03	0.07	0.00	0.00	0.00	0.54
1	189	0.00	0.00	0.26	0.00	0.39	0.06	0.04	0.03	0.01	0.07	0.00	0.56
1	190	0.00	0.00	0.41	0.00	0.39	0.07	0.07	0.04	0.01	0.00	0.00	0.62
1	191	0.01	0.00	0.42	0.00	0.38	0.07	0.11	0.00	0.01	0.00	0.00	0.68
1	192	0.01	0.02	0.67	0.00	0.38	0.09	0.15	0.00	0.01	0.12	0.00	0.72
1	193	0.00	0.00	0.73	0.00	0.36	0.10	0.19	0.04	0.00	0.20	0.00	0.79
1	194	0.00	0.00	0.90	0.00	0.35	0.11	0.23	0.00	0.01	0.07	0.00	0.84
1	195	0.00	0.01	0.84	0.00	0.33	0.11	0.27	0.04	0.00	0.23	0.00	0.87
1	196	0.11	0.00	0.86	0.00	0.33	0.12	0.28	0.00	0.01	0.34	0.00	0.97
1	197	0.00	0.00	0.86	0.00	0.34	0.10	0.28	0.00	0.00	0.37	0.00	1.08
1	198	0.10	0.00	0.91	0.00	0.35	0.08	0.29	0.00	0.00	0.37	0.00	1.19
1	199	0.10	0.00	0.92	0.00	0.35	0.08	0.31	0.06	0.01	0.14	0.00	1.31
1	200	0.10	0.00	0.90	0.00	0.35	0.06	0.31	0.08	0.00	0.41	0.00	1.42
1	201	0.10	0.00	0.93	0.00	0.35	0.05	0.32	0.07	0.01	0.38	0.00	1.48
1	202	0.00	0.00	0.88	0.00	0.35	0.03	0.32	0.06	0.01	0.30	0.00	1.47
1	203	0.01	0.00	0.95	0.00	0.35	0.03	0.33	0.05	0.01	0.25	0.00	1.41
1	204	0.02	0.00	0.84	0.00	0.35	0.02	0.33	0.02	0.01	0.00	0.00	1.32
1	205	0.00	0.00	0.76	0.00	0.34	0.01	0.33	0.02	0.00	0.11	0.00	1.18
1	206	0.00	0.00	0.59	0.00	0.34	0.00	0.33	0.00	0.00	0.19	0.00	1.06
1	207	0.00	0.00	0.38	0.00	0.33	0.00	0.33	0.06	0.01	0.00	0.00	0.93
1	208	0.03	0.00	0.31	0.00	0.37	0.00	0.32	0.10	0.00	0.21	0.00	0.95
1	209	0.00	0.00	0.38	0.00	0.45	0.00	0.32	0.17	0.00	0.26	0.00	0.99
1	210	0.00	0.00	0.30	0.00	0.52	0.00	0.32	0.26	0.01	0.21	0.00	1.02
1	211	0.00	0.00	0.27	0.00	0.59	0.00	0.31	0.31	0.01	0.35	0.00	1.03
1	212	0.13	0.02	0.27	0.00	0.65	0.00	0.32	0.32	0.01	0.44	0.00	1.02
1	213	0.18	0.00	0.25	0.00	0.71	0.00	0.35	0.33	0.01	0.38	0.00	0.97

1	214	0.06	0.00	0.35	0.00	0.76	0.00	0.39	0.28	0.01	0.37	0.00	0.95
1	215	0.10	0.02	0.19	0.00	0.80	0.00	0.43	0.28	0.00	0.29	0.00	0.93
1	216	0.00	0.00	0.22	0.00	0.78	0.00	0.48	0.23	0.01	0.24	0.00	0.90
1	217	0.00	0.00	0.25	0.00	0.75	0.00	0.51	0.21	0.00	0.31	0.00	0.86
1	218	0.20	0.03	0.30	0.00	0.72	0.02	0.55	0.16	0.00	0.25	0.00	0.82
1	219	0.18	0.00	0.29	0.00	0.69	0.02	0.58	0.14	0.02	0.23	0.00	0.78
1	220	0.12	0.00	0.27	0.00	0.65	0.03	0.59	0.17	0.04	0.14	0.00	0.74
1	221	0.20	0.06	0.24	0.00	0.61	0.03	0.59	0.13	0.07	0.00	0.00	0.67
1	222	0.17	0.10	0.22	0.00	0.56	0.04	0.59	0.22	0.11	0.00	0.00	0.61
1	223	0.17	0.08	0.16	0.00	0.52	0.05	0.59	0.19	0.15	0.04	0.00	0.55
1	224	0.08	0.14	0.15	0.00	0.48	0.06	0.57	0.25	0.20	0.00	0.00	0.48
1	225	0.21	0.00	0.11	0.00	0.43	0.06	0.57	0.19	0.24	0.00	0.00	0.42
1	226	0.16	0.20	0.12	0.00	0.39	0.07	0.61	0.26	0.29	0.00	0.00	0.41
1	227	0.13	0.12	0.11	0.00	0.34	0.07	0.68	0.22	0.34	0.00	0.00	0.39
1	228	0.14	0.22	0.11	0.00	0.30	0.08	0.75	0.23	0.38	0.00	0.00	0.37
1	229	0.21	0.20	0.10	0.00	0.29	0.07	0.82	0.12	0.36	0.07	0.00	0.34
1	230	0.30	0.23	0.12	0.00	0.29	0.06	0.86	0.09	0.32	0.00	0.00	0.31
1	231	0.30	0.17	0.11	0.00	0.28	0.05	0.94	0.22	0.28	0.00	0.00	0.29
1	232	0.09	0.20	0.11	0.00	0.28	0.04	0.99	0.20	0.24	0.10	0.00	0.27
1	233	0.38	0.14	0.12	0.00	0.27	0.03	1.04	0.10	0.20	0.04	0.00	0.24
1	234	0.40	0.16	0.01	0.00	0.26	0.02	1.07	0.11	0.16	0.00	0.01	0.20
1	235	0.35	0.13	0.15	0.00	0.25	0.01	1.11	0.24	0.12	0.00	0.00	0.21
1	236	0.39	0.06	0.16	0.00	0.24	0.00	1.14	0.19	0.08	0.00	0.00	0.22
1	237	0.27	0.06	0.12	0.00	0.23	0.00	1.13	0.14	0.05	0.01	0.00	0.21
1	238	0.15	0.04	0.07	0.00	0.22	0.00	1.15	0.13	0.02	0.00	0.00	0.19
1	239	0.00	0.05	0.18	0.00	0.21	0.00	1.14	0.06	0.01	0.00	0.00	0.20
1	240	0.04	0.00	0.20	0.00	0.20	0.00	1.12	0.05	0.00	0.00	0.00	0.23
1	241	0.04	0.03	0.50	0.00	0.18	0.00	1.09	0.00	0.00	0.03	0.00	0.26
1	242	0.00	0.02	0.62	0.00	0.17	0.00	1.07	0.06	0.00	0.08	0.00	0.32
1	243	0.02	0.00	0.96	0.00	0.17	0.00	1.04	0.06	0.00	0.07	0.00	0.35
1	244	0.11	0.03	1.24	0.00	0.16	0.00	1.00	0.02	0.00	0.00	0.00	0.40
1	245	0.16	0.03	1.39	0.00	0.16	0.00	0.98	0.03	0.00	0.05	0.00	0.43
1	246	0.27	0.07	1.39	0.00	0.15	0.00	0.97	0.05	0.01	0.00	0.00	0.48
1	247	0.24	0.10	1.43	0.00	0.14	0.00	0.95	0.03	0.01	0.00	0.02	0.53
1	248	0.32	0.00	1.39	0.01	0.13	0.00	0.93	0.05	0.01	0.03	0.04	0.57
1	249	0.20	0.11	1.40	0.03	0.12	0.00	0.91	0.05	0.01	0.00	0.06	0.61

1	250	0.35	0.13	1.38	0.04	0.11	0.01	0.88	0.05	0.01	0.05	0.07	0.63
1	251	0.42	0.27	1.33	0.05	0.10	0.01	0.85	0.00	0.01	0.07	0.08	0.68
1	252	0.40	0.34	1.24	0.07	0.09	0.01	0.81	0.00	0.01	0.00	0.04	0.70
1	253	0.44	0.30	1.10	0.07	0.08	0.03	0.78	0.00	0.01	0.00	0.12	0.73
1	254	0.40	0.32	1.18	0.07	0.07	0.03	0.75	0.00	0.01	0.00	0.11	0.77
1	255	0.36	0.31	1.15	0.05	0.06	0.04	0.70	0.00	0.00	0.00	0.11	0.82
1	256	0.00	0.27	1.05	0.02	0.06	0.05	0.67	0.04	0.00	0.00	0.07	0.85
1	257	0.00	0.21	1.03	0.07	0.05	0.06	0.64	0.04	0.00	0.00	0.05	0.87
1	258	0.04	0.28	1.04	0.04	0.03	0.02	0.63	0.00	0.00	0.00	0.12	0.87
1	259	0.28	0.31	1.07	0.08	0.02	0.03	0.60	0.04	0.00	0.09	0.12	0.85
1	260	0.26	0.36	1.24	0.12	0.00	0.01	0.57	0.00	0.02	0.00	0.14	0.84
1	261	0.21	0.37	1.33	0.17	0.00	0.00	0.54	0.03	0.02	0.03	0.16	0.83
1	262	0.25	0.39	1.39	0.21	0.00	0.00	0.50	0.03	0.04	0.13	0.21	0.82
1	263	0.21	0.37	1.35	0.23	0.00	0.00	0.45	0.03	0.04	0.00	0.20	0.79
1	264	0.30	0.25	1.16	0.24	0.00	0.00	0.42	0.00	0.04	0.24	0.19	0.76
1	265	0.11	0.27	1.21	0.23	0.00	0.00	0.42	0.00	0.03	0.17	0.17	0.71
1	266	0.17	0.16	1.07	0.20	0.00	0.00	0.41	0.00	0.02	0.24	0.13	0.67
1	267	0.00	0.21	1.02	0.18	0.00	0.00	0.40	0.00	0.02	0.22	0.14	0.62
1	268	0.13	0.18	0.96	0.18	0.00	0.00	0.39	0.00	0.02	0.24	0.15	0.57
1	269	0.05	0.00	0.88	0.16	0.00	0.00	0.38	0.00	0.01	0.21	0.13	0.51
1	270	0.08	0.14	0.79	0.16	0.00	0.00	0.36	0.00	0.01	0.12	0.12	0.45
1	271	0.01	0.12	0.75	0.15	0.00	0.00	0.35	0.00	0.01	0.19	0.07	0.40
1	272	0.03	0.07	0.70	0.13	0.00	0.00	0.32	0.00	0.01	0.20	0.07	0.33
1	273	0.00	0.00	0.73	0.16	0.00	0.00	0.30	0.00	0.01	0.19	0.07	0.33
1	274	0.00	0.08	0.77	0.19	0.00	0.00	0.28	0.00	0.01	0.00	0.09	0.33
1	275	0.00	0.00	0.76	0.00	0.00	0.00	0.26	0.00	0.01	0.07	0.07	0.33
1	276	0.00	0.00	0.73	0.00	0.00	0.00	0.25	0.00	0.00	0.10	0.12	0.34
2	0	0.00	0.00	0.37	0.00	0.00	0.00	0.00	0.00	0.11	0.00	0.00	0.00
2	1	0.00	0.00	0.37	0.00	0.00	0.00	0.00	0.00	0.11	0.00	0.00	0.00
2	2	0.00	0.00	0.37	0.00	0.00	0.00	0.00	0.00	0.11	0.00	0.00	0.00
2	3	0.00	0.02	0.37	0.04	0.00	0.00	0.02	0.00	0.10	0.00	0.00	0.00
2	4	0.00	0.00	0.26	0.06	0.00	0.00	0.02	0.00	0.09	0.09	0.00	0.00
2	5	0.00	0.00	0.41	0.07	0.00	0.00	0.02	0.00	0.10	0.05	0.00	0.00
2	6	0.00	0.03	0.39	0.09	0.00	0.00	0.02	0.03	0.10	0.06	0.00	0.00
2	7	0.01	0.00	0.36	0.08	0.00	0.00	0.02	0.00	0.10	0.06	0.00	0.00
2	8	0.00	0.02	0.29	0.07	0.00	0.00	0.02	0.04	0.10	0.03	0.00	0.01

2	9	0.00	0.00	0.40	0.05	0.00	0.00	0.02	0.04	0.11	0.01	0.00	0.01
2	10	0.00	0.00	0.30	0.05	0.00	0.00	0.02	0.00	0.11	0.05	0.00	0.01
2	11	0.00	0.03	0.35	0.05	0.00	0.00	0.02	0.00	0.11	0.00	0.00	0.00
2	12	0.00	0.00	0.38	0.03	0.00	0.00	0.02	0.04	0.11	0.00	0.00	0.03
2	13	0.00	0.00	0.40	0.02	0.00	0.00	0.02	0.00	0.11	0.00	0.00	0.05
2	14	0.00	0.01	0.35	0.00	0.00	0.00	0.02	0.00	0.11	0.00	0.00	0.08
2	15	0.00	0.03	0.41	0.00	0.00	0.00	0.02	0.02	0.11	0.06	0.00	0.10
2	16	0.00	0.00	0.32	0.00	0.00	0.00	0.02	0.00	0.11	0.07	0.00	0.13
2	17	0.00	0.00	0.42	0.00	0.00	0.00	0.02	0.04	0.11	0.04	0.00	0.17
2	18	0.02	0.00	0.45	0.00	0.00	0.00	0.02	0.00	0.11	0.05	0.00	0.20
2	19	0.00	0.00	0.44	0.00	0.00	0.00	0.02	0.00	0.10	0.02	0.00	0.22
2	20	0.00	0.00	0.51	0.00	0.00	0.00	0.01	0.00	0.11	0.06	0.00	0.24
2	21	0.00	0.03	0.48	0.00	0.00	0.00	0.02	0.03	0.11	0.02	0.00	0.30
2	22	0.00	0.01	0.42	0.00	0.00	0.00	0.02	0.07	0.10	0.03	0.00	0.34
2	23	0.00	0.01	0.40	0.00	0.00	0.00	0.01	0.23	0.10	0.03	0.00	0.41
2	24	0.01	0.03	0.41	0.00	0.00	0.00	0.02	0.28	0.12	0.09	0.00	0.50
2	25	0.00	0.00	0.40	0.00	0.00	0.00	0.02	0.33	0.13	0.00	0.00	0.59
2	26	0.02	0.00	0.39	0.00	0.00	0.00	0.02	0.43	0.15	0.12	0.00	0.66
2	27	0.00	0.00	0.41	0.00	0.00	0.00	0.02	0.50	0.14	0.09	0.00	0.71
2	28	0.00	0.00	0.37	0.00	0.00	0.00	0.02	0.56	0.15	0.06	0.00	0.75
2	29	0.00	0.00	0.39	0.00	0.00	0.00	0.02	0.62	0.17	0.10	0.00	0.78
2	30	0.00	0.00	0.36	0.00	0.00	0.00	0.01	0.62	0.14	0.00	0.00	0.78
2	31	0.00	0.03	0.39	0.00	0.00	0.00	0.02	0.66	0.11	0.12	0.00	0.80
2	32	0.01	0.00	0.38	0.00	0.00	0.00	0.02	0.76	0.08	0.04	0.00	0.80
2	33	0.00	0.00	0.35	0.00	0.00	0.00	0.01	0.86	0.05	0.04	0.00	0.81
2	34	0.00	0.00	0.29	0.00	0.00	0.00	0.02	0.93	0.03	0.08	0.00	0.79
2	35	0.00	0.03	0.41	0.00	0.00	0.00	0.02	1.00	0.01	0.11	0.00	0.74
2	36	0.00	0.00	0.41	0.00	0.00	0.00	0.01	1.09	0.01	0.05	0.00	0.73
2	37	0.01	0.00	0.43	0.00	0.00	0.00	0.02	1.17	0.01	0.00	0.00	0.71
2	38	0.00	0.00	0.43	0.00	0.00	0.00	0.01	1.22	0.00	0.08	0.00	0.69
2	39	0.00	0.00	0.42	0.00	0.00	0.02	0.02	1.29	0.01	0.08	0.00	0.65
2	40	0.00	0.00	0.43	0.00	0.00	0.02	0.01	1.32	0.00	0.08	0.00	0.59
2	41	0.00	0.00	0.33	0.00	0.00	0.04	0.01	1.30	0.00	0.02	0.00	0.53
2	42	0.01	0.00	0.50	0.00	0.00	0.05	0.02	1.28	0.01	0.08	0.00	0.46
2	43	0.02	0.00	0.58	0.00	0.00	0.06	0.02	1.19	0.00	0.00	0.00	0.42
2	44	0.00	0.00	0.60	0.00	0.00	0.07	0.02	1.10	0.01	0.00	0.00	0.36

2	45	0.00	0.00	0.65	0.00	0.00	0.07	0.02	1.00	0.01	0.00	0.00	0.29
2	46	0.00	0.00	0.69	0.00	0.00	0.09	0.01	0.88	0.00	0.04	0.00	0.20
2	47	0.02	0.02	0.70	0.00	0.00	0.09	0.01	0.74	0.01	0.00	0.00	0.16
2	48	0.00	0.00	0.71	0.00	0.00	0.10	0.02	0.58	0.01	0.00	0.00	0.11
2	49	0.01	0.01	0.72	0.00	0.01	0.10	0.01	0.46	0.01	0.00	0.00	0.08
2	50	0.02	0.00	0.61	0.00	0.02	0.10	0.02	0.42	0.01	0.05	0.00	0.06
2	51	0.00	0.00	0.74	0.00	0.04	0.11	0.03	0.48	0.00	0.05	0.00	0.03
2	52	0.00	0.00	0.79	0.00	0.06	0.11	0.04	0.44	0.01	0.08	0.00	0.03
2	53	0.00	0.00	0.83	0.00	0.09	0.10	0.03	0.54	0.01	0.05	0.00	0.03
2	54	0.00	0.02	0.82	0.00	0.11	0.09	0.04	0.52	0.00	0.02	0.00	0.02
2	55	0.00	0.03	0.88	0.00	0.15	0.08	0.04	0.50	0.01	0.05	0.00	0.02
2	56	0.00	0.00	0.91	0.00	0.17	0.07	0.05	0.43	0.01	0.00	0.00	0.01
2	57	0.03	0.00	0.88	0.00	0.20	0.06	0.06	0.39	0.01	0.08	0.00	0.02
2	58	0.03	0.00	0.82	0.00	0.24	0.05	0.06	0.32	0.01	0.04	0.00	0.01
2	59	0.04	0.00	0.70	0.00	0.26	0.05	0.07	0.23	0.01	0.08	0.00	0.02
2	60	0.06	0.00	0.85	0.00	0.28	0.05	0.08	0.15	0.01	0.07	0.00	0.01
2	61	0.06	0.00	0.84	0.01	0.31	0.06	0.06	0.13	0.01	0.08	0.00	0.03
2	62	0.07	0.02	0.75	0.00	0.33	0.06	0.04	0.10	0.01	0.00	0.00	0.04
2	63	0.00	0.00	0.81	0.04	0.36	0.06	0.03	0.03	0.01	0.04	0.00	0.05
2	64	0.06	0.03	0.79	0.02	0.38	0.06	0.02	0.00	0.01	0.02	0.00	0.07
2	65	0.04	0.03	0.71	0.10	0.41	0.07	0.02	0.10	0.00	0.07	0.00	0.10
2	66	0.13	0.00	0.74	0.12	0.43	0.07	0.02	0.07	0.01	0.04	0.00	0.13
2	67	0.13	0.00	0.61	0.15	0.45	0.07	0.02	0.07	0.01	0.07	0.00	0.14
2	68	0.14	0.00	0.64	0.19	0.51	0.07	0.02	0.05	0.00	0.00	0.00	0.15
2	69	0.14	0.00	0.61	0.27	0.59	0.07	0.02	0.00	0.01	0.07	0.00	0.15
2	70	0.15	0.00	0.67	0.27	0.67	0.07	0.02	0.00	0.01	0.07	0.00	0.19
2	71	0.11	0.00	0.66	0.25	0.75	0.06	0.02	0.00	0.01	0.03	0.00	0.22
2	72	0.17	0.00	0.78	0.25	0.83	0.06	0.02	0.02	0.01	0.03	0.00	0.25
2	73	0.17	0.00	0.77	0.23	0.87	0.06	0.02	0.04	0.01	0.06	0.00	0.29
2	74	0.02	0.00	0.73	0.19	0.88	0.07	0.03	0.04	0.00	0.08	0.00	0.33
2	75	0.06	0.00	0.99	0.17	0.87	0.06	0.04	0.05	0.00	0.07	0.00	0.38
2	76	0.28	0.00	1.04	0.15	0.81	0.09	0.06	0.06	0.00	0.07	0.00	0.43
2	77	0.27	0.00	0.87	0.10	0.79	0.09	0.06	0.06	0.01	0.08	0.00	0.47
2	78	0.20	0.00	1.07	0.06	0.72	0.09	0.07	0.08	0.00	0.00	0.00	0.53
2	79	0.20	0.00	0.98	0.03	0.64	0.09	0.06	0.12	0.00	0.00	0.00	0.57
2	80	0.02	0.00	1.06	0.02	0.56	0.08	0.07	0.11	0.01	0.00	0.00	0.59

2	81	0.07	0.02	1.00	0.00	0.48	0.07	0.07	0.14	0.00	0.00	0.00	0.60
2	82	0.10	0.01	0.88	0.00	0.39	0.06	0.06	0.28	0.01	0.02	0.00	0.57
2	83	0.04	0.00	0.91	0.00	0.32	0.04	0.05	0.42	0.00	0.00	0.00	0.53
2	84	0.09	0.00	0.98	0.00	0.26	0.03	0.05	0.61	0.01	0.05	0.00	0.52
2	85	0.05	0.00	1.05	0.00	0.26	0.04	0.05	0.73	0.01	0.08	0.00	0.48
2	86	0.01	0.00	1.21	0.00	0.29	0.06	0.05	0.75	0.00	0.00	0.00	0.48
2	87	0.03	0.00	1.33	0.00	0.32	0.08	0.05	0.77	0.00	0.09	0.00	0.48
2	88	0.02	0.00	1.46	0.00	0.34	0.11	0.05	0.79	0.00	0.12	0.00	0.52
2	89	0.07	0.02	1.55	0.00	0.35	0.12	0.06	0.79	0.01	0.01	0.00	0.58
2	90	0.07	0.02	1.53	0.00	0.36	0.14	0.05	0.82	0.01	0.08	0.00	0.54
2	91	0.06	0.03	1.37	0.00	0.35	0.15	0.05	0.85	0.01	0.09	0.00	0.42
2	92	0.08	0.02	1.22	0.00	0.34	0.16	0.04	0.86	0.01	0.03	0.00	0.37
2	93	0.12	0.00	1.04	0.00	0.32	0.18	0.05	0.83	0.01	0.04	0.00	0.27
2	94	0.05	0.01	0.84	0.00	0.30	0.19	0.05	0.86	0.00	0.05	0.00	0.19
2	95	0.13	0.00	0.78	0.00	0.28	0.21	0.05	0.79	0.01	0.06	0.00	0.12
2	96	0.12	0.00	0.58	0.00	0.26	0.22	0.05	0.78	0.01	0.12	0.00	0.07
2	97	0.09	0.00	0.53	0.01	0.23	0.23	0.05	0.78	0.01	0.00	0.00	0.03
2	98	0.16	0.00	0.55	0.01	0.21	0.23	0.06	0.66	0.00	0.00	0.00	0.02
2	99	0.19	0.00	0.59	0.05	0.18	0.25	0.06	0.57	0.01	0.00	0.00	0.02
2	100	0.22	0.00	0.51	0.08	0.16	0.26	0.06	0.40	0.01	0.07	0.00	0.03
2	101	0.22	0.00	0.50	0.12	0.13	0.26	0.06	0.14	0.00	0.06	0.00	0.01
2	102	0.25	0.02	0.66	0.17	0.10	0.26	0.06	0.10	0.00	0.06	0.00	0.01
2	103	0.24	0.00	0.44	0.18	0.07	0.26	0.04	0.03	0.00	0.07	0.00	0.00
2	104	0.22	0.01	0.34	0.15	0.04	0.26	0.04	0.00	0.01	0.03	0.00	0.00
2	105	0.22	0.00	0.47	0.17	0.02	0.26	0.03	0.05	0.00	0.00	0.00	0.00
2	106	0.17	0.03	0.41	0.16	0.01	0.25	0.02	0.00	0.01	0.04	0.00	0.00
2	107	0.10	0.00	0.37	0.16	0.00	0.24	0.02	0.03	0.01	0.08	0.00	0.00
2	108	0.01	0.00	0.30	0.13	0.00	0.24	0.02	0.04	0.00	0.08	0.00	0.00
2	109	0.04	0.00	0.18	0.13	0.00	0.24	0.02	0.02	0.01	0.07	0.00	0.00
2	110	0.04	0.00	0.22	0.13	0.00	0.18	0.02	0.04	0.01	0.05	0.00	0.00
2	111	0.03	0.00	0.24	0.15	0.00	0.11	0.02	0.00	0.00	0.00	0.00	0.00
2	112	0.06	0.00	0.21	0.15	0.00	0.06	0.02	0.04	0.00	0.00	0.00	0.00
2	113	0.10	0.00	0.20	0.15	0.00	0.06	0.01	0.04	0.01	0.07	0.00	0.00
2	114	0.03	0.00	0.15	0.14	0.00	0.07	0.02	0.03	0.01	0.05	0.00	0.00
2	115	0.00	0.02	0.19	0.12	0.00	0.07	0.02	0.03	0.00	0.00	0.00	0.00
2	116	0.06	0.00	0.13	0.11	0.00	0.06	0.02	0.03	0.01	0.00	0.00	0.00

2	117	0.01	0.00	0.15	0.10	0.00	0.06	0.02	0.00	0.03	0.08	0.00	0.00
2	118	0.07	0.00	0.18	0.10	0.00	0.06	0.02	0.00	0.04	0.10	0.01	0.00
2	119	0.00	0.01	0.20	0.09	0.00	0.05	0.02	0.00	0.03	0.14	0.01	0.00
2	120	0.08	0.00	0.23	0.11	0.00	0.05	0.02	0.00	0.02	0.03	0.02	0.00
2	121	0.00	0.03	0.23	0.11	0.00	0.03	0.02	0.05	0.02	0.16	0.03	0.00
2	122	0.00	0.03	0.28	0.07	0.01	0.03	0.02	0.03	0.01	0.00	0.04	0.00
2	123	0.00	0.00	0.33	0.10	0.01	0.01	0.02	0.11	0.03	0.28	0.07	0.00
2	124	0.23	0.07	0.37	0.06	0.01	0.02	0.02	0.11	0.04	0.32	0.06	0.00
2	125	0.24	0.00	0.43	0.08	0.02	0.01	0.02	0.05	0.05	0.40	0.09	0.00
2	126	0.28	0.06	0.49	0.08	0.02	0.01	0.03	0.17	0.05	0.38	0.14	0.00
2	127	0.26	0.10	0.56	0.07	0.02	0.01	0.04	0.14	0.07	0.45	0.14	0.04
2	128	0.06	0.11	0.48	0.09	0.02	0.00	0.04	0.43	0.08	0.47	0.13	0.07
2	129	0.26	0.03	0.65	0.09	0.02	0.00	0.03	0.40	0.09	0.55	0.12	0.09
2	130	0.30	0.09	0.68	0.11	0.01	0.00	0.02	0.39	0.09	0.56	0.11	0.10
2	131	0.23	0.01	0.73	0.19	0.01	0.00	0.02	0.32	0.07	0.49	0.07	0.11
2	132	0.03	0.23	0.64	0.24	0.01	0.00	0.02	0.32	0.06	0.61	0.10	0.15
2	133	0.25	0.25	0.77	0.30	0.00	0.00	0.01	0.35	0.03	0.55	0.08	0.17
2	134	0.30	0.28	0.79	0.38	0.00	0.00	0.02	0.31	0.01	0.50	0.07	0.19
2	135	0.22	0.29	0.80	0.45	0.00	0.00	0.02	0.30	0.02	0.52	0.09	0.20
2	136	0.17	0.18	0.85	0.53	0.00	0.00	0.02	0.28	0.01	0.46	0.07	0.26
2	137	0.00	0.25	0.92	0.55	0.00	0.00	0.02	0.28	0.00	0.51	0.00	0.34
2	138	0.09	0.18	0.95	0.57	0.00	0.00	0.02	0.23	0.01	0.36	0.00	0.41
2	139	0.09	0.13	0.83	0.59	0.00	0.00	0.02	0.23	0.00	0.38	0.00	0.44
2	140	0.03	0.11	0.81	0.62	0.00	0.00	0.02	0.23	0.00	0.51	0.00	0.46
2	141	0.13	0.01	0.78	0.62	0.00	0.00	0.02	0.25	0.00	0.43	0.00	0.48
2	142	0.11	0.10	0.81	0.56	0.00	0.00	0.02	0.24	0.00	0.55	0.00	0.52
2	143	0.00	0.00	0.70	0.62	0.00	0.00	0.02	0.20	0.00	0.53	0.00	0.54
2	144	0.00	0.07	0.87	0.59	0.00	0.00	0.02	0.20	0.00	0.55	0.00	0.61
2	145	0.00	0.03	0.91	0.53	0.00	0.00	0.02	0.21	0.01	0.54	0.00	0.67
2	146	0.00	0.00	0.78	0.49	0.00	0.00	0.02	0.21	0.00	0.51	0.00	0.71
2	147	0.02	0.00	0.85	0.40	0.00	0.00	0.02	0.15	0.00	0.50	0.00	0.70
2	148	0.02	0.00	0.86	0.30	0.00	0.00	0.01	0.14	0.02	0.36	0.00	0.68
2	149	0.01	0.01	0.76	0.23	0.00	0.00	0.02	0.10	0.04	0.43	0.00	0.64
2	150	0.00	0.03	0.68	0.28	0.00	0.00	0.02	0.08	0.06	0.36	0.00	0.56
2	151	0.01	0.00	0.56	0.22	0.00	0.00	0.02	0.07	0.05	0.29	0.00	0.47
2	152	0.00	0.00	0.47	0.15	0.00	0.00	0.02	0.06	0.02	0.22	0.00	0.37

2	153	0.01	0.00	0.39	0.11	0.00	0.00	0.02	0.02	0.03	0.17	0.00	0.32
2	154	0.00	0.00	0.28	0.08	0.00	0.00	0.02	0.00	0.03	0.18	0.00	0.29
2	155	0.00	0.00	0.13	0.02	0.00	0.00	0.02	0.00	0.03	0.15	0.00	0.24
2	156	0.02	0.00	0.09	0.02	0.00	0.00	0.02	0.00	0.03	0.04	0.00	0.22
2	157	0.00	0.00	0.00	0.00	0.00	0.00	0.01	0.00	0.03	0.12	0.00	0.18
2	158	0.00	0.00	0.00	0.00	0.00	0.00	0.02	0.02	0.03	0.07	0.00	0.17
2	159	0.00	0.00	0.00	0.00	0.00	0.00	0.02	0.00	0.03	0.07	0.00	0.18
2	160	0.00	0.00	0.00	0.00	0.00	0.00	0.02	0.00	0.03	0.15	0.00	0.20
2	161	0.00	0.00	0.00	0.00	0.00	0.00	0.02	0.00	0.03	0.14	0.00	0.21
2	162	0.00	0.00	0.00	0.00	0.01	0.00	0.02	0.00	0.03	0.07	0.00	0.22
2	163	0.02	0.00	0.09	0.01	0.01	0.00	0.02	0.04	0.02	0.00	0.00	0.29
2	164	0.00	0.02	0.00	0.04	0.01	0.01	0.02	0.04	0.02	0.13	0.00	0.33
2	165	0.00	0.00	0.11	0.08	0.02	0.02	0.02	0.03	0.02	0.04	0.00	0.34
2	166	0.00	0.00	0.09	0.12	0.02	0.03	0.02	0.00	0.01	0.00	0.00	0.34
2	167	0.00	0.01	0.11	0.17	0.01	0.02	0.02	0.00	0.00	0.08	0.00	0.32
2	168	0.01	0.00	0.11	0.21	0.01	0.03	0.01	0.00	0.00	0.03	0.00	0.30
2	169	0.01	0.00	0.01	0.22	0.00	0.02	0.02	0.00	0.01	0.07	0.00	0.32
2	170	0.00	0.00	0.07	0.27	0.00	0.03	0.02	0.04	0.00	0.04	0.00	0.33
2	171	0.03	0.00	0.08	0.32	0.00	0.03	0.02	0.04	0.01	0.05	0.00	0.35
2	172	0.04	0.00	0.06	0.31	0.00	0.03	0.02	0.00	0.01	0.08	0.00	0.39
2	173	0.00	0.00	0.08	0.29	0.00	0.03	0.02	0.02	0.01	0.00	0.00	0.42
2	174	0.00	0.00	0.09	0.27	0.00	0.04	0.02	0.00	0.01	0.08	0.00	0.43
2	175	0.01	0.02	0.09	0.26	0.00	0.04	0.02	0.00	0.01	0.00	0.00	0.41
2	176	0.04	0.00	0.08	0.30	0.00	0.05	0.02	0.00	0.00	0.09	0.00	0.40
2	177	0.03	0.00	0.09	0.31	0.00	0.04	0.02	0.03	0.00	0.04	0.00	0.38
2	178	0.00	0.00	0.09	0.33	0.00	0.07	0.02	0.00	0.01	0.12	0.00	0.39
2	179	0.03	0.00	0.07	0.32	0.00	0.11	0.02	0.00	0.01	0.00	0.00	0.40
2	180	0.00	0.03	0.05	0.31	0.00	0.18	0.02	0.00	0.01	0.10	0.00	0.39
2	181	0.00	0.00	0.08	0.28	0.00	0.28	0.02	0.04	0.01	0.15	0.01	0.35
2	182	0.00	0.10	0.09	0.26	0.00	0.41	0.02	0.04	0.02	0.00	0.00	0.35
2	183	0.00	0.12	0.08	0.24	0.00	0.56	0.02	0.00	0.02	0.27	0.03	0.35
2	184	0.00	0.00	0.08	0.28	0.00	0.72	0.01	0.00	0.01	0.24	0.07	0.34
2	185	0.16	0.12	0.07	0.33	0.00	0.87	0.02	0.01	0.02	0.24	0.10	0.32
2	186	0.13	0.00	0.08	0.38	0.00	1.04	0.02	0.04	0.02	0.41	0.14	0.31
2	187	0.09	0.04	0.01	0.42	0.00	1.19	0.02	0.04	0.02	0.54	0.17	0.29
2	188	0.18	0.01	0.08	0.46	0.00	1.32	0.02	0.04	0.02	0.59	0.11	0.26

2	189	0.57	0.12	0.04	0.74	0.00	1.47	0.01	0.00	0.03	0.70	0.24	0.24
2	190	0.66	0.12	0.16	0.85	0.00	1.63	0.02	0.00	0.03	0.73	0.20	0.27
2	191	0.75	0.09	0.17	0.86	0.00	1.78	0.02	0.00	0.03	0.67	0.18	0.33
2	192	0.79	0.08	0.11	0.87	0.00	1.90	0.02	0.04	0.02	0.65	0.17	0.38
2	193	0.83	0.10	0.28	0.88	0.00	2.02	0.02	0.04	0.02	0.64	0.13	0.44
2	194	0.80	0.08	0.35	0.92	0.00	2.09	0.02	0.00	0.02	0.59	0.09	0.51
2	195	0.81	0.05	0.39	0.85	0.00	2.19	0.02	0.00	0.03	0.50	0.04	0.56
2	196	0.87	0.00	0.42	0.90	0.00	2.27	0.02	0.00	0.05	0.41	0.05	0.56
2	197	0.89	0.00	0.46	0.95	0.00	2.36	0.01	0.00	0.04	0.35	0.02	0.58
2	198	0.91	0.00	0.45	1.00	0.00	2.40	0.02	0.00	0.03	0.22	0.01	0.60
2	199	0.75	0.03	0.58	0.91	0.00	2.42	0.02	0.00	0.01	0.12	0.00	0.63
2	200	0.59	0.00	0.59	0.77	0.00	2.41	0.02	0.00	0.00	0.00	0.00	0.65
2	201	0.49	0.00	0.52	0.63	0.00	2.41	0.02	0.00	0.01	0.00	0.00	0.67
2	202	0.34	0.00	0.67	0.46	0.00	2.37	0.02	0.00	0.01	0.03	0.00	0.67
2	203	0.30	0.00	0.61	0.35	0.00	2.31	0.02	0.00	0.01	0.01	0.00	0.68
2	204	0.26	0.00	0.53	0.30	0.00	2.24	0.02	0.00	0.00	0.00	0.00	0.69
2	205	0.06	0.00	0.57	0.19	0.00	2.16	0.02	0.00	0.01	0.00	0.00	0.69
2	206	0.14	0.00	0.53	0.20	0.00	2.17	0.02	0.00	0.01	0.03	0.00	0.67
2	207	0.11	0.00	0.49	0.14	0.00	2.20	0.03	0.01	0.01	0.02	0.00	0.65
2	208	0.07	0.00	0.44	0.07	0.00	2.18	0.03	0.01	0.00	0.01	0.00	0.63
2	209	0.00	0.00	0.36	0.03	0.00	2.11	0.03	0.00	0.00	0.03	0.00	0.58
2	210	0.04	0.00	0.32	0.00	0.00	2.01	0.04	0.04	0.00	0.00	0.00	0.58
2	211	0.01	0.01	0.29	0.00	0.00	1.86	0.04	0.04	0.00	0.04	0.00	0.58
2	212	0.04	0.00	0.26	0.02	0.00	1.66	0.04	0.04	0.01	0.07	0.00	0.61
2	213	0.12	0.02	0.22	0.03	0.00	1.45	0.04	0.04	0.03	0.07	0.00	0.62
2	214	0.11	0.00	0.20	0.07	0.00	1.21	0.04	0.00	0.09	0.05	0.00	0.65
2	215	0.22	0.00	0.22	0.15	0.00	1.19	0.04	0.04	0.15	0.16	0.01	0.79
2	216	0.31	0.00	0.31	0.23	0.00	1.24	0.04	0.00	0.11	0.04	0.00	0.95
2	217	0.06	0.00	0.30	0.23	0.00	1.29	0.04	0.00	0.08	0.19	0.00	1.03
2	218	0.15	0.00	0.22	0.22	0.00	1.32	0.05	0.00	0.10	0.16	0.00	1.10
2	219	0.16	0.00	0.12	0.21	0.00	1.34	0.04	0.00	0.13	0.32	0.05	1.16
2	220	0.32	0.33	0.15	0.18	0.00	1.34	0.05	0.04	0.22	0.45	0.14	1.20
2	221	0.19	0.55	0.23	0.14	0.00	1.34	0.05	0.00	0.34	0.55	0.28	1.22
2	222	0.35	0.67	0.22	0.13	0.00	1.31	0.06	0.00	0.42	0.72	0.32	1.29
2	223	0.37	0.74	0.14	0.14	0.00	1.28	0.06	0.04	0.49	0.84	0.47	1.39
2	224	0.45	0.45	0.26	0.13	0.00	1.26	0.06	0.00	0.47	0.96	0.53	1.46

2	225	0.43	0.72	0.29	0.17	0.00	1.32	0.07	0.03	0.40	0.98	0.48	1.48
2	226	0.38	0.74	0.17	0.14	0.00	1.38	0.08	0.02	0.36	0.88	0.42	1.47
2	227	0.39	0.71	0.22	0.12	0.00	1.43	0.09	0.04	0.36	0.86	0.39	1.42
2	228	0.39	0.59	0.21	0.08	0.00	1.45	0.09	0.04	0.35	0.99	0.35	1.33
2	229	0.36	0.67	0.14	0.07	0.00	1.46	0.10	0.00	0.37	0.96	0.33	1.24
2	230	0.33	0.62	0.17	0.03	0.00	1.46	0.10	0.00	0.32	0.89	0.28	1.11
2	231	0.28	0.52	0.19	0.03	0.00	1.43	0.10	0.00	0.28	0.64	0.27	0.94
2	232	0.00	0.51	0.15	0.00	0.00	1.39	0.10	0.00	0.22	0.73	0.24	0.80
2	233	0.24	0.45	0.13	0.02	0.00	1.33	0.10	0.04	0.18	0.57	0.22	0.67
2	234	0.16	0.29	0.01	0.01	0.00	1.27	0.09	0.03	0.15	0.57	0.19	0.57
2	235	0.02	0.53	0.02	0.01	0.00	1.19	0.09	0.01	0.08	0.55	0.13	0.50
2	236	0.10	0.45	0.03	0.00	0.00	1.14	0.09	0.00	0.04	0.46	0.04	0.46
2	237	0.05	0.44	0.04	0.01	0.00	1.14	0.08	0.02	0.00	0.44	0.09	0.46
2	238	0.10	0.40	0.00	0.01	0.00	1.15	0.08	0.02	0.02	0.28	0.06	0.48
2	239	0.07	0.34	0.00	0.01	0.00	1.15	0.08	0.04	0.01	0.35	0.06	0.44
2	240	0.10	0.47	0.00	0.00	0.00	1.17	0.08	0.04	0.01	0.24	0.06	0.44
2	241	0.04	0.50	0.08	0.00	0.00	1.21	0.09	0.04	0.01	0.43	0.07	0.41
2	242	0.00	0.62	0.00	0.00	0.00	1.23	0.10	0.04	0.03	0.23	0.13	0.40
2	243	0.09	0.51	0.08	0.00	0.00	1.27	0.11	0.04	0.06	0.44	0.16	0.38
2	244	0.05	0.56	0.07	0.00	0.00	1.28	0.11	0.04	0.09	0.50	0.26	0.35
2	245	0.05	0.58	0.03	0.00	0.00	1.28	0.12	0.04	0.10	0.53	0.32	0.33
2	246	0.00	0.66	0.00	0.00	0.04	1.26	0.13	0.00	0.06	0.35	0.32	0.30
2	247	0.00	0.81	0.09	0.00	0.07	1.22	0.14	0.04	0.03	0.33	0.32	0.27
2	248	0.02	0.82	0.09	0.00	0.12	1.19	0.15	0.00	0.01	0.43	0.34	0.24
2	249	0.04	0.94	0.08	0.00	0.16	1.17	0.16	0.00	0.01	0.44	0.36	0.21
2	250	0.06	1.03	0.00	0.00	0.21	1.16	0.17	0.00	0.02	0.63	0.46	0.20
2	251	0.23	1.13	0.08	0.00	0.26	1.14	0.17	0.04	0.03	0.74	0.50	0.19
2	252	0.27	1.19	0.09	0.00	0.32	1.10	0.17	0.00	0.04	0.66	0.55	0.18
2	253	0.35	1.18	0.08	0.00	0.38	1.07	0.17	0.04	0.05	0.87	0.54	0.18
2	254	0.33	1.28	0.09	0.00	0.44	1.02	0.17	0.02	0.05	0.81	0.66	0.17
2	255	0.28	1.28	0.00	0.00	0.51	0.96	0.17	0.02	0.06	0.78	0.66	0.16
2	256	0.35	1.30	0.09	0.00	0.57	0.89	0.17	0.04	0.07	1.05	0.73	0.15
2	257	0.26	1.32	0.09	0.00	0.63	0.81	0.17	0.04	0.08	1.10	0.76	0.13
2	258	0.25	1.14	0.09	0.00	0.69	0.73	0.17	0.03	0.06	1.12	0.70	0.12
2	259	0.30	1.24	0.08	0.00	0.72	0.69	0.17	0.04	0.06	1.20	0.71	0.10
2	260	0.27	1.33	0.08	0.00	0.00	0.00	0.18	0.06	0.00	1.14	0.73	0.15

2	261	0.29	1.36	0.00	0.00	0.00	0.00	0.19	0.00	0.00	1.16	0.70	0.22
2	262	0.59	1.42	0.08	0.00	0.00	0.00	0.19	0.00	0.00	1.22	0.69	0.22
2	263	0.63	1.40	0.08	0.00	0.00	0.00	0.20	0.00	0.00	1.04	0.61	0.21
2	264	0.59	1.36	0.09	0.00	0.00	0.00	0.19	0.00	0.00	1.14	0.63	0.20
2	265	0.67	1.45	0.07	0.00	0.00	0.00	0.19	0.00	0.00	1.19	0.64	0.20
3	1	0.00	0.00	0.09	0.00	0.00	0.00	0.00	0.00	0.01	0.00	0.00	0.00
3	2	0.01	0.00	0.09	0.00	0.00	0.00	0.02	0.01	0.01	0.01	0.00	0.00
3	3	0.01	0.00	0.08	0.00	0.00	0.00	0.02	0.00	0.01	0.04	0.00	0.00
3	4	0.01	0.00	0.08	0.00	0.00	0.00	0.02	0.02	0.01	0.07	0.00	0.00
3	5	0.00	0.00	0.04	0.00	0.02	0.00	0.01	0.00	0.01	0.00	0.00	0.00
3	6	0.00	0.00	0.07	0.00	0.02	0.00	0.00	0.03	0.01	0.05	0.00	0.00
3	7	0.02	0.03	0.08	0.00	0.02	0.00	0.02	0.04	0.01	0.00	0.00	0.00
3	8	0.00	0.00	0.08	0.00	0.02	0.00	0.02	0.04	0.01	0.06	0.00	0.00
3	9	0.00	0.00	0.08	0.00	0.02	0.00	0.02	0.00	0.01	0.00	0.00	0.00
3	10	0.00	0.00	0.09	0.00	0.02	0.00	0.02	0.00	0.01	0.00	0.00	0.01
3	11	0.00	0.01	0.09	0.00	0.02	0.00	0.02	0.00	0.01	0.05	0.00	0.01
3	12	0.00	0.00	0.03	0.00	0.02	0.00	0.02	0.00	0.01	0.07	0.00	0.00
3	13	0.00	0.00	0.09	0.00	0.02	0.00	0.02	0.02	0.02	0.00	0.00	0.00
3	14	0.00	0.00	0.09	0.00	0.02	0.00	0.02	0.06	0.02	0.00	0.00	0.01
3	15	0.00	0.03	0.00	0.00	0.01	0.00	0.02	0.09	0.01	0.03	0.00	0.02
3	16	0.00	0.00	0.08	0.00	0.01	0.01	0.00	0.16	0.02	0.04	0.00	0.03
3	17	0.00	0.01	0.06	0.00	0.01	0.02	0.00	0.11	0.02	0.00	0.00	0.03
3	18	0.00	0.00	0.09	0.00	0.01	0.02	0.02	0.10	0.01	0.00	0.00	0.06
3	19	0.00	0.00	0.06	0.00	0.01	0.02	0.02	0.10	0.01	0.04	0.00	0.08
3	20	0.00	0.00	0.07	0.00	0.01	0.01	0.02	0.06	0.01	0.06	0.00	0.08
3	21	0.00	0.01	0.08	0.00	0.01	0.04	0.02	0.06	0.01	0.03	0.00	0.06
3	22	0.00	0.00	0.09	0.00	0.02	0.04	0.02	0.01	0.01	0.07	0.00	0.04
3	23	0.00	0.00	0.09	0.00	0.02	0.06	0.02	0.00	0.01	0.04	0.00	0.02
3	24	0.00	0.01	0.08	0.00	0.02	0.08	0.02	0.01	0.00	0.05	0.00	0.02
3	25	0.00	0.02	0.08	0.00	0.03	0.10	0.02	0.00	0.00	0.06	0.00	0.02
3	26	0.00	0.03	0.00	0.00	0.03	0.11	0.02	0.01	0.01	0.00	0.00	0.02
3	27	0.00	0.00	0.00	0.00	0.03	0.15	0.02	0.00	0.01	0.00	0.00	0.03
3	28	0.00	0.00	0.01	0.00	0.03	0.17	0.02	0.04	0.01	0.00	0.00	0.04
3	29	0.01	0.01	0.05	0.00	0.03	0.20	0.02	0.03	0.01	0.04	0.00	0.04
3	30	0.00	0.00	0.08	0.00	0.03	0.20	0.02	0.04	0.01	0.06	0.00	0.04
3	31	0.00	0.00	0.08	0.00	0.03	0.23	0.02	0.04	0.01	0.00	0.00	0.03

3	32	0.00	0.03	0.08	0.00	0.03	0.23	0.02	0.00	0.00	0.07	0.00	0.02
3	33	0.00	0.03	0.00	0.00	0.02	0.24	0.02	0.00	0.00	0.03	0.00	0.00
3	34	0.00	0.02	0.09	0.00	0.02	0.23	0.02	0.04	0.00	0.01	0.00	0.01
3	35	0.00	0.00	0.08	0.00	0.02	0.23	0.02	0.04	0.01	0.07	0.00	0.00
3	36	0.01	0.00	0.00	0.00	0.01	0.23	0.02	0.04	0.00	0.00	0.00	0.00
3	37	0.00	0.00	0.05	0.00	0.01	0.22	0.02	0.04	0.00	0.00	0.00	0.00
3	38	0.00	0.00	0.00	0.00	0.01	0.22	0.02	0.04	0.00	0.07	0.00	0.01
3	39	0.00	0.00	0.05	0.00	0.01	0.23	0.02	0.04	0.00	0.00	0.00	0.01
3	40	0.00	0.00	0.00	0.00	0.00	0.23	0.02	0.04	0.01	0.04	0.00	0.00
3	41	0.00	0.00	0.07	0.00	0.00	0.23	0.02	0.04	0.00	0.00	0.00	0.00
3	42	0.00	0.02	0.00	0.00	0.00	0.23	0.02	0.02	0.00	0.00	0.00	0.00
3	43	0.00	0.00	0.07	0.00	0.00	0.22	0.02	0.00	0.00	0.04	0.00	0.01
3	44	0.00	0.01	0.00	0.00	0.00	0.20	0.02	0.02	0.00	0.00	0.00	0.01
3	45	0.00	0.03	0.09	0.00	0.00	0.22	0.02	0.02	0.01	0.00	0.00	0.00
3	46	0.00	0.02	0.08	0.00	0.00	0.21	0.02	0.04	0.01	0.02	0.00	0.02
3	47	0.00	0.01	0.08	0.00	0.00	0.20	0.02	0.04	0.03	0.00	0.00	0.04
3	48	0.00	0.02	0.07	0.00	0.00	0.19	0.02	0.00	0.04	0.07	0.00	0.05
3	49	0.00	0.00	0.08	0.00	0.00	0.18	0.02	0.00	0.04	0.00	0.00	0.05
3	50	0.00	0.01	0.09	0.00	0.00	0.16	0.02	0.00	0.09	0.08	0.00	0.08
3	51	0.00	0.02	0.08	0.00	0.00	0.17	0.02	0.00	0.07	0.04	0.00	0.09
3	52	0.00	0.00	0.03	0.00	0.00	0.20	0.02	0.02	0.07	0.04	0.00	0.08
3	53	0.00	0.00	0.08	0.00	0.00	0.22	0.02	0.00	0.06	0.08	0.00	0.07
3	54	0.00	0.01	0.07	0.00	0.00	0.21	0.02	0.00	0.05	0.00	0.00	0.06
3	55	0.00	0.02	0.00	0.00	0.00	0.26	0.02	0.00	0.03	0.06	0.00	0.05
3	56	0.00	0.00	0.08	0.00	0.00	0.28	0.02	0.00	0.02	0.04	0.00	0.04
3	57	0.00	0.09	0.01	0.00	0.00	0.29	0.02	0.00	0.01	0.04	0.01	0.04
3	58	0.00	0.01	0.07	0.00	0.01	0.29	0.02	0.00	0.01	0.00	0.00	0.03
3	59	0.00	0.11	0.00	0.00	0.01	0.29	0.02	0.01	0.01	0.00	0.00	0.03
3	60	0.00	0.17	0.08	0.00	0.01	0.29	0.01	0.03	0.00	0.05	0.00	0.01
3	61	0.00	0.19	0.00	0.00	0.01	0.28	0.02	0.00	0.00	0.06	0.01	0.01
3	62	0.00	0.14	0.09	0.00	0.01	0.28	0.02	0.00	0.00	0.07	0.00	0.01
3	63	0.00	0.07	0.05	0.00	0.01	0.28	0.02	0.00	0.00	0.07	0.00	0.00
3	64	0.00	0.31	0.04	0.00	0.01	0.27	0.02	0.00	0.00	0.02	0.04	0.01
3	65	0.00	0.45	0.08	0.00	0.01	0.27	0.02	0.00	0.01	0.00	0.04	0.01
3	66	0.00	0.61	0.08	0.00	0.01	0.25	0.02	0.00	0.02	0.03	0.10	0.01
3	67	0.00	0.41	0.09	0.00	0.02	0.24	0.02	0.00	0.05	0.02	0.19	0.00

3	68	0.00	0.63	0.09	0.00	0.02	0.22	0.02	0.00	0.09	0.07	0.19	0.01
3	69	0.00	0.85	0.09	0.00	0.02	0.21	0.02	0.00	0.13	0.06	0.35	0.02
3	70	0.00	0.65	0.00	0.00	0.02	0.20	0.02	0.02	0.18	0.00	0.35	0.03
3	71	0.00	0.91	0.05	0.00	0.01	0.18	0.02	0.03	0.21	0.04	0.47	0.02
3	72	0.00	1.08	0.09	0.00	0.01	0.17	0.01	0.04	0.21	0.00	0.53	0.03
3	73	0.00	1.16	0.08	0.00	0.02	0.14	0.02	0.04	0.21	0.00	0.57	0.03
3	74	0.00	1.22	0.02	0.00	0.01	0.14	0.02	0.00	0.22	0.05	0.54	0.02
3	75	0.00	1.24	0.08	0.00	0.00	0.11	0.02	0.00	0.22	0.02	0.53	0.01
3	76	0.00	1.31	0.03	0.01	0.00	0.09	0.02	0.00	0.24	0.09	0.70	0.00
3	77	0.00	1.32	0.07	0.01	0.00	0.08	0.02	0.04	0.31	0.00	0.78	0.00
3	78	0.00	1.02	0.09	0.00	0.00	0.06	0.02	0.04	0.32	0.02	0.78	0.00
3	79	0.00	1.01	0.00	0.00	0.00	0.04	0.01	0.00	0.37	0.05	0.78	0.00
3	80	0.00	0.98	0.07	0.00	0.00	0.03	0.02	0.03	0.37	0.07	0.79	0.00
3	81	0.00	0.74	0.00	0.00	0.00	0.02	0.02	0.00	0.37	0.00	0.80	0.00
3	82	0.00	0.88	0.08	0.00	0.00	0.01	0.02	0.00	0.36	0.00	0.75	0.00
3	83	0.00	0.76	0.08	0.00	0.00	0.02	0.02	0.03	0.40	0.00	0.67	0.00
3	84	0.00	0.76	0.01	0.00	0.00	0.03	0.03	0.00	0.44	0.06	0.66	0.00
3	85	0.00	0.71	0.08	0.00	0.00	0.03	0.04	0.00	0.49	0.00	0.59	0.00
3	86	0.00	0.63	0.08	0.00	0.00	0.04	0.05	0.00	0.49	0.06	0.54	0.00
3	87	0.03	0.51	0.07	0.01	0.00	0.05	0.07	0.04	0.51	0.02	0.53	0.00
3	88	0.00	0.43	0.00	0.01	0.00	0.05	0.08	0.00	0.57	0.07	0.53	0.00
3	89	0.00	0.50	0.01	0.00	0.00	0.06	0.10	0.04	0.59	0.08	0.46	0.00
3	90	0.00	0.36	0.09	0.00	0.00	0.05	0.13	0.03	0.59	0.00	0.46	0.00
3	91	0.00	0.39	0.09	0.00	0.00	0.04	0.15	0.03	0.57	0.03	0.52	0.00
3	92	0.00	0.37	0.09	0.00	0.00	0.03	0.17	0.03	0.55	0.08	0.47	0.00
3	93	0.05	0.44	0.04	0.00	0.00	0.02	0.19	0.02	0.56	0.00	0.53	0.00
3	94	0.06	0.44	0.07	0.00	0.00	0.01	0.21	0.02	0.51	0.04	0.51	0.00
3	95	0.00	0.47	0.00	0.00	0.00	0.00	0.23	0.01	0.51	0.08	0.56	0.00
3	96	0.16	0.52	0.00	0.00	0.00	0.01	0.25	0.05	0.51	0.01	0.57	0.00
3	97	0.10	0.51	0.06	0.00	0.00	0.02	0.26	0.04	0.49	0.06	0.64	0.00
3	98	0.08	0.47	0.06	0.00	0.00	0.02	0.27	0.10	0.47	0.06	0.66	0.00
3	99	0.10	0.42	0.07	0.00	0.00	0.03	0.28	0.19	0.44	0.00	0.66	0.00
3	100	0.25	0.25	0.00	0.00	0.00	0.03	0.28	0.22	0.34	0.07	0.54	0.00
3	101	0.22	0.11	0.07	0.00	0.00	0.04	0.29	0.23	0.27	0.00	0.54	0.00
3	102	0.27	0.15	0.08	0.00	0.00	0.04	0.29	0.22	0.21	0.07	0.50	0.00
3	103	0.23	0.24	0.01	0.00	0.00	0.05	0.30	0.27	0.15	0.05	0.44	0.00

3	104	0.03	0.20	0.08	0.00	0.00	0.05	0.30	0.25	0.10	0.01	0.28	0.00
3	105	0.05	0.09	0.08	0.00	0.00	0.05	0.30	0.17	0.06	0.05	0.25	0.00
3	106	0.09	0.06	0.08	0.00	0.00	0.05	0.31	0.08	0.04	0.03	0.17	0.00
3	107	0.08	0.08	0.08	0.00	0.00	0.05	0.31	0.13	0.02	0.05	0.14	0.00
3	108	0.09	0.07	0.08	0.00	0.00	0.04	0.31	0.01	0.01	0.00	0.14	0.00
3	109	0.08	0.05	0.07	0.00	0.00	0.03	0.28	0.09	0.01	0.10	0.11	0.00
3	110	0.08	0.07	0.09	0.00	0.00	0.02	0.24	0.02	0.01	0.02	0.08	0.00
3	111	0.06	0.05	0.09	0.03	0.00	0.01	0.20	0.00	0.01	0.01	0.08	0.00
3	112	0.00	0.02	0.08	0.00	0.00	0.01	0.15	0.03	0.01	0.00	0.02	0.01
3	113	0.00	0.00	0.08	0.00	0.00	0.01	0.13	0.04	0.01	0.07	0.02	0.01
3	114	0.00	0.01	0.07	0.00	0.00	0.00	0.10	0.02	0.01	0.07	0.00	0.00
3	115	0.01	0.03	0.00	0.00	0.00	0.00	0.08	0.04	0.01	0.00	0.00	0.00
3	116	0.00	0.02	0.09	0.00	0.00	0.00	0.06	0.02	0.01	0.00	0.00	0.00
3	117	0.00	0.00	0.08	0.00	0.00	0.00	0.04	0.00	0.01	0.00	0.00	0.00
3	118	0.00	0.00	0.08	0.00	0.00	0.00	0.06	0.04	0.00	0.00	0.00	0.00
3	119	0.02	0.00	0.07	0.00	0.00	0.00	0.09	0.00	0.01	0.01	0.00	0.00
3	120	0.00	0.00	0.08	0.00	0.00	0.00	0.11	0.00	0.01	0.00	0.00	0.00
3	121	0.00	0.00	0.08	0.00	0.00	0.00	0.14	0.04	0.01	0.05	0.00	0.00
3	122	0.00	0.00	0.09	0.00	0.00	0.00	0.17	0.00	0.00	0.07	0.00	0.00
3	123	0.01	0.01	0.08	0.00	0.00	0.00	0.19	0.03	0.01	0.00	0.00	0.00
3	124	0.02	0.00	0.08	0.00	0.00	0.00	0.19	0.04	0.00	0.00	0.00	0.00
3	125	0.00	0.02	0.05	0.00	0.00	0.00	0.19	0.04	0.01	0.06	0.00	0.00
3	126	0.02	0.01	0.08	0.00	0.00	0.00	0.17	0.02	0.00	0.01	0.00	0.00
3	127	0.00	0.00	0.02	0.00	0.00	0.00	0.12	0.03	0.01	0.00	0.00	0.00
3	128	0.00	0.00	0.09	0.00	0.00	0.00	0.10	0.03	0.00	0.04	0.00	0.00
3	129	0.00	0.02	0.09	0.00	0.00	0.00	0.08	0.04	0.00	0.04	0.00	0.00
3	130	0.02	0.00	0.09	0.00	0.00	0.00	0.07	0.04	0.01	0.05	0.00	0.00
3	131	0.00	0.00	0.05	0.00	0.00	0.00	0.07	0.03	0.01	0.04	0.00	0.00
3	132	0.00	0.00	0.08	0.00	0.00	0.00	0.07	0.04	0.01	0.00	0.01	0.00
3	133	0.00	0.00	0.08	0.00	0.00	0.00	0.07	0.04	0.01	0.16	0.00	0.00
3	134	0.00	0.01	0.00	0.00	0.00	0.00	0.09	0.04	0.01	0.00	0.03	0.00
3	135	0.00	0.00	0.09	0.00	0.00	0.00	0.09	0.06	0.01	0.32	0.05	0.00
3	136	0.00	0.01	0.05	0.00	0.00	0.00	0.09	0.00	0.01	0.36	0.09	0.00
3	137	0.01	0.01	0.08	0.00	0.00	0.00	0.10	0.02	0.00	0.39	0.11	0.00
3	138	0.01	0.00	0.08	0.00	0.00	0.01	0.11	0.14	0.01	0.50	0.13	0.00
3	139	0.00	0.08	0.08	0.00	0.00	0.02	0.12	0.10	0.01	0.58	0.12	0.00

3	140	0.05	0.02	0.07	0.00	0.00	0.02	0.14	0.13	0.00	0.66	0.15	0.00
3	141	0.13	0.00	0.08	0.00	0.01	0.03	0.15	0.18	0.01	0.53	0.17	0.00
3	142	0.12	0.09	0.08	0.02	0.01	0.04	0.16	0.18	0.01	0.67	0.11	0.00
3	143	0.00	0.14	0.07	0.06	0.02	0.05	0.18	0.14	0.01	0.86	0.17	0.00
3	144	0.25	0.19	0.07	0.08	0.03	0.05	0.19	0.13	0.00	0.80	0.22	0.00
3	145	0.14	0.18	0.09	0.11	0.04	0.06	0.21	0.15	0.01	0.95	0.23	0.00
3	146	0.33	0.21	0.09	0.11	0.05	0.06	0.24	0.15	0.01	0.98	0.22	0.00
3	147	0.24	0.09	0.00	0.10	0.07	0.08	0.28	0.10	0.01	0.96	0.23	0.00
3	148	0.27	0.15	0.09	0.08	0.08	0.08	0.31	0.12	0.01	0.87	0.23	0.00
3	149	0.25	0.28	0.01	0.05	0.10	0.08	0.34	0.14	0.00	0.81	0.22	0.00
3	150	0.26	0.27	0.08	0.04	0.12	0.09	0.37	0.08	0.01	0.82	0.23	0.00
3	151	0.17	0.26	0.08	0.02	0.12	0.09	0.38	0.10	0.01	0.81	0.16	0.00
3	152	0.10	0.19	0.00	0.01	0.12	0.07	0.39	0.00	0.00	0.57	0.11	0.00
3	153	0.07	0.14	0.08	0.00	0.11	0.05	0.39	0.03	0.01	0.60	0.09	0.00
3	154	0.00	0.13	0.08	0.01	0.10	0.04	0.46	0.04	0.01	0.40	0.03	0.00
3	155	0.00	0.08	0.08	0.02	0.09	0.02	0.56	0.03	0.01	0.32	0.00	0.00
3	156	0.00	0.02	0.00	0.02	0.08	0.01	0.64	0.00	0.01	0.22	0.00	0.00
3	157	0.05	0.00	0.00	0.03	0.07	0.00	0.72	0.04	0.01	0.18	0.00	0.00
3	158	0.03	0.00	0.00	0.06	0.06	0.00	0.79	0.03	0.01	0.15	0.00	0.00
3	159	0.00	0.00	0.00	0.04	0.05	0.00	0.84	0.02	0.01	0.00	0.00	0.00
3	160	0.00	0.02	0.04	0.03	0.04	0.00	0.88	0.00	0.01	0.00	0.00	0.00
3	161	0.01	0.00	0.00	0.04	0.02	0.00	0.94	0.04	0.01	0.05	0.00	0.00
3	162	0.00	0.00	0.00	0.00	0.02	0.00	1.06	0.02	0.01	0.06	0.00	0.00
3	163	0.00	0.00	0.00	0.01	0.01	0.00	1.19	0.03	0.01	0.07	0.00	0.00
3	164	0.00	0.00	0.00	0.03	0.00	0.00	1.29	0.00	0.01	0.07	0.00	0.00
3	165	0.00	0.00	0.08	0.02	0.00	0.00	1.40	0.04	0.01	0.00	0.00	0.00
3	166	0.00	0.00	0.05	0.03	0.00	0.00	1.47	0.04	0.01	0.07	0.00	0.00
3	167	0.07	0.01	0.00	0.16	0.00	0.00	1.54	0.01	0.01	0.05	0.00	0.00
3	168	0.00	0.03	0.16	0.14	0.00	0.00	1.57	0.01	0.01	0.00	0.00	0.00
3	169	0.00	0.01	0.00	0.14	0.00	0.00	1.50	0.00	0.01	0.00	0.00	0.00
3	170	0.04	0.03	0.08	0.14	0.00	0.00	1.37	0.03	0.01	0.00	0.00	0.00
3	171	0.00	0.01	0.00	0.16	0.00	0.00	1.26	0.04	0.00	0.00	0.00	0.00
3	172	0.00	0.00	0.00	0.16	0.00	0.00	1.14	0.00	0.01	0.00	0.00	0.00
3	173	0.00	0.00	0.08	0.16	0.00	0.00	1.00	0.00	0.01	0.04	0.00	0.00
3	174	0.00	0.00	0.08	0.14	0.00	0.00	0.86	0.00	0.01	0.06	0.00	0.00
3	175	0.00	0.00	0.08	0.11	0.00	0.00	0.71	0.00	0.01	0.00	0.00	0.00

3	176	0.00	0.00	0.07	0.10	0.00	0.00	0.57	0.01	0.01	0.07	0.00	0.00
3	177	0.00	0.00	0.07	0.10	0.00	0.00	0.44	0.04	0.01	0.07	0.00	0.00
3	178	0.00	0.00	0.09	0.06	0.00	0.01	0.31	0.01	0.00	0.05	0.00	0.00
3	179	0.00	0.02	0.06	0.07	0.00	0.02	0.21	0.04	0.01	0.06	0.00	0.00
3	180	0.00	0.03	0.08	0.05	0.00	0.04	0.13	0.04	0.01	0.03	0.00	0.00
3	181	0.00	0.00	0.09	0.05	0.00	0.06	0.07	0.04	0.01	0.08	0.00	0.00
3	182	0.00	0.00	0.06	0.11	0.00	0.09	0.08	0.03	0.01	0.05	0.00	0.00
3	183	0.00	0.02	0.08	0.21	0.00	0.12	0.08	0.04	0.01	0.03	0.00	0.00
3	184	0.02	0.03	0.00	0.30	0.00	0.16	0.08	0.00	0.01	0.03	0.00	0.00
3	185	0.11	0.02	0.08	0.34	0.00	0.20	0.07	0.02	0.01	0.01	0.00	0.00
3	186	0.00	0.03	0.09	0.25	0.00	0.25	0.07	0.00	0.01	0.07	0.00	0.00
3	187	0.00	0.00	0.00	0.17	0.00	0.30	0.06	0.00	0.01	0.04	0.00	0.00
3	188	0.05	0.00	0.08	0.11	0.00	0.35	0.05	0.00	0.01	0.05	0.00	0.00
3	189	0.02	0.02	0.07	0.08	0.00	0.40	0.04	0.00	0.01	0.06	0.00	0.00
3	190	0.00	0.00	0.00	0.04	0.00	0.42	0.04	0.01	0.01	0.01	0.00	0.00
3	191	0.00	0.00	0.00	0.00	0.00	0.47	0.03	0.00	0.01	0.04	0.00	0.00
3	192	0.00	0.01	0.00	0.00	0.00	0.50	0.02	0.00	0.01	0.04	0.00	0.00
3	193	0.00	0.00	0.00	0.00	0.00	0.53	0.02	0.00	0.01	0.07	0.00	0.00
3	194	0.01	0.01	0.07	0.00	0.00	0.53	0.02	0.00	0.01	0.05	0.00	0.00
3	195	0.01	0.02	0.00	0.00	0.00	0.55	0.02	0.00	0.00	0.08	0.00	0.00
3	196	0.00	0.00	0.00	0.00	0.00	0.56	0.02	0.00	0.00	0.00	0.00	0.00
3	197	0.00	0.02	0.00	0.00	0.00	0.56	0.02	0.00	0.01	0.01	0.00	0.00
3	198	0.00	0.00	0.00	0.00	0.00	0.55	0.02	0.00	0.01	0.03	0.00	0.00
3	199	0.00	0.00	0.07	0.00	0.00	0.53	0.02	0.00	0.01	0.00	0.00	0.00
3	200	0.00	0.00	0.00	0.00	0.00	0.50	0.02	0.00	0.01	0.03	0.00	0.00
3	201	0.01	0.03	0.09	0.00	0.00	0.46	0.02	0.02	0.01	0.04	0.00	0.00
3	202	0.00	0.02	0.09	0.00	0.00	0.43	0.02	0.01	0.01	0.00	0.00	0.00
3	203	0.01	0.02	0.00	0.02	0.00	0.38	0.02	0.00	0.00	0.05	0.00	0.00
3	204	0.00	0.02	0.00	0.02	0.00	0.34	0.02	0.01	0.01	0.00	0.00	0.00
3	205	0.00	0.00	0.08	0.00	0.00	0.29	0.02	0.06	0.01	0.06	0.00	0.00
3	206	0.01	0.00	0.09	0.00	0.00	0.24	0.02	0.06	0.01	0.05	0.00	0.00
3	207	0.00	0.01	0.08	0.00	0.00	0.22	0.02	0.04	0.01	0.03	0.00	0.00
3	208	0.00	0.00	0.09	0.00	0.00	0.18	0.02	0.04	0.01	0.00	0.01	0.00
3	209	0.01	0.00	0.07	0.00	0.00	0.17	0.02	0.09	0.00	0.00	0.00	0.00
3	210	0.00	0.02	0.00	0.00	0.00	0.16	0.02	0.07	0.00	0.07	0.00	0.00
3	211	0.00	0.00	0.08	0.00	0.00	0.16	0.02	0.08	0.00	0.05	0.00	0.00

3	212	0.00	0.00	0.07	0.00	0.00	0.16	0.02	0.12	0.01	0.00	0.00	0.00
3	213	0.00	0.00	0.09	0.00	0.00	0.15	0.02	0.14	0.01	0.00	0.03	0.00
3	214	0.00	0.01	0.08	0.00	0.00	0.15	0.02	0.11	0.01	0.03	0.03	0.00
3	215	0.00	0.00	0.07	0.00	0.00	0.15	0.02	0.16	0.02	0.07	0.05	0.00
3	216	0.00	0.00	0.04	0.00	0.00	0.14	0.02	0.21	0.04	0.07	0.02	0.00
3	217	0.00	0.03	0.00	0.00	0.00	0.14	0.02	0.23	0.05	0.07	0.07	0.00
3	218	0.00	0.02	0.02	0.00	0.00	0.13	0.02	0.23	0.06	0.00	0.08	0.00
3	219	0.00	0.02	0.04	0.00	0.00	0.13	0.02	0.21	0.08	0.06	0.04	0.00
3	220	0.00	0.00	0.07	0.00	0.00	0.12	0.02	0.19	0.07	0.00	0.07	0.00
3	221	0.00	0.02	0.00	0.00	0.00	0.12	0.00	0.19	0.10	0.00	0.08	0.00
3	222	0.00	0.00	0.08	0.00	0.00	0.00	0.00	0.00	0.08	0.00	0.06	0.00
4	1	0.00	0.00	0.06	0.00	0.00	0.00	0.02	1.03	0.01	0.00	0.00	0.06
4	2	0.01	0.00	0.09	0.00	0.00	0.00	0.02	1.07	0.01	0.04	0.00	0.04
4	3	0.00	0.00	0.09	0.00	0.00	0.00	0.02	1.04	0.00	0.00	0.00	0.01
4	4	0.00	0.03	0.05	0.00	0.00	0.00	0.02	1.01	0.01	0.00	0.00	0.03
4	5	0.00	0.00	0.04	0.00	0.00	0.00	0.02	0.95	0.00	0.02	0.00	0.03
4	6	0.00	0.00	0.06	0.00	0.00	0.01	0.02	0.91	0.01	0.05	0.00	0.03
4	7	0.01	0.00	0.06	0.00	0.00	0.00	0.03	0.84	0.01	0.07	0.00	0.03
4	8	0.00	0.00	0.00	0.00	0.00	0.02	0.03	0.84	0.01	0.07	0.00	0.04
4	9	0.00	0.00	0.02	0.00	0.00	0.03	0.04	0.86	0.00	0.01	0.00	0.04
4	10	0.00	0.02	0.00	0.00	0.00	0.04	0.05	0.88	0.00	0.00	0.00	0.05
4	11	0.00	0.00	0.01	0.00	0.00	0.04	0.06	0.89	0.01	0.03	0.00	0.05
4	12	0.00	0.00	0.00	0.00	0.00	0.06	0.08	0.86	0.01	0.05	0.00	0.06
4	13	0.01	0.00	0.10	0.00	0.00	0.06	0.09	0.87	0.01	0.01	0.00	0.06
4	14	0.00	0.00	0.00	0.00	0.00	0.05	0.07	0.66	0.00	0.00	0.00	0.05
4	15	0.02	0.00	0.08	0.00	0.00	0.04	0.06	0.62	0.01	0.02	0.00	0.04
4	16	0.00	0.02	0.00	0.00	0.00	0.03	0.05	0.52	0.01	0.07	0.00	0.01
4	17	0.00	0.00	0.07	0.00	0.00	0.03	0.04	0.43	0.01	0.04	0.00	0.00
4	18	0.00	0.00	0.00	0.00	0.00	0.02	0.03	0.36	0.01	0.00	0.00	0.00
4	19	0.02	0.00	0.09	0.00	0.00	0.02	0.02	0.29	0.01	0.00	0.00	0.00
4	20	0.00	0.01	0.09	0.00	0.00	0.01	0.02	0.21	0.01	0.00	0.00	0.00
4	21	0.00	0.00	0.09	0.00	0.00	0.00	0.02	0.17	0.01	0.00	0.00	0.00
4	22	0.00	0.00	0.05	0.00	0.00	0.00	0.02	0.17	0.00	0.08	0.00	0.00
4	23	0.00	0.00	0.09	0.00	0.00	0.00	0.02	0.06	0.00	0.00	0.00	0.00
4	24	0.00	0.00	0.08	0.00	0.00	0.00	0.02	0.11	0.00	0.07	0.00	0.00
4	25	0.00	0.00	0.09	0.00	0.00	0.00	0.02	0.05	0.01	0.06	0.00	0.00

4	26	0.00	0.00	0.09	0.00	0.00	0.00	0.02	0.04	0.01	0.08	0.00	0.00
4	27	0.00	0.00	0.07	0.00	0.00	0.00	0.02	0.03	0.00	0.06	0.00	0.00
4	28	0.02	0.00	0.03	0.00	0.00	0.00	0.02	0.07	0.01	0.00	0.00	0.00
4	29	0.00	0.00	0.07	0.00	0.00	0.00	0.02	0.06	0.00	0.00	0.00	0.00
4	30	0.00	0.00	0.07	0.00	0.00	0.00	0.02	0.18	0.01	0.02	0.00	0.00
4	31	0.00	0.00	0.03	0.00	0.00	0.00	0.02	0.24	0.01	0.07	0.00	0.00
4	32	0.01	0.02	0.09	0.00	0.00	0.00	0.02	0.23	0.00	0.08	0.00	0.00
4	33	0.00	0.03	0.08	0.00	0.00	0.00	0.02	0.18	0.01	0.03	0.00	0.00
4	34	0.00	0.03	0.09	0.00	0.00	0.00	0.01	0.22	0.01	0.05	0.00	0.00
4	35	0.00	0.00	0.07	0.00	0.00	0.00	0.02	0.29	0.01	0.07	0.00	0.00
4	36	0.00	0.00	0.00	0.00	0.00	0.00	0.02	0.33	0.00	0.07	0.00	0.00
4	37	0.01	0.00	0.08	0.00	0.00	0.00	0.02	0.23	0.00	0.00	0.00	0.00
4	38	0.00	0.00	0.08	0.00	0.00	0.01	0.02	0.22	0.01	0.06	0.00	0.02
4	39	0.00	0.00	0.07	0.00	0.00	0.02	0.03	0.26	0.01	0.00	0.00	0.06
4	40	0.00	0.00	0.08	0.00	0.00	0.04	0.04	0.30	0.00	0.00	0.00	0.11
4	41	0.00	0.00	0.17	0.00	0.00	0.06	0.07	0.33	0.01	0.08	0.00	0.18
4	42	0.01	0.00	0.20	0.00	0.00	0.10	0.09	0.36	0.01	0.08	0.00	0.27
4	43	0.00	0.00	0.45	0.00	0.00	0.14	0.12	0.41	0.01	0.02	0.00	0.36
4	44	0.08	0.00	0.42	0.00	0.00	0.18	0.15	0.45	0.01	0.09	0.00	0.45
4	45	0.13	0.00	0.86	0.00	0.00	0.22	0.19	0.46	0.00	0.05	0.00	0.53
4	46	0.10	0.02	1.05	0.00	0.00	0.28	0.22	0.57	0.01	0.10	0.00	0.62
4	47	0.14	0.02	1.19	0.00	0.00	0.32	0.24	0.54	0.01	0.06	0.00	0.71
4	48	0.25	0.00	1.38	0.00	0.00	0.33	0.24	0.54	0.01	0.12	0.00	0.76
4	49	0.31	0.00	1.25	0.00	0.00	0.33	0.24	0.76	0.01	0.21	0.00	0.77
4	50	0.24	0.03	1.27	0.00	0.01	0.33	0.24	0.84	0.01	0.13	0.00	0.76
4	51	0.30	0.04	1.21	0.00	0.02	0.33	0.23	1.02	0.01	0.26	0.00	0.75
4	52	0.28	0.04	1.25	0.00	0.04	0.32	0.21	1.13	0.01	0.32	0.00	0.69
4	53	0.30	0.06	1.18	0.00	0.07	0.32	0.20	1.18	0.01	0.31	0.00	0.58
4	54	0.31	0.06	1.17	0.00	0.11	0.31	0.18	1.23	0.01	0.28	0.00	0.47
4	55	0.36	0.00	1.19	0.02	0.16	0.31	0.21	1.17	0.01	0.30	0.00	0.43
4	56	0.42	0.00	1.25	0.03	0.21	0.31	0.25	1.02	0.01	0.28	0.00	0.42
4	57	0.47	0.00	1.25	0.03	0.26	0.30	0.30	1.04	0.01	0.27	0.00	0.40
4	58	0.36	0.11	1.22	0.07	0.32	0.29	0.35	0.93	0.01	0.26	0.00	0.38
4	59	0.54	0.12	1.30	0.09	0.38	0.28	0.41	0.73	0.00	0.23	0.00	0.36
4	60	0.61	0.00	1.39	0.10	0.43	0.27	0.46	0.53	0.01	0.22	0.00	0.32
4	61	0.50	0.15	1.28	0.07	0.45	0.27	0.50	0.61	0.01	0.18	0.00	0.28

4	62	0.51	0.00	1.16	0.07	0.46	0.26	0.53	0.56	0.01	0.18	0.00	0.24
4	63	0.37	0.05	1.03	0.05	0.46	0.24	0.55	0.55	0.00	0.11	0.00	0.19
4	64	0.32	0.00	0.87	0.03	0.48	0.22	0.56	0.43	0.00	0.07	0.00	0.13
4	65	0.27	0.01	0.62	0.02	0.48	0.19	0.55	0.44	0.00	0.02	0.00	0.10
4	66	0.10	0.08	0.59	0.00	0.48	0.16	0.55	0.31	0.01	0.00	0.00	0.05
4	67	0.08	0.05	0.48	0.00	0.47	0.12	0.51	0.24	0.01	0.03	0.00	0.00
4	68	0.04	0.10	0.14	0.00	0.46	0.09	0.49	0.22	0.01	0.00	0.00	0.00
4	69	0.00	0.15	0.10	0.00	0.45	0.06	0.46	0.18	0.01	0.00	0.00	0.00
4	70	0.00	0.17	0.17	0.00	0.43	0.03	0.42	0.15	0.01	0.02	0.00	0.00
4	71	0.00	0.00	0.02	0.00	0.40	0.01	0.37	0.14	0.01	0.07	0.00	0.00
4	72	0.00	0.19	0.09	0.00	0.38	0.00	0.32	0.15	0.01	0.06	0.00	0.00
4	73	0.00	0.21	0.07	0.00	0.36	0.00	0.28	0.16	0.00	0.02	0.00	0.00
4	74	0.00	0.00	0.00	0.00	0.32	0.01	0.26	0.15	0.01	0.03	0.00	0.00
4	75	0.00	0.00	0.04	0.00	0.31	0.00	0.24	0.16	0.00	0.05	0.00	0.00
4	76	0.00	0.25	0.00	0.00	0.27	0.02	0.19	0.10	0.01	0.05	0.00	0.00
4	77	0.01	0.19	0.09	0.00	0.24	0.04	0.13	0.15	0.01	0.03	0.00	0.00
4	78	0.00	0.00	0.07	0.00	0.21	0.05	0.09	0.12	0.00	0.04	0.00	0.00
4	79	0.00	0.25	0.01	0.00	0.18	0.07	0.06	0.14	0.00	0.03	0.00	0.00
4	80	0.00	0.22	0.07	0.00	0.15	0.08	0.05	0.13	0.01	0.03	0.00	0.00
4	81	0.00	0.00	0.03	0.00	0.18	0.07	0.07	0.11	0.01	0.01	0.00	0.00
4	82	0.01	0.00	0.09	0.00	0.21	0.07	0.09	0.04	0.00	0.06	0.00	0.00
4	83	0.00	0.00	0.00	0.00	0.23	0.06	0.12	0.06	0.01	0.08	0.00	0.00
4	84	0.00	0.00	0.06	0.00	0.25	0.05	0.15	0.06	0.01	0.05	0.00	0.00
4	85	0.00	0.20	0.00	0.00	0.27	0.03	0.18	0.09	0.00	0.05	0.01	0.00
4	86	0.00	0.08	0.00	0.00	0.28	0.03	0.21	0.06	0.01	0.07	0.01	0.00
4	87	0.00	0.16	0.00	0.00	0.29	0.02	0.24	0.10	0.01	0.08	0.00	0.00
4	88	0.00	0.12	0.12	0.00	0.30	0.01	0.28	0.11	0.01	0.00	0.00	0.00
4	89	0.00	0.06	0.01	0.00	0.31	0.01	0.29	0.15	0.01	0.07	0.00	0.00
4	90	0.00	0.00	0.11	0.00	0.32	0.01	0.32	0.19	0.01	0.00	0.00	0.00
4	91	0.00	0.00	0.09	0.00	0.33	0.01	0.33	0.20	0.01	0.00	0.00	0.00
4	92	0.00	0.00	0.01	0.00	0.33	0.01	0.34	0.15	0.01	0.00	0.00	0.00
4	93	0.00	0.00	0.01	0.00	0.34	0.01	0.38	0.15	0.01	0.00	0.00	0.00
4	94	0.00	0.00	0.03	0.00	0.41	0.00	0.40	0.20	0.01	0.07	0.00	0.00
4	95	0.00	0.00	0.08	0.00	0.48	0.00	0.40	0.22	0.00	0.07	0.00	0.00
4	96	0.00	0.00	0.00	0.00	0.53	0.00	0.42	0.21	0.01	0.00	0.00	0.00
4	97	0.00	0.00	0.13	0.00	0.59	0.00	0.42	0.22	0.01	0.07	0.00	0.00

4	98	0.00	0.00	0.09	0.00	0.64	0.00	0.42	0.24	0.01	0.03	0.00	0.00
4	99	0.00	0.00	0.03	0.00	0.68	0.00	0.40	0.27	0.01	0.00	0.00	0.00
4	100	0.00	0.00	0.19	0.00	0.69	0.00	0.38	0.26	0.01	0.07	0.00	0.00
4	101	0.01	0.00	0.22	0.00	0.63	0.00	0.36	0.14	0.01	0.03	0.00	0.00
4	102	0.00	0.00	0.12	0.02	0.56	0.00	0.33	0.05	0.01	0.08	0.00	0.00
4	103	0.00	0.00	0.24	0.06	0.49	0.00	0.30	0.06	0.00	0.03	0.00	0.00
4	104	0.00	0.00	0.08	0.10	0.42	0.00	0.28	0.01	0.01	0.07	0.00	0.00
4	105	0.00	0.02	0.05	0.10	0.34	0.00	0.29	0.04	0.01	0.06	0.00	0.00
4	106	0.00	0.01	0.02	0.10	0.28	0.00	0.29	0.00	0.00	0.03	0.00	0.00
4	107	0.02	0.01	0.06	0.09	0.22	0.00	0.28	0.04	0.01	0.00	0.00	0.00
4	108	0.00	0.02	0.10	0.11	0.21	0.00	0.27	0.00	0.01	0.00	0.00	0.00
4	109	0.00	0.00	0.08	0.11	0.20	0.00	0.28	0.00	0.01	0.08	0.00	0.00
4	110	0.00	0.00	0.09	0.11	0.20	0.00	0.27	0.00	0.01	0.06	0.00	0.00
4	111	0.00	0.00	0.00	0.10	0.19	0.00	0.26	0.04	0.01	0.00	0.00	0.00
4	112	0.00	0.00	0.07	0.11	0.18	0.00	0.25	0.04	0.01	0.03	0.00	0.00
4	113	0.00	0.03	0.02	0.11	0.17	0.00	0.23	0.04	0.00	0.03	0.00	0.00
4	114	0.00	0.00	0.00	0.10	0.16	0.00	0.21	0.04	0.00	0.00	0.00	0.00
4	115	0.00	0.03	0.09	0.12	0.13	0.00	0.22	0.00	0.00	0.08	0.00	0.00
4	116	0.00	0.00	0.10	0.07	0.11	0.00	0.22	0.00	0.01	0.08	0.00	0.00
4	117	0.00	0.00	0.00	0.13	0.08	0.00	0.23	0.00	0.00	0.00	0.00	0.00
4	118	0.02	0.00	0.13	0.13	0.05	0.00	0.23	0.01	0.01	0.08	0.00	0.00
4	119	0.00	0.00	0.16	0.15	0.03	0.00	0.23	0.00	0.01	0.01	0.00	0.00
4	120	0.00	0.00	0.17	0.12	0.02	0.00	0.22	0.00	0.00	0.08	0.00	0.00
4	121	0.00	0.00	0.21	0.13	0.00	0.00	0.21	0.00	0.01	0.02	0.00	0.00
4	122	0.00	0.00	0.23	0.19	0.00	0.00	0.19	0.04	0.01	0.05	0.00	0.00
4	123	0.00	0.00	0.16	0.23	0.00	0.00	0.17	0.04	0.01	0.00	0.00	0.00
4	124	0.04	0.00	0.29	0.27	0.00	0.00	0.14	0.00	0.00	0.03	0.00	0.00
4	125	0.00	0.03	0.35	0.28	0.00	0.00	0.09	0.04	0.01	0.02	0.00	0.00
4	126	0.01	0.00	0.24	0.27	0.00	0.00	0.09	0.04	0.01	0.06	0.00	0.00
4	127	0.00	0.00	0.29	0.27	0.00	0.00	0.07	0.05	0.01	0.04	0.00	0.00
4	128	0.00	0.00	0.17	0.26	0.00	0.00	0.05	0.06	0.01	0.04	0.00	0.00
4	129	0.00	0.02	0.24	0.21	0.00	0.00	0.04	0.01	0.01	0.03	0.00	0.00
4	130	0.00	0.01	0.24	0.17	0.00	0.00	0.03	0.07	0.00	0.01	0.00	0.00
4	131	0.00	0.00	0.10	0.13	0.00	0.00	0.03	0.05	0.01	0.06	0.00	0.00
4	132	0.00	0.00	0.07	0.10	0.00	0.00	0.03	0.05	0.01	0.00	0.00	0.00
4	133	0.00	0.00	0.13	0.00	0.00	0.00	0.02	0.04	0.00	0.00	0.00	0.00

4	134	0.00	0.00	0.12	0.00	0.00	0.01	0.02	0.04	0.00	0.03	0.00	0.00
4	135	0.00	0.00	0.01	0.02	0.00	0.02	0.02	0.03	0.00	0.02	0.00	0.00
4	136	0.00	0.00	0.08	0.03	0.00	0.04	0.02	0.00	0.01	0.03	0.00	0.00
4	137	0.00	0.00	0.00	0.03	0.00	0.06	0.02	0.03	0.01	0.04	0.00	0.00
4	138	0.00	0.00	0.05	0.02	0.00	0.08	0.02	0.00	0.01	0.00	0.00	0.00
4	139	0.00	0.00	0.05	0.05	0.00	0.11	0.02	0.01	0.01	0.00	0.00	0.00
4	140	0.02	0.03	0.08	0.04	0.00	0.13	0.03	0.04	0.00	0.00	0.00	0.00
4	141	0.00	0.02	0.01	0.02	0.00	0.15	0.02	0.04	0.00	0.09	0.00	0.00
4	142	0.00	0.00	0.00	0.01	0.00	0.17	0.02	0.02	0.00	0.00	0.00	0.00
4	143	0.00	0.00	0.00	0.00	0.00	0.20	0.01	0.00	0.01	0.09	0.00	0.00
4	144	0.00	0.00	0.08	0.00	0.00	0.23	0.02	0.00	0.01	0.09	0.00	0.00
4	145	0.00	0.01	0.00	0.00	0.00	0.26	0.02	0.00	0.01	0.08	0.00	0.00
4	146	0.00	0.00	0.00	0.00	0.00	0.30	0.02	0.02	0.01	0.10	0.00	0.00
4	147	0.00	0.00	0.00	0.00	0.00	0.33	0.02	0.00	0.01	0.08	0.00	0.00
4	148	0.00	0.00	0.08	0.00	0.00	0.37	0.02	0.00	0.01	0.12	0.00	0.00
4	149	0.00	0.00	0.00	0.00	0.00	0.39	0.02	0.00	0.01	0.17	0.01	0.00
4	150	0.00	0.01	0.00	0.00	0.00	0.44	0.02	0.01	0.00	0.11	0.02	0.00
4	151	0.00	0.01	0.05	0.00	0.00	0.48	0.02	0.00	0.00	0.22	0.04	0.01
4	152	0.00	0.03	0.08	0.00	0.00	0.50	0.02	0.00	0.00	0.24	0.06	0.02
4	153	0.00	0.02	0.00	0.00	0.00	0.55	0.02	0.00	0.01	0.23	0.06	0.01
4	154	0.02	0.00	0.08	0.00	0.00	0.57	0.02	0.00	0.01	0.21	0.03	0.00
4	155	0.00	0.03	0.07	0.00	0.00	0.61	0.02	0.00	0.01	0.16	0.02	0.00
4	156	0.00	0.02	0.07	0.00	0.00	0.63	0.02	0.01	0.01	0.06	0.01	0.00
4	157	0.00	0.01	0.00	0.00	0.00	0.65	0.02	0.00	0.00	0.16	0.00	0.00
4	158	0.00	0.01	0.00	0.00	0.00	0.78	0.02	0.02	0.01	0.16	0.02	0.00
4	159	0.00	0.03	0.00	0.00	0.00	0.93	0.02	0.00	0.01	0.13	0.00	0.03
4	160	0.00	0.00	0.00	0.00	0.00	1.04	0.02	0.00	0.01	0.22	0.03	0.05
4	161	0.00	0.02	0.00	0.00	0.00	1.15	0.02	0.00	0.01	0.24	0.01	0.06
4	162	0.00	0.02	0.00	0.00	0.00	1.22	0.02	0.04	0.01	0.20	0.00	0.03
4	163	0.00	0.00	0.00	0.00	0.00	1.28	0.02	0.03	0.00	0.10	0.00	0.01
4	164	0.00	0.00	0.07	0.00	0.00	1.28	0.02	0.00	0.00	0.00	0.00	0.01
4	165	0.00	0.00	0.08	0.00	0.00	1.25	0.02	0.00	0.01	0.00	0.00	0.01
4	166	0.00	0.00	0.00	0.00	0.00	1.21	0.02	0.01	0.01	0.00	0.00	0.01
4	167	0.01	0.00	0.04	0.00	0.00	1.18	0.02	0.04	0.01	0.08	0.00	0.00
4	168	0.00	0.00	0.08	0.00	0.00	1.14	0.02	0.04	0.00	0.07	0.00	0.01
4	169	0.00	0.00	0.08	0.00	0.00	1.08	0.02	0.00	0.01	0.04	0.00	0.00

4	170	0.00	0.00	0.07	0.00	0.00	1.05	0.02	0.04	0.01	0.06	0.00	0.00
4	171	0.01	0.00	0.00	0.00	0.00	1.03	0.02	0.04	0.01	0.07	0.00	0.01
4	172	0.00	0.00	0.00	0.00	0.00	0.96	0.02	0.02	0.01	0.00	0.00	0.01
4	173	0.00	0.02	0.00	0.00	0.00	0.89	0.02	0.04	0.01	0.00	0.00	0.01
4	174	0.00	0.01	0.00	0.00	0.00	0.87	0.02	0.00	0.01	0.00	0.00	0.02
4	175	0.00	0.00	0.07	0.00	0.00	0.85	0.02	0.00	0.01	0.00	0.00	0.02
4	176	0.00	0.00	0.00	0.00	0.00	0.83	0.02	0.04	0.00	0.01	0.00	0.04
4	177	0.00	0.00	0.00	0.00	0.00	0.81	0.02	0.00	0.00	0.07	0.00	0.05
4	178	0.00	0.00	0.09	0.00	0.00	0.79	0.02	0.04	0.01	0.05	0.00	0.08
4	179	0.00	0.00	0.08	0.00	0.00	0.75	0.02	0.00	0.01	0.03	0.00	0.10
4	180	0.00	0.02	0.09	0.00	0.00	0.71	0.02	0.00	0.01	0.00	0.00	0.15
4	181	0.00	0.03	0.00	0.00	0.00	0.66	0.01	0.00	0.00	0.07	0.00	0.20
4	182	0.00	0.00	0.00	0.00	0.00	0.62	0.02	0.00	0.01	0.04	0.00	0.22
4	183	0.00	0.02	0.00	0.00	0.01	0.56	0.01	0.00	0.01	0.05	0.00	0.26
4	184	0.00	0.03	0.08	0.00	0.01	0.52	0.02	0.00	0.01	0.05	0.00	0.29
4	185	0.00	0.00	0.09	0.00	0.02	0.45	0.02	0.00	0.01	0.00	0.00	0.32
4	186	0.00	0.00	0.00	0.00	0.03	0.40	0.02	0.04	0.01	0.06	0.00	0.33
4	187	0.00	0.00	0.09	0.00	0.03	0.34	0.01	0.04	0.01	0.06	0.00	0.33
4	188	0.00	0.00	0.06	0.00	0.04	0.27	0.02	0.00	0.01	0.00	0.00	0.33
4	189	0.00	0.00	0.09	0.00	0.05	0.24	0.02	0.00	0.00	0.05	0.00	0.32
4	190	0.00	0.00	0.08	0.00	0.05	0.24	0.02	0.00	0.01	0.00	0.00	0.35
4	191	0.00	0.00	0.00	0.00	0.06	0.23	0.02	0.04	0.00	0.08	0.00	0.41
4	192	0.00	0.02	0.01	0.00	0.06	0.22	0.01	0.04	0.01	0.06	0.00	0.48
4	193	0.00	0.03	0.00	0.00	0.07	0.20	0.02	0.01	0.01	0.07	0.00	0.54
4	194	0.03	0.00	0.00	0.00	0.07	0.21	0.02	0.02	0.01	0.05	0.00	0.59
4	195	0.00	0.00	0.08	0.00	0.08	0.20	0.02	0.01	0.01	0.00	0.00	0.64
4	196	0.00	0.02	0.05	0.00	0.08	0.19	0.02	0.04	0.01	0.00	0.00	0.68
4	197	0.00	0.02	0.00	0.00	0.09	0.18	0.02	0.03	0.01	0.09	0.00	0.72
4	198	0.00	0.01	0.00	0.00	0.09	0.17	0.01	0.04	0.01	0.08	0.00	0.75
4	199	0.00	0.02	0.00	0.00	0.09	0.15	0.02	0.04	0.01	0.00	0.00	0.77
4	200	0.00	0.01	0.00	0.00	0.10	0.14	0.02	0.01	0.01	0.03	0.00	0.79
4	201	0.00	0.00	0.00	0.00	0.10	0.13	0.02	0.00	0.01	0.00	0.00	0.75
4	202	0.00	0.01	0.00	0.00	0.10	0.12	0.01	0.00	0.00	0.02	0.00	0.80
4	203	0.00	0.00	0.00	0.04	0.10	0.12	0.02	0.04	0.01	0.08	0.00	0.76
4	204	0.00	0.01	0.00	0.01	0.00	0.00	0.02	0.04	0.01	0.04	0.00	0.66
4	205	0.00	0.00	0.00	0.02	0.00	0.00	0.02	0.04	0.01	0.05	0.00	0.63

5	1	0.00	0.00	0.00	0.00	0.00	0.00	0.09	0.54	0.87	0.00	0.20	0.05
5	2	0.00	0.00	0.09	0.00	0.00	0.00	0.11	0.61	0.84	0.63	0.21	0.06
5	3	0.00	0.03	0.08	0.00	0.00	0.00	0.12	0.68	0.83	0.65	0.16	0.06
5	4	0.00	0.13	0.07	0.00	0.00	0.00	0.12	0.72	0.79	0.50	0.20	0.07
5	5	0.08	0.14	0.07	0.00	0.00	0.00	0.12	0.77	0.76	0.63	0.15	0.08
5	6	0.01	0.15	0.00	0.00	0.00	0.00	0.13	0.80	0.72	0.68	0.18	0.09
5	7	0.03	0.16	0.07	0.00	0.00	0.00	0.13	0.84	0.66	0.72	0.18	0.09
5	8	0.00	0.06	0.00	0.00	0.00	0.01	0.12	0.86	0.62	0.59	0.12	0.09
5	9	0.07	0.02	0.00	0.00	0.00	0.01	0.12	0.88	0.56	0.73	0.08	0.12
5	10	0.05	0.07	0.00	0.00	0.00	0.01	0.09	0.83	0.51	0.70	0.15	0.15
5	11	0.09	0.00	0.01	0.00	0.00	0.03	0.11	0.82	0.46	0.70	0.17	0.14
5	12	0.03	0.11	0.06	0.00	0.00	0.03	0.11	0.75	0.40	0.66	0.21	0.17
5	13	0.00	0.17	0.11	0.00	0.00	0.03	0.11	0.67	0.37	0.68	0.26	0.20
5	14	0.07	0.00	0.10	0.00	0.00	0.02	0.10	0.64	0.34	0.56	0.31	0.23
5	15	0.08	0.00	0.14	0.00	0.00	0.01	0.11	0.62	0.31	0.69	0.32	0.29
5	16	0.10	0.03	0.37	0.00	0.00	0.01	0.12	0.55	0.29	0.45	0.39	0.35
5	17	0.07	0.25	0.43	0.00	0.00	0.00	0.15	0.47	0.25	0.73	0.43	0.42
5	18	0.00	0.29	0.51	0.00	0.00	0.00	0.19	0.45	0.23	0.78	0.40	0.51
5	19	0.00	0.31	0.56	0.00	0.00	0.00	0.23	0.38	0.20	0.61	0.46	0.60
5	20	0.00	0.30	0.65	0.00	0.00	0.00	0.28	0.41	0.17	0.76	0.49	0.70
5	21	0.28	0.25	0.79	0.00	0.00	0.00	0.34	0.37	0.14	0.86	0.50	0.81
5	22	0.28	0.33	0.90	0.00	0.00	0.00	0.39	0.35	0.11	0.81	0.51	0.91
5	23	0.00	0.32	0.77	0.00	0.00	0.00	0.45	0.32	0.08	0.76	0.46	0.92
5	24	0.06	0.24	0.99	0.00	0.00	0.00	0.52	0.27	0.07	0.62	0.40	0.88
5	25	0.31	0.07	0.89	0.00	0.00	0.00	0.57	0.22	0.07	0.65	0.41	0.85
5	26	0.08	0.30	1.01	0.00	0.00	0.00	0.62	0.22	0.07	0.53	0.48	0.81
5	27	0.17	0.30	1.01	0.00	0.00	0.00	0.67	0.31	0.06	0.45	0.45	0.77
5	28	0.19	0.32	0.97	0.00	0.01	0.00	0.72	0.28	0.06	0.46	0.51	0.72
5	29	0.07	0.33	0.95	0.00	0.01	0.00	0.76	0.32	0.05	0.44	0.52	0.67
5	30	0.23	0.32	0.94	0.00	0.02	0.00	0.81	0.33	0.05	0.36	0.55	0.61
5	31	0.06	0.32	0.91	0.00	0.03	0.00	0.92	0.35	0.04	0.29	0.52	0.54
5	32	0.34	0.33	0.84	0.00	0.04	0.00	1.02	0.31	0.00	0.29	0.55	0.44
5	33	0.32	0.22	0.73	0.00	0.06	0.00	1.13	0.28	0.03	0.21	0.53	0.34
5	34	0.27	0.20	0.69	0.00	0.07	0.00	1.23	0.25	0.02	0.19	0.51	0.26
5	35	0.36	0.28	0.62	0.01	0.08	0.00	1.32	0.20	0.01	0.12	0.39	0.19
5	36	0.36	0.22	0.44	0.00	0.10	0.00	1.42	0.12	0.02	0.10	0.37	0.10

5	37	0.37	0.01	0.53	0.00	0.11	0.00	1.48	0.11	0.02	0.00	0.37	0.07
5	38	0.37	0.00	0.57	0.00	0.13	0.00	1.53	0.00	0.02	0.00	0.38	0.06
5	39	0.34	0.03	0.53	0.00	0.15	0.00	1.56	0.06	0.03	0.00	0.39	0.06
5	40	0.21	0.15	0.74	0.00	0.16	0.00	1.58	0.06	0.02	0.03	0.40	0.05
5	41	0.32	0.00	0.78	0.00	0.19	0.00	1.58	0.07	0.01	0.00	0.49	0.06
5	42	0.29	0.26	0.88	0.00	0.21	0.00	1.59	0.07	0.05	0.12	0.60	0.06
5	43	0.52	0.33	0.62	0.00	0.23	0.00	1.61	0.12	0.06	0.00	0.69	0.05
5	44	0.43	0.40	0.78	0.00	0.25	0.00	1.63	0.06	0.06	0.05	0.76	0.03
5	45	0.56	0.46	0.72	0.00	0.26	0.00	1.60	0.06	0.06	0.10	0.79	0.03
5	46	0.52	0.35	0.50	0.00	0.28	0.00	1.55	0.18	0.08	0.12	0.78	0.02
5	47	0.66	0.56	0.72	0.01	0.33	0.00	1.56	0.14	0.05	0.14	0.85	0.02
5	48	0.55	0.53	0.71	0.03	0.39	0.00	1.57	0.10	0.02	0.10	0.81	0.01
5	49	0.67	0.51	0.73	0.05	0.45	0.00	1.55	0.02	0.01	0.00	0.74	0.00
5	50	0.72	0.51	0.77	0.05	0.51	0.00	1.53	0.00	0.00	0.00	0.61	0.00
5	51	0.53	0.40	0.77	0.09	0.56	0.00	1.48	0.00	0.00	0.11	0.61	0.00
5	52	0.73	0.23	0.79	0.10	0.60	0.00	1.41	0.06	0.01	0.01	0.56	0.00
5	53	0.73	0.38	0.77	0.13	0.62	0.00	1.33	0.00	0.01	0.06	0.48	0.00
5	54	0.71	0.31	0.63	0.15	0.65	0.00	1.25	0.05	0.00	0.10	0.36	0.00
5	55	0.70	0.20	0.57	0.17	0.66	0.00	1.21	0.00	0.01	0.01	0.38	0.00
5	56	0.65	0.25	0.64	0.17	0.66	0.00	1.16	0.00	0.01	0.00	0.31	0.00
5	57	0.56	0.03	0.43	0.17	0.65	0.00	1.11	0.00	0.00	0.00	0.29	0.00
5	58	0.30	0.00	0.07	0.10	0.63	0.00	1.05	0.00	0.01	0.08	0.24	0.00
5	59	0.33	0.05	0.05	0.06	0.60	0.00	0.98	0.03	0.00	0.08	0.23	0.00
5	60	0.16	0.01	0.09	0.07	0.57	0.00	0.91	0.02	0.01	0.00	0.08	0.00
5	61	0.14	0.00	0.00	0.07	0.54	0.00	0.83	0.00	0.01	0.00	0.14	0.00
5	62	0.00	0.00	0.02	0.07	0.50	0.00	0.76	0.02	0.01	0.00	0.10	0.00
5	63	0.00	0.00	0.00	0.07	0.46	0.00	0.68	0.03	0.01	0.01	0.07	0.00
5	64	0.11	0.01	0.08	0.06	0.43	0.00	0.60	0.02	0.01	0.00	0.05	0.00
5	65	0.00	0.00	0.00	0.09	0.43	0.00	0.54	0.02	0.00	0.04	0.00	0.00
5	66	0.00	0.02	0.00	0.13	0.45	0.00	0.52	0.02	0.01	0.02	0.03	0.00
5	67	0.06	0.00	0.00	0.17	0.47	0.00	0.51	0.03	0.01	0.03	0.00	0.00
5	68	0.07	0.00	0.00	0.19	0.48	0.00	0.49	0.04	0.01	0.07	0.01	0.00
5	69	0.12	0.02	0.08	0.23	0.49	0.00	0.48	0.03	0.01	0.02	0.02	0.00
5	70	0.00	0.00	0.08	0.26	0.50	0.00	0.46	0.03	0.01	0.08	0.00	0.00
5	71	0.07	0.01	0.00	0.30	0.50	0.00	0.44	0.07	0.01	0.00	0.01	0.00
5	72	0.00	0.01	0.00	0.31	0.50	0.00	0.42	0.03	0.01	0.08	0.01	0.00

5	73	0.09	0.00	0.00	0.31	0.47	0.00	0.43	0.09	0.01	0.08	0.01	0.00
5	74	0.08	0.00	0.07	0.33	0.44	0.00	0.43	0.07	0.01	0.07	0.01	0.00
5	75	0.09	0.00	0.07	0.33	0.41	0.00	0.43	0.08	0.01	0.00	0.00	0.00
5	76	0.00	0.00	0.08	0.25	0.38	0.00	0.41	0.07	0.01	0.00	0.00	0.00
5	77	0.00	0.00	0.08	0.17	0.34	0.00	0.39	0.07	0.01	0.00	0.00	0.00
5	78	0.02	0.00	0.08	0.06	0.30	0.00	0.36	0.07	0.01	0.00	0.00	0.00
5	79	0.00	0.01	0.09	0.02	0.26	0.00	0.33	0.07	0.01	0.00	0.00	0.00
5	80	0.00	0.00	0.07	0.00	0.21	0.00	0.30	0.09	0.01	0.08	0.00	0.00
5	81	0.00	0.01	0.09	0.00	0.15	0.00	0.26	0.11	0.01	0.00	0.00	0.00
5	82	0.00	0.00	0.08	0.00	0.11	0.00	0.22	0.14	0.01	0.07	0.01	0.00
5	83	0.00	0.00	0.09	0.00	0.07	0.00	0.18	0.15	0.01	0.08	0.00	0.00
5	84	0.00	0.00	0.00	0.00	0.03	0.00	0.14	0.16	0.01	0.08	0.00	0.00
5	85	0.00	0.00	0.08	0.00	0.01	0.00	0.10	0.21	0.00	0.00	0.00	0.00
5	86	0.00	0.00	0.04	0.00	0.00	0.00	0.07	0.22	0.01	0.00	0.00	0.00
5	87	0.00	0.00	0.08	0.00	0.00	0.00	0.05	0.25	0.00	0.00	0.00	0.00
5	88	0.00	0.02	0.09	0.00	0.00	0.00	0.03	0.29	0.00	0.06	0.00	0.00
5	89	0.00	0.00	0.08	0.00	0.00	0.00	0.02	0.33	0.01	0.00	0.00	0.00
5	90	0.01	0.00	0.07	0.00	0.00	0.00	0.02	0.35	0.01	0.02	0.00	0.00
5	91	0.00	0.03	0.09	0.00	0.00	0.00	0.02	0.35	0.00	0.04	0.00	0.00
5	92	0.01	0.03	0.09	0.00	0.00	0.00	0.02	0.40	0.00	0.07	0.00	0.00
5	93	0.00	0.03	0.08	0.00	0.00	0.00	0.01	0.41	0.00	0.06	0.00	0.00
5	94	0.00	0.00	0.07	0.00	0.00	0.00	0.02	0.43	0.01	0.00	0.00	0.00
5	95	0.01	0.02	0.06	0.00	0.00	0.00	0.02	0.42	0.01	0.00	0.00	0.00
5	96	0.00	0.00	0.07	0.00	0.00	0.00	0.02	0.42	0.01	0.01	0.00	0.00
5	97	0.00	0.00	0.08	0.00	0.00	0.00	0.02	0.44	0.00	0.06	0.00	0.00
5	98	0.00	0.01	0.00	0.00	0.00	0.00	0.02	0.45	0.01	0.01	0.00	0.00
5	99	0.00	0.00	0.08	0.00	0.00	0.00	0.02	0.45	0.01	0.00	0.00	0.00
5	100	0.00	0.01	0.09	0.00	0.01	0.00	0.03	0.44	0.01	0.05	0.01	0.00
5	101	0.00	0.00	0.08	0.00	0.03	0.00	0.03	0.47	0.01	0.00	0.00	0.00
5	102	0.00	0.00	0.09	0.00	0.06	0.00	0.04	0.45	0.01	0.00	0.00	0.00
5	103	0.00	0.00	0.08	0.00	0.06	0.00	0.05	0.49	0.01	0.00	0.00	0.00
5	104	0.00	0.00	0.00	0.00	0.06	0.00	0.05	0.50	0.00	0.08	0.00	0.00
5	105	0.00	0.03	0.08	0.00	0.06	0.00	0.05	0.50	0.00	0.05	0.00	0.00
5	106	0.00	0.00	0.07	0.00	0.07	0.00	0.05	0.48	0.00	0.00	0.00	0.00
5	107	0.00	0.00	0.09	0.00	0.07	0.00	0.05	0.68	0.00	0.00	0.06	0.00
5	108	0.00	0.00	0.07	0.00	0.06	0.00	0.05	0.84	0.00	0.00	0.08	0.00

5	109	0.00	0.03	0.09	0.00	0.08	0.00	0.05	0.89	0.01	0.00	0.09	0.00
5	110	0.00	0.00	0.07	0.00	0.11	0.01	0.05	0.89	0.01	0.10	0.09	0.00
5	111	0.00	0.01	0.05	0.00	0.13	0.04	0.05	0.66	0.01	0.10	0.01	0.00
5	112	0.00	0.00	0.08	0.00	0.16	0.07	0.06	0.39	0.01	0.11	0.03	0.00
5	113	0.00	0.00	0.00	0.00	0.18	0.11	0.07	0.30	0.00	0.08	0.00	0.00
5	114	0.00	0.03	0.08	0.00	0.18	0.14	0.08	0.13	0.01	0.11	0.00	0.00
5	115	0.00	0.00	0.09	0.00	0.19	0.19	0.08	0.11	0.01	0.09	0.00	0.00
5	116	0.00	0.00	0.09	0.00	0.19	0.24	0.09	0.07	0.01	0.06	0.00	0.00
5	117	0.00	0.00	0.06	0.00	0.19	0.28	0.09	0.08	0.01	0.08	0.00	0.00
5	118	0.02	0.00	0.08	0.00	0.19	0.32	0.08	0.07	0.01	0.00	0.00	0.00
5	119	0.00	0.02	0.08	0.00	0.21	0.32	0.07	0.13	0.01	0.05	0.00	0.00
5	120	0.00	0.00	0.09	0.00	0.22	0.32	0.05	0.13	0.00	0.06	0.00	0.00
5	121	0.00	0.00	0.08	0.00	0.26	0.32	0.04	0.12	0.01	0.08	0.00	0.00
5	122	0.00	0.00	0.08	0.00	0.29	0.32	0.03	0.09	0.01	0.04	0.00	0.00
5	123	0.00	0.00	0.08	0.00	0.31	0.31	0.03	0.07	0.01	0.06	0.00	0.00
5	124	0.00	0.00	0.07	0.00	0.34	0.30	0.02	0.14	0.01	0.00	0.00	0.00
5	125	0.00	0.01	0.09	0.00	0.35	0.29	0.02	0.12	0.01	0.06	0.00	0.00
5	126	0.00	0.00	0.09	0.00	0.35	0.25	0.02	0.16	0.00	0.06	0.00	0.00
5	127	0.00	0.02	0.09	0.00	0.35	0.22	0.02	0.22	0.01	0.07	0.00	0.00
5	128	0.00	0.02	0.07	0.00	0.34	0.19	0.01	0.22	0.01	0.11	0.00	0.00
5	129	0.00	0.00	0.08	0.00	0.34	0.15	0.02	0.23	0.01	0.14	0.00	0.00
5	130	0.00	0.00	0.09	0.00	0.31	0.12	0.03	0.27	0.00	0.16	0.00	0.00
5	131	0.02	0.00	0.07	0.00	0.30	0.09	0.03	0.30	0.01	0.26	0.00	0.00
5	132	0.00	0.02	0.07	0.00	0.28	0.07	0.04	0.38	0.01	0.11	0.00	0.00
5	133	0.00	0.00	0.09	0.00	0.25	0.05	0.04	0.38	0.01	0.38	0.00	0.00
5	134	0.02	0.00	0.09	0.00	0.22	0.03	0.05	0.38	0.01	0.43	0.00	0.00
5	135	0.00	0.00	0.08	0.00	0.20	0.02	0.06	0.38	0.01	0.35	0.03	0.00
5	136	0.01	0.00	0.07	0.00	0.17	0.01	0.06	0.36	0.01	0.45	0.02	0.00
5	137	0.02	0.01	0.09	0.00	0.16	0.01	0.06	0.32	0.00	0.47	0.02	0.00
5	138	0.00	0.00	0.00	0.00	0.18	0.01	0.08	0.36	0.01	0.46	0.04	0.00
5	139	0.00	0.00	0.00	0.00	0.19	0.00	0.07	0.32	0.04	0.46	0.06	0.00
5	140	0.00	0.00	0.07	0.00	0.20	0.01	0.07	0.28	0.09	0.48	0.01	0.00
5	141	0.00	0.00	0.06	0.00	0.21	0.01	0.06	0.21	0.17	0.55	0.10	0.00
5	142	0.00	0.00	0.09	0.00	0.22	0.02	0.05	0.22	0.26	0.64	0.19	0.00
5	143	0.00	0.00	0.07	0.00	0.23	0.02	0.05	0.24	0.36	0.69	0.25	0.00
5	144	0.00	0.13	0.08	0.00	0.23	0.02	0.04	0.18	0.46	0.93	0.25	0.00

5	145	0.00	0.18	0.07	0.00	0.23	0.01	0.03	0.22	0.56	0.95	0.40	0.00
5	146	0.07	0.17	0.09	0.00	0.24	0.02	0.03	0.20	0.64	1.25	0.45	0.00
5	147	0.20	0.20	0.09	0.00	0.24	0.02	0.02	0.29	0.64	1.13	0.40	0.00
5	148	0.18	0.15	0.08	0.00	0.19	0.03	0.03	0.27	0.61	1.05	0.41	0.00
5	149	0.03	0.17	0.08	0.00	0.16	0.04	0.03	0.28	0.59	1.12	0.40	0.00
5	150	0.07	0.11	0.09	0.00	0.12	0.06	0.03	0.34	0.60	1.17	0.40	0.00
5	151	0.06	0.15	0.01	0.00	0.08	0.07	0.04	0.26	0.60	1.06	0.39	0.00
5	152	0.07	0.16	0.08	0.00	0.06	0.08	0.05	0.28	0.61	0.83	0.35	0.00
5	153	0.01	0.10	0.02	0.00	0.03	0.09	0.06	0.28	0.58	0.73	0.34	0.00
5	154	0.01	0.15	0.07	0.00	0.01	0.10	0.07	0.28	0.59	0.67	0.33	0.00
5	155	0.00	0.14	0.09	0.00	0.00	0.10	0.09	0.28	0.57	0.64	0.29	0.00
5	156	0.00	0.00	0.06	0.00	0.00	0.10	0.10	0.27	0.55	0.50	0.23	0.00
5	157	0.00	0.06	0.09	0.00	0.00	0.10	0.11	0.27	0.51	0.38	0.22	0.00
5	158	0.00	0.09	0.08	0.00	0.00	0.09	0.13	0.26	0.47	0.42	0.18	0.00
5	159	0.00	0.11	0.00	0.00	0.00	0.10	0.14	0.29	0.43	0.28	0.16	0.00
5	160	0.00	0.10	0.06	0.00	0.00	0.11	0.15	0.33	0.39	0.33	0.14	0.00
5	161	0.00	0.07	0.07	0.00	0.00	0.11	0.15	0.29	0.35	0.30	0.15	0.00
5	162	0.00	0.11	0.08	0.00	0.00	0.12	0.15	0.36	0.30	0.33	0.15	0.00
5	163	0.00	0.05	0.06	0.00	0.00	0.12	0.14	0.32	0.25	0.20	0.12	0.00
5	164	0.01	0.11	0.09	0.00	0.00	0.12	0.14	0.32	0.20	0.33	0.10	0.00
5	165	0.00	0.09	0.08	0.00	0.00	0.12	0.14	0.39	0.17	0.15	0.09	0.00
5	166	0.00	0.07	0.09	0.00	0.00	0.12	0.13	0.39	0.14	0.29	0.00	0.00
5	167	0.03	0.05	0.08	0.00	0.00	0.16	0.12	0.37	0.11	0.18	0.07	0.00
5	168	0.00	0.00	0.09	0.00	0.00	0.22	0.11	0.51	0.09	0.30	0.06	0.00
5	169	0.01	0.03	0.07	0.00	0.00	0.28	0.10	0.47	0.07	0.38	0.05	0.02
5	170	0.00	0.00	0.08	0.00	0.00	0.34	0.09	0.52	0.04	0.26	0.05	0.03
5	171	0.06	0.00	0.07	0.00	0.00	0.42	0.08	0.51	0.00	0.36	0.03	0.04
5	172	0.00	0.00	0.07	0.00	0.00	0.50	0.07	0.59	0.01	0.44	0.04	0.05
5	173	0.00	0.00	0.09	0.00	0.00	0.57	0.06	0.61	0.00	0.46	0.02	0.05
5	174	0.00	0.03	0.08	0.00	0.00	0.63	0.05	0.55	0.00	0.32	0.01	0.07
5	175	0.01	0.03	0.08	0.00	0.00	0.72	0.05	0.55	0.00	0.32	0.00	0.08
5	176	0.06	0.02	0.07	0.00	0.00	0.78	0.05	0.49	0.01	0.40	0.01	0.09
5	177	0.00	0.02	0.02	0.00	0.00	0.84	0.04	0.43	0.01	0.34	0.02	0.09
5	178	0.00	0.00	0.06	0.00	0.01	0.90	0.04	0.41	0.00	0.39	0.04	0.09
5	179	0.04	0.00	0.07	0.00	0.01	0.96	0.04	0.34	0.00	0.39	0.06	0.08
5	180	0.10	0.03	0.08	0.00	0.01	1.04	0.05	0.28	0.05	0.45	0.08	0.07

5	181	0.06	0.00	0.09	0.00	0.01	1.11	0.07	0.24	0.10	0.55	0.10	0.07
5	182	0.12	0.02	0.09	0.00	0.01	1.19	0.08	0.22	0.17	0.56	0.12	0.06
5	183	0.12	0.00	0.04	0.00	0.02	1.26	0.09	0.23	0.25	0.52	0.14	0.06
5	184	0.05	0.00	0.07	0.00	0.01	1.35	0.11	0.12	0.36	0.67	0.15	0.04
5	185	0.00	0.02	0.09	0.00	0.02	1.42	0.13	0.07	0.38	0.67	0.19	0.05
5	186	0.00	0.00	0.08	0.00	0.02	1.50	0.15	0.14	0.40	0.65	0.19	0.05
5	187	0.00	0.01	0.07	0.00	0.03	1.57	0.17	0.13	0.42	0.52	0.19	0.04
5	188	0.07	0.00	0.05	0.00	0.03	1.63	0.20	0.08	0.43	0.60	0.19	0.04
5	189	0.02	0.04	0.08	0.00	0.03	1.69	0.23	0.06	0.46	0.53	0.12	0.04
5	190	0.09	0.05	0.00	0.00	0.04	1.73	0.26	0.16	0.47	0.45	0.20	0.03
5	191	0.00	0.06	0.08	0.00	0.03	1.74	0.28	0.05	0.50	0.54	0.17	0.00
5	192	0.04	0.00	0.08	0.00	0.04	1.77	0.29	0.07	0.52	0.43	0.18	0.02
5	193	0.00	0.10	0.08	0.00	0.05	1.76	0.30	0.06	0.53	0.40	0.17	0.02
5	194	0.09	0.10	0.01	0.00	0.05	1.75	0.30	0.03	0.55	0.50	0.17	0.01
5	195	0.00	0.13	0.08	0.00	0.05	1.71	0.28	0.06	0.56	0.50	0.13	0.00
5	196	0.00	0.07	0.03	0.00	0.06	1.69	0.26	0.00	0.57	0.48	0.16	0.00
5	197	0.01	0.16	0.09	0.00	0.06	1.65	0.24	0.00	0.58	0.40	0.15	0.00
5	198	0.02	0.00	0.07	0.00	0.06	1.60	0.20	0.00	0.58	0.41	0.12	0.00
5	199	0.01	0.05	0.07	0.00	0.06	1.55	0.16	0.00	0.59	0.27	0.12	0.00
5	200	0.00	0.17	0.00	0.00	0.06	1.50	0.12	0.03	0.58	0.26	0.16	0.00
5	201	0.07	0.17	0.02	0.00	0.06	1.44	0.08	0.04	0.58	0.29	0.14	0.00
5	202	0.02	0.18	0.04	0.00	0.06	1.39	0.06	0.00	0.57	0.24	0.14	0.00
5	203	0.00	0.11	0.00	0.00	0.06	1.34	0.03	0.01	0.57	0.39	0.17	0.00
5	204	0.00	0.15	0.05	0.00	0.06	1.30	0.03	0.00	0.56	0.42	0.14	0.00
5	205	0.00	0.00	0.06	0.00	0.06	1.26	0.02	0.00	0.56	0.34	0.21	0.00
5	206	0.02	0.00	0.08	0.00	0.00	0.00	0.02	0.00	0.55	0.28	0.16	0.00
5	207	0.04	0.18	0.00	0.00	0.00	0.00	0.00	0.00	0.00	0.26	0.00	0.00
5	208	0.00	0.18	0.07	0.00	0.00	0.00	0.00	0.00	0.00	0.35	0.00	0.00
5	209	0.04	0.18	0.00	0.00	0.00	0.00	0.00	0.00	0.00	0.25	0.00	0.00
5	210	0.00	0.00	0.08	0.00	0.00	0.00	0.00	0.00	0.00	0.18	0.00	0.00
5	211	0.01	0.00	0.04	0.00	0.00	0.00	0.00	0.00	0.00	0.35	0.00	0.00
5	212	0.00	0.17	0.00	0.00	0.00	0.00	0.00	0.00	0.00	0.28	0.00	0.00
6	1	0.15	0.00	1.09	0.00	0.00	0.00	0.87	0.00	0.15	0.00	0.00	0.02
6	2	0.10	0.00	1.09	0.00	0.00	0.00	0.90	0.00	0.16	0.00	0.00	0.01
6	3	0.00	0.00	1.09	0.00	0.00	0.00	0.91	0.00	0.16	0.04	0.00	0.00
6	4	0.07	0.00	1.04	0.00	0.00	0.00	0.92	0.00	0.16	0.04	0.00	0.00

6	5	0.07	0.00	1.03	0.00	0.00	0.00	0.92	0.07	0.18	0.00	0.00	0.00
6	6	0.07	0.00	1.02	0.00	0.00	0.00	0.92	0.08	0.14	0.07	0.00	0.00
6	7	0.05	0.00	0.90	0.00	0.00	0.00	0.91	0.01	0.21	0.00	0.00	0.00
6	8	0.00	0.02	0.97	0.00	0.00	0.00	0.90	0.00	0.22	0.03	0.00	0.00
6	9	0.00	0.00	0.95	0.00	0.00	0.00	0.89	0.00	0.24	0.00	0.00	0.00
6	10	0.00	0.00	0.88	0.00	0.00	0.00	0.88	0.04	0.25	0.00	0.00	0.00
6	11	0.04	0.01	0.83	0.00	0.00	0.00	0.87	0.03	0.25	0.03	0.00	0.00
6	12	0.00	0.00	0.73	0.00	0.00	0.00	0.84	0.08	0.27	0.00	0.00	0.00
6	13	0.00	0.00	0.63	0.00	0.00	0.00	0.84	0.09	0.28	0.03	0.00	0.00
6	14	0.00	0.00	0.54	0.00	0.00	0.00	0.81	0.09	0.28	0.03	0.00	0.00
6	15	0.00	0.00	0.46	0.00	0.00	0.01	0.80	0.12	0.28	0.08	0.00	0.00
6	16	0.08	0.00	0.99	0.00	0.00	0.01	0.77	0.24	0.23	0.01	0.00	0.00
6	17	0.10	0.00	0.87	0.00	0.00	0.02	0.77	0.29	0.18	0.06	0.00	0.00
6	18	0.01	0.02	0.86	0.00	0.00	0.03	0.76	0.38	0.16	0.00	0.00	0.00
6	19	0.04	0.01	0.79	0.00	0.00	0.04	0.73	0.41	0.13	0.07	0.00	0.00
6	20	0.00	0.00	0.82	0.00	0.00	0.04	0.70	0.37	0.10	0.03	0.00	0.00
6	21	0.00	0.00	0.74	0.00	0.00	0.07	0.68	0.51	0.07	0.00	0.00	0.00
6	22	0.04	0.00	0.66	0.00	0.00	0.11	0.64	0.65	0.05	0.00	0.00	0.00
6	23	0.04	0.00	0.68	0.00	0.00	0.15	0.61	0.82	0.04	0.02	0.00	0.00
6	24	0.00	0.00	0.67	0.00	0.00	0.19	0.57	0.85	0.02	0.01	0.00	0.00
6	25	0.03	0.00	0.68	0.00	0.00	0.24	0.53	0.76	0.01	0.00	0.00	0.00
6	26	0.01	0.00	0.68	0.00	0.00	0.29	0.49	0.68	0.00	0.06	0.00	0.00
6	27	0.00	0.00	0.61	0.00	0.00	0.37	0.45	0.50	0.01	0.08	0.00	0.00
6	28	0.00	0.00	0.69	0.00	0.00	0.45	0.40	0.49	0.00	0.00	0.00	0.00
6	29	0.00	0.00	0.68	0.00	0.00	0.53	0.37	0.42	0.00	0.05	0.00	0.00
6	30	0.00	0.00	0.68	0.00	0.00	0.62	0.33	0.29	0.01	0.01	0.00	0.00
6	31	0.00	0.00	0.57	0.00	0.00	0.69	0.32	0.15	0.01	0.08	0.00	0.00
6	32	0.01	0.00	0.39	0.00	0.00	0.79	0.32	0.10	0.01	0.06	0.00	0.00
6	33	0.02	0.00	0.40	0.00	0.00	0.86	0.31	0.05	0.01	0.06	0.00	0.00
6	34	0.00	0.00	0.42	0.00	0.00	0.93	0.30	0.04	0.00	0.06	0.00	0.00
6	35	0.00	0.02	0.32	0.00	0.00	0.98	0.29	0.04	0.01	0.05	0.00	0.00
6	36	0.00	0.00	0.30	0.00	0.00	1.00	0.28	0.00	0.00	0.01	0.00	0.00
6	37	0.00	0.00	0.42	0.00	0.00	1.02	0.26	0.00	0.01	0.02	0.00	0.00
6	38	0.00	0.02	0.41	0.00	0.00	1.02	0.25	0.03	0.01	0.06	0.00	0.00
6	39	0.00	0.03	0.42	0.00	0.00	0.99	0.24	0.04	0.00	0.07	0.00	0.00
6	40	0.00	0.03	0.39	0.00	0.00	0.99	0.22	0.01	0.00	0.08	0.00	0.00

6	41	0.00	0.00	0.39	0.00	0.00	0.97	0.21	0.00	0.01	0.07	0.00	0.00
6	42	0.00	0.00	0.36	0.00	0.00	0.93	0.19	0.04	0.01	0.06	0.00	0.00
6	43	0.00	0.00	0.34	0.00	0.00	0.91	0.14	0.02	0.01	0.05	0.00	0.00
6	44	0.00	0.00	0.16	0.00	0.00	0.92	0.10	0.00	0.00	0.05	0.00	0.00
6	45	0.01	0.00	0.28	0.00	0.00	0.91	0.07	0.01	0.01	0.08	0.00	0.00
6	46	0.00	0.00	0.27	0.00	0.00	0.90	0.05	0.04	0.01	0.02	0.00	0.00
6	47	0.00	0.03	0.25	0.00	0.00	0.87	0.03	0.03	0.01	0.00	0.00	0.00
6	48	0.01	0.00	0.17	0.00	0.00	0.84	0.02	0.02	0.01	0.01	0.00	0.00
6	49	0.00	0.00	0.16	0.00	0.00	0.84	0.02	0.00	0.01	0.08	0.00	0.00
6	50	0.00	0.00	0.18	0.00	0.00	0.82	0.02	0.00	0.01	0.00	0.00	0.00
6	51	0.00	0.00	0.26	0.00	0.00	0.83	0.03	0.04	0.00	0.04	0.00	0.00
6	52	0.00	0.00	0.14	0.00	0.00	0.81	0.03	0.00	0.01	0.00	0.00	0.00
6	53	0.00	0.00	0.23	0.00	0.00	0.82	0.03	0.03	0.01	0.08	0.00	0.00
6	54	0.00	0.00	0.20	0.00	0.00	0.80	0.03	0.04	0.01	0.08	0.00	0.00
6	55	0.01	0.00	0.19	0.00	0.00	0.79	0.04	0.03	0.01	0.00	0.00	0.00
6	56	0.00	0.00	0.15	0.00	0.00	0.77	0.04	0.00	0.01	0.00	0.00	0.00
6	57	0.05	0.00	0.10	0.00	0.00	0.75	0.05	0.00	0.01	0.00	0.00	0.00
6	58	0.00	0.00	0.09	0.01	0.00	0.73	0.06	0.00	0.01	0.00	0.00	0.00
6	59	0.08	0.10	0.08	0.00	0.00	0.75	0.08	0.00	0.01	0.01	0.00	0.00
6	60	0.05	0.08	0.00	0.01	0.00	0.78	0.09	0.04	0.01	0.07	0.00	0.00
6	61	0.00	0.12	0.10	0.00	0.00	0.81	0.11	0.00	0.01	0.06	0.01	0.00
6	62	0.00	0.00	0.10	0.00	0.00	0.85	0.13	0.03	0.00	0.00	0.00	0.00
6	63	0.03	0.14	0.00	0.00	0.00	0.89	0.14	0.04	0.00	0.07	0.01	0.00
6	64	0.08	0.14	0.01	0.00	0.00	0.91	0.17	0.00	0.00	0.01	0.00	0.00
6	65	0.11	0.15	0.10	0.01	0.00	0.93	0.21	0.00	0.00	0.04	0.00	0.00
6	66	0.13	0.07	0.10	0.00	0.00	0.97	0.26	0.00	0.01	0.07	0.00	0.00
6	67	0.13	0.15	0.07	0.00	0.00	0.98	0.32	0.00	0.01	0.05	0.01	0.00
6	68	0.05	0.00	0.11	0.00	0.00	1.00	0.37	0.03	0.01	0.05	0.00	0.00
6	69	0.00	0.12	0.12	0.00	0.00	1.01	0.43	0.00	0.01	0.07	0.00	0.00
6	70	0.00	0.11	0.12	0.00	0.00	1.01	0.47	0.00	0.00	0.08	0.00	0.00
6	71	0.03	0.00	0.00	0.01	0.00	0.98	0.54	0.00	0.00	0.05	0.00	0.00
6	72	0.06	0.00	0.01	0.00	0.00	0.98	0.59	0.00	0.00	0.00	0.00	0.00
6	73	0.07	0.00	0.00	0.00	0.00	0.97	0.64	0.01	0.00	0.00	0.00	0.00
6	74	0.22	0.00	0.00	0.02	0.01	0.91	0.68	0.00	0.00	0.07	0.00	0.00
6	75	0.19	0.00	0.00	0.02	0.01	0.91	0.71	0.00	0.00	0.03	0.00	0.00
6	76	0.16	0.03	0.00	0.03	0.01	0.88	0.77	0.00	0.01	0.00	0.00	0.00

6	77	0.25	0.03	0.00	0.03	0.00	0.85	0.81	0.00	0.02	0.07	0.00	0.00
6	78	0.25	0.00	0.17	0.01	0.00	0.82	0.84	0.00	0.03	0.00	0.00	0.00
6	79	0.31	0.08	0.00	0.04	0.01	0.78	0.87	0.04	0.04	0.07	0.00	0.00
6	80	0.27	0.08	0.16	0.03	0.01	0.76	0.88	0.00	0.03	0.05	0.00	0.00
6	81	0.31	0.12	0.10	0.03	0.00	0.75	0.88	0.00	0.03	0.00	0.00	0.00
6	82	0.28	0.08	0.12	0.04	0.00	0.72	0.91	0.04	0.03	0.00	0.00	0.00
6	83	0.24	0.00	0.00	0.03	0.00	0.71	0.92	0.00	0.02	0.00	0.00	0.00
6	84	0.24	0.00	0.00	0.03	0.00	0.68	0.92	0.00	0.02	0.07	0.00	0.00
6	85	0.34	0.03	0.09	0.02	0.00	0.64	0.91	0.00	0.02	0.07	0.00	0.00
6	86	0.25	0.00	0.10	0.03	0.00	0.60	0.89	0.00	0.02	0.02	0.00	0.00
6	87	0.31	0.00	0.01	0.00	0.00	0.56	0.85	0.03	0.02	0.00	0.00	0.00
6	88	0.20	0.00	0.06	0.02	0.00	0.51	0.80	0.00	0.01	0.00	0.00	0.00
6	89	0.18	0.00	0.00	0.02	0.00	0.45	0.73	0.00	0.00	0.07	0.00	0.00
6	90	0.23	0.00	0.09	0.00	0.00	0.42	0.68	0.00	0.01	0.00	0.00	0.00
6	91	0.16	0.03	0.03	0.00	0.00	0.37	0.61	0.00	0.01	0.00	0.00	0.00
6	92	0.00	0.00	0.08	0.00	0.00	0.30	0.53	0.04	0.02	0.07	0.00	0.00
6	93	0.03	0.00	0.09	0.00	0.00	0.25	0.46	0.00	0.03	0.00	0.00	0.00
6	94	0.00	0.00	0.00	0.00	0.00	0.20	0.39	0.00	0.04	0.05	0.00	0.00
6	95	0.00	0.00	0.05	0.00	0.00	0.16	0.31	0.00	0.06	0.00	0.00	0.00
6	96	0.00	0.03	0.09	0.00	0.00	0.13	0.26	0.00	0.09	0.00	0.00	0.00
6	97	0.00	0.01	0.09	0.00	0.00	0.10	0.21	0.04	0.10	0.00	0.00	0.00
6	98	0.00	0.02	0.00	0.00	0.00	0.10	0.16	0.04	0.12	0.08	0.00	0.00
6	99	0.00	0.00	0.08	0.00	0.00	0.10	0.12	0.00	0.13	0.00	0.00	0.00
6	100	0.00	0.00	0.08	0.00	0.00	0.11	0.09	0.04	0.15	0.05	0.00	0.00
6	101	0.01	0.00	0.09	0.00	0.00	0.12	0.08	0.04	0.16	0.04	0.00	0.00
6	102	0.00	0.00	0.06	0.00	0.00	0.13	0.10	0.04	0.17	0.02	0.00	0.00
6	103	0.03	0.00	0.09	0.00	0.00	0.13	0.12	0.00	0.19	0.07	0.00	0.00
6	104	0.00	0.00	0.08	0.00	0.00	0.14	0.14	0.00	0.19	0.01	0.00	0.00
6	105	0.00	0.00	0.08	0.00	0.00	0.14	0.15	0.00	0.20	0.00	0.00	0.00
6	106	0.00	0.01	0.00	0.00	0.00	0.14	0.16	0.00	0.21	0.05	0.00	0.00
6	107	0.00	0.00	0.08	0.00	0.00	0.15	0.18	0.00	0.21	0.07	0.01	0.00
6	108	0.01	0.00	0.07	0.00	0.00	0.15	0.24	0.00	0.22	0.00	0.02	0.00
6	109	0.00	0.00	0.07	0.00	0.00	0.14	0.31	0.04	0.22	0.10	0.02	0.00
6	110	0.00	0.00	0.00	0.00	0.01	0.13	0.39	0.00	0.22	0.03	0.04	0.00
6	111	0.00	0.00	0.09	0.00	0.01	0.13	0.47	0.00	0.22	0.10	0.03	0.00
6	112	0.00	0.05	0.11	0.00	0.01	0.13	0.53	0.00	0.19	0.12	0.09	0.00

6	113	0.00	0.04	0.00	0.00	0.01	0.12	0.63	0.01	0.17	0.11	0.08	0.00
6	114	0.00	0.00	0.00	0.00	0.01	0.11	0.69	0.01	0.15	0.10	0.07	0.00
6	115	0.00	0.00	0.05	0.00	0.01	0.10	0.75	0.01	0.15	0.10	0.07	0.00
6	116	0.01	0.00	0.14	0.00	0.01	0.09	0.79	0.01	0.13	0.08	0.07	0.00
6	117	0.02	0.00	0.05	0.00	0.01	0.08	0.82	0.01	0.11	0.00	0.07	0.00
6	118	0.08	0.00	0.24	0.00	0.01	0.07	0.80	0.02	0.09	0.08	0.07	0.00
6	119	0.05	0.00	0.08	0.00	0.01	0.06	0.79	0.00	0.08	0.04	0.09	0.00
6	120	0.00	0.00	0.11	0.00	0.00	0.05	0.77	0.03	0.06	0.07	0.10	0.00
6	121	0.08	0.02	0.22	0.00	0.01	0.03	0.74	0.00	0.05	0.09	0.09	0.00
6	122	0.08	0.02	0.23	0.00	0.00	0.02	0.69	0.00	0.03	0.05	0.04	0.00
6	123	0.00	0.00	0.24	0.00	0.00	0.01	0.64	0.04	0.03	0.06	0.06	0.00
6	124	0.00	0.00	0.23	0.01	0.00	0.00	0.58	0.04	0.02	0.08	0.00	0.00
6	125	0.04	0.00	0.22	0.01	0.00	0.00	0.52	0.04	0.01	0.00	0.03	0.00
6	126	0.01	0.00	0.04	0.03	0.00	0.00	0.45	0.02	0.01	0.10	0.01	0.00
6	127	0.00	0.00	0.06	0.04	0.00	0.00	0.38	0.00	0.01	0.05	0.02	0.00
6	128	0.00	0.00	0.18	0.03	0.00	0.00	0.33	0.04	0.01	0.09	0.02	0.00
6	129	0.00	0.01	0.18	0.02	0.00	0.00	0.27	0.04	0.00	0.06	0.01	0.00
6	130	0.01	0.00	0.15	0.05	0.00	0.00	0.22	0.00	0.01	0.00	0.00	0.00
6	131	0.00	0.00	0.03	0.03	0.00	0.00	0.21	0.00	0.01	0.06	0.00	0.00
6	132	0.00	0.05	0.18	0.09	0.00	0.00	0.22	0.00	0.00	0.00	0.00	0.00
6	133	0.00	0.10	0.04	0.16	0.00	0.00	0.22	0.03	0.01	0.00	0.03	0.00
6	134	0.02	0.06	0.24	0.19	0.00	0.00	0.24	0.04	0.00	0.08	0.00	0.00
6	135	0.03	0.00	0.26	0.23	0.00	0.00	0.24	0.04	0.00	0.01	0.01	0.00
6	136	0.04	0.00	0.35	0.27	0.00	0.00	0.25	0.00	0.01	0.05	0.00	0.00
6	137	0.09	0.00	0.39	0.30	0.00	0.00	0.26	0.00	0.01	0.06	0.01	0.00
6	138	0.07	0.00	0.50	0.31	0.00	0.00	0.27	0.04	0.01	0.02	0.01	0.00
6	139	0.12	0.00	0.53	0.34	0.00	0.00	0.26	0.00	0.01	0.01	0.00	0.00
6	140	0.00	0.00	0.48	0.37	0.00	0.00	0.20	0.04	0.00	0.04	0.00	0.00
6	141	0.11	0.00	0.34	0.36	0.00	0.00	0.13	0.02	0.01	0.08	0.00	0.00
6	142	0.00	0.00	0.46	0.42	0.00	0.00	0.08	0.04	0.01	0.02	0.03	0.00
6	143	0.10	0.04	0.42	0.41	0.00	0.00	0.05	0.04	0.01	0.00	0.01	0.00
6	144	0.09	0.00	0.35	0.46	0.00	0.00	0.03	0.00	0.01	0.06	0.03	0.00
6	145	0.03	0.08	0.20	0.46	0.00	0.00	0.02	0.03	0.00	0.00	0.06	0.00
6	146	0.01	0.06	0.27	0.49	0.00	0.00	0.02	0.03	0.00	0.00	0.06	0.00
6	147	0.08	0.07	0.24	0.51	0.00	0.00	0.01	0.00	0.00	0.01	0.07	0.00
6	148	0.02	0.00	0.15	0.63	0.00	0.00	0.02	0.00	0.01	0.08	0.06	0.00

6	149	0.03	0.08	0.17	0.73	0.00	0.00	0.02	0.03	0.00	0.00	0.07	0.00
6	150	0.00	0.08	0.20	0.64	0.00	0.00	0.02	0.04	0.00	0.00	0.07	0.00
6	151	0.05	0.04	0.22	0.48	0.00	0.00	0.02	0.04	0.00	0.00	0.03	0.00
6	152	0.00	0.00	0.13	0.33	0.00	0.00	0.02	0.02	0.01	0.01	0.03	0.00
6	153	0.00	0.00	0.22	0.21	0.00	0.00	0.02	0.00	0.01	0.00	0.00	0.00
6	154	0.00	0.08	0.26	0.24	0.00	0.00	0.01	0.01	0.01	0.00	0.00	0.00
6	155	0.02	0.00	0.26	0.29	0.00	0.00	0.02	0.04	0.01	0.05	0.00	0.00
6	156	0.00	0.00	0.25	0.31	0.00	0.00	0.02	0.02	0.02	0.07	0.00	0.00
6	157	0.00	0.00	0.29	0.36	0.00	0.00	0.02	0.04	0.01	0.00	0.00	0.00
6	158	0.00	0.04	0.51	0.39	0.00	0.00	0.02	0.04	0.02	0.07	0.00	0.01
6	159	0.02	0.00	0.56	0.41	0.00	0.00	0.02	0.00	0.03	0.08	0.00	0.02
6	160	0.00	0.04	0.51	0.42	0.00	0.00	0.02	0.00	0.03	0.07	0.00	0.03
6	161	0.00	0.01	0.66	0.36	0.00	0.00	0.02	0.00	0.02	0.08	0.00	0.04
6	162	0.04	0.00	0.65	0.46	0.00	0.00	0.02	0.04	0.04	0.00	0.00	0.04
6	163	0.00	0.06	0.65	0.52	0.00	0.00	0.02	0.04	0.05	0.00	0.00	0.04
6	164	0.04	0.03	0.63	0.60	0.00	0.00	0.02	0.00	0.05	0.00	0.00	0.02
6	165	0.02	0.06	0.49	0.69	0.00	0.00	0.02	0.00	0.06	0.00	0.00	0.03
6	166	0.05	0.00	0.60	0.74	0.00	0.00	0.02	0.00	0.06	0.04	0.00	0.02
6	167	0.01	0.05	0.61	0.82	0.00	0.00	0.02	0.00	0.08	0.00	0.00	0.01
6	168	0.09	0.03	0.49	0.88	0.00	0.00	0.01	0.00	0.12	0.00	0.00	0.01
6	169	0.11	0.05	0.51	0.91	0.00	0.00	0.01	0.00	0.16	0.05	0.00	0.01
6	170	0.09	0.00	0.50	0.92	0.00	0.00	0.02	0.00	0.19	0.08	0.00	0.01
6	171	0.12	0.00	0.47	0.93	0.00	0.00	0.02	0.00	0.20	0.07	0.00	0.01
6	172	0.05	0.00	0.48	0.89	0.00	0.00	0.02	0.00	0.17	0.05	0.00	0.02
6	173	0.07	0.00	0.53	0.85	0.00	0.00	0.02	0.00	0.13	0.03	0.00	0.02
6	174	0.07	0.00	0.52	0.75	0.00	0.00	0.02	0.00	0.09	0.01	0.00	0.02
6	175	0.00	0.00	0.55	0.67	0.00	0.00	0.02	0.00	0.07	0.07	0.00	0.03
6	176	0.01	0.01	0.56	0.60	0.00	0.00	0.01	0.01	0.05	0.02	0.00	0.04
6	177	0.00	0.02	0.65	0.55	0.00	0.00	0.02	0.00	0.03	0.00	0.00	0.06
6	178	0.00	0.02	0.66	0.49	0.00	0.00	0.02	0.02	0.02	0.08	0.00	0.07
6	179	0.00	0.02	0.68	0.43	0.00	0.00	0.02	0.00	0.02	0.06	0.00	0.08
6	180	0.01	0.00	0.68	0.37	0.00	0.00	0.02	0.05	0.03	0.08	0.00	0.09
6	181	0.01	0.00	0.67	0.31	0.00	0.00	0.02	0.05	0.03	0.07	0.00	0.10
6	182	0.00	0.01	0.72	0.25	0.00	0.00	0.02	0.04	0.03	0.05	0.00	0.13
6	183	0.00	0.00	0.76	0.20	0.00	0.00	0.02	0.07	0.02	0.03	0.00	0.15
6	184	0.00	0.00	0.75	0.14	0.00	0.00	0.02	0.00	0.01	0.02	0.00	0.19

6	185	0.00	0.00	0.78	0.05	0.00	0.00	0.02	0.02	0.01	0.08	0.00	0.21
6	186	0.01	0.00	0.78	0.00	0.00	0.00	0.01	0.00	0.01	0.05	0.00	0.23
6	187	0.00	0.03	0.78	0.11	0.00	0.00	0.02	0.03	0.01	0.07	0.00	0.24
6	188	0.00	0.00	0.83	0.37	0.00	0.00	0.02	0.05	0.01	0.06	0.00	0.25
6	189	0.01	0.00	0.85	0.23	0.00	0.00	0.02	0.05	0.01	0.01	0.00	0.30
6	190	0.00	0.00	0.84	0.19	0.00	0.00	0.02	0.00	0.01	0.04	0.00	0.30
7	0	2.03	0.00	0.78	1.10	1.02	0.01	0.48	0.00	0.06	0.00	0.05	0.52
7	1	2.03	0.00	0.78	1.10	1.02	0.01	0.48	0.00	0.06	0.00	0.05	0.52
7	2	1.96	0.20	0.60	1.09	1.02	0.01	0.47	0.00	0.06	0.58	0.09	0.51
7	3	1.94	0.17	0.64	1.07	1.02	0.01	0.46	0.00	0.06	0.53	0.08	0.51
7	4	1.89	0.27	0.58	1.04	1.01	0.01	0.44	0.58	0.06	0.68	0.20	0.47
7	5	1.74	0.30	0.49	1.02	1.00	0.01	0.43	0.54	0.06	0.53	0.26	0.48
7	6	1.62	0.30	0.45	0.99	0.99	0.01	0.41	0.49	0.06	0.65	0.32	0.47
7	7	1.53	0.23	0.27	0.95	0.96	0.01	0.39	0.44	0.06	0.67	0.37	0.46
7	8	1.47	0.40	0.17	0.87	0.95	0.01	0.37	0.39	0.06	0.66	0.43	0.45
7	9	1.38	0.46	0.33	0.86	0.95	0.01	0.35	0.33	0.07	0.66	0.45	0.48
7	10	1.37	0.52	0.33	0.86	0.98	0.01	0.33	0.24	0.07	0.76	0.47	0.53
7	11	1.32	0.55	0.36	0.86	0.99	0.01	0.30	0.18	0.08	0.85	0.48	0.58
7	12	1.20	0.66	0.37	0.89	1.01	0.01	0.28	0.08	0.10	0.86	0.51	0.59
7	13	1.36	0.66	0.35	0.97	1.01	0.00	0.25	0.09	0.12	0.85	0.50	0.55
7	14	1.37	0.90	0.23	0.92	1.02	0.01	0.22	0.11	0.16	1.03	0.64	0.55
7	15	1.29	0.95	0.23	0.79	1.01	0.01	0.19	0.09	0.21	1.07	0.65	0.57
7	16	1.19	0.97	0.18	0.66	0.99	0.01	0.17	0.05	0.26	1.15	0.74	0.58
7	17	1.05	0.96	0.18	0.53	0.96	0.01	0.14	0.05	0.32	1.03	0.72	0.58
7	18	0.92	0.94	0.27	0.39	0.93	0.00	0.12	0.04	0.33	1.06	0.73	0.57
7	19	0.72	0.85	0.16	0.27	0.89	0.01	0.10	0.00	0.33	1.06	0.67	0.56
7	20	0.59	0.88	0.18	0.28	0.84	0.01	0.09	0.04	0.32	0.89	0.68	0.49
7	21	0.62	0.90	0.17	0.33	0.79	0.00	0.09	0.03	0.32	0.98	0.69	0.41
7	22	0.62	0.88	0.09	0.36	0.73	0.00	0.08	0.01	0.31	0.95	0.68	0.34
7	23	0.66	0.88	0.09	0.26	0.66	0.00	0.08	0.02	0.30	0.80	0.61	0.26
7	24	0.56	0.75	0.05	0.25	0.57	0.00	0.08	0.04	0.30	0.66	0.54	0.17
7	25	0.44	0.60	0.02	0.15	0.49	0.00	0.09	0.00	0.27	0.43	0.39	0.07
7	26	0.41	0.53	0.08	0.18	0.41	0.00	0.09	0.00	0.28	0.28	0.40	0.04
7	27	0.37	0.51	0.07	0.19	0.33	0.00	0.10	0.00	0.24	0.35	0.31	0.02
7	28	0.31	0.23	0.08	0.17	0.26	0.00	0.11	0.04	0.20	0.20	0.23	0.01
7	29	0.18	0.11	0.00	0.18	0.19	0.00	0.11	0.04	0.16	0.13	0.14	0.01

7	30	0.28	0.17	0.00	0.16	0.14	0.00	0.11	0.00	0.12	0.08	0.08	0.00
7	31	0.00	0.29	0.09	0.17	0.09	0.00	0.12	0.00	0.09	0.01	0.00	0.00
7	32	0.07	0.14	0.00	0.14	0.07	0.00	0.14	0.00	0.10	0.21	0.03	0.01
7	33	0.01	0.21	0.00	0.13	0.07	0.00	0.26	0.08	0.13	0.13	0.02	0.00
7	34	0.36	0.24	0.08	0.11	0.06	0.00	0.40	0.09	0.16	0.28	0.00	0.00
7	35	0.35	0.10	0.09	0.09	0.06	0.00	0.52	0.15	0.19	0.33	0.03	0.00
7	36	0.16	0.20	0.08	0.09	0.05	0.00	0.51	0.22	0.22	0.33	0.06	0.00
7	37	0.19	0.23	0.09	0.10	0.05	0.00	0.46	0.25	0.25	0.38	0.07	0.00
7	38	0.41	0.14	0.04	0.12	0.06	0.00	0.41	0.21	0.27	0.38	0.07	0.00
7	39	0.37	0.01	0.00	0.14	0.07	0.00	0.36	0.32	0.30	0.45	0.11	0.00
7	40	0.37	0.20	0.00	0.11	0.09	0.00	0.30	0.36	0.36	0.40	0.16	0.00
7	41	0.37	0.32	0.03	0.10	0.10	0.00	0.25	0.44	0.42	0.47	0.20	0.00
7	42	0.33	0.26	0.05	0.07	0.11	0.00	0.20	0.54	0.48	0.62	0.24	0.00
7	43	0.24	0.26	0.09	0.07	0.13	0.00	0.15	0.58	0.55	0.48	0.27	0.00
7	44	0.21	0.35	0.07	0.05	0.14	0.00	0.11	0.71	0.60	0.60	0.27	0.00
7	45	0.25	0.20	0.08	0.05	0.15	0.00	0.10	0.76	0.60	0.53	0.19	0.01
7	46	0.24	0.21	0.04	0.05	0.16	0.00	0.09	0.75	0.60	0.58	0.11	0.01
7	47	0.08	0.10	0.00	0.04	0.16	0.00	0.07	0.73	0.60	0.44	0.17	0.03
7	48	0.03	0.25	0.00	0.04	0.17	0.00	0.06	0.70	0.59	0.59	0.14	0.03
7	49	0.07	0.23	0.00	0.02	0.18	0.00	0.05	0.67	0.58	0.57	0.13	0.06
7	50	0.16	0.00	0.00	0.00	0.18	0.00	0.03	0.62	0.56	0.53	0.04	0.08
7	51	0.04	0.24	0.10	0.01	0.19	0.00	0.02	0.57	0.54	0.41	0.08	0.10
7	52	0.00	0.18	0.00	0.00	0.19	0.00	0.02	0.51	0.52	0.35	0.06	0.13
7	53	0.26	0.18	0.10	0.02	0.19	0.00	0.04	0.42	0.50	0.31	0.06	0.14
7	54	0.27	0.28	0.00	0.03	0.19	0.00	0.07	0.36	0.48	0.46	0.12	0.17
7	55	0.41	0.11	0.08	0.05	0.19	0.00	0.11	0.30	0.46	0.30	0.12	0.20
7	56	0.47	0.23	0.00	0.07	0.18	0.00	0.17	0.33	0.45	0.15	0.09	0.25
7	57	0.53	0.20	0.00	0.09	0.16	0.00	0.24	0.36	0.43	0.38	0.10	0.30
7	58	0.58	0.26	0.11	0.08	0.15	0.00	0.32	0.39	0.40	0.16	0.02	0.31
7	59	0.55	0.15	0.08	0.07	0.14	0.00	0.39	0.40	0.36	0.00	0.00	0.30
7	60	0.60	0.23	0.10	0.08	0.12	0.00	0.43	0.30	0.35	0.19	0.07	0.27
7	61	0.55	0.17	0.07	0.09	0.13	0.00	0.47	0.35	0.33	0.09	0.05	0.22
7	62	0.43	0.19	0.08	0.07	0.16	0.00	0.50	0.23	0.29	0.10	0.06	0.20
7	63	0.45	0.00	0.05	0.07	0.19	0.00	0.53	0.20	0.27	0.00	0.10	0.17
7	64	0.44	0.11	0.08	0.10	0.23	0.00	0.57	0.16	0.24	0.17	0.14	0.17
7	65	0.45	0.06	0.00	0.13	0.25	0.00	0.59	0.11	0.20	0.21	0.05	0.17

7	66	0.41	0.10	0.08	0.14	0.30	0.00	0.61	0.05	0.18	0.24	0.15	0.14
7	67	0.41	0.17	0.08	0.15	0.33	0.00	0.62	0.04	0.15	0.25	0.12	0.14
7	68	0.38	0.15	0.09	0.20	0.36	0.00	0.62	0.00	0.12	0.19	0.22	0.13
7	69	0.31	0.12	0.02	0.28	0.38	0.00	0.62	0.04	0.10	0.25	0.18	0.09
7	70	0.22	0.00	0.09	0.41	0.37	0.00	0.61	0.00	0.06	0.21	0.23	0.04
7	71	0.31	0.00	0.08	0.48	0.36	0.00	0.62	0.00	0.03	0.15	0.18	0.02
7	72	0.41	0.00	0.04	0.49	0.34	0.00	0.65	0.03	0.01	0.07	0.07	0.00
7	73	0.34	0.00	0.03	0.51	0.33	0.00	0.67	0.00	0.00	0.00	0.07	0.00
7	74	0.41	0.02	0.09	0.51	0.32	0.00	0.69	0.04	0.00	0.01	0.03	0.00
7	75	0.47	0.03	0.00	0.55	0.30	0.00	0.70	0.03	0.00	0.00	0.00	0.00
7	76	0.48	0.02	0.07	0.56	0.28	0.00	0.69	0.00	0.01	0.00	0.01	0.00
7	77	0.56	0.00	0.08	0.56	0.26	0.00	0.70	0.02	0.00	0.05	0.01	0.00
7	78	0.48	0.02	0.09	0.55	0.24	0.00	0.69	0.00	0.01	0.00	0.01	0.01
7	79	0.51	0.00	0.00	0.52	0.22	0.00	0.67	0.05	0.01	0.02	0.01	0.00
7	80	0.48	0.03	0.12	0.46	0.20	0.00	0.65	0.04	0.01	0.00	0.01	0.00
7	81	0.00	0.00	0.00	0.42	0.17	0.00	0.62	0.04	0.00	0.00	0.01	0.00
7	82	0.14	0.00	0.13	0.38	0.14	0.00	0.64	0.03	0.00	0.00	0.00	0.00
7	83	0.13	0.00	0.13	0.36	0.10	0.00	0.63	0.00	0.01	0.00	0.01	0.00
7	84	0.00	0.00	0.00	0.36	0.09	0.00	0.62	0.01	0.00	0.00	0.01	0.00
7	85	0.00	0.00	0.00	0.32	0.06	0.00	0.59	0.04	0.01	0.01	0.01	0.00
7	86	0.00	0.00	0.00	0.27	0.05	0.00	0.57	0.00	0.01	0.00	0.00	0.00
7	87	0.00	0.00	0.08	0.27	0.03	0.00	0.53	0.04	0.01	0.00	0.00	0.00
7	88	0.00	0.00	0.00	0.24	0.01	0.00	0.49	0.04	0.01	0.00	0.01	0.00
7	89	0.00	0.00	0.00	0.20	0.01	0.00	0.45	0.04	0.01	0.01	0.00	0.00
7	90	0.00	0.02	0.05	0.18	0.01	0.00	0.40	0.00	0.01	0.01	0.01	0.00
7	91	0.00	0.01	0.09	0.20	0.01	0.00	0.40	0.02	0.01	0.00	0.01	0.00
7	92	0.01	0.02	0.07	0.31	0.02	0.00	0.39	0.00	0.01	0.00	0.01	0.00
7	93	0.07	0.03	0.06	0.37	0.02	0.00	0.37	0.00	0.01	0.00	0.00	0.00
7	94	0.00	0.03	0.08	0.36	0.02	0.00	0.36	0.00	0.01	0.00	0.00	0.00
7	95	0.00	0.00	0.08	0.31	0.05	0.00	0.35	0.04	0.00	0.00	0.00	0.00
7	96	0.04	0.00	0.03	0.30	0.06	0.00	0.33	0.00	0.01	0.03	0.00	0.00
7	97	0.05	0.00	0.06	0.37	0.07	0.00	0.31	0.03	0.01	0.00	0.01	0.00
7	98	0.04	0.00	0.07	0.40	0.07	0.00	0.28	0.04	0.01	0.00	0.00	0.00
7	99	0.08	0.00	0.07	0.39	0.09	0.00	0.23	0.00	0.01	0.01	0.01	0.00
7	100	0.09	0.00	0.03	0.39	0.11	0.00	0.18	0.00	0.01	0.00	0.00	0.00
7	101	0.00	0.02	0.07	0.40	0.18	0.00	0.13	0.04	0.01	0.00	0.00	0.00

7	102	0.00	0.02	0.07	0.41	0.26	0.00	0.09	0.00	0.01	0.00	0.03	0.00
7	103	0.11	0.00	0.04	0.44	0.35	0.00	0.06	0.02	0.01	0.00	0.03	0.00
7	104	0.15	0.01	0.03	0.43	0.40	0.00	0.04	0.00	0.01	0.00	0.05	0.00
7	105	0.09	0.01	0.09	0.44	0.39	0.00	0.02	0.00	0.01	0.00	0.08	0.00
7	106	0.06	0.06	0.09	0.41	0.37	0.00	0.01	0.04	0.01	0.00	0.07	0.00
7	107	0.00	0.01	0.09	0.43	0.36	0.00	0.02	0.02	0.01	0.00	0.07	0.00
7	108	0.09	0.00	0.07	0.45	0.34	0.00	0.02	0.00	0.01	0.00	0.05	0.00
7	109	0.08	0.00	0.02	0.45	0.31	0.00	0.02	0.04	0.01	0.00	0.04	0.00
7	110	0.05	0.00	0.07	0.42	0.29	0.00	0.01	0.03	0.01	0.00	0.05	0.00
7	111	0.02	0.02	0.09	0.40	0.27	0.00	0.01	0.02	0.00	0.00	0.01	0.00
7	112	0.00	0.02	0.09	0.46	0.29	0.00	0.02	0.00	0.00	0.00	0.07	0.00
7	113	0.00	0.00	0.09	0.46	0.35	0.00	0.02	0.00	0.00	0.00	0.12	0.00
7	114	0.00	0.00	0.08	0.43	0.42	0.00	0.02	0.00	0.00	0.00	0.12	0.00
7	115	0.08	0.01	0.08	0.41	0.49	0.00	0.02	0.00	0.01	0.00	0.14	0.00
7	116	0.00	0.02	0.04	0.37	0.55	0.00	0.01	0.00	0.00	0.00	0.14	0.00
7	117	0.00	0.00	0.04	0.33	0.63	0.00	0.02	0.04	0.01	0.00	0.16	0.00
7	118	0.00	0.00	0.03	0.30	0.68	0.00	0.01	0.00	0.01	0.00	0.08	0.00
7	119	0.00	0.00	0.04	0.27	0.74	0.00	0.02	0.00	0.01	0.01	0.09	0.00
7	120	0.00	0.00	0.09	0.28	0.81	0.00	0.02	0.04	0.00	0.00	0.05	0.01
7	121	0.00	0.01	0.07	0.27	0.86	0.00	0.00	0.01	0.01	0.00	0.03	0.03
7	122	0.00	0.01	0.09	0.26	0.90	0.00	0.02	0.00	0.00	0.00	0.01	0.06
7	123	0.00	0.00	0.08	0.25	0.88	0.00	0.02	0.00	0.01	0.00	0.00	0.07
7	124	0.00	0.03	0.00	0.28	0.81	0.00	0.02	0.00	0.00	0.00	0.01	0.08
7	125	0.00	0.00	0.06	0.26	0.72	0.00	0.02	0.01	0.01	0.00	0.01	0.08
7	126	0.01	0.00	0.06	0.24	0.63	0.00	0.02	0.03	0.01	0.00	0.01	0.08
7	127	0.00	0.00	0.01	0.19	0.53	0.01	0.02	0.00	0.01	0.00	0.01	0.06
7	128	0.00	0.02	0.08	0.14	0.44	0.02	0.02	0.00	0.01	0.00	0.01	0.02
7	129	0.00	0.00	0.01	0.12	0.34	0.03	0.02	0.02	0.01	0.00	0.00	0.01
7	130	0.00	0.00	0.00	0.10	0.26	0.03	0.02	0.00	0.01	0.00	0.01	0.06
7	131	0.00	0.00	0.00	0.07	0.19	0.04	0.02	0.00	0.01	0.00	0.01	0.09
7	132	0.00	0.00	0.00	0.04	0.15	0.04	0.02	0.00	0.00	0.00	0.00	0.13
7	133	0.00	0.00	0.04	0.00	0.11	0.04	0.02	0.00	0.01	0.00	0.01	0.18
7	134	0.00	0.00	0.17	0.00	0.08	0.04	0.02	0.00	0.00	0.01	0.01	0.19
7	135	0.00	0.00	0.10	0.01	0.05	0.04	0.02	0.00	0.01	0.02	0.00	0.16
7	136	0.00	0.00	0.09	0.00	0.03	0.04	0.02	0.04	0.01	0.00	0.01	0.13
7	137	0.00	0.00	0.00	0.00	0.01	0.03	0.02	0.00	0.00	0.00	0.01	0.11

7	138	0.00	0.00	0.00	0.00	0.00	0.03	0.02	0.00	0.01	0.00	0.01	0.09
7	139	0.00	0.00	0.00	0.00	0.00	0.04	0.01	0.00	0.00	0.00	0.01	0.09
7	140	0.00	0.00	0.08	0.00	0.00	0.04	0.02	0.00	0.01	0.01	0.01	0.09
7	141	0.00	0.00	0.04	0.00	0.00	0.03	0.02	0.03	0.01	0.00	0.01	0.09
7	142	0.00	0.00	0.00	0.00	0.00	0.03	0.01	0.00	0.01	0.00	0.00	0.10
7	143	0.00	0.00	0.07	0.00	0.00	0.02	0.00	0.00	0.01	0.00	0.01	0.10
7	144	0.00	0.00	0.06	0.00	0.00	0.01	0.01	0.00	0.00	0.01	0.01	0.09
7	145	0.00	0.00	0.08	0.00	0.01	0.00	0.01	0.00	0.01	0.00	0.01	0.09
7	146	0.00	0.00	0.00	0.00	0.01	0.00	0.01	0.00	0.00	0.00	0.01	0.07
7	147	0.00	0.00	0.04	0.00	0.01	0.00	0.01	0.00	0.00	0.01	0.01	0.07
7	148	0.00	0.00	0.01	0.00	0.02	0.00	0.02	0.00	0.01	0.00	0.01	0.05
7	149	0.00	0.00	0.07	0.00	0.02	0.00	0.02	0.00	0.01	0.00	0.01	0.04
7	150	0.02	0.00	0.07	0.00	0.03	0.00	0.01	0.00	0.01	0.01	0.01	0.03
7	151	0.00	0.02	0.07	0.00	0.03	0.00	0.02	0.00	0.01	0.00	0.01	0.03
7	152	0.00	0.02	0.00	0.00	0.03	0.00	0.02	0.00	0.01	0.00	0.01	0.03
7	153	0.00	0.03	0.08	0.00	0.02	0.00	0.02	0.00	0.01	0.00	0.01	0.04
7	154	0.01	0.00	0.09	0.00	0.02	0.00	0.02	0.00	0.01	0.00	0.01	0.05
7	155	0.02	0.01	0.00	0.00	0.01	0.00	0.02	0.00	0.01	0.00	0.01	0.05
7	156	0.00	0.00	0.00	0.00	0.01	0.00	0.02	0.00	0.01	0.00	0.01	0.06
7	157	0.00	0.00	0.08	0.00	0.00	0.00	0.02	0.00	0.01	0.00	0.01	0.06
7	158	0.01	0.00	0.00	0.00	0.00	0.00	0.02	0.00	0.01	0.00	0.01	0.06
7	159	0.00	0.00	0.00	0.02	0.00	0.00	0.01	0.00	0.01	0.00	0.00	0.06
7	160	0.00	0.01	0.00	0.04	0.00	0.00	0.02	0.00	0.01	0.00	0.01	0.05
7	161	0.00	0.02	0.08	0.03	0.00	0.00	0.02	0.00	0.01	0.00	0.01	0.05
7	162	0.00	0.02	0.00	0.12	0.00	0.00	0.02	0.02	0.01	0.00	0.01	0.03
7	163	0.00	0.00	0.08	0.14	0.00	0.00	0.02	0.00	0.01	0.00	0.01	0.07
7	164	0.02	0.00	0.08	0.16	0.00	0.00	0.01	0.00	0.01	0.00	0.01	0.08
7	165	0.00	0.03	0.07	0.15	0.00	0.00	0.02	0.00	0.01	0.02	0.01	0.07
7	166	0.00	0.00	0.07	0.17	0.00	0.00	0.02	0.04	0.01	0.00	0.01	0.09
7	167	0.00	0.02	0.08	0.16	0.00	0.00	0.02	0.01	0.01	0.00	0.01	0.13
7	168	0.00	0.00	0.04	0.15	0.00	0.00	0.02	0.02	0.00	0.01	0.01	0.14
7	169	0.01	0.00	0.05	0.13	0.00	0.00	0.02	0.00	0.01	0.00	0.01	0.15
7	170	0.02	0.00	0.07	0.11	0.00	0.00	0.02	0.00	0.00	0.00	0.01	0.16
7	171	0.00	0.05	0.10	0.09	0.00	0.00	0.02	0.00	0.01	0.00	0.01	0.15
7	172	0.00	0.00	0.05	0.08	0.00	0.00	0.02	0.00	0.01	0.00	0.01	0.14
7	173	0.00	0.00	0.15	0.04	0.00	0.00	0.02	0.04	0.01	0.00	0.01	0.13

7	174	0.00	0.00	0.19	0.02	0.00	0.00	0.02	0.00	0.01	0.00	0.00	0.12
7	175	0.00	0.13	0.15	0.00	0.00	0.00	0.03	0.07	0.01	0.00	0.01	0.10
7	176	0.00	0.19	0.11	0.00	0.00	0.00	0.04	0.07	0.01	0.00	0.01	0.08
7	177	0.00	0.25	0.08	0.00	0.00	0.00	0.03	0.04	0.01	0.00	0.01	0.04
7	178	0.00	0.14	0.01	0.00	0.00	0.00	0.02	0.07	0.01	0.01	0.01	0.02
7	179	0.00	0.14	0.10	0.00	0.00	0.00	0.02	0.06	0.01	0.00	0.00	0.00
7	180	0.00	0.21	0.00	0.00	0.00	0.00	0.02	0.05	0.01	0.00	0.01	0.00
7	181	0.00	0.18	0.00	0.00	0.00	0.00	0.02	0.03	0.01	0.01	0.01	0.00
7	182	0.00	0.14	0.07	0.00	0.00	0.00	0.02	0.04	0.01	0.00	0.00	0.00
7	183	0.00	0.00	0.05	0.00	0.00	0.00	0.02	0.00	0.00	0.00	0.00	0.00
7	184	0.00	0.09	0.00	0.00	0.00	0.00	0.00	0.00	0.00	0.00	0.00	0.00
7	185	0.00	0.14	0.00	0.00	0.00	0.00	0.00	0.00	0.00	0.01	0.00	0.00
8	0	0.58	0.07	0.00	0.00	0.00	0.00	0.70	0.00	0.01	0.08	0.19	0.03
8	1	0.58	0.07	0.00	0.00	0.00	0.00	0.70	0.00	0.01	0.08	0.19	0.03
8	2	0.61	0.07	0.02	0.00	0.00	0.00	0.72	0.00	0.00	0.09	0.16	0.03
8	3	0.37	0.14	0.00	0.40	0.00	1.86	0.73	0.01	0.01	0.02	0.09	0.00
8	4	0.39	0.27	0.00	0.43	0.00	1.99	0.75	0.00	0.01	0.05	0.15	0.00
8	5	0.52	0.00	0.08	0.46	0.00	2.13	0.77	0.00	0.01	0.00	0.08	0.03
8	6	0.50	0.00	0.09	0.50	0.00	2.22	0.78	0.00	0.00	0.03	0.12	0.02
8	7	0.26	0.01	0.00	0.52	0.00	2.35	0.80	0.00	0.00	0.10	0.12	0.02
8	8	0.42	0.00	0.00	0.56	0.00	2.41	0.81	0.00	0.01	0.04	0.09	0.02
8	9	0.36	0.00	0.01	0.60	0.00	2.42	0.82	0.00	0.01	0.09	0.11	0.02
8	10	0.56	0.02	0.00	0.63	0.00	2.41	0.82	0.03	0.01	0.08	0.10	0.01
8	11	0.42	0.00	0.09	0.66	0.00	2.36	0.82	0.00	0.01	0.06	0.08	0.01
8	12	0.59	0.01	0.02	0.67	0.00	2.24	0.82	0.02	0.01	0.00	0.06	0.03
8	13	0.40	0.00	0.00	0.68	0.00	2.13	0.81	0.01	0.01	0.00	0.05	0.04
8	14	0.54	0.00	0.03	0.67	0.00	1.99	0.80	0.04	0.01	0.02	0.03	0.03
8	15	0.54	0.00	0.05	0.67	0.01	1.83	0.77	0.00	0.01	0.00	0.00	0.04
8	16	0.51	0.01	0.09	0.65	0.01	1.68	0.76	0.00	0.00	0.00	0.00	0.06
8	17	0.38	0.01	0.13	0.63	0.00	1.50	0.75	0.00	0.00	0.00	0.00	0.09
8	18	0.43	0.02	0.27	0.60	0.00	1.31	0.74	0.00	0.01	0.00	0.00	0.11
8	19	0.49	0.00	0.32	0.54	0.00	1.12	0.71	0.00	0.01	0.00	0.00	0.15
8	20	0.25	0.00	0.23	0.54	0.00	0.98	0.69	0.00	0.01	0.00	0.01	0.19
8	21	0.40	0.02	0.34	0.50	0.00	0.97	0.67	0.00	0.01	0.00	0.01	0.24
8	22	0.38	0.00	0.23	0.45	0.00	0.95	0.64	0.00	0.00	0.00	0.01	0.27
8	23	0.38	0.01	0.14	0.41	0.00	0.94	0.52	0.00	0.01	0.00	0.01	0.18

8	24	0.25	0.00	0.28	0.49	0.00	0.90	0.35	0.00	0.01	0.01	0.01	0.17
8	25	0.53	0.00	0.57	0.55	0.00	0.88	0.23	0.00	0.00	0.00	0.01	0.22
8	26	0.57	0.00	0.69	0.63	0.00	0.83	0.20	0.04	0.01	0.00	0.01	0.23
8	27	0.56	0.00	0.71	0.48	0.00	0.77	0.19	0.00	0.00	0.00	0.01	0.22
8	28	0.44	0.00	0.68	0.39	0.00	0.70	0.19	0.03	0.01	0.00	0.01	0.19
8	29	0.41	0.00	0.64	0.31	0.00	0.63	0.18	0.04	0.00	0.00	0.01	0.20
8	30	0.33	0.00	0.60	0.23	0.00	0.57	0.17	0.04	0.01	0.00	0.01	0.19
8	31	0.25	0.00	0.55	0.14	0.00	0.50	0.16	0.00	0.01	0.00	0.01	0.19
8	32	0.14	0.02	0.52	0.08	0.00	0.44	0.18	0.00	0.01	0.00	0.01	0.18
8	33	0.11	0.00	0.45	0.03	0.00	0.38	0.21	0.02	0.01	0.00	0.01	0.18
8	34	0.13	0.00	0.42	0.01	0.00	0.34	0.23	0.03	0.00	0.00	0.01	0.17
8	35	0.15	0.03	0.38	0.00	0.00	0.31	0.25	0.02	0.00	0.00	0.01	0.17
8	36	0.15	0.02	0.42	0.00	0.00	0.30	0.27	0.03	0.01	0.00	0.01	0.21
8	37	0.18	0.01	0.43	0.00	0.00	0.27	0.29	0.06	0.00	0.00	0.01	0.26
8	38	0.18	0.00	0.46	0.00	0.00	0.28	0.30	0.10	0.01	0.00	0.01	0.31
8	39	0.20	0.03	0.47	0.00	0.00	0.26	0.30	0.13	0.04	0.00	0.01	0.35
8	40	0.21	0.00	0.48	0.00	0.00	0.23	0.30	0.03	0.08	0.00	0.01	0.38
8	41	0.19	0.00	0.49	0.00	0.00	0.23	0.29	0.18	0.12	0.00	0.01	0.40
8	42	0.19	0.02	0.48	0.00	0.01	0.21	0.29	0.22	0.17	0.00	0.01	0.41
8	43	0.21	0.00	0.47	0.00	0.02	0.18	0.28	0.38	0.23	0.00	0.01	0.42
8	44	0.18	0.01	0.31	0.00	0.02	0.20	0.26	0.51	0.27	0.00	0.01	0.41
8	45	0.25	0.00	0.46	0.00	0.02	0.22	0.25	0.61	0.26	0.00	0.01	0.38
8	46	0.08	0.00	0.31	0.00	0.02	0.24	0.23	0.70	0.25	0.00	0.01	0.37
8	47	0.04	0.00	0.43	0.00	0.02	0.26	0.25	0.80	0.24	0.00	0.01	0.35
8	48	0.17	0.00	0.48	0.00	0.02	0.28	0.29	0.89	0.23	0.00	0.01	0.34
8	49	0.22	0.00	0.50	0.00	0.01	0.30	0.32	0.95	0.22	0.00	0.01	0.33
8	50	0.18	0.02	0.54	0.00	0.01	0.32	0.35	1.06	0.21	0.00	0.01	0.32
8	51	0.26	0.00	0.56	0.00	0.01	0.34	0.37	1.10	0.20	0.00	0.01	0.31
8	52	0.22	0.02	0.54	0.00	0.01	0.35	0.39	1.13	0.19	0.08	0.00	0.32
8	53	0.19	0.00	0.50	0.00	0.01	0.37	0.40	1.20	0.18	0.11	0.00	0.33
8	54	0.25	0.07	0.60	0.00	0.01	0.39	0.37	1.21	0.17	0.15	0.00	0.31
8	55	0.35	0.07	0.63	0.00	0.01	0.44	0.33	1.27	0.13	0.20	0.00	0.35
8	56	0.42	0.02	0.73	0.00	0.00	0.50	0.28	1.32	0.11	0.17	0.00	0.38
8	57	0.28	0.02	0.79	0.00	0.00	0.57	0.24	1.39	0.08	0.16	0.00	0.43
8	58	0.47	0.00	0.89	0.00	0.00	0.63	0.20	1.47	0.07	0.25	0.00	0.49
8	59	0.51	0.00	1.01	0.00	0.00	0.69	0.16	1.54	0.05	0.29	0.00	0.54

8	60	0.54	0.03	1.01	0.00	0.00	0.75	0.12	1.61	0.03	0.24	0.01	0.62
8	61	0.32	0.00	1.23	0.00	0.00	0.81	0.09	1.66	0.02	0.16	0.00	0.68
8	62	0.54	0.00	1.32	0.00	0.00	0.87	0.07	1.74	0.01	0.31	0.01	0.78
8	63	0.50	0.00	1.45	0.00	0.00	0.92	0.04	1.72	0.01	0.28	0.01	0.86
8	64	0.51	0.01	1.49	0.00	0.00	0.97	0.03	1.70	0.00	0.24	0.01	0.93
8	65	0.56	0.00	1.60	0.00	0.00	1.01	0.02	1.73	0.01	0.22	0.01	1.00
8	66	0.41	0.00	1.71	0.02	0.00	1.04	0.02	1.68	0.01	0.27	0.01	1.10
8	67	0.80	0.03	1.86	0.08	0.00	1.08	0.02	1.69	0.01	0.29	0.01	1.20
8	68	1.00	0.00	1.96	0.13	0.00	1.11	0.03	1.64	0.02	0.35	0.01	1.28
8	69	1.15	0.04	2.17	0.21	0.00	1.26	0.03	1.56	0.03	0.39	0.00	1.45
8	70	1.08	0.00	2.27	0.28	0.00	1.38	0.03	1.48	0.03	0.35	0.00	1.55
8	71	1.46	0.14	2.42	0.37	0.01	1.51	0.04	1.40	0.02	0.36	0.01	1.57
8	72	1.64	0.10	2.55	0.48	0.01	1.61	0.04	1.29	0.02	0.36	0.00	1.48
8	73	1.82	0.41	2.61	0.65	0.03	1.68	0.04	1.21	0.02	0.50	0.00	1.33
8	74	1.97	0.43	2.72	0.78	0.02	1.75	0.04	1.14	0.01	0.51	0.04	1.28
8	75	2.11	0.45	2.97	0.91	0.04	1.82	0.04	1.06	0.01	0.54	0.04	1.35
8	76	2.16	0.45	3.18	1.01	0.04	1.88	0.05	1.00	0.01	0.56	0.02	1.42
8	77	2.19	0.45	3.29	1.08	0.04	1.95	0.05	0.92	0.01	0.53	0.00	1.48
8	78	2.29	0.13	3.32	1.10	0.04	2.00	0.05	0.86	0.01	0.43	0.00	1.48
8	79	2.35	0.48	3.33	1.13	0.04	2.07	0.06	0.79	0.01	0.33	0.00	1.48
8	80	2.42	0.39	3.27	1.14	0.04	2.14	0.06	0.76	0.01	0.37	0.00	1.48
8	81	2.50	0.57	3.31	1.12	0.05	2.19	0.06	0.77	0.02	0.37	0.01	1.48
8	82	2.48	0.60	3.24	1.10	0.05	2.23	0.06	0.77	0.03	0.37	0.01	1.48
8	83	2.55	0.63	3.23	1.05	0.05	2.25	0.06	0.70	0.04	0.31	0.00	1.47
8	84	2.51	0.58	3.08	1.00	0.04	2.26	0.05	0.66	0.04	0.11	0.00	1.45
8	85	2.44	0.53	3.09	0.99	0.05	2.25	0.06	0.56	0.06	0.26	0.00	1.43
8	86	2.51	0.63	3.09	1.00	0.05	2.22	0.06	0.59	0.07	0.21	0.00	1.44
8	87	2.51	0.75	3.04	1.00	0.06	2.19	0.06	0.52	0.10	0.20	0.00	1.43
8	88	2.54	0.77	2.99	0.98	0.06	2.14	0.09	0.39	0.11	0.19	0.00	1.43
8	89	2.60	0.72	2.75	0.91	0.06	2.06	0.13	0.35	0.14	0.13	0.00	1.40
8	90	2.61	0.67	2.76	0.89	0.06	2.00	0.18	0.27	0.16	0.07	0.04	1.35
8	91	2.81	0.49	2.57	0.96	0.06	1.91	0.24	0.31	0.20	0.39	0.09	1.27
8	92	2.92	0.76	2.21	1.00	0.06	1.81	0.30	0.25	0.24	0.64	0.15	1.18
8	93	3.11	0.91	2.15	1.06	0.06	1.71	0.36	0.28	0.28	0.78	0.21	1.06
8	94	3.14	1.23	1.89	1.08	0.06	1.61	0.41	0.26	0.33	0.90	0.30	0.95
8	95	3.21	1.36	1.61	1.08	0.06	1.51	0.47	0.23	0.37	1.04	0.40	0.83

8	96	3.17	1.49	1.34	1.06	0.06	1.42	0.51	0.22	0.41	1.12	0.50	0.66
8	97	3.28	1.58	1.07	0.96	0.06	1.32	0.52	0.22	0.45	1.25	0.57	0.51
8	98	3.10	1.53	0.88	0.87	0.06	1.26	0.50	0.16	0.50	1.28	0.65	0.48
8	99	2.84	1.44	0.70	0.78	0.07	1.23	0.46	0.16	0.54	1.10	0.61	0.37
8	100	2.72	1.46	0.55	0.69	0.07	1.17	0.42	0.19	0.60	1.06	0.58	0.30
8	101	2.50	1.39	0.41	0.60	0.07	1.10	0.38	0.17	0.64	0.92	0.55	0.24
8	102	2.21	1.31	0.31	0.48	0.07	1.02	0.34	0.16	0.66	0.78	0.47	0.18
8	103	2.04	1.18	0.23	0.40	0.08	0.96	0.30	0.12	0.71	0.63	0.42	0.13
8	104	1.84	1.08	0.12	0.32	0.08	0.88	0.26	0.11	0.76	0.46	0.33	0.09
8	105	1.69	0.92	0.01	0.31	0.08	0.80	0.23	0.09	0.78	0.41	0.31	0.08
8	106	1.53	0.91	0.14	0.28	0.08	0.72	0.21	0.06	0.82	0.34	0.25	0.05
8	107	1.46	0.71	0.06	0.26	0.08	0.64	0.18	0.06	0.85	0.29	0.20	0.06
8	108	1.29	0.74	0.20	0.23	0.08	0.56	0.16	0.04	0.82	0.18	0.14	0.04
8	109	1.12	0.78	0.26	0.21	0.08	0.49	0.14	0.02	0.88	0.10	0.13	0.03
8	110	1.09	0.75	0.00	0.19	0.08	0.40	0.11	0.00	0.88	0.12	0.01	0.01
8	111	0.97	0.80	0.01	0.17	0.07	0.36	0.09	0.00	0.87	0.11	0.08	0.01
8	112	0.61	0.78	0.00	0.14	0.07	0.31	0.08	0.02	0.85	0.06	0.08	0.00
8	113	0.52	0.45	0.13	0.11	0.05	0.25	0.06	0.00	0.83	0.11	0.04	0.00
8	114	0.11	0.42	0.00	0.08	0.03	0.20	0.05	0.00	0.81	0.00	0.02	0.00
8	115	0.61	0.52	0.00	0.05	0.02	0.15	0.05	0.01	0.78	0.05	0.00	0.00
8	116	0.49	0.32	0.00	0.03	0.01	0.10	0.04	0.02	0.74	0.02	0.00	0.00
8	117	0.40	0.30	0.00	0.00	0.00	0.07	0.03	0.02	0.69	0.00	0.00	0.00
8	118	0.12	0.33	0.00	0.00	0.00	0.04	0.03	0.04	0.65	0.00	0.00	0.00
8	119	0.05	0.15	0.00	0.00	0.00	0.02	0.02	0.03	0.59	0.00	0.00	0.00
8	120	0.22	0.23	0.00	0.00	0.00	0.01	0.03	0.04	0.53	0.00	0.01	0.00
8	121	0.23	0.32	0.08	0.00	0.00	0.00	0.04	0.01	0.47	0.00	0.00	0.00
8	122	0.25	0.10	0.07	0.01	0.00	0.00	0.05	0.03	0.41	0.00	0.00	0.00
8	123	0.29	0.05	0.09	0.03	0.00	0.00	0.06	0.02	0.35	0.00	0.00	0.00
8	124	0.29	0.08	0.09	0.08	0.00	0.00	0.08	0.03	0.30	0.00	0.01	0.00
8	125	0.20	0.15	0.08	0.09	0.00	0.00	0.10	0.00	0.28	0.01	0.01	0.00
8	126	0.33	0.17	0.07	0.10	0.00	0.00	0.11	0.00	0.32	0.00	0.01	0.00
8	127	0.40	0.14	0.09	0.11	0.00	0.00	0.13	0.00	0.35	0.00	0.00	0.00
8	128	0.36	0.18	0.00	0.14	0.00	0.00	0.15	0.00	0.38	0.00	0.00	0.00
8	129	0.40	0.16	0.00	0.19	0.00	0.00	0.17	0.04	0.41	0.00	0.00	0.00
8	130	0.35	0.17	0.07	0.19	0.00	0.00	0.19	0.00	0.46	0.00	0.00	0.00
8	131	0.50	0.09	0.09	0.20	0.00	0.00	0.21	0.00	0.49	0.01	0.00	0.00

8	132	0.51	0.00	0.09	0.18	0.00	0.00	0.22	0.01	0.53	0.00	0.00	0.00
8	133	0.52	0.16	0.08	0.20	0.00	0.00	0.23	0.00	0.56	0.05	0.00	0.00
8	134	0.60	0.16	0.06	0.18	0.00	0.00	0.24	0.00	0.58	0.00	0.03	0.00
8	135	0.65	0.18	0.00	0.20	0.00	0.00	0.24	0.04	0.58	0.14	0.00	0.00
8	136	0.58	0.24	0.00	0.16	0.00	0.00	0.24	0.04	0.63	0.17	0.08	0.00
8	137	0.60	0.24	0.07	0.14	0.00	0.00	0.26	0.03	0.64	0.14	0.11	0.00
8	138	0.53	0.30	0.00	0.10	0.00	0.00	0.27	0.00	0.64	0.16	0.16	0.00
8	139	0.54	0.39	0.08	0.07	0.00	0.00	0.28	0.05	0.65	0.26	0.17	0.00
8	140	0.46	0.42	0.08	0.04	0.00	0.00	0.30	0.06	0.65	0.14	0.20	0.00
8	141	0.48	0.47	0.08	0.03	0.00	0.00	0.30	0.04	0.61	0.23	0.27	0.00
8	142	0.49	0.38	0.00	0.01	0.00	0.00	0.31	0.03	0.58	0.11	0.29	0.01
8	143	0.48	0.52	0.08	0.01	0.00	0.03	0.32	0.10	0.53	0.21	0.28	0.04
8	144	0.55	0.66	0.08	0.02	0.00	0.06	0.33	0.05	0.48	0.34	0.31	0.06
8	145	0.66	0.67	0.12	0.02	0.00	0.10	0.32	0.08	0.44	0.39	0.34	0.05
8	146	0.71	0.67	0.17	0.06	0.00	0.16	0.34	0.11	0.38	0.40	0.29	0.05
8	147	0.73	0.66	0.17	0.07	0.00	0.20	0.37	0.13	0.33	0.31	0.34	0.03
8	148	0.79	0.66	0.27	0.11	0.00	0.28	0.40	0.18	0.29	0.47	0.36	0.02
8	149	0.92	0.55	0.42	0.13	0.00	0.35	0.35	0.16	0.24	0.45	0.35	0.04
8	150	1.00	0.45	0.52	0.15	0.00	0.41	0.30	0.19	0.20	0.62	0.32	0.06
8	151	1.05	0.42	0.50	0.18	0.00	0.00	0.24	0.23	0.20	0.65	0.31	0.13
8	152	0.96	0.41	0.59	0.17	0.00	0.00	0.19	0.24	0.20	0.68	0.20	0.16
8	153	0.91	0.19	0.61	0.12	0.00	0.00	0.14	0.30	0.20	0.79	0.25	0.18
8	154	0.83	0.36	0.59	0.11	0.00	0.00	0.09	0.39	0.19	0.77	0.28	0.21
8	155	0.81	0.11	0.57	0.12	0.00	0.00	0.06	0.43	0.16	0.92	0.19	0.24
8	156	0.69	0.03	0.46	0.10	0.00	0.00	0.03	0.44	0.19	0.95	0.21	0.26
8	157	0.60	0.08	0.43	0.05	0.00	0.00	0.02	0.48	0.20	0.97	0.24	0.28
8	158	0.51	0.25	0.35	0.06	0.00	0.00	0.02	0.49	0.19	0.98	0.22	0.29
8	159	0.41	0.18	0.31	0.00	0.00	0.00	0.02	0.49	0.20	0.88	0.22	0.30
8	160	0.26	0.01	0.24	0.03	0.00	0.00	0.02	0.50	0.19	0.92	0.11	0.30
8	161	0.22	0.15	0.23	0.00	0.00	0.00	0.01	0.52	0.19	0.86	0.14	0.30
8	162	0.15	0.03	0.19	0.00	0.00	0.00	0.02	0.51	0.20	0.92	0.13	0.30
8	163	0.00	0.17	0.11	0.00	0.00	0.00	0.02	0.53	0.18	0.84	0.15	0.30
8	164	0.00	0.06	0.11	0.00	0.00	0.00	0.02	0.54	0.18	0.83	0.12	0.29
8	165	0.02	0.04	0.06	0.00	0.00	0.00	0.01	0.53	0.00	0.80	0.15	0.28
9	1	0.00	0.00	0.06	0.00	0.00	0.00	0.02	0.43	0.00	0.00	0.00	0.00
9	2	0.00	0.00	0.07	0.00	0.00	0.00	0.02	0.37	0.00	0.00	0.00	0.00

9	3	0.00	0.03	0.08	0.00	0.00	0.00	0.02	0.31	0.01	0.00	0.01	0.00
9	4	0.00	0.00	0.03	0.00	0.00	0.00	0.02	0.20	0.01	0.00	0.01	0.00
9	5	0.00	0.02	0.08	0.00	0.00	0.00	0.02	0.23	0.01	0.00	0.01	0.00
9	6	0.00	0.00	0.08	0.00	0.00	0.00	0.02	0.19	0.01	0.00	0.01	0.00
9	7	0.00	0.02	0.08	0.00	0.00	0.00	0.02	0.13	0.01	0.00	0.01	0.00
9	8	0.00	0.00	0.08	0.00	0.00	0.00	0.02	0.07	0.01	0.00	0.01	0.00
9	9	0.00	0.02	0.06	0.00	0.00	0.00	0.02	0.07	0.01	0.00	0.01	0.00
9	10	0.02	0.00	0.09	0.00	0.00	0.00	0.04	0.15	0.01	0.00	0.00	0.00
9	11	0.00	0.00	0.06	0.00	0.00	0.00	0.10	0.16	0.00	0.00	0.01	0.00
9	12	0.00	0.00	0.07	0.00	0.00	0.01	0.17	0.19	0.00	0.00	0.01	0.00
9	13	0.00	0.00	0.08	0.00	0.01	0.03	0.23	0.20	0.00	0.00	0.01	0.00
9	14	0.03	0.01	0.09	0.00	0.02	0.05	0.28	0.24	0.01	0.00	0.01	0.00
9	15	0.00	0.00	0.06	0.00	0.03	0.07	0.33	0.37	0.01	0.00	0.01	0.00
9	16	0.00	0.00	0.08	0.00	0.05	0.10	0.38	0.36	0.01	0.01	0.01	0.00
9	17	0.00	0.03	0.08	0.00	0.07	0.13	0.43	0.39	0.00	0.00	0.01	0.00
9	18	0.00	0.01	0.09	0.00	0.08	0.14	0.47	0.46	0.01	0.00	0.00	0.00
9	19	0.00	0.00	0.06	0.00	0.08	0.13	0.47	0.53	0.00	0.01	0.01	0.00
9	20	0.00	0.00	0.01	0.00	0.09	0.12	0.48	0.39	0.01	0.00	0.01	0.00
9	21	0.00	0.00	0.09	0.00	0.09	0.10	0.47	0.51	0.00	0.00	0.01	0.00
9	22	0.00	0.01	0.08	0.00	0.10	0.11	0.45	0.36	0.01	0.01	0.01	0.00
9	23	0.00	0.04	0.07	0.00	0.11	0.09	0.43	0.45	0.01	0.00	0.01	0.00
9	24	0.05	0.07	0.03	0.00	0.12	0.08	0.40	0.40	0.01	0.00	0.01	0.00
9	25	0.07	0.06	0.00	0.00	0.12	0.07	0.37	0.30	0.00	0.00	0.01	0.00
9	26	0.03	0.00	0.00	0.00	0.13	0.06	0.34	0.32	0.00	0.00	0.01	0.01
9	27	0.00	0.00	0.07	0.00	0.13	0.04	0.30	0.27	0.01	0.00	0.01	0.03
9	28	0.00	0.00	0.08	0.00	0.14	0.03	0.27	0.25	0.00	0.00	0.01	0.07
9	29	0.00	0.01	0.12	0.00	0.13	0.02	0.24	0.22	0.01	0.00	0.01	0.12
9	30	0.00	0.00	0.14	0.00	0.14	0.01	0.21	0.19	0.01	0.00	0.01	0.14
9	31	0.02	0.04	0.15	0.00	0.14	0.01	0.18	0.19	0.00	0.00	0.01	0.24
9	32	0.00	0.00	0.20	0.00	0.14	0.00	0.15	0.08	0.01	0.01	0.01	0.32
9	33	0.00	0.00	0.24	0.00	0.14	0.00	0.12	0.05	0.00	0.00	0.01	0.39
9	34	0.00	0.00	0.24	0.00	0.13	0.00	0.11	0.09	0.00	0.00	0.01	0.45
9	35	0.00	0.00	0.25	0.00	0.12	0.00	0.09	0.07	0.01	0.01	0.01	0.50
9	36	0.00	0.01	0.30	0.00	0.11	0.00	0.08	0.00	0.00	0.01	0.01	0.53
9	37	0.01	0.00	0.26	0.00	0.09	0.00	0.07	0.00	0.00	0.00	0.00	0.54
9	38	0.00	0.00	0.32	0.00	0.07	0.00	0.07	0.01	0.00	0.01	0.01	0.59

9	39	0.00	0.00	0.29	0.00	0.06	0.00	0.06	0.00	0.00	0.00	0.01	0.61
9	40	0.00	0.00	0.41	0.00	0.04	0.00	0.06	0.02	0.00	0.00	0.01	0.68
9	41	0.00	0.00	0.26	0.00	0.03	0.00	0.06	0.06	0.00	0.00	0.01	0.72
9	42	0.00	0.01	0.44	0.00	0.02	0.00	0.05	0.00	0.01	0.00	0.01	0.75
9	43	0.00	0.02	0.45	0.00	0.01	0.00	0.05	0.01	0.01	0.00	0.01	0.85
9	44	0.01	0.02	0.41	0.00	0.00	0.00	0.05	0.05	0.01	0.00	0.01	0.90
9	45	0.01	0.00	0.37	0.00	0.00	0.00	0.04	0.15	0.01	0.01	0.01	0.90
9	46	0.00	0.00	0.35	0.00	0.00	0.00	0.04	0.14	0.01	0.00	0.00	0.89
9	47	0.00	0.00	0.32	0.00	0.00	0.00	0.03	0.15	0.01	0.00	0.01	0.89
9	48	0.00	0.00	0.30	0.00	0.00	0.00	0.04	0.18	0.01	0.00	0.01	0.89
9	49	0.00	0.00	0.24	0.00	0.01	0.00	0.04	0.23	0.01	0.00	0.01	0.91
9	50	0.00	0.00	0.37	0.00	0.01	0.00	0.04	0.09	0.01	0.00	0.01	0.94
9	51	0.00	0.00	0.36	0.00	0.01	0.00	0.05	0.15	0.01	0.00	0.01	0.97
9	52	0.00	0.00	0.47	0.00	0.01	0.00	0.05	0.10	0.01	0.00	0.01	1.07
9	53	0.00	0.00	0.52	0.00	0.01	0.00	0.05	0.01	0.00	0.00	0.01	1.22
9	54	0.02	0.00	0.66	0.00	0.00	0.00	0.05	0.00	0.00	0.00	0.01	1.45
9	55	0.00	0.02	0.69	0.00	0.00	0.00	0.06	0.03	0.00	0.10	0.00	1.58
9	56	0.00	0.00	0.92	0.00	0.00	0.00	0.06	0.06	0.01	0.22	0.00	1.65
9	57	0.00	0.00	1.00	0.00	0.00	0.00	0.07	0.05	0.01	0.12	0.00	1.67
9	58	0.00	0.02	1.02	0.00	0.00	0.00	0.08	0.00	0.00	0.18	0.00	1.64
9	59	0.00	0.01	1.02	0.00	0.01	0.00	0.08	0.04	0.00	0.10	0.00	1.58
9	60	0.01	0.00	1.10	0.00	0.02	0.00	0.09	0.00	0.01	0.09	0.00	1.54
9	61	0.00	0.00	1.13	0.00	0.03	0.00	0.11	0.00	0.00	0.00	0.01	1.49
9	62	0.00	0.00	1.16	0.00	0.04	0.00	0.12	0.04	0.01	0.00	0.01	1.42
9	63	0.00	0.00	1.12	0.00	0.06	0.00	0.13	0.03	0.01	0.01	0.01	1.33
9	64	0.00	0.00	1.16	0.00	0.08	0.00	0.14	0.04	0.01	0.02	0.01	1.23
9	65	0.03	0.00	1.10	0.00	0.10	0.00	0.15	0.03	0.01	0.00	0.00	1.11
9	66	0.01	0.00	0.96	0.00	0.12	0.00	0.15	0.00	0.01	0.00	0.01	1.00
9	67	0.00	0.00	1.04	0.00	0.15	0.00	0.16	0.03	0.01	0.00	0.01	0.88
9	68	0.03	0.01	1.02	0.00	0.17	0.00	0.19	0.01	0.01	0.00	0.01	0.85
9	69	0.02	0.00	1.03	0.00	0.20	0.00	0.21	0.00	0.01	0.00	0.01	0.78
9	70	0.04	0.00	1.13	0.00	0.22	0.00	0.23	0.04	0.01	0.00	0.00	0.70
9	71	0.05	0.00	1.18	0.00	0.23	0.00	0.26	0.04	0.01	0.00	0.01	0.57
9	72	0.13	0.03	1.32	0.01	0.24	0.00	0.26	0.04	0.00	0.00	0.01	0.63
9	73	0.10	0.00	1.42	0.00	0.24	0.00	0.27	0.04	0.01	0.00	0.01	0.61
9	74	0.15	0.02	1.43	0.02	0.25	0.00	0.26	0.00	0.01	0.00	0.01	0.63

9	75	0.18	0.00	1.34	0.01	0.24	0.00	0.28	0.00	0.01	0.00	0.01	0.61
9	76	0.20	0.00	1.25	0.03	0.25	0.00	0.30	0.00	0.01	0.00	0.01	0.54
9	77	0.21	0.03	1.07	0.03	0.25	0.00	0.30	0.00	0.01	0.00	0.01	0.43
9	78	0.06	0.00	0.85	0.02	0.25	0.00	0.29	0.02	0.01	0.00	0.01	0.32
9	79	0.10	0.00	0.58	0.01	0.25	0.00	0.23	0.04	0.01	0.01	0.00	0.25
9	80	0.00	0.02	0.48	0.00	0.24	0.00	0.18	0.04	0.00	0.00	0.01	0.20
9	81	0.00	0.03	0.28	0.00	0.24	0.00	0.13	0.04	0.00	0.00	0.02	0.17
9	82	0.00	0.04	0.33	0.00	0.24	0.00	0.10	0.04	0.01	0.00	0.01	0.15
9	83	0.00	0.00	0.21	0.00	0.25	0.00	0.06	0.00	0.01	0.00	0.00	0.14
9	84	0.00	0.00	0.29	0.00	0.25	0.00	0.04	0.04	0.01	0.00	0.04	0.11
9	85	0.00	0.05	0.29	0.00	0.25	0.00	0.04	0.04	0.01	0.00	0.04	0.15
9	86	0.00	0.00	0.32	0.00	0.25	0.00	0.04	0.04	0.01	0.00	0.07	0.14
9	87	0.00	0.00	0.34	0.00	0.25	0.00	0.04	0.04	0.01	0.00	0.09	0.14
9	88	0.01	0.04	0.40	0.00	0.24	0.00	0.04	0.04	0.00	0.00	0.10	0.18
9	89	0.00	0.05	0.41	0.00	0.23	0.00	0.03	0.01	0.00	0.00	0.10	0.19
9	90	0.00	0.00	0.43	0.00	0.23	0.00	0.03	0.00	0.01	0.00	0.09	0.21
9	91	0.01	0.03	0.41	0.00	0.22	0.00	0.03	0.00	0.00	0.00	0.10	0.22
9	92	0.00	0.02	0.42	0.00	0.21	0.00	0.02	0.00	0.00	0.00	0.10	0.20
9	93	0.02	0.03	0.45	0.00	0.21	0.00	0.02	0.02	0.01	0.00	0.10	0.22
9	94	0.00	0.02	0.42	0.00	0.21	0.00	0.03	0.04	0.01	0.00	0.10	0.24
9	95	0.00	0.03	0.43	0.00	0.22	0.00	0.03	0.04	0.02	0.00	0.06	0.23
9	96	0.00	0.03	0.46	0.00	0.23	0.00	0.02	0.04	0.03	0.00	0.10	0.26
9	97	0.01	0.02	0.53	0.00	0.23	0.00	0.02	0.04	0.03	0.01	0.03	0.28
9	98	0.00	0.00	0.57	0.00	0.23	0.00	0.02	0.00	0.05	0.00	0.06	0.32
9	99	0.00	0.00	0.71	0.00	0.22	0.00	0.02	0.03	0.05	0.00	0.03	0.36
9	100	0.00	0.00	0.85	0.00	0.20	0.00	0.00	0.00	0.04	0.00	0.00	0.41
9	101	0.00	0.00	1.00	0.00	0.19	0.00	0.02	0.04	0.03	0.00	0.00	0.46
9	102	0.00	0.00	1.08	0.00	0.17	0.00	0.02	0.00	0.02	0.00	0.00	0.48
9	103	0.00	0.02	1.30	0.00	0.15	0.00	0.02	0.03	0.02	0.00	0.00	0.47
9	104	0.00	0.00	1.25	0.00	0.13	0.00	0.02	0.00	0.01	0.00	0.00	0.46
9	105	0.00	0.00	1.31	0.00	0.11	0.00	0.01	0.00	0.01	0.00	0.01	0.39
9	106	0.00	0.00	1.41	0.00	0.08	0.00	0.02	0.03	0.01	0.00	0.01	0.31
9	107	0.00	0.00	1.27	0.00	0.05	0.00	0.02	0.01	0.01	0.00	0.01	0.29
9	108	0.00	0.00	1.39	0.00	0.04	0.00	0.02	0.03	0.00	0.00	0.01	0.26
9	109	0.02	0.00	1.41	0.00	0.02	0.00	0.02	0.00	0.00	0.00	0.01	0.24
9	110	0.00	0.00	1.51	0.00	0.01	0.00	0.02	0.04	0.01	0.00	0.01	0.24

9	111	0.03	0.00	1.66	0.04	0.00	0.00	0.02	0.04	0.01	0.00	0.01	0.23
9	112	0.00	0.00	1.75	0.12	0.00	0.00	0.02	0.04	0.01	0.00	0.01	0.21
9	113	0.00	0.01	1.85	0.21	0.00	0.00	0.02	0.04	0.01	0.00	0.01	0.21
9	114	0.00	0.00	1.89	0.25	0.00	0.00	0.00	0.04	0.01	0.01	0.01	0.21
9	115	0.00	0.00	1.91	0.23	0.00	0.00	0.02	0.03	0.01	0.00	0.01	0.21
9	116	0.00	0.00	1.96	0.21	0.00	0.00	0.02	0.00	0.01	0.00	0.01	0.25
9	117	0.00	0.00	1.95	0.19	0.00	0.00	0.02	0.05	0.01	0.00	0.01	0.27
9	118	0.00	0.00	1.92	0.17	0.00	0.00	0.02	0.07	0.00	0.00	0.01	0.30
9	119	0.00	0.01	1.90	0.15	0.00	0.00	0.01	0.11	0.00	0.00	0.00	0.31
9	120	0.00	0.00	1.71	0.12	0.00	0.00	0.02	0.14	0.01	0.00	0.00	0.34
9	121	0.00	0.00	1.72	0.11	0.01	0.00	0.02	0.24	0.00	0.00	0.01	0.33
9	122	0.00	0.00	1.57	0.09	0.01	0.00	0.02	0.25	0.01	0.00	0.01	0.33
9	123	0.00	0.00	1.42	0.07	0.02	0.00	0.02	0.24	0.01	0.01	0.01	0.31
9	124	0.00	0.01	1.39	0.09	0.03	0.00	0.02	0.31	0.00	0.00	0.01	0.28
9	125	0.00	0.00	1.30	0.12	0.05	0.00	0.02	0.31	0.00	0.00	0.01	0.28
9	126	0.00	0.00	1.07	0.10	0.06	0.00	0.01	0.28	0.01	0.00	0.01	0.28
9	127	0.00	0.00	0.86	0.08	0.09	0.00	0.02	0.24	0.01	0.00	0.00	0.29
9	128	0.00	0.00	0.64	0.07	0.11	0.00	0.02	0.19	0.00	0.00	0.01	0.29
9	129	0.01	0.02	0.56	0.07	0.13	0.00	0.01	0.19	0.00	0.00	0.01	0.28
9	130	0.00	0.00	0.47	0.10	0.15	0.00	0.02	0.15	0.01	0.00	0.01	0.30
9	131	0.00	0.00	0.30	0.12	0.17	0.00	0.02	0.09	0.01	0.05	0.01	0.30
9	132	0.03	0.00	0.18	0.14	0.19	0.00	0.02	0.09	0.01	0.04	0.00	0.33
9	133	0.09	0.00	0.24	0.13	0.21	0.00	0.02	0.07	0.01	0.06	0.01	0.36
9	134	0.04	0.00	0.07	0.15	0.23	0.00	0.02	0.01	0.01	0.09	0.00	0.37
9	135	0.06	0.00	0.10	0.17	0.24	0.00	0.02	0.00	0.03	0.00	0.01	0.38
9	136	0.11	0.00	0.13	0.17	0.26	0.00	0.02	0.04	0.05	0.10	0.01	0.39
9	137	0.13	0.01	0.07	0.18	0.25	0.00	0.02	0.02	0.08	0.06	0.00	0.37
9	138	0.12	0.02	0.13	0.20	0.22	0.00	0.02	0.02	0.13	0.07	0.01	0.39
9	139	0.22	0.00	0.12	0.22	0.20	0.00	0.02	0.04	0.18	0.24	0.05	0.39
9	140	0.17	0.00	0.14	0.25	0.17	0.00	0.02	0.02	0.22	0.28	0.10	0.38
9	141	0.18	0.03	0.14	0.29	0.14	0.00	0.02	0.00	0.32	0.42	0.15	0.38
9	142	0.24	0.00	0.13	0.32	0.11	0.00	0.02	0.00	0.40	0.43	0.26	0.36
9	143	0.40	0.00	0.16	0.33	0.08	0.00	0.03	0.00	0.48	0.59	0.39	0.34
9	144	0.48	0.00	0.15	0.36	0.06	0.00	0.04	0.00	0.57	0.51	0.50	0.31
9	145	0.67	0.02	0.18	0.35	0.04	0.00	0.05	0.00	0.66	0.76	0.63	0.28
9	146	0.73	0.00	0.13	0.39	0.03	0.00	0.05	0.02	0.75	0.84	0.77	0.25

9	147	0.82	0.02	0.09	0.43	0.01	0.00	0.07	0.00	0.79	0.93	0.95	0.21
9	148	0.76	0.00	0.17	0.50	0.01	0.00	0.08	0.02	0.76	1.06	1.10	0.18
9	149	0.85	0.12	0.17	0.56	0.00	0.00	0.10	0.01	0.73	1.26	1.22	0.16
9	150	0.91	0.00	0.13	0.62	0.00	0.00	0.11	0.04	0.68	1.35	1.34	0.13
9	151	0.95	0.00	0.13	0.70	0.00	0.00	0.14	0.00	0.63	1.38	1.44	0.10
9	152	0.94	0.00	0.18	0.74	0.00	0.00	0.16	0.03	0.58	1.48	1.55	0.08
9	153	0.96	0.22	0.19	0.81	0.00	0.02	0.18	0.04	0.51	1.52	1.64	0.05
9	154	0.98	0.41	0.19	0.86	0.00	0.03	0.20	0.04	0.44	1.50	1.70	0.03
9	155	0.98	0.15	0.10	0.90	0.00	0.05	0.22	0.04	0.39	1.55	1.63	0.02
9	156	1.02	0.13	0.13	0.90	0.00	0.07	0.24	0.04	0.35	1.38	1.56	0.00
9	157	0.91	0.48	0.06	0.90	0.00	0.09	0.26	0.04	0.30	1.24	1.57	0.00
9	158	1.02	0.46	0.03	0.92	0.00	0.11	0.28	0.04	0.24	1.37	1.51	0.00
9	159	1.00	0.46	0.15	0.93	0.00	0.12	0.30	0.03	0.19	1.16	1.43	0.00
9	160	0.94	0.45	0.17	0.00	0.00	0.00	0.00	0.00	0.00	1.16	0.00	0.00
9	161	0.98	0.42	0.13	0.00	0.00	0.00	0.00	0.00	0.00	1.30	0.00	0.00
10	0	0.01	0.00	0.00	0.00	0.00	0.47	0.02	0.05	0.01	0.00	0.01	0.00
10	1	0.01	0.00	0.00	0.00	0.00	0.47	0.02	0.05	0.01	0.00	0.01	0.00
10	2	0.02	0.00	0.08	0.00	0.00	0.49	0.02	0.08	0.01	0.00	0.01	0.00
10	3	0.01	0.00	0.00	0.00	0.00	0.50	0.02	0.09	0.01	0.00	0.01	0.00
10	4	0.00	0.00	0.07	0.00	0.00	0.52	0.02	0.11	0.02	0.00	0.01	0.00
10	5	0.02	0.00	0.08	0.00	0.00	0.53	0.02	0.13	0.03	0.00	0.01	0.00
10	6	0.00	0.00	0.09	0.00	0.00	0.53	0.02	0.01	0.04	0.00	0.01	0.00
10	7	0.00	0.00	0.08	0.00	0.00	0.53	0.02	0.05	0.05	0.00	0.01	0.00
10	8	0.00	0.00	0.08	0.00	0.00	0.54	0.02	0.03	0.07	0.00	0.01	0.00
10	9	0.00	0.00	0.08	0.00	0.00	0.54	0.02	0.02	0.09	0.00	0.01	0.00
10	10	0.00	0.02	0.08	0.00	0.00	0.52	0.02	0.04	0.11	0.00	0.00	0.01
10	11	0.00	0.00	0.06	0.00	0.00	0.51	0.02	0.00	0.14	0.00	0.01	0.01
10	12	0.00	0.00	0.07	0.00	0.00	0.49	0.02	0.05	0.17	0.00	0.00	0.01
10	13	0.00	0.02	0.02	0.00	0.00	0.47	0.02	0.05	0.21	0.00	0.01	0.01
10	14	0.02	0.00	0.08	0.00	0.00	0.45	0.02	0.00	0.24	0.00	0.00	0.01
10	15	0.00	0.01	0.09	0.00	0.00	0.41	0.02	0.00	0.24	0.00	0.01	0.01
10	16	0.02	0.03	0.00	0.00	0.00	0.36	0.02	0.01	0.27	0.00	0.01	0.00
10	17	0.00	0.01	0.07	0.00	0.00	0.30	0.02	0.00	0.27	0.00	0.00	0.00
10	18	0.00	0.02	0.05	0.00	0.00	0.26	0.02	0.00	0.25	0.00	0.00	0.00
10	19	0.00	0.00	0.07	0.00	0.00	0.21	0.02	0.00	0.24	0.00	0.00	0.00
10	20	0.01	0.00	0.09	0.00	0.00	0.19	0.02	0.00	0.21	0.00	0.01	0.00

10	21	0.00	0.00	0.02	0.00	0.00	0.20	0.02	0.00	0.17	0.00	0.00	0.00
10	22	0.01	0.00	0.02	0.00	0.00	0.20	0.01	0.00	0.17	0.00	0.01	0.00
10	23	0.02	0.00	0.00	0.01	0.00	0.18	0.02	0.02	0.16	0.00	0.00	0.00
10	24	0.00	0.00	0.00	0.03	0.00	0.16	0.02	0.02	0.16	0.01	0.00	0.02
10	25	0.03	0.03	0.08	0.08	0.00	0.14	0.02	0.00	0.15	0.00	0.00	0.06
10	26	0.03	0.00	0.11	0.06	0.00	0.11	0.02	0.02	0.13	0.00	0.03	0.07
10	27	0.00	0.00	0.09	0.00	0.00	0.09	0.02	0.00	0.10	0.00	0.01	0.05
10	28	0.00	0.00	0.05	0.00	0.00	0.07	0.02	0.00	0.09	0.00	0.01	0.04
10	29	0.02	0.02	0.10	0.00	0.00	0.05	0.02	0.00	0.10	0.00	0.00	0.04
10	30	0.01	0.00	0.02	0.00	0.00	0.03	0.02	0.03	0.10	0.00	0.00	0.04
10	31	0.00	0.00	0.08	0.00	0.00	0.01	0.02	0.00	0.11	0.00	0.00	0.03
10	32	0.00	0.00	0.00	0.00	0.01	0.00	0.02	0.00	0.12	0.00	0.01	0.03
10	33	0.00	0.00	0.00	0.00	0.02	0.00	0.02	0.03	0.12	0.04	0.00	0.05
10	34	0.00	0.00	0.09	0.00	0.03	0.00	0.02	0.00	0.13	0.00	0.01	0.06
10	35	0.00	0.00	0.02	0.00	0.04	0.00	0.02	0.00	0.13	0.08	0.01	0.06
10	36	0.00	0.00	0.03	0.00	0.05	0.00	0.01	0.04	0.14	0.09	0.01	0.10
10	37	0.00	0.00	0.24	0.00	0.06	0.00	0.02	0.00	0.14	0.13	0.01	0.14
10	38	0.00	0.00	0.27	0.00	0.08	0.00	0.02	0.00	0.15	0.16	0.01	0.17
10	39	0.01	0.00	0.20	0.00	0.08	0.00	0.02	0.01	0.18	0.15	0.01	0.18
10	40	0.00	0.00	0.27	0.00	0.09	0.00	0.02	0.00	0.19	0.18	0.01	0.19
10	41	0.00	0.00	0.26	0.00	0.10	0.00	0.02	0.03	0.24	0.14	0.01	0.19
10	42	0.02	0.03	0.22	0.00	0.11	0.00	0.02	0.00	0.27	0.19	0.01	0.19
10	43	0.00	0.00	0.17	0.00	0.12	0.00	0.02	0.01	0.29	0.18	0.00	0.19
10	44	0.00	0.02	0.23	0.00	0.13	0.00	0.02	0.00	0.31	0.17	0.00	0.18
10	45	0.00	0.02	0.13	0.00	0.14	0.00	0.04	0.03	0.33	0.13	0.00	0.14
10	46	0.00	0.02	0.00	0.00	0.16	0.00	0.07	0.00	0.34	0.00	0.00	0.10
10	47	0.00	0.00	0.10	0.00	0.17	0.00	0.10	0.00	0.35	0.07	0.00	0.07
10	48	0.00	0.00	0.00	0.00	0.18	0.00	0.13	0.00	0.36	0.03	0.00	0.05
10	49	0.00	0.00	0.00	0.00	0.19	0.00	0.18	0.00	0.38	0.03	0.01	0.03
10	50	0.00	0.00	0.00	0.00	0.19	0.00	0.22	0.00	0.42	0.00	0.00	0.02
10	51	0.00	0.02	0.08	0.00	0.19	0.00	0.27	0.02	0.44	0.00	0.00	0.01
10	52	0.00	0.04	0.00	0.00	0.20	0.00	0.32	0.07	0.46	0.00	0.03	0.00
10	53	0.00	0.00	0.05	0.00	0.19	0.00	0.37	0.07	0.48	0.00	0.03	0.00
10	54	0.03	0.00	0.09	0.00	0.19	0.00	0.41	0.08	0.49	0.00	0.02	0.00
10	55	0.00	0.00	0.09	0.00	0.18	0.00	0.45	0.10	0.49	0.00	0.03	0.00
10	56	0.00	0.03	0.09	0.00	0.17	0.00	0.49	0.03	0.49	0.00	0.00	0.00

10	57	0.00	0.09	0.08	0.00	0.16	0.00	0.51	0.03	0.47	0.00	0.02	0.00
10	58	0.00	0.00	0.08	0.00	0.15	0.00	0.46	0.00	0.48	0.00	0.00	0.00
10	59	0.00	0.12	0.08	0.00	0.13	0.00	0.33	0.04	0.49	0.00	0.08	0.00
10	60	0.00	0.15	0.08	0.00	0.14	0.00	0.22	0.00	0.50	0.00	0.11	0.00
10	61	0.00	0.21	0.07	0.00	0.16	0.00	0.12	0.04	0.54	0.00	0.15	0.00
10	62	0.02	0.26	0.08	0.00	0.18	0.00	0.07	0.06	0.57	0.01	0.19	0.00
10	63	0.00	0.19	0.06	0.00	0.20	0.00	0.03	0.06	0.54	0.00	0.16	0.01
10	64	0.00	0.26	0.09	0.00	0.22	0.00	0.02	0.03	0.49	0.00	0.13	0.02
10	65	0.00	0.25	0.00	0.00	0.24	0.00	0.02	0.08	0.44	0.02	0.09	0.03
10	66	0.00	0.22	0.00	0.00	0.26	0.00	0.01	0.17	0.39	0.04	0.10	0.04
10	67	0.03	0.12	0.04	0.00	0.28	0.00	0.02	0.24	0.34	0.01	0.11	0.04
10	68	0.01	0.24	0.06	0.00	0.30	0.00	0.02	0.30	0.29	0.04	0.08	0.06
10	69	0.00	0.23	0.06	0.00	0.31	0.00	0.01	0.36	0.24	0.06	0.06	0.07
10	70	0.00	0.20	0.09	0.00	0.32	0.00	0.01	0.41	0.18	0.07	0.03	0.07
10	71	0.00	0.22	0.08	0.00	0.35	0.00	0.02	0.47	0.16	0.01	0.01	0.08
10	72	0.00	0.18	0.08	0.00	0.38	0.00	0.02	0.55	0.16	0.02	0.00	0.10
10	73	0.01	0.03	0.07	0.00	0.40	0.00	0.02	0.59	0.15	0.10	0.00	0.10
10	74	0.00	0.14	0.08	0.00	0.43	0.00	0.02	0.60	0.13	0.06	0.00	0.09
10	75	0.00	0.21	0.09	0.00	0.45	0.00	0.02	0.58	0.12	0.07	0.00	0.08
10	76	0.00	0.27	0.06	0.00	0.47	0.00	0.02	0.60	0.11	0.00	0.03	0.08
10	77	0.00	0.16	0.08	0.00	0.49	0.00	0.01	0.60	0.09	0.04	0.02	0.08
10	78	0.00	0.32	0.09	0.00	0.50	0.00	0.02	0.53	0.08	0.07	0.05	0.08
10	79	0.00	0.36	0.08	0.00	0.50	0.00	0.02	0.56	0.07	0.08	0.07	0.08
10	80	0.00	0.38	0.09	0.00	0.50	0.00	0.02	0.52	0.05	0.05	0.05	0.08
10	81	0.02	0.42	0.08	0.04	0.53	0.00	0.02	0.57	0.04	0.02	0.08	0.09
10	82	0.00	0.38	0.07	0.05	0.57	0.00	0.02	0.67	0.04	0.02	0.07	0.12
10	83	0.00	0.51	0.08	0.08	0.61	0.00	0.02	0.72	0.03	0.14	0.01	0.14
10	84	0.00	0.46	0.07	0.18	0.64	0.00	0.01	0.72	0.01	0.09	0.03	0.14
10	85	0.00	0.68	0.08	0.21	0.66	0.00	0.02	0.79	0.01	0.14	0.07	0.14
10	86	0.00	0.72	0.00	0.25	0.68	0.00	0.02	0.73	0.01	0.10	0.08	0.14
10	87	0.17	0.78	0.04	0.25	0.68	0.00	0.02	0.67	0.01	0.07	0.00	0.13
10	88	0.21	0.85	0.01	0.26	0.69	0.00	0.02	0.59	0.01	0.03	0.01	0.12
10	89	0.22	0.70	0.02	0.25	0.68	0.00	0.02	0.51	0.01	0.07	0.05	0.13
10	90	0.00	0.71	0.08	0.26	0.68	0.00	0.02	0.43	0.01	0.16	0.02	0.16
10	91	0.22	0.62	0.00	0.27	0.68	0.00	0.02	0.38	0.02	0.19	0.07	0.20
10	92	0.16	0.48	0.05	0.25	0.68	0.00	0.01	0.38	0.03	0.24	0.03	0.25

10	93	0.29	0.43	0.35	0.26	0.67	0.00	0.02	0.32	0.03	0.29	0.03	0.30
10	94	0.07	0.32	0.46	0.26	0.65	0.00	0.02	0.31	0.04	0.31	0.00	0.36
10	95	0.41	0.34	0.56	0.38	0.63	0.00	0.02	0.27	0.04	0.39	0.00	0.40
10	96	0.45	0.40	0.52	0.45	0.61	0.00	0.02	0.21	0.05	0.43	0.00	0.45
10	97	0.09	0.29	0.46	0.42	0.58	0.00	0.02	0.19	0.07	0.45	0.01	0.48
10	98	0.34	0.16	0.35	0.34	0.56	0.00	0.02	0.22	0.08	0.49	0.01	0.52
10	99	0.39	0.19	0.32	0.34	0.52	0.00	0.02	0.22	0.08	0.45	0.00	0.53
10	100	0.41	0.25	0.28	0.36	0.49	0.00	0.02	0.22	0.08	0.41	0.01	0.52
10	101	0.22	0.23	0.21	0.35	0.45	0.00	0.02	0.21	0.08	0.50	0.00	0.51
10	102	0.38	0.19	0.18	0.34	0.43	0.00	0.02	0.21	0.08	0.48	0.02	0.49
10	103	0.26	0.04	0.05	0.34	0.39	0.00	0.02	0.26	0.08	0.42	0.00	0.49
10	104	0.44	0.06	0.09	0.38	0.36	0.00	0.02	0.31	0.08	0.47	0.00	0.48
10	105	0.42	0.00	0.16	0.42	0.33	0.00	0.02	0.35	0.08	0.44	0.00	0.45
10	106	0.56	0.05	0.03	0.54	0.29	0.00	0.02	0.40	0.09	0.43	0.01	0.43
10	107	0.75	0.20	0.02	0.72	0.25	0.00	0.02	0.44	0.09	0.40	0.00	0.42
10	108	0.92	0.26	0.05	0.91	0.21	0.00	0.02	0.48	0.11	0.38	0.02	0.41
10	109	0.90	0.30	0.18	0.85	0.17	0.00	0.02	0.52	0.10	0.33	0.00	0.39
10	110	0.68	0.07	0.00	0.60	0.13	0.00	0.02	0.56	0.10	0.35	0.00	0.35
10	111	0.52	0.09	0.00	0.40	0.10	0.00	0.02	0.57	0.09	0.30	0.00	0.31
10	112	0.30	0.00	0.00	0.31	0.07	0.00	0.02	0.61	0.09	0.21	0.00	0.23
10	113	0.32	0.00	0.04	0.21	0.05	0.00	0.02	0.60	0.12	0.09	0.00	0.16
10	114	0.18	0.02	0.23	0.17	0.03	0.00	0.01	0.67	0.17	0.08	0.00	0.14
10	115	0.02	0.10	0.13	0.13	0.02	0.00	0.02	0.76	0.23	0.11	0.00	0.12
10	116	0.29	0.00	0.12	0.19	0.01	0.00	0.02	0.80	0.28	0.11	0.00	0.11
10	117	0.21	0.02	0.13	0.20	0.00	0.00	0.02	0.82	0.35	0.09	0.01	0.10
10	118	0.27	0.17	0.09	0.22	0.00	0.00	0.02	0.78	0.42	0.08	0.04	0.09
10	119	0.38	0.25	0.20	0.24	0.00	0.00	0.02	0.66	0.50	0.02	0.08	0.02
10	120	0.25	0.22	0.16	0.26	0.00	0.00	0.02	0.56	0.58	0.04	0.10	0.05
10	121	0.46	0.32	0.21	0.32	0.00	0.00	0.02	0.49	0.62	0.03	0.14	0.03
10	122	0.42	0.44	0.37	0.42	0.00	0.00	0.03	0.47	0.65	0.02	0.19	0.02
10	123	0.42	0.37	0.35	0.44	0.00	0.00	0.03	0.44	0.67	0.01	0.24	0.02
10	124	0.55	0.36	0.30	0.46	0.00	0.00	0.03	0.45	0.67	0.00	0.25	0.01
10	125	0.53	0.56	0.21	0.49	0.00	0.00	0.02	0.42	0.67	0.00	0.32	0.01
10	126	0.49	0.67	0.20	0.52	0.00	0.00	0.04	0.36	0.66	0.00	0.34	0.01
10	127	0.45	0.68	0.15	0.55	0.00	0.00	0.04	0.34	0.66	0.00	0.34	0.00
10	128	0.06	0.70	0.24	0.54	0.00	0.00	0.04	0.33	0.67	0.00	0.39	0.00

10	129	0.23	0.73	0.27	0.56	0.00	0.00	0.04	0.27	0.68	0.00	0.34	0.00
10	130	0.33	0.55	0.26	0.57	0.00	0.00	0.03	0.28	0.69	0.00	0.37	0.00
10	131	0.32	0.66	0.17	0.58	0.00	0.00	0.04	0.25	0.69	0.00	0.49	0.00
10	132	0.20	0.68	0.10	0.59	0.00	0.00	0.04	0.21	0.69	0.00	0.48	0.00
10	133	0.28	0.68	0.15	0.57	0.00	0.00	0.04	0.18	0.68	0.00	0.45	0.00
10	134	0.22	0.85	0.19	0.57	0.00	0.00	0.04	0.18	0.67	0.00	0.55	0.00
10	135	0.20	0.89	0.01	0.55	0.00	0.00	0.04	0.15	0.65	0.00	0.60	0.00
10	136	0.00	0.69	0.02	0.54	0.00	0.00	0.04	0.12	0.63	0.00	0.62	0.00
10	137	0.06	0.62	0.09	0.55	0.00	0.00	0.12	0.14	0.61	0.00	0.64	0.00
10	138	0.34	0.85	0.04	0.57	0.00	0.00	0.23	0.23	0.56	0.00	0.63	0.00
10	139	0.39	0.63	0.15	0.60	0.00	0.00	0.32	0.20	0.58	0.00	0.68	0.00
10	140	0.33	0.69	0.15	0.63	0.00	0.00	0.43	0.15	0.57	0.00	0.66	0.00
10	141	0.31	0.81	0.15	0.66	0.00	0.00	0.58	0.07	0.54	0.00	0.67	0.00
10	142	0.32	0.78	0.13	0.68	0.00	0.00	0.72	0.08	0.51	0.00	0.62	0.00
10	143	0.32	0.80	0.14	0.68	0.00	0.00	0.85	0.01	0.48	0.00	0.68	0.00
10	144	0.30	0.75	0.14	0.68	0.00	0.00	0.99	0.00	0.45	0.00	0.65	0.00
10	145	0.30	0.70	0.10	0.68	0.00	0.00	1.15	0.04	0.41	0.00	0.60	0.00
10	146	0.05	0.63	0.11	0.62	0.00	0.00	1.38	0.04	0.37	0.00	0.46	0.00
10	147	0.17	0.64	0.00	0.53	0.00	0.00	1.64	0.03	0.33	0.00	0.45	0.00
10	148	0.09	0.53	0.09	0.47	0.00	0.00	1.92	0.00	0.29	0.00	0.34	0.00
10	149	0.05	0.56	0.03	0.39	0.00	0.00	2.17	0.00	0.27	0.00	0.23	0.00
10	150	0.13	0.53	0.00	0.37	0.00	0.00	2.36	0.04	0.28	0.00	0.30	0.00
10	151	0.19	0.53	0.09	0.32	0.00	0.00	2.53	0.00	0.30	0.00	0.28	0.00
10	152	0.12	0.50	0.08	0.29	0.00	0.00	2.69	0.04	0.32	0.00	0.32	0.00
10	153	0.21	0.57	0.00	0.28	0.00	0.00	2.82	0.04	0.33	0.00	0.28	0.00
10	154	0.22	0.55	0.08	0.28	0.00	0.00	2.92	0.00	0.35	0.00	0.28	0.00
10	155	0.27	0.49	0.01	0.26	0.00	0.00	3.00	0.04	0.36	0.00	0.27	0.00
10	156	0.34	0.61	0.06	0.26	0.00	0.00	3.04	0.04	0.36	0.00	0.24	0.00
10	157	0.34	0.26	0.08	0.23	0.00	0.00	3.03	0.04	0.37	0.00	0.21	0.00
10	158	0.39	0.40	0.01	0.19	0.00	0.00	3.04	0.04	0.37	0.00	0.13	0.00
10	159	0.29	0.55	0.09	0.23	0.00	0.00	2.99	0.04	0.36	0.00	0.14	0.00
10	160	0.36	0.43	0.00	0.20	0.00	0.00	2.91	0.04	0.37	0.00	0.13	0.00
10	161	0.38	0.25	0.09	0.21	0.00	0.00	2.80	0.04	0.39	0.00	0.13	0.00
10	162	0.38	0.16	0.07	0.20	0.00	0.00	2.66	0.00	0.41	0.00	0.14	0.00
10	163	0.34	0.29	0.07	0.19	0.00	0.00	2.49	0.00	0.43	0.00	0.14	0.00
10	164	0.25	0.02	0.09	0.16	0.00	0.00	2.31	0.01	0.45	0.00	0.10	0.00

10	165	0.31	0.28	0.00	0.15	0.00	0.00	2.12	0.00	0.46	0.00	0.14	0.00
10	166	0.27	0.13	0.07	0.12	0.00	0.00	1.92	0.04	0.47	0.00	0.09	0.00
10	167	0.17	0.16	0.08	0.12	0.00	0.00	1.75	0.04	0.47	0.00	0.14	0.00
10	168	0.14	0.18	0.06	0.11	0.00	0.00	1.62	0.01	0.48	0.00	0.10	0.00
10	169	0.03	0.23	0.06	0.10	0.00	0.00	1.46	0.01	0.48	0.00	0.18	0.00
10	170	0.09	0.21	0.08	0.11	0.00	0.00	1.30	0.00	0.48	0.00	0.20	0.00
10	171	0.00	0.22	0.00	0.09	0.00	0.00	1.12	0.00	0.48	0.00	0.21	0.00
10	172	0.00	0.05	0.00	0.08	0.00	0.00	0.98	0.00	0.47	0.00	0.20	0.00
10	173	0.00	0.22	0.00	0.09	0.00	0.00	0.90	0.00	0.46	0.00	0.21	0.00
10	174	0.04	0.00	0.08	0.12	0.00	0.00	0.80	0.00	0.45	0.00	0.18	0.00
10	175	0.00	0.10	0.09	0.12	0.00	0.00	0.71	0.00	0.44	0.00	0.24	0.00
10	176	0.00	0.03	0.08	0.12	0.00	0.00	0.61	0.01	0.43	0.00	0.27	0.00
10	177	0.02	0.08	0.04	0.11	0.00	0.00	0.51	0.01	0.41	0.00	0.27	0.00
10	178	0.00	0.14	0.09	0.12	0.00	0.00	0.51	0.04	0.41	0.00	0.28	0.00
10	179	0.00	0.12	0.08	0.00	0.00	0.00	0.00	0.00	0.00	0.00	0.00	0.00
11	1	0.00	0.00	0.53	0.12	0.00	0.02	0.02	0.00	0.01	0.00	0.01	0.79
11	2	0.00	0.00	0.60	0.18	0.00	0.04	0.02	0.02	0.01	0.00	0.01	0.76
11	3	0.00	0.00	0.69	0.22	0.00	0.06	0.02	0.02	0.01	0.00	0.01	0.79
11	4	0.00	0.00	0.71	0.24	0.00	0.10	0.02	0.01	0.01	0.01	0.01	0.80
11	5	0.00	0.00	0.75	0.29	0.00	0.13	0.02	0.00	0.01	0.00	0.01	0.78
11	6	0.00	0.00	0.76	0.32	0.00	0.19	0.03	0.04	0.01	0.00	0.01	0.71
11	7	0.05	0.00	0.76	0.34	0.00	0.25	0.03	0.02	0.01	0.01	0.01	0.63
11	8	0.00	0.00	0.71	0.32	0.00	0.32	0.04	0.00	0.01	0.00	0.01	0.59
11	9	0.00	0.00	0.71	0.23	0.00	0.39	0.04	0.00	0.01	0.01	0.01	0.55
11	10	0.00	0.00	0.66	0.22	0.00	0.45	0.04	0.03	0.00	0.01	0.01	0.48
11	11	0.00	0.00	0.54	0.18	0.00	0.42	0.04	0.04	0.00	0.00	0.01	0.36
11	12	0.00	0.00	0.45	0.12	0.00	0.39	0.04	0.03	0.00	0.00	0.01	0.26
11	13	0.00	0.01	0.34	0.07	0.00	0.33	0.04	0.04	0.01	0.00	0.01	0.17
11	14	0.00	0.02	0.26	0.06	0.00	0.32	0.04	0.00	0.01	0.00	0.01	0.09
11	15	0.00	0.03	0.13	0.04	0.00	0.28	0.04	0.04	0.01	0.00	0.00	0.03
11	16	0.01	0.02	0.00	0.03	0.00	0.25	0.04	0.00	0.00	0.00	0.01	0.02
11	17	0.00	0.01	0.00	0.04	0.00	0.21	0.04	0.03	0.01	0.00	0.01	0.00
11	18	0.02	0.00	0.00	0.02	0.00	0.19	0.03	0.00	0.01	0.00	0.00	0.00
11	19	0.00	0.00	0.00	0.00	0.00	0.15	0.02	0.04	0.01	0.00	0.01	0.00
11	20	0.02	0.00	0.00	0.00	0.00	0.13	0.02	0.00	0.00	0.00	0.01	0.00
11	21	0.00	0.00	0.00	0.00	0.00	0.10	0.02	0.00	0.01	0.00	0.01	0.00

11	22	0.01	0.02	0.00	0.00	0.00	0.08	0.02	0.03	0.01	0.01	0.01	0.00
11	23	0.00	0.00	0.01	0.00	0.00	0.06	0.02	0.04	0.00	0.00	0.00	0.00
11	24	0.00	0.02	0.08	0.00	0.00	0.04	0.01	0.00	0.01	0.00	0.01	0.00
11	25	0.00	0.00	0.07	0.00	0.00	0.03	0.02	0.04	0.00	0.00	0.01	0.00
11	26	0.00	0.00	0.00	0.00	0.00	0.02	0.02	0.04	0.00	0.00	0.01	0.00
11	27	0.00	0.00	0.09	0.00	0.00	0.01	0.02	0.04	0.00	0.00	0.01	0.00
11	28	0.00	0.02	0.04	0.00	0.00	0.00	0.02	0.04	0.01	0.01	0.01	0.00
11	29	0.00	0.00	0.01	0.00	0.00	0.00	0.02	0.03	0.01	0.00	0.01	0.00
11	30	0.00	0.00	0.01	0.00	0.00	0.00	0.02	0.04	0.00	0.00	0.01	0.00
11	31	0.00	0.00	0.00	0.00	0.00	0.00	0.02	0.00	0.00	0.00	0.01	0.00
11	32	0.03	0.00	0.08	0.00	0.00	0.00	0.02	0.00	0.00	0.00	0.00	0.00
11	33	0.00	0.00	0.00	0.00	0.00	0.00	0.02	0.03	0.01	0.00	0.01	0.00
11	34	0.00	0.00	0.09	0.00	0.00	0.00	0.02	0.01	0.01	0.00	0.01	0.00
11	35	0.00	0.00	0.01	0.00	0.00	0.00	0.02	0.04	0.01	0.00	0.01	0.00
11	36	0.00	0.00	0.00	0.00	0.00	0.00	0.02	0.04	0.00	0.00	0.01	0.00
11	37	0.00	0.00	0.00	0.00	0.00	0.00	0.02	0.00	0.01	0.00	0.01	0.00
11	38	0.00	0.01	0.00	0.00	0.00	0.00	0.02	0.00	0.01	0.00	0.00	0.00
11	39	0.00	0.00	0.02	0.00	0.00	0.00	0.02	0.00	0.00	0.00	0.01	0.00
11	40	0.00	0.00	0.00	0.00	0.00	0.00	0.02	0.00	0.01	0.00	0.01	0.00
11	41	0.00	0.00	0.08	0.00	0.00	0.00	0.01	0.00	0.01	0.00	0.01	0.00
11	42	0.02	0.00	0.07	0.00	0.00	0.00	0.02	0.00	0.00	0.00	0.01	0.00
11	43	0.00	0.00	0.08	0.00	0.00	0.00	0.02	0.04	0.00	0.00	0.01	0.00
11	44	0.00	0.00	0.01	0.00	0.00	0.00	0.02	0.00	0.00	0.00	0.00	0.00
11	45	0.00	0.00	0.09	0.00	0.00	0.00	0.02	0.04	0.01	0.01	0.01	0.00
11	46	0.00	0.00	0.07	0.00	0.00	0.00	0.02	0.04	0.00	0.00	0.01	0.00
11	47	0.00	0.01	0.08	0.00	0.00	0.00	0.01	0.04	0.00	0.00	0.01	0.00
11	48	0.00	0.00	0.08	0.00	0.00	0.00	0.01	0.03	0.01	0.00	0.01	0.00
11	49	0.00	0.00	0.09	0.00	0.01	0.00	0.02	0.00	0.00	0.00	0.00	0.00
11	50	0.00	0.00	0.08	0.00	0.03	0.00	0.02	0.00	0.00	0.00	0.01	0.00
11	51	0.00	0.00	0.06	0.00	0.01	0.00	0.01	0.03	0.01	0.00	0.01	0.00
11	52	0.00	0.00	0.03	0.00	0.05	0.00	0.02	0.04	0.01	0.00	0.01	0.00
11	53	0.00	0.00	0.09	0.01	0.08	0.00	0.02	0.03	0.00	0.00	0.01	0.00
11	54	0.00	0.00	0.08	0.00	0.10	0.00	0.02	0.02	0.00	0.00	0.01	0.00
11	55	0.00	0.00	0.09	0.02	0.13	0.00	0.01	0.00	0.00	0.00	0.00	0.00
11	56	0.00	0.00	0.08	0.03	0.17	0.00	0.02	0.00	0.00	0.00	0.01	0.00
11	57	0.00	0.00	0.09	0.04	0.22	0.00	0.02	0.01	0.01	0.01	0.01	0.00

11	58	0.01	0.00	0.04	0.00	0.26	0.00	0.02	0.00	0.01	0.00	0.01	0.00
11	59	0.00	0.00	0.05	0.03	0.31	0.00	0.02	0.00	0.01	0.00	0.01	0.00
11	60	0.00	0.00	0.00	0.06	0.36	0.00	0.03	0.00	0.01	0.00	0.01	0.00
11	61	0.01	0.00	0.09	0.07	0.41	0.00	0.03	0.00	0.01	0.00	0.01	0.00
11	62	0.00	0.01	0.08	0.11	0.45	0.00	0.05	0.00	0.01	0.02	0.01	0.00
11	63	0.01	0.00	0.09	0.12	0.50	0.00	0.06	0.00	0.01	0.00	0.01	0.00
11	64	0.01	0.00	0.07	0.14	0.54	0.00	0.07	0.00	0.01	0.00	0.01	0.02
11	65	0.02	0.00	0.00	0.13	0.58	0.00	0.16	0.04	0.01	0.00	0.01	0.03
11	66	0.00	0.00	0.00	0.24	0.59	0.00	0.15	0.00	0.02	0.00	0.01	0.03
11	67	0.02	0.00	0.00	0.38	0.60	0.00	0.16	0.00	0.03	0.01	0.00	0.02
11	68	0.09	0.04	0.00	0.54	0.61	0.00	0.16	0.04	0.05	0.00	0.00	0.02
11	69	0.05	0.06	0.05	0.69	0.61	0.00	0.15	0.00	0.08	0.00	0.02	0.01
11	70	0.19	0.01	0.07	0.66	0.61	0.00	0.16	0.00	0.10	0.00	0.06	0.01
11	71	0.00	0.09	0.07	0.63	0.60	0.00	0.15	0.03	0.13	0.00	0.09	0.00
11	72	0.17	0.16	0.03	0.61	0.59	0.00	0.15	0.00	0.16	0.03	0.13	0.01
11	73	0.00	0.07	0.09	0.64	0.57	0.00	0.14	0.02	0.15	0.00	0.15	0.01
11	74	0.20	0.00	0.10	0.67	0.54	0.00	0.14	0.00	0.14	0.09	0.17	0.02
11	75	0.30	0.20	0.10	0.68	0.51	0.00	0.13	0.00	0.14	0.00	0.19	0.03
11	76	0.32	0.22	0.12	0.68	0.47	0.00	0.12	0.02	0.14	0.00	0.16	0.04
11	77	0.34	0.00	0.15	0.67	0.44	0.00	0.11	0.04	0.15	0.02	0.24	0.06
11	78	0.34	0.00	0.15	0.64	0.41	0.00	0.10	0.04	0.15	0.04	0.26	0.07
11	79	0.36	0.02	0.05	0.59	0.36	0.00	0.10	0.00	0.14	0.17	0.30	0.06
11	80	0.00	0.00	0.07	0.50	0.30	0.00	0.10	0.02	0.11	0.28	0.29	0.09
11	81	0.30	0.00	0.23	0.60	0.24	0.00	0.10	0.03	0.07	0.14	0.29	0.06
11	82	0.42	0.00	0.28	0.85	0.18	0.00	0.11	0.00	0.04	0.23	0.24	0.04
11	83	0.48	0.15	0.32	1.05	0.13	0.00	0.12	0.00	0.02	0.12	0.21	0.03
11	84	0.38	0.16	0.31	1.10	0.10	0.00	0.12	0.00	0.01	0.17	0.16	0.02
11	85	0.31	0.00	0.16	1.07	0.09	0.00	0.09	0.01	0.01	0.00	0.11	0.01
11	86	0.35	0.13	0.18	1.02	0.08	0.00	0.08	0.00	0.01	0.00	0.10	0.00
11	87	0.26	0.21	0.16	0.96	0.06	0.00	0.05	0.02	0.00	0.00	0.13	0.00
11	88	0.22	0.31	0.14	0.97	0.05	0.00	0.05	0.02	0.01	0.00	0.12	0.00
11	89	0.26	0.28	0.16	0.97	0.04	0.00	0.06	0.00	0.01	0.00	0.14	0.00
11	90	0.31	0.09	0.12	0.94	0.05	0.00	0.06	0.00	0.01	0.00	0.16	0.00
11	91	0.28	0.21	0.14	0.89	0.05	0.00	0.08	0.00	0.01	0.00	0.20	0.00
11	92	0.26	0.16	0.12	0.82	0.05	0.00	0.09	0.00	0.01	0.01	0.24	0.00
11	93	0.34	0.16	0.13	0.77	0.06	0.00	0.10	0.03	0.01	0.00	0.31	0.00

11	94	0.30	0.31	0.00	0.76	0.05	0.00	0.12	0.00	0.01	0.00	0.35	0.00
11	95	0.25	0.16	0.11	0.73	0.05	0.00	0.13	0.03	0.01	0.04	0.38	0.00
11	96	0.34	0.37	0.01	0.71	0.04	0.00	0.14	0.04	0.01	0.01	0.40	0.00
11	97	0.34	0.46	0.11	0.65	0.04	0.03	0.15	0.04	0.00	0.12	0.41	0.00
11	98	0.32	0.44	0.02	0.64	0.04	0.05	0.16	0.00	0.00	0.00	0.43	0.00
11	99	0.33	0.33	0.11	0.58	0.03	0.09	0.15	0.00	0.00	0.01	0.34	0.00
11	100	0.28	0.45	0.11	0.52	0.03	0.14	0.17	0.04	0.01	0.00	0.46	0.00
11	101	0.17	0.18	0.05	0.44	0.02	0.19	0.16	0.03	0.01	0.14	0.42	0.00
11	102	0.16	0.33	0.00	0.33	0.02	0.25	0.16	0.04	0.01	0.02	0.41	0.00
11	103	0.07	0.35	0.10	0.27	0.01	0.31	0.16	0.00	0.01	0.06	0.42	0.00
11	104	0.03	0.22	0.07	0.20	0.01	0.34	0.13	0.00	0.01	0.10	0.41	0.00
11	105	0.16	0.03	0.00	0.14	0.01	0.34	0.14	0.00	0.02	0.16	0.39	0.00
11	106	0.00	0.07	0.08	0.07	0.01	0.31	0.12	0.00	0.03	0.21	0.43	0.00
11	107	0.00	0.00	0.07	0.05	0.01	0.31	0.10	0.04	0.04	0.06	0.40	0.00
11	108	0.02	0.03	0.08	0.05	0.01	0.30	0.08	0.04	0.05	0.28	0.43	0.00
11	109	0.06	0.19	0.05	0.02	0.00	0.29	0.06	0.07	0.06	0.17	0.46	0.00
11	110	0.00	0.19	0.04	0.00	0.00	0.27	0.04	0.08	0.08	0.16	0.48	0.00
11	111	0.00	0.33	0.08	0.00	0.00	0.25	0.03	0.10	0.09	0.18	0.51	0.00
11	112	0.00	0.29	0.09	0.00	0.00	0.23	0.02	0.12	0.11	0.26	0.57	0.00
11	113	0.02	0.30	0.08	0.00	0.00	0.21	0.01	0.14	0.13	0.26	0.63	0.00
11	114	0.00	0.22	0.08	0.00	0.00	0.18	0.02	0.16	0.15	0.30	0.62	0.00
11	115	0.00	0.27	0.08	0.00	0.00	0.16	0.01	0.14	0.17	0.27	0.72	0.00
11	116	0.00	0.02	0.08	0.00	0.00	0.14	0.02	0.26	0.19	0.28	0.78	0.00
11	117	0.00	0.22	0.00	0.00	0.00	0.11	0.02	0.27	0.22	0.31	0.83	0.00
11	118	0.00	0.18	0.05	0.00	0.00	0.07	0.02	0.36	0.26	0.16	0.93	0.00
11	119	0.01	0.40	0.08	0.00	0.00	0.04	0.02	0.42	0.30	0.34	1.03	0.00
11	120	0.00	0.07	0.08	0.00	0.00	0.02	0.02	0.47	0.35	0.24	1.06	0.00
11	121	0.00	0.25	0.09	0.00	0.00	0.01	0.02	0.43	0.40	0.31	1.15	0.00
11	122	0.00	0.44	0.00	0.00	0.00	0.00	0.02	0.38	0.45	0.28	1.15	0.00
11	123	0.02	0.16	0.08	0.00	0.00	0.00	0.02	0.37	0.49	0.23	1.10	0.00
11	124	0.03	0.10	0.07	0.00	0.00	0.00	0.02	0.42	0.54	0.16	0.98	0.00
11	125	0.00	0.06	0.06	0.00	0.00	0.00	0.02	0.49	0.54	0.12	0.75	0.00
11	126	0.00	0.00	0.07	0.00	0.00	0.00	0.02	0.53	0.53	0.08	0.70	0.00
11	127	0.01	0.00	0.09	0.00	0.00	0.00	0.02	0.56	0.51	0.12	0.62	0.00
11	128	0.00	0.14	0.09	0.00	0.00	0.00	0.01	0.58	0.50	0.00	0.53	0.00
11	129	0.00	0.00	0.01	0.00	0.00	0.00	0.02	0.59	0.48	0.04	0.46	0.00

11	130	0.02	0.10	0.00	0.00	0.00	0.00	0.02	0.57	0.45	0.00	0.34	0.00
11	131	0.02	0.04	0.07	0.00	0.00	0.00	0.02	0.53	0.43	0.00	0.29	0.00
11	132	0.02	0.00	0.07	0.00	0.00	0.01	0.02	0.51	0.39	0.00	0.23	0.00
11	133	0.00	0.00	0.08	0.00	0.00	0.01	0.02	0.42	0.37	0.00	0.18	0.00
11	134	0.00	0.00	0.08	0.00	0.00	0.01	0.01	0.47	0.35	0.00	0.16	0.00
11	135	0.00	0.00	0.08	0.00	0.00	0.00	0.01	0.46	0.34	0.00	0.14	0.00
11	136	0.01	0.00	0.02	0.00	0.00	0.00	0.02	0.45	0.31	0.00	0.12	0.00
11	137	0.00	0.03	0.09	0.00	0.00	0.00	0.02	0.41	0.29	0.00	0.09	0.00
11	138	0.01	0.02	0.08	0.00	0.00	0.00	0.01	0.40	0.27	0.00	0.06	0.00
11	139	0.00	0.00	0.00	0.00	0.00	0.00	0.02	0.41	0.25	0.00	0.03	0.00
11	140	0.00	0.01	0.01	0.00	0.00	0.00	0.02	0.41	0.23	0.00	0.03	0.00
11	141	0.00	0.00	0.06	0.00	0.00	0.00	0.02	0.42	0.21	0.00	0.00	0.00
11	142	0.01	0.00	0.06	0.00	0.00	0.00	0.02	0.46	0.20	0.00	0.00	0.00
11	143	0.00	0.00	0.08	0.00	0.00	0.00	0.01	0.45	0.18	0.00	0.00	0.00
11	144	0.00	0.00	0.00	0.00	0.00	0.00	0.02	0.44	0.15	0.00	0.00	0.00
11	145	0.00	0.00	0.00	0.00	0.00	0.00	0.02	0.43	0.14	0.00	0.00	0.00
11	146	0.00	0.03	0.00	0.00	0.00	0.00	0.02	0.41	0.12	0.00	0.01	0.00
11	147	0.03	0.00	0.00	0.00	0.00	0.00	0.02	0.36	0.11	0.00	0.01	0.00
11	148	0.00	0.01	0.11	0.00	0.00	0.00	0.02	0.42	0.11	0.00	0.01	0.00
11	149	0.00	0.03	0.16	0.00	0.00	0.00	0.02	0.47	0.10	0.00	0.01	0.00
11	150	0.00	0.00	0.21	0.00	0.00	0.00	0.02	0.49	0.11	0.00	0.00	0.00
11	151	0.00	0.01	0.27	0.00	0.00	0.00	0.02	0.52	0.11	0.03	0.00	0.00
11	152	0.00	0.01	0.36	0.00	0.00	0.00	0.02	0.46	0.11	0.00	0.00	0.00
11	153	0.00	0.00	0.42	0.00	0.00	0.01	0.01	0.57	0.11	0.00	0.00	0.00
12	1	0.01	0.00	0.19	0.00	0.10	0.00	0.08	0.10	0.06	0.00	0.22	0.05
12	2	0.00	0.06	0.20	0.00	0.09	0.00	0.09	0.13	0.06	0.05	0.34	0.05
12	3	0.00	0.05	0.19	0.00	0.07	0.00	0.10	0.13	0.06	0.04	0.34	0.06
12	4	0.00	0.35	0.19	0.00	0.05	0.00	0.11	0.08	0.06	0.00	0.34	0.06
12	5	0.00	0.34	0.20	0.00	0.04	0.00	0.12	0.09	0.06	0.07	0.33	0.06
12	6	0.00	0.27	0.19	0.00	0.03	0.00	0.11	0.07	0.06	0.00	0.34	0.06
12	7	0.00	0.26	0.16	0.00	0.01	0.00	0.13	0.05	0.06	0.00	0.32	0.06
12	8	0.02	0.22	0.20	0.00	0.01	0.00	0.14	0.12	0.07	0.08	0.26	0.06
12	9	0.00	0.19	0.17	0.00	0.00	0.00	0.14	0.03	0.07	0.04	0.30	0.06
12	10	0.00	0.14	0.20	0.00	0.00	0.00	0.13	0.00	0.07	0.00	0.26	0.06
12	11	0.00	0.08	0.04	0.00	0.00	0.00	0.12	0.04	0.07	0.00	0.27	0.06
12	12	0.00	0.05	0.18	0.00	0.00	0.00	0.12	0.03	0.07	0.01	0.23	0.06

12	13	0.00	0.05	0.19	0.00	0.00	0.00	0.11	0.00	0.07	0.00	0.13	0.06
12	14	0.00	0.05	0.11	0.00	0.00	0.00	0.10	0.02	0.07	0.00	0.09	0.06
12	15	0.00	0.06	0.20	0.00	0.00	0.00	0.09	0.00	0.07	0.00	0.15	0.06
12	16	0.00	0.05	0.18	0.00	0.00	0.00	0.08	0.04	0.08	0.00	0.13	0.06
12	17	0.00	0.00	0.20	0.00	0.00	0.00	0.07	0.00	0.08	0.00	0.16	0.06
12	18	0.00	0.00	0.19	0.00	0.00	0.00	0.06	0.00	0.09	0.00	0.14	0.06
12	19	0.00	0.01	0.15	0.00	0.00	0.00	0.05	0.00	0.10	0.00	0.14	0.06
12	20	0.00	0.10	0.19	0.00	0.00	0.00	0.04	0.00	0.10	0.00	0.17	0.06
12	21	0.00	0.12	0.05	0.00	0.00	0.00	0.03	0.00	0.11	0.00	0.15	0.06
12	22	0.00	0.00	0.12	0.00	0.00	0.00	0.03	0.00	0.11	0.00	0.11	0.06
12	23	0.00	0.02	0.12	0.00	0.00	0.00	0.02	0.03	0.12	0.00	0.18	0.06
12	24	0.00	0.00	0.20	0.00	0.00	0.00	0.02	0.04	0.12	0.01	0.26	0.06
12	25	0.00	0.11	0.19	0.00	0.00	0.00	0.02	0.03	0.12	0.01	0.33	0.06
12	26	0.00	0.13	0.20	0.00	0.00	0.00	0.02	0.02	0.11	0.02	0.35	0.06
12	27	0.00	0.17	0.07	0.00	0.00	0.00	0.02	0.00	0.12	0.00	0.31	0.06
12	28	0.01	0.00	0.19	0.00	0.00	0.00	0.02	0.00	0.12	0.00	0.42	0.06
12	29	0.00	0.05	0.18	0.00	0.01	0.00	0.01	0.00	0.12	0.05	0.36	0.06
12	30	0.02	0.00	0.20	0.00	0.03	0.00	0.02	0.07	0.11	0.06	0.46	0.05
12	31	0.00	0.47	0.12	0.00	0.05	0.00	0.01	0.10	0.11	0.07	0.45	0.05
12	32	0.00	0.20	0.19	0.00	0.05	0.00	0.01	0.10	0.10	0.03	0.43	0.05
12	33	0.01	0.08	0.07	0.00	0.05	0.00	0.01	0.13	0.09	0.01	0.32	0.05
12	34	0.00	0.03	0.20	0.00	0.05	0.00	0.01	0.11	0.08	0.00	0.23	0.06
12	35	0.00	0.11	0.12	0.00	0.04	0.00	0.02	0.12	0.07	0.01	0.14	0.06
12	36	0.00	0.20	0.19	0.00	0.04	0.00	0.02	0.13	0.06	0.00	0.15	0.06
12	37	0.00	0.21	0.18	0.00	0.05	0.00	0.02	0.13	0.04	0.00	0.11	0.06
12	38	0.00	0.18	0.18	0.00	0.07	0.00	0.01	0.13	0.06	0.00	0.03	0.06
12	39	0.01	0.05	0.17	0.00	0.09	0.00	0.02	0.11	0.06	0.00	0.04	0.05
12	40	0.00	0.00	0.18	0.00	0.11	0.00	0.02	0.11	0.07	0.00	0.00	0.05
12	41	0.00	0.00	0.18	0.00	0.14	0.00	0.02	0.10	0.07	0.00	0.00	0.06
12	42	0.00	0.12	0.20	0.00	0.16	0.00	0.01	0.12	0.07	0.00	0.01	0.04
12	43	0.00	0.08	0.20	0.00	0.19	0.00	0.01	0.11	0.07	0.00	0.00	0.06
12	44	0.00	0.07	0.19	0.00	0.22	0.00	0.01	0.08	0.07	0.00	0.00	0.06
12	45	0.00	0.05	0.00	0.00	0.25	0.00	0.02	0.10	0.07	0.00	0.00	0.06
12	46	0.00	0.00	0.18	0.00	0.27	0.00	0.01	0.08	0.07	0.00	0.00	0.06
12	47	0.01	0.03	0.20	0.00	0.27	0.00	0.02	0.12	0.07	0.00	0.01	0.06
12	48	0.00	0.01	0.12	0.00	0.27	0.00	0.02	0.17	0.06	0.00	0.01	0.06

12	49	0.00	0.00	0.20	0.00	0.26	0.00	0.02	0.15	0.06	0.00	0.01	0.06
12	50	0.00	0.02	0.15	0.00	0.26	0.00	0.02	0.16	0.05	0.00	0.01	0.05
12	51	0.00	0.00	0.04	0.00	0.25	0.00	0.02	0.16	0.05	0.00	0.01	0.05
12	52	0.00	0.00	0.20	0.00	0.24	0.01	0.02	0.18	0.04	0.00	0.00	0.06
12	53	0.00	0.00	0.23	0.00	0.24	0.02	0.02	0.22	0.04	0.00	0.01	0.07
12	54	0.00	0.00	0.25	0.00	0.23	0.03	0.02	0.21	0.03	0.00	0.01	0.08
12	55	0.00	0.00	0.27	0.00	0.22	0.03	0.03	0.17	0.03	0.00	0.01	0.09
12	56	0.01	0.00	0.21	0.00	0.22	0.02	0.05	0.20	0.03	0.00	0.01	0.08
12	57	0.00	0.00	0.10	0.00	0.23	0.00	0.08	0.23	0.05	0.00	0.01	0.06
12	58	0.05	0.00	0.27	0.00	0.23	0.00	0.11	0.26	0.06	0.00	0.01	0.05
12	59	0.03	0.00	0.25	0.00	0.23	0.00	0.13	0.26	0.07	0.00	0.01	0.06
12	60	0.00	0.00	0.21	0.00	0.23	0.00	0.11	0.30	0.08	0.01	0.01	0.06
12	61	0.07	0.00	0.18	0.00	0.22	0.00	0.10	0.32	0.09	0.00	0.01	0.06
12	62	0.07	0.00	0.18	0.00	0.21	0.00	0.10	0.32	0.10	0.00	0.01	0.06
12	63	0.10	0.00	0.07	0.00	0.19	0.00	0.10	0.31	0.11	0.03	0.01	0.06
12	64	0.00	0.00	0.23	0.00	0.17	0.00	0.10	0.33	0.13	0.01	0.01	0.06
12	65	0.00	0.00	0.25	0.00	0.15	0.00	0.11	0.27	0.14	0.02	0.01	0.07
12	66	0.14	0.00	0.27	0.00	0.13	0.00	0.11	0.27	0.15	0.00	0.00	0.09
12	67	0.04	0.00	0.28	0.00	0.12	0.00	0.11	0.25	0.16	0.00	0.01	0.08
12	68	0.04	0.02	0.20	0.00	0.12	0.00	0.12	0.26	0.15	0.15	0.01	0.10
12	69	0.22	0.00	0.11	0.00	0.12	0.00	0.13	0.20	0.14	0.03	0.01	0.09
12	70	0.20	0.02	0.22	0.00	0.12	0.00	0.14	0.22	0.14	0.19	0.01	0.08
12	71	0.17	0.00	0.21	0.00	0.12	0.01	0.15	0.17	0.14	0.03	0.01	0.08
12	72	0.01	0.02	0.14	0.00	0.11	0.03	0.16	0.15	0.13	0.10	0.01	0.08
12	73	0.10	0.02	0.19	0.00	0.11	0.06	0.16	0.09	0.09	0.15	0.01	0.08
12	74	0.04	0.00	0.19	0.00	0.10	0.09	0.17	0.10	0.11	0.19	0.01	0.07
12	75	0.00	0.00	0.06	0.00	0.10	0.10	0.15	0.10	0.12	0.04	0.01	0.07
12	76	0.04	0.01	0.18	0.00	0.10	0.11	0.14	0.09	0.12	0.00	0.01	0.06
12	77	0.00	0.02	0.18	0.00	0.10	0.12	0.13	0.12	0.13	0.28	0.01	0.06
12	78	0.06	0.00	0.19	0.00	0.10	0.13	0.11	0.17	0.14	0.14	0.01	0.05
12	79	0.02	0.00	0.04	0.00	0.10	0.14	0.10	0.21	0.15	0.20	0.01	0.05
12	80	0.00	0.00	0.19	0.00	0.09	0.15	0.08	0.24	0.16	0.42	0.01	0.05
12	81	0.00	0.02	0.18	0.00	0.09	0.16	0.06	0.33	0.17	0.45	0.00	0.06
12	82	0.05	0.02	0.20	0.00	0.09	0.17	0.06	0.37	0.17	0.41	0.01	0.04
12	83	0.12	0.00	0.04	0.00	0.08	0.18	0.04	0.41	0.18	0.57	0.01	0.06
12	84	0.19	0.02	0.05	0.00	0.07	0.18	0.03	0.43	0.18	0.54	0.01	0.06

12	85	0.15	0.00	0.19	0.00	0.07	0.19	0.02	0.32	0.19	0.52	0.01	0.06
12	86	0.14	0.00	0.19	0.00	0.05	0.19	0.02	0.27	0.20	0.49	0.00	0.06
12	87	0.11	0.00	0.11	0.00	0.05	0.19	0.02	0.14	0.20	0.48	0.01	0.06
12	88	0.09	0.00	0.19	0.00	0.05	0.19	0.02	0.16	0.20	0.32	0.00	0.06
12	89	0.06	0.00	0.19	0.00	0.04	0.19	0.02	0.15	0.21	0.44	0.01	0.06
12	90	0.06	0.00	0.20	0.00	0.04	0.18	0.02	0.10	0.21	0.45	0.00	0.06
12	91	0.06	0.00	0.19	0.00	0.03	0.19	0.02	0.06	0.20	0.50	0.00	0.05
12	92	0.02	0.00	0.19	0.00	0.04	0.14	0.02	0.09	0.27	0.48	0.03	0.05
12	93	0.00	0.08	0.00	0.00	0.06	0.10	0.02	0.04	0.37	0.44	0.02	0.06
12	94	0.00	0.00	0.18	0.00	0.08	0.06	0.01	0.03	0.48	0.51	0.09	0.06
12	95	0.00	0.16	0.03	0.00	0.09	0.03	0.02	0.13	0.59	0.65	0.13	0.06
12	96	0.01	0.02	0.19	0.00	0.12	0.01	0.02	0.15	0.72	0.67	0.14	0.06
12	97	0.03	0.00	0.19	0.00	0.14	0.00	0.02	0.20	0.83	0.54	0.24	0.06
12	98	0.06	0.00	0.08	0.00	0.17	0.00	0.02	0.21	0.95	0.67	0.34	0.06
12	99	0.11	0.07	0.11	0.01	0.19	0.00	0.02	0.23	1.06	0.66	0.35	0.05
12	100	0.06	0.40	0.17	0.02	0.21	0.00	0.02	0.24	1.16	0.59	0.29	0.06
12	101	0.11	0.41	0.19	0.01	0.24	0.00	0.02	0.27	1.25	0.68	0.50	0.06
12	102	0.17	0.16	0.18	0.02	0.25	0.00	0.02	0.26	1.28	0.66	0.59	0.06
12	103	0.04	0.58	0.20	0.02	0.29	0.00	0.02	0.28	1.30	0.61	0.65	0.06
12	104	0.06	0.51	0.19	0.03	0.31	0.00	0.02	0.28	1.32	0.62	0.64	0.06
12	105	0.22	0.45	0.19	0.04	0.34	0.00	0.02	0.29	1.32	0.56	0.68	0.06
12	106	0.16	0.41	0.18	0.04	0.36	0.00	0.02	0.31	1.32	0.57	0.65	0.06
12	107	0.26	0.63	0.08	0.03	0.37	0.00	0.02	0.32	1.30	0.29	0.62	0.05
12	108	0.21	0.24	0.19	0.04	0.39	0.00	0.02	0.32	1.28	0.41	0.67	0.06
12	109	0.15	0.61	0.17	0.05	0.41	0.00	0.02	0.27	1.24	0.44	0.66	0.06
12	110	0.09	0.41	0.20	0.02	0.42	0.00	0.03	0.17	1.20	0.34	0.53	0.06
12	111	0.21	0.61	0.15	0.05	0.43	0.00	0.03	0.18	1.16	0.26	0.65	0.06
12	112	0.26	0.28	0.20	0.08	0.42	0.00	0.04	0.14	1.21	0.25	0.63	0.06
12	113	0.25	0.38	0.19	0.08	0.40	0.00	0.05	0.14	1.26	0.10	0.62	0.06
12	114	0.23	0.58	0.19	0.07	0.38	0.00	0.06	0.16	1.31	0.05	0.56	0.05
12	115	0.24	0.40	0.01	0.05	0.35	0.00	0.07	0.15	1.34	0.00	0.59	0.06
12	116	0.26	0.53	0.19	0.05	0.32	0.00	0.08	0.14	1.35	0.11	0.59	0.05
12	117	0.22	0.50	0.19	0.07	0.32	0.00	0.09	0.04	1.31	0.07	0.55	0.06
12	118	0.17	0.12	0.18	0.06	0.32	0.00	0.09	0.11	1.24	0.00	0.52	0.06
12	119	0.12	0.34	0.19	0.05	0.32	0.00	0.12	0.12	1.16	0.00	0.41	0.06
12	120	0.08	0.24	0.19	0.04	0.32	0.00	0.14	0.13	1.07	0.00	0.47	0.06

12	121	0.00	0.27	0.16	0.02	0.32	0.00	0.17	0.07	0.96	0.03	0.42	0.06
12	122	0.02	0.39	0.16	0.00	0.31	0.00	0.20	0.11	0.87	0.00	0.47	0.06
12	123	0.20	0.43	0.20	0.03	0.29	0.00	0.22	0.22	0.91	0.07	0.42	0.06
12	124	0.24	0.30	0.18	0.01	0.28	0.00	0.25	0.25	0.95	0.10	0.53	0.06
12	125	0.22	0.17	0.19	0.02	0.27	0.00	0.27	0.27	0.98	0.12	0.57	0.06
12	126	0.19	0.35	0.19	0.01	0.25	0.00	0.30	0.25	0.99	0.14	0.54	0.06
12	127	0.28	0.34	0.20	0.01	0.23	0.00	0.32	0.21	0.98	0.17	0.60	0.05
12	128	0.27	0.29	0.19	0.00	0.21	0.00	0.34	0.19	0.99	0.13	0.56	0.06
12	129	0.11	0.19	0.11	0.01	0.19	0.00	0.36	0.16	0.95	0.16	0.57	0.06
12	130	0.22	0.22	0.04	0.00	0.17	0.00	0.38	0.16	0.89	0.05	0.55	0.05
12	131	0.20	0.17	0.18	0.02	0.16	0.00	0.40	0.15	0.83	0.09	0.53	0.06
12	132	0.27	0.16	0.18	0.02	0.14	0.00	0.41	0.07	0.78	0.04	0.49	0.06
12	133	0.21	0.11	0.17	0.03	0.11	0.00	0.37	0.29	0.82	0.01	0.50	0.06
12	134	0.00	0.11	0.01	0.07	0.08	0.00	0.33	0.35	0.83	0.03	0.53	0.06
12	135	0.21	0.10	0.18	0.10	0.05	0.00	0.28	0.39	0.83	0.11	0.54	0.06
12	136	0.12	0.07	0.19	0.11	0.03	0.00	0.23	0.49	0.86	0.02	0.47	0.06
12	137	0.00	0.02	0.20	0.10	0.01	0.00	0.19	0.56	0.81	0.12	0.44	0.06
12	138	0.00	0.00	0.19	0.10	0.00	0.00	0.15	0.57	0.79	0.02	0.48	0.06
12	139	0.00	0.02	0.19	0.06	0.00	0.00	0.12	0.49	0.77	0.00	0.41	0.06
12	140	0.07	0.00	0.19	0.03	0.00	0.00	0.10	0.42	0.71	0.00	0.28	0.06
12	141	0.03	0.03	0.20	0.01	0.00	0.00	0.08	0.44	0.69	0.03	0.29	0.06
12	142	0.00	0.03	0.15	0.00	0.00	0.00	0.08	0.36	0.66	0.01	0.18	0.06
12	143	0.00	0.00	0.20	0.00	0.00	0.00	0.07	0.22	0.65	0.00	0.22	0.06
12	144	0.00	0.02	0.20	0.00	0.00	0.00	0.06	0.21	0.71	0.00	0.17	0.06
12	145	0.00	0.00	0.18	0.00	0.00	0.00	0.06	0.14	0.76	0.00	0.16	0.06
12	146	0.00	0.00	0.18	0.00	0.00	0.00	0.05	0.00	0.83	0.00	0.14	0.06
12	147	0.00	0.00	0.20	0.00	0.00	0.00	0.04	0.00	0.98	0.00	0.06	0.06
12	148	0.00	0.00	0.18	0.00	0.00	0.00	0.04	0.01	1.09	0.00	0.16	0.06
12	149	0.00	0.02	0.19	0.00	0.00	0.00	0.03	0.04	1.20	0.00	0.15	0.04
12	150	0.01	0.00	0.17	0.00	0.00	0.00	0.03	0.04	1.31	0.00	0.18	0.06
12	151	0.00	0.01	0.10	0.00	0.00	0.00	0.03	0.02	1.41	0.04	0.31	0.06
12	152	0.00	0.00	0.19	0.00	0.00	0.00	0.03	0.00	1.51	0.00	0.44	0.06
12	153	0.00	0.00	0.13	0.00	0.00	0.00	0.03	0.00	1.59	0.03	0.54	0.06
12	154	0.00	0.00	0.18	0.00	0.00	0.00	0.04	0.02	1.56	0.09	0.60	0.06
12	155	0.00	0.01	0.02	0.00	0.00	0.00	0.05	0.04	1.43	0.05	0.60	0.06
12	156	0.00	0.00	0.18	0.00	0.00	0.00	0.09	0.02	1.33	0.05	0.59	0.06

12	157	0.00	0.00	0.13	0.00	0.00	0.00	0.13	0.04	1.25	0.08	0.58	0.06
12	158	0.00	0.04	0.11	0.00	0.00	0.00	0.18	0.02	1.14	0.00	0.63	0.06
12	159	0.00	0.20	0.10	0.00	0.00	0.00	0.25	0.03	1.03	0.00	0.64	0.06
12	160	0.00	0.18	0.06	0.00	0.00	0.00	0.32	0.03	0.92	0.11	0.64	0.06
12	161	0.00	0.12	0.19	0.00	0.00	0.00	0.39	0.04	0.84	0.07	0.56	0.06
12	162	0.00	0.07	0.10	0.00	0.00	0.00	0.47	0.03	0.75	0.00	0.58	0.06
12	163	0.00	0.20	0.08	0.00	0.00	0.00	0.55	0.03	0.65	0.08	0.47	0.06
12	164	0.00	0.10	0.09	0.00	0.00	0.00	0.62	0.03	0.56	0.08	0.40	0.06
12	165	0.06	0.13	0.14	0.00	0.00	0.00	0.66	0.03	0.52	0.00	0.42	0.06
12	166	0.07	0.00	0.16	0.00	0.00	0.00	0.67	0.05	0.48	0.09	0.36	0.06
12	167	0.04	0.07	0.28	0.00	0.00	0.00	0.68	0.08	0.41	0.04	0.32	0.06
12	168	0.00	0.00	0.22	0.00	0.00	0.00	0.68	0.11	0.34	0.07	0.24	0.06
12	169	0.09	0.00	0.28	0.00	0.00	0.00	0.69	0.15	0.28	0.01	0.19	0.05
12	170	0.03	0.00	0.41	0.00	0.00	0.00	0.70	0.19	0.25	0.16	0.22	0.04
12	171	0.09	0.00	0.47	0.00	0.00	0.00	0.72	0.18	0.25	0.28	0.22	0.05
12	172	0.15	0.01	0.36	0.00	0.00	0.00	0.73	0.17	0.24	0.15	0.23	0.06
13	1	0.22	0.00	0.30	0.00	0.07	0.61	0.02	0.65	0.71	0.00	0.10	0.29
13	2	0.20	0.00	0.31	0.00	0.06	0.64	0.02	0.62	0.75	0.47	0.04	0.31
13	3	0.18	0.00	0.16	0.00	0.06	0.66	0.02	0.56	0.84	0.41	0.07	0.30
13	4	0.19	0.01	0.10	0.00	0.05	0.68	0.02	0.50	0.91	0.59	0.05	0.31
13	5	0.17	0.01	0.17	0.00	0.04	0.71	0.02	0.46	0.97	0.47	0.00	0.32
13	6	0.03	0.02	0.21	0.00	0.04	0.73	0.02	0.41	1.05	0.34	0.00	0.31
13	7	0.02	0.00	0.22	0.00	0.03	0.75	0.02	0.30	1.11	0.28	0.01	0.31
13	8	0.00	0.00	0.23	0.00	0.02	0.76	0.02	0.24	1.17	0.48	0.01	0.31
13	9	0.00	0.00	0.25	0.00	0.02	0.79	0.02	0.20	1.19	0.38	0.00	0.31
13	10	0.03	0.02	0.09	0.00	0.01	0.78	0.02	0.28	1.25	0.21	0.00	0.28
13	11	0.00	0.00	0.14	0.00	0.01	0.81	0.02	0.26	1.30	0.41	0.00	0.27
13	12	0.00	0.00	0.00	0.00	0.00	0.82	0.02	0.23	1.33	0.41	0.01	0.22
13	13	0.00	0.00	0.16	0.00	0.00	0.82	0.02	0.18	1.35	0.37	0.01	0.18
13	14	0.00	0.00	0.04	0.00	0.00	0.83	0.02	0.22	1.35	0.58	0.00	0.13
13	15	0.00	0.00	0.02	0.00	0.00	0.79	0.02	0.16	1.35	0.56	0.01	0.10
13	16	0.00	0.00	0.20	0.00	0.00	0.83	0.02	0.09	1.35	0.23	0.04	0.08
13	17	0.00	0.00	0.03	0.00	0.00	0.83	0.02	0.11	1.34	0.39	0.03	0.07
13	18	0.00	0.00	0.20	0.00	0.00	0.82	0.02	0.24	1.32	0.49	0.07	0.07
13	19	0.00	0.00	0.04	0.00	0.00	0.82	0.02	0.16	1.30	0.53	0.06	0.07
13	20	0.01	0.00	0.19	0.00	0.00	0.81	0.02	0.26	1.27	0.60	0.06	0.07

13	21	0.00	0.00	0.19	0.00	0.00	0.80	0.02	0.28	1.25	0.59	0.11	0.07
13	22	0.00	0.04	0.16	0.00	0.00	0.80	0.02	0.32	1.22	0.54	0.11	0.06
13	23	0.04	0.02	0.07	0.00	0.00	0.70	0.02	0.32	1.22	0.60	0.10	0.06
13	24	0.00	0.02	0.19	0.00	0.00	0.61	0.02	0.30	1.23	0.42	0.13	0.05
13	25	0.02	0.03	0.11	0.00	0.00	0.51	0.02	0.20	1.24	0.41	0.13	0.06
13	26	0.00	0.01	0.16	0.00	0.00	0.40	0.00	0.23	1.21	0.45	0.17	0.06
13	27	0.00	0.00	0.19	0.00	0.00	0.31	0.02	0.17	1.21	0.40	0.19	0.06
13	28	0.01	0.00	0.19	0.00	0.00	0.22	0.02	0.13	1.19	0.31	0.19	0.06
13	29	0.00	0.00	0.14	0.00	0.00	0.15	0.02	0.12	1.17	0.37	0.19	0.06
13	30	0.00	0.00	0.14	0.00	0.00	0.09	0.02	0.17	1.14	0.32	0.21	0.06
13	31	0.00	0.00	0.20	0.00	0.00	0.04	0.02	0.11	1.10	0.20	0.21	0.06
13	32	0.00	0.02	0.18	0.00	0.00	0.01	0.02	0.05	1.04	0.13	0.13	0.06
13	33	0.00	0.03	0.04	0.00	0.00	0.00	0.02	0.07	1.01	0.08	0.11	0.06
13	34	0.00	0.02	0.19	0.00	0.00	0.00	0.02	0.07	0.97	0.00	0.07	0.06
13	35	0.00	0.00	0.19	0.00	0.00	0.00	0.02	0.06	0.92	0.02	0.04	0.06
13	36	0.00	0.01	0.17	0.00	0.00	0.00	0.02	0.00	0.86	0.00	0.00	0.06
13	37	0.00	0.00	0.19	0.00	0.00	0.00	0.02	0.01	0.80	0.00	0.00	0.06
13	38	0.00	0.00	0.14	0.00	0.00	0.00	0.02	0.00	0.73	0.00	0.00	0.06
13	39	0.00	0.00	0.18	0.00	0.00	0.00	0.02	0.00	0.66	0.00	0.01	0.06
13	40	0.00	0.00	0.18	0.00	0.00	0.00	0.02	0.00	0.62	0.00	0.01	0.06
13	41	0.00	0.00	0.19	0.00	0.00	0.00	0.02	0.04	0.53	0.00	0.00	0.06
13	42	0.00	0.00	0.15	0.00	0.00	0.00	0.02	0.04	0.52	0.00	0.00	0.06
13	43	0.01	0.00	0.16	0.00	0.00	0.00	0.02	0.04	0.47	0.00	0.00	0.06
13	44	0.00	0.00	0.15	0.00	0.00	0.00	0.02	0.03	0.43	0.00	0.00	0.06
13	45	0.00	0.00	0.18	0.00	0.00	0.00	0.02	0.02	0.39	0.00	0.00	0.06
13	46	0.00	0.00	0.19	0.00	0.00	0.00	0.02	0.04	0.35	0.00	0.00	0.06
13	47	0.00	0.02	0.16	0.00	0.00	0.00	0.02	0.00	0.31	0.00	0.01	0.06
13	48	0.00	0.00	0.19	0.00	0.00	0.00	0.02	0.00	0.26	0.00	0.00	0.06
13	49	0.00	0.03	0.19	0.00	0.00	0.00	0.02	0.02	0.22	0.00	0.01	0.06
13	50	0.00	0.00	0.20	0.00	0.00	0.00	0.01	0.09	0.19	0.00	0.01	0.05
13	51	0.00	0.00	0.19	0.00	0.00	0.00	0.02	0.10	0.15	0.00	0.01	0.05
13	52	0.00	0.00	0.18	0.00	0.00	0.00	0.02	0.14	0.13	0.00	0.01	0.06
13	53	0.00	0.02	0.19	0.00	0.00	0.00	0.02	0.14	0.14	0.00	0.00	0.06
13	54	0.01	0.00	0.04	0.00	0.00	0.00	0.02	0.17	0.14	0.00	0.00	0.06
13	55	0.00	0.00	0.12	0.00	0.00	0.00	0.02	0.31	0.15	0.00	0.00	0.06
13	56	0.02	0.02	0.18	0.00	0.00	0.00	0.02	0.42	0.15	0.00	0.00	0.05

13	57	0.00	0.00	0.21	0.00	0.00	0.00	0.01	0.56	0.16	0.00	0.02	0.06
13	58	0.00	0.01	0.05	0.00	0.00	0.00	0.02	0.68	0.16	0.00	0.02	0.07
13	59	0.00	0.00	0.09	0.00	0.00	0.00	0.02	0.78	0.16	0.00	0.02	0.10
13	60	0.01	0.00	0.13	0.00	0.00	0.00	0.02	0.70	0.17	0.00	0.00	0.13
13	61	0.00	0.00	0.20	0.00	0.00	0.00	0.02	0.66	0.17	0.00	0.00	0.13
13	62	0.00	0.00	0.11	0.00	0.00	0.00	0.02	0.62	0.17	0.00	0.00	0.13
13	63	0.00	0.00	0.08	0.00	0.00	0.00	0.02	0.54	0.16	0.00	0.01	0.13
13	64	0.00	0.00	0.07	0.00	0.00	0.00	0.02	0.51	0.17	0.00	0.07	0.13
13	65	0.00	0.03	0.15	0.00	0.00	0.00	0.02	0.52	0.17	0.00	0.10	0.14
13	66	0.00	0.05	0.19	0.00	0.00	0.00	0.02	0.50	0.17	0.01	0.13	0.16
13	67	0.01	0.01	0.19	0.00	0.00	0.00	0.02	0.50	0.16	0.00	0.15	0.16
13	68	0.00	0.00	0.19	0.00	0.01	0.00	0.02	0.36	0.16	0.00	0.16	0.17
13	69	0.00	0.00	0.12	0.00	0.02	0.00	0.02	0.42	0.15	0.00	0.11	0.16
13	70	0.00	0.17	0.19	0.00	0.02	0.00	0.02	0.40	0.13	0.00	0.15	0.16
13	71	0.00	0.13	0.22	0.00	0.03	0.00	0.03	0.36	0.11	0.00	0.13	0.16
13	72	0.00	0.22	0.17	0.00	0.03	0.00	0.04	0.34	0.07	0.00	0.11	0.13
13	73	0.00	0.21	0.10	0.00	0.03	0.00	0.07	0.27	0.04	0.00	0.09	0.12
13	74	0.04	0.07	0.19	0.00	0.04	0.00	0.09	0.17	0.04	0.00	0.06	0.12
13	75	0.00	0.19	0.11	0.00	0.04	0.00	0.13	0.06	0.02	0.00	0.04	0.14
13	76	0.08	0.00	0.15	0.00	0.03	0.00	0.16	0.11	0.01	0.00	0.02	0.15
13	77	0.00	0.00	0.25	0.00	0.02	0.00	0.19	0.06	0.01	0.00	0.00	0.16
13	78	0.00	0.00	0.25	0.00	0.01	0.01	0.19	0.02	0.01	0.00	0.00	0.18
13	79	0.00	0.00	0.23	0.00	0.00	0.01	0.18	0.05	0.00	0.00	0.01	0.21
13	80	0.02	0.00	0.30	0.00	0.00	0.02	0.17	0.07	0.01	0.00	0.01	0.25
13	81	0.00	0.02	0.15	0.00	0.00	0.03	0.16	0.08	0.00	0.00	0.01	0.30
13	82	0.00	0.00	0.32	0.00	0.00	0.06	0.15	0.13	0.01	0.00	0.01	0.32
13	83	0.05	0.00	0.36	0.00	0.00	0.09	0.14	0.14	0.01	0.00	0.01	0.33
13	84	0.01	0.01	0.25	0.00	0.00	0.14	0.13	0.20	0.01	0.00	0.01	0.33
13	85	0.06	0.00	0.36	0.00	0.00	0.19	0.12	0.25	0.00	0.00	0.01	0.34
13	86	0.02	0.00	0.42	0.01	0.00	0.25	0.10	0.26	0.00	0.00	0.01	0.33
13	87	0.08	0.00	0.45	0.04	0.00	0.30	0.09	0.29	0.00	0.00	0.01	0.39
13	88	0.11	0.01	0.48	0.05	0.00	0.34	0.07	0.31	0.01	0.00	0.01	0.41
13	89	0.00	0.02	0.53	0.08	0.00	0.38	0.06	0.37	0.01	0.00	0.01	0.43
13	90	0.00	0.02	0.46	0.07	0.00	0.42	0.06	0.39	0.01	0.00	0.01	0.48
13	91	0.00	0.00	0.64	0.09	0.00	0.44	0.08	0.42	0.01	0.00	0.01	0.52
13	92	0.00	0.00	0.58	0.03	0.00	0.45	0.09	0.38	0.01	0.00	0.00	0.56

13	93	0.00	0.00	0.73	0.00	0.00	0.45	0.10	0.30	0.01	0.00	0.01	0.58
13	94	0.01	0.00	0.71	0.00	0.00	0.45	0.12	0.24	0.01	0.00	0.01	0.69
13	95	0.00	0.00	0.69	0.00	0.00	0.43	0.13	0.17	0.01	0.00	0.01	0.74
13	96	0.00	0.00	0.41	0.00	0.00	0.41	0.12	0.12	0.01	0.00	0.01	0.71
13	97	0.00	0.00	0.42	0.00	0.00	0.39	0.10	0.08	0.01	0.00	0.01	0.57
13	98	0.00	0.00	0.33	0.00	0.00	0.36	0.07	0.07	0.00	0.00	0.01	0.44
13	99	0.01	0.00	0.14	0.00	0.00	0.33	0.06	0.08	0.00	0.00	0.01	0.36
13	100	0.00	0.00	0.06	0.00	0.00	0.39	0.06	0.01	0.00	0.06	0.01	0.35
13	101	0.00	0.00	0.24	0.00	0.00	0.44	0.06	0.00	0.01	0.00	0.00	0.39
13	102	0.00	0.00	0.14	0.00	0.00	0.50	0.06	0.00	0.00	0.13	0.01	0.45
13	103	0.00	0.01	0.28	0.00	0.00	0.56	0.07	0.01	0.01	0.14	0.00	0.49
13	104	0.00	0.00	0.18	0.00	0.00	0.61	0.06	0.04	0.01	0.21	0.01	0.53
13	105	0.00	0.00	0.31	0.00	0.00	0.66	0.06	0.02	0.00	0.23	0.01	0.56
13	106	0.00	0.00	0.17	0.00	0.00	0.70	0.06	0.02	0.00	0.30	0.00	0.59
13	107	0.02	0.00	0.36	0.00	0.00	0.72	0.06	0.02	0.00	0.30	0.00	0.60
13	108	0.01	0.00	0.29	0.00	0.00	0.74	0.06	0.03	0.00	0.39	0.00	0.60
13	109	0.02	0.00	0.42	0.00	0.00	0.73	0.06	0.04	0.01	0.49	0.02	0.61
13	110	0.04	0.00	0.49	0.03	0.00	0.80	0.07	0.00	0.01	0.46	0.01	0.49
13	111	0.06	0.00	0.38	0.03	0.00	0.85	0.10	0.00	0.00	0.41	0.01	0.38
13	112	0.00	0.00	0.51	0.03	0.00	0.88	0.14	0.02	0.00	0.25	0.00	0.33
13	113	0.06	0.00	0.53	0.03	0.00	0.89	0.18	0.00	0.01	0.25	0.02	0.24
13	114	0.07	0.01	0.33	0.01	0.00	0.87	0.22	0.00	0.01	0.31	0.00	0.19
13	115	0.02	0.00	0.50	0.01	0.00	0.86	0.23	0.02	0.01	0.25	0.04	0.14
13	116	0.00	0.00	0.16	0.00	0.00	0.85	0.22	0.04	0.00	0.23	0.00	0.09
13	117	0.02	0.00	0.29	0.00	0.00	0.83	0.20	0.00	0.00	0.20	0.05	0.06
13	118	0.02	0.00	0.30	0.00	0.00	0.80	0.18	0.00	0.01	0.14	0.06	0.06
13	119	0.00	0.02	0.26	0.00	0.00	0.76	0.16	0.00	0.01	0.04	0.08	0.06
13	120	0.00	0.00	0.21	0.00	0.00	0.71	0.14	0.00	0.01	0.09	0.02	0.06
13	121	0.00	0.00	0.19	0.00	0.00	0.66	0.11	0.02	0.01	0.00	0.09	0.06
13	122	0.00	0.00	0.12	0.00	0.00	0.61	0.10	0.04	0.01	0.01	0.08	0.06
13	123	0.00	0.00	0.19	0.00	0.00	0.55	0.08	0.03	0.00	0.00	0.11	0.06
13	124	0.00	0.00	0.09	0.00	0.00	0.50	0.07	0.04	0.00	0.01	0.12	0.06
13	125	0.00	0.00	0.18	0.00	0.00	0.00	0.00	0.02	0.00	0.02	0.12	0.06
13	126	0.00	0.00	0.16	0.00	0.00	0.00	0.00	0.00	0.00	0.01	0.11	0.06
13	127	0.00	0.02	0.19	0.00	0.00	0.00	0.00	0.00	0.00	0.00	0.14	0.06
13	128	0.00	0.00	0.06	0.00	0.00	0.00	0.00	0.04	0.00	0.00	0.14	0.06

13	129	0.00	0.02	0.17	0.00	0.00	0.00	0.00	0.04	0.00	0.00	0.15	0.06
14	4	0.00	0.00	0.04	0.00	0.00	0.31	0.02	0.00	0.06	0.00	0.41	0.06
14	5	0.00	0.04	0.00	0.00	0.00	0.28	0.02	0.04	0.08	0.30	0.44	0.06
14	6	0.00	0.03	0.03	0.00	0.00	0.24	0.02	0.04	0.10	0.25	0.48	0.06
14	7	0.00	0.05	0.20	0.00	0.00	0.21	0.02	0.00	0.11	0.34	0.56	0.06
14	8	0.00	0.05	0.19	0.00	0.00	0.18	0.02	0.04	0.14	0.36	0.60	0.06
14	9	0.00	0.00	0.20	0.00	0.00	0.15	0.02	0.03	0.17	0.29	0.59	0.06
14	10	0.00	0.05	0.15	0.00	0.00	0.12	0.02	0.02	0.17	0.38	0.54	0.05
14	11	0.00	0.05	0.08	0.00	0.00	0.10	0.02	0.00	0.16	0.37	0.61	0.06
14	12	0.00	0.00	0.20	0.00	0.00	0.08	0.02	0.00	0.15	0.27	0.59	0.06
14	13	0.00	0.04	0.20	0.00	0.00	0.07	0.02	0.00	0.13	0.22	0.58	0.06
14	14	0.00	0.02	0.16	0.00	0.00	0.05	0.02	0.04	0.12	0.30	0.53	0.02
14	15	0.00	0.02	0.18	0.01	0.00	0.05	0.02	0.00	0.14	0.34	0.56	0.03
14	16	0.00	0.00	0.17	0.03	0.00	0.05	0.02	0.04	0.13	0.41	0.54	0.08
14	17	0.00	0.00	0.23	0.05	0.00	0.05	0.02	0.00	0.14	0.50	0.52	0.09
14	18	0.00	0.00	0.10	0.04	0.00	0.05	0.02	0.02	0.13	0.51	0.49	0.10
14	19	0.00	0.04	0.23	0.12	0.00	0.05	0.02	0.00	0.13	0.51	0.45	0.10
14	20	0.00	0.03	0.24	0.17	0.00	0.05	0.00	0.04	0.14	0.60	0.44	0.11
14	21	0.00	0.04	0.25	0.26	0.00	0.05	0.02	0.00	0.15	0.65	0.43	0.11
14	22	0.00	0.00	0.29	0.34	0.00	0.06	0.02	0.03	0.16	0.71	0.41	0.11
14	23	0.00	0.03	0.29	0.28	0.00	0.06	0.02	0.00	0.17	0.68	0.50	0.06
14	24	0.01	0.00	0.30	0.24	0.01	0.07	0.02	0.03	0.21	0.68	0.51	0.07
14	25	0.00	0.03	0.29	0.23	0.01	0.09	0.01	0.01	0.24	0.78	0.55	0.06
14	26	0.04	0.00	0.28	0.23	0.02	0.09	0.02	0.00	0.26	0.80	0.59	0.03
14	27	0.00	0.00	0.22	0.21	0.04	0.10	0.02	0.00	0.23	0.76	0.57	0.06
14	28	0.04	0.03	0.24	0.18	0.05	0.11	0.02	0.04	0.17	0.71	0.51	0.05
14	29	0.00	0.00	0.14	0.21	0.07	0.12	0.02	0.00	0.14	0.52	0.48	0.06
14	30	0.02	0.00	0.17	0.20	0.09	0.12	0.02	0.01	0.13	0.39	0.46	0.06
14	31	0.00	0.00	0.21	0.20	0.11	0.13	0.02	0.00	0.11	0.48	0.39	0.05
14	32	0.01	0.01	0.21	0.16	0.13	0.13	0.02	0.00	0.09	0.43	0.39	0.06
14	33	0.00	0.02	0.01	0.17	0.15	0.13	0.02	0.00	0.08	0.41	0.36	0.06
14	34	0.00	0.00	0.20	0.16	0.17	0.14	0.02	0.02	0.09	0.44	0.41	0.06
14	35	0.00	0.03	0.13	0.26	0.19	0.13	0.02	0.04	0.09	0.38	0.39	0.06
14	36	0.00	0.03	0.06	0.31	0.21	0.12	0.02	0.04	0.10	0.20	0.37	0.06
14	37	0.07	0.00	0.19	0.30	0.22	0.16	0.02	0.04	0.11	0.36	0.28	0.05
14	38	0.08	0.00	0.09	0.32	0.22	0.19	0.03	0.00	0.13	0.31	0.30	0.06

14	39	0.00	0.00	0.18	0.36	0.23	0.22	0.04	0.04	0.14	0.37	0.28	0.06
14	40	0.05	0.00	0.19	0.35	0.23	0.24	0.05	0.00	0.14	0.53	0.26	0.06
14	41	0.23	0.03	0.19	0.35	0.24	0.27	0.07	0.00	0.15	0.57	0.22	0.06
14	42	0.26	0.00	0.19	0.34	0.24	0.29	0.09	0.00	0.15	0.51	0.19	0.06
14	43	0.20	0.00	0.19	0.31	0.24	0.31	0.11	0.00	0.08	0.55	0.11	0.06
14	44	0.07	0.02	0.19	0.28	0.24	0.32	0.12	0.00	0.03	0.45	0.06	0.06
14	45	0.14	0.00	0.04	0.18	0.24	0.30	0.12	0.04	0.04	0.44	0.00	0.04
14	46	0.08	0.00	0.19	0.15	0.23	0.30	0.11	0.00	0.03	0.26	0.04	0.06
14	47	0.09	0.00	0.12	0.12	0.24	0.29	0.11	0.00	0.02	0.29	0.03	0.06
14	48	0.06	0.00	0.07	0.09	0.24	0.27	0.09	0.04	0.03	0.19	0.01	0.05
14	49	0.09	0.00	0.20	0.06	0.24	0.26	0.09	0.04	0.03	0.20	0.00	0.06
14	50	0.08	0.00	0.18	0.01	0.24	0.24	0.08	0.01	0.02	0.21	0.00	0.05
14	51	0.05	0.00	0.11	0.00	0.24	0.22	0.07	0.04	0.04	0.09	0.01	0.06
14	52	0.06	0.00	0.20	0.01	0.23	0.20	0.09	0.00	0.04	0.11	0.00	0.06
14	53	0.00	0.00	0.18	0.00	0.23	0.18	0.13	0.04	0.02	0.13	0.01	0.06
14	54	0.04	0.00	0.19	0.00	0.22	0.16	0.17	0.04	0.04	0.21	0.00	0.06
14	55	0.00	0.00	0.19	0.00	0.20	0.14	0.23	0.03	0.08	0.12	0.00	0.06
14	56	0.00	0.00	0.16	0.00	0.18	0.12	0.31	0.04	0.11	0.00	0.03	0.06
14	57	0.03	0.00	0.18	0.00	0.17	0.10	0.39	0.04	0.10	0.19	0.00	0.06
14	58	0.07	0.00	0.16	0.00	0.15	0.08	0.48	0.04	0.04	0.15	0.00	0.05
14	59	0.00	0.01	0.13	0.00	0.13	0.06	0.57	0.01	0.04	0.17	0.00	0.05
14	60	0.04	0.03	0.13	0.00	0.11	0.05	0.66	0.02	0.04	0.20	0.00	0.06
14	61	0.04	0.00	0.19	0.00	0.10	0.03	0.70	0.03	0.06	0.19	0.00	0.06
14	62	0.00	0.00	0.02	0.00	0.08	0.02	0.72	0.03	0.08	0.07	0.00	0.06
14	63	0.08	0.00	0.18	0.00	0.06	0.01	0.73	0.04	0.10	0.15	0.00	0.06
14	64	0.10	0.00	0.20	0.00	0.06	0.01	0.69	0.04	0.16	0.19	0.06	0.06
14	65	0.04	0.01	0.19	0.00	0.07	0.02	0.65	0.04	0.25	0.45	0.09	0.06
14	66	0.26	0.00	0.19	0.00	0.07	0.02	0.59	0.04	0.35	0.61	0.21	0.06
14	67	0.28	0.11	0.06	0.01	0.07	0.01	0.53	0.00	0.45	0.86	0.30	0.06
14	68	0.27	0.02	0.19	0.00	0.07	0.03	0.49	0.00	0.51	0.89	0.38	0.05
14	69	0.34	0.21	0.18	0.04	0.08	0.03	0.48	0.02	0.51	1.15	0.38	0.06
14	70	0.42	0.24	0.19	0.06	0.09	0.03	0.47	0.00	0.49	1.18	0.43	0.06
14	71	0.42	0.18	0.15	0.07	0.10	0.04	0.46	0.01	0.46	1.19	0.51	0.08
14	72	0.18	0.25	0.19	0.07	0.10	0.04	0.45	0.00	0.36	1.07	0.47	0.09
14	73	0.17	0.14	0.20	0.03	0.12	0.05	0.42	0.00	0.20	1.02	0.35	0.09
14	74	0.01	0.06	0.14	0.01	0.13	0.05	0.40	0.00	0.15	1.00	0.30	0.16

14	75	0.03	0.08	0.18	0.00	0.14	0.06	0.37	0.00	0.10	0.87	0.24	0.21
14	76	0.07	0.00	0.20	0.00	0.16	0.06	0.34	0.04	0.06	0.89	0.20	0.26
14	77	0.04	0.01	0.19	0.00	0.16	0.07	0.31	0.00	0.03	0.55	0.20	0.29
14	78	0.00	0.00	0.14	0.00	0.16	0.07	0.28	0.00	0.01	0.64	0.14	0.36
14	79	0.02	0.00	0.18	0.00	0.18	0.06	0.24	0.00	0.01	0.78	0.15	0.34
14	80	0.00	0.00	0.18	0.00	0.19	0.05	0.22	0.01	0.00	0.53	0.14	0.38
14	81	0.02	0.00	0.18	0.00	0.19	0.04	0.19	0.03	0.00	0.47	0.14	0.42
14	82	0.00	0.00	0.14	0.00	0.19	0.04	0.21	0.00	0.01	0.51	0.09	0.43
14	83	0.00	0.01	0.14	0.00	0.20	0.03	0.24	0.00	0.00	0.57	0.04	0.43
14	84	0.00	0.00	0.15	0.00	0.19	0.02	0.25	0.00	0.01	0.48	0.02	0.46
14	85	0.00	0.00	0.20	0.00	0.19	0.01	0.28	0.03	0.01	0.40	0.02	0.48
14	86	0.00	0.01	0.19	0.00	0.21	0.01	0.29	0.00	0.00	0.19	0.00	0.51
14	87	0.00	0.00	0.19	0.00	0.20	0.01	0.29	0.00	0.01	0.26	0.00	0.55
14	88	0.00	0.00	0.18	0.00	0.20	0.00	0.28	0.00	0.01	0.20	0.00	0.61
14	89	0.00	0.02	0.18	0.00	0.19	0.00	0.26	0.03	0.01	0.24	0.00	0.66
14	90	0.03	0.00	0.19	0.00	0.19	0.00	0.23	0.00	0.01	0.11	0.01	0.71
14	91	0.03	0.00	0.17	0.02	0.18	0.00	0.20	0.04	0.01	0.23	0.00	0.72
14	92	0.03	0.03	0.16	0.02	0.16	0.00	0.17	0.04	0.00	0.22	0.00	0.71
14	93	0.04	0.03	0.11	0.04	0.16	0.00	0.14	0.00	0.01	0.31	0.00	0.68
14	94	0.05	0.01	0.19	0.06	0.15	0.00	0.12	0.04	0.01	0.24	0.00	0.65
14	95	0.01	0.00	0.19	0.02	0.13	0.00	0.10	0.00	0.00	0.26	0.00	0.65
14	96	0.00	0.00	0.06	0.00	0.11	0.00	0.08	0.03	0.00	0.20	0.01	0.66
14	97	0.00	0.00	0.18	0.00	0.09	0.00	0.06	0.04	0.01	0.16	0.01	0.66
14	98	0.00	0.00	0.16	0.00	0.07	0.00	0.05	0.00	0.01	0.19	0.01	0.60
14	99	0.00	0.00	0.18	0.00	0.05	0.00	0.03	0.04	0.01	0.20	0.01	0.51
14	100	0.00	0.00	0.20	0.00	0.04	0.00	0.03	0.00	0.01	0.07	0.00	0.44
14	101	0.02	0.00	0.20	0.00	0.02	0.00	0.02	0.02	0.00	0.15	0.01	0.39
14	102	0.00	0.00	0.19	0.00	0.01	0.00	0.03	0.00	0.00	0.07	0.01	0.35
14	103	0.00	0.00	0.19	0.00	0.01	0.01	0.03	0.03	0.01	0.03	0.00	0.31
14	104	0.00	0.00	0.19	0.00	0.00	0.01	0.03	0.01	0.00	0.00	0.01	0.28
14	105	0.00	0.02	0.19	0.00	0.00	0.01	0.03	0.04	0.01	0.00	0.01	0.24
14	106	0.00	0.01	0.19	0.00	0.00	0.01	0.02	0.04	0.01	0.00	0.01	0.22
14	107	0.01	0.00	0.17	0.00	0.00	0.03	0.02	0.03	0.00	0.00	0.01	0.22
14	108	0.00	0.00	0.17	0.00	0.00	0.03	0.02	0.00	0.01	0.00	0.01	0.21
14	109	0.00	0.01	0.19	0.00	0.00	0.04	0.02	0.04	0.01	0.00	0.01	0.19
14	110	0.00	0.02	0.15	0.00	0.00	0.05	0.02	0.01	0.01	0.00	0.00	0.18

14	111	0.00	0.03	0.04	0.00	0.00	0.05	0.02	0.00	0.01	0.00	0.01	0.20
14	112	0.00	0.02	0.06	0.02	0.00	0.05	0.02	0.00	0.00	0.00	0.01	0.20
14	113	0.00	0.01	0.06	0.01	0.00	0.06	0.02	0.00	0.01	0.00	0.01	0.20
14	114	0.02	0.00	0.18	0.06	0.00	0.06	0.02	0.00	0.01	0.00	0.01	0.19
14	115	0.00	0.01	0.09	0.07	0.01	0.05	0.03	0.01	0.01	0.00	0.01	0.19
14	116	0.00	0.02	0.07	0.07	0.00	0.06	0.04	0.00	0.01	0.00	0.01	0.22
14	117	0.00	0.02	0.07	0.06	0.00	0.07	0.05	0.00	0.01	0.00	0.01	0.21
14	118	0.00	0.00	0.11	0.06	0.00	0.08	0.05	0.00	0.01	0.00	0.01	0.19
14	119	0.05	0.00	0.19	0.03	0.00	0.10	0.07	0.00	0.01	0.00	0.01	0.18
14	120	0.00	0.00	0.11	0.05	0.00	0.11	0.09	0.00	0.01	0.00	0.01	0.18
14	121	0.00	0.00	0.04	0.03	0.00	0.13	0.11	0.00	0.01	0.00	0.01	0.16
14	122	0.00	0.00	0.11	0.02	0.00	0.14	0.12	0.00	0.00	0.00	0.01	0.15
14	123	0.00	0.00	0.14	0.04	0.00	0.15	0.14	0.00	0.00	0.00	0.01	0.14
14	124	0.00	0.00	0.10	0.00	0.00	0.00	0.16	0.00	0.01	0.00	0.01	0.14
14	125	0.02	0.00	0.08	0.00	0.00	0.00	0.17	0.00	0.01	0.00	0.01	0.14
15	0	0.00	0.00	0.00	0.00	0.00	0.00	0.00	0.00	0.00	0.00	0.00	0.00
15	1	0.00	0.00	0.00	0.00	0.00	0.00	0.00	0.00	0.00	0.00	0.00	0.00
15	2	0.00	0.00	0.00	0.00	0.00	0.00	0.00	0.00	0.00	0.00	0.00	0.00
15	3	0.00	0.00	0.00	0.00	0.00	0.00	0.00	0.00	0.00	0.00	0.00	0.00
15	4	0.00	0.00	0.38	0.00	0.00	0.00	0.11	0.00	0.01	0.00	0.01	0.06
15	5	0.00	0.00	0.38	0.00	0.00	0.00	0.11	0.00	0.01	0.00	0.01	0.06
15	6	0.02	0.03	0.43	0.09	0.06	0.20	0.09	0.00	0.01	0.02	0.01	0.06
15	7	0.00	0.02	0.40	0.08	0.05	0.15	0.08	0.00	0.01	0.00	0.01	0.05
15	8	0.01	0.00	0.39	0.05	0.07	0.11	0.07	0.00	0.01	0.02	0.01	0.05
15	9	0.00	0.00	0.37	0.04	0.07	0.07	0.06	0.03	0.01	0.00	0.01	0.05
15	10	0.00	0.00	0.32	0.00	0.08	0.04	0.05	0.00	0.01	0.00	0.01	0.03
15	11	0.00	0.00	0.29	0.00	0.07	0.02	0.04	0.00	0.01	0.00	0.01	0.03
15	12	0.00	0.00	0.14	0.00	0.08	0.00	0.03	0.02	0.01	0.00	0.01	0.02
15	13	0.00	0.01	0.20	0.00	0.08	0.00	0.03	0.04	0.00	0.00	0.01	0.02
15	14	0.00	0.03	0.22	0.00	0.08	0.00	0.02	0.03	0.01	0.00	0.01	0.02
15	15	0.00	0.01	0.08	0.00	0.08	0.00	0.02	0.04	0.01	0.00	0.01	0.02
15	16	0.00	0.00	0.23	0.00	0.08	0.00	0.02	0.00	0.00	0.00	0.01	0.03
15	17	0.02	0.02	0.22	0.00	0.07	0.00	0.01	0.00	0.00	0.00	0.01	0.03
15	18	0.00	0.00	0.20	0.00	0.07	0.00	0.01	0.04	0.01	0.00	0.01	0.03
15	19	0.00	0.00	0.19	0.00	0.07	0.00	0.02	0.04	0.01	0.00	0.01	0.03
15	20	0.00	0.03	0.05	0.00	0.06	0.00	0.02	0.01	0.00	0.00	0.01	0.03

15	21	0.01	0.00	0.20	0.00	0.06	0.00	0.02	0.00	0.01	0.00	0.01	0.03
15	22	0.00	0.00	0.19	0.00	0.05	0.00	0.02	0.00	0.01	0.02	0.01	0.04
15	23	0.00	0.00	0.05	0.00	0.05	0.00	0.02	0.03	0.00	0.02	0.01	0.04
15	24	0.00	0.00	0.19	0.00	0.03	0.00	0.02	0.00	0.01	0.02	0.01	0.08
15	25	0.00	0.00	0.19	0.00	0.01	0.00	0.02	0.04	0.00	0.06	0.01	0.10
15	26	0.00	0.00	0.03	0.00	0.00	0.00	0.02	0.00	0.01	0.12	0.01	0.14
15	27	0.00	0.06	0.20	0.00	0.00	0.00	0.02	0.00	0.00	0.17	0.01	0.19
15	28	0.00	0.00	0.17	0.00	0.00	0.00	0.02	0.01	0.01	0.17	0.01	0.22
15	29	0.00	0.06	0.20	0.00	0.00	0.00	0.01	0.03	0.01	0.15	0.01	0.22
15	30	0.00	0.08	0.22	0.02	0.00	0.00	0.02	0.00	0.00	0.22	0.01	0.26
15	31	0.00	0.12	0.22	0.01	0.00	0.00	0.02	0.07	0.01	0.22	0.00	0.28
15	32	0.00	0.17	0.13	0.01	0.00	0.00	0.02	0.07	0.01	0.25	0.00	0.31
15	33	0.00	0.00	0.25	0.01	0.00	0.00	0.02	0.17	0.00	0.26	0.00	0.32
15	34	0.00	0.07	0.18	0.01	0.00	0.00	0.02	0.31	0.00	0.24	0.01	0.31
15	35	0.00	0.24	0.23	0.00	0.00	0.00	0.01	0.35	0.00	0.24	0.02	0.30
15	36	0.00	0.00	0.20	0.00	0.00	0.00	0.02	0.48	0.00	0.20	0.02	0.26
15	37	0.00	0.00	0.25	0.00	0.00	0.00	0.02	0.57	0.01	0.20	0.00	0.22
15	38	0.00	0.26	0.26	0.00	0.00	0.00	0.02	0.59	0.00	0.14	0.00	0.19
15	39	0.00	0.23	0.23	0.00	0.00	0.00	0.02	0.75	0.01	0.11	0.04	0.15
15	40	0.00	0.15	0.23	0.00	0.00	0.00	0.02	0.76	0.01	0.01	0.07	0.12
15	41	0.02	0.10	0.21	0.01	0.00	0.00	0.01	0.78	0.00	0.05	0.10	0.11
15	42	0.00	0.36	0.19	0.01	0.00	0.00	0.01	0.68	0.01	0.06	0.14	0.08
15	43	0.00	0.53	0.24	0.00	0.01	0.00	0.01	0.84	0.00	0.03	0.20	0.07
15	44	0.00	0.24	0.19	0.00	0.01	0.00	0.02	0.86	0.01	0.03	0.25	0.05
15	45	0.00	0.22	0.23	0.02	0.01	0.00	0.02	0.94	0.00	0.02	0.28	0.06
15	46	0.00	0.35	0.09	0.03	0.01	0.00	0.02	1.00	0.01	0.03	0.41	0.06
15	47	0.00	0.74	0.23	0.05	0.01	0.00	0.01	1.02	0.00	0.01	0.52	0.07
15	48	0.00	0.87	0.26	0.06	0.01	0.00	0.02	0.94	0.01	0.00	0.57	0.07
15	49	0.01	0.95	0.27	0.10	0.01	0.00	0.02	0.93	0.00	0.02	0.68	0.08
15	50	0.00	1.00	0.29	0.12	0.01	0.00	0.02	0.93	0.01	0.07	0.80	0.09
15	51	0.00	1.02	0.32	0.15	0.01	0.00	0.02	0.92	0.00	0.06	0.85	0.11
15	52	0.00	1.08	0.23	0.19	0.01	0.00	0.02	0.94	0.00	0.09	0.92	0.11
15	53	0.00	1.15	0.31	0.20	0.00	0.00	0.02	0.89	0.01	0.07	0.98	0.10
15	54	0.04	1.15	0.29	0.25	0.01	0.00	0.02	0.93	0.01	0.07	1.01	0.11
15	55	0.17	1.22	0.10	0.30	0.01	0.00	0.02	0.92	0.01	0.01	1.05	0.11
15	56	0.09	1.17	0.29	0.34	0.00	0.00	0.02	0.86	0.00	0.05	1.06	0.10

15	57	0.00	1.14	0.16	0.39	0.00	0.01	0.02	0.84	0.00	0.02	1.02	0.09
15	58	0.04	1.07	0.26	0.39	0.00	0.00	0.02	0.86	0.01	0.00	1.07	0.08
15	59	0.00	0.99	0.10	0.41	0.00	0.01	0.02	0.94	0.01	0.03	1.07	0.07
15	60	0.02	0.88	0.03	0.44	0.00	0.01	0.02	0.80	0.01	0.04	1.07	0.06
15	61	0.02	0.83	0.10	0.57	0.00	0.01	0.02	0.70	0.01	0.00	1.04	0.04
15	62	0.16	0.70	0.15	0.71	0.00	0.01	0.02	0.63	0.00	0.00	0.93	0.03
15	63	0.00	0.62	0.07	0.88	0.00	0.01	0.02	0.52	0.00	0.00	0.91	0.02
15	64	0.10	0.59	0.03	1.04	0.00	0.01	0.02	0.31	0.00	0.00	0.86	0.02
15	65	0.25	0.48	0.06	1.20	0.00	0.01	0.02	0.39	0.00	0.00	0.79	0.02
15	66	0.34	0.47	0.33	1.65	0.00	0.01	0.02	0.31	0.01	0.00	0.69	0.04
15	67	0.46	0.35	0.50	1.95	0.00	0.01	0.02	0.28	0.01	0.04	0.65	0.06
15	68	0.62	0.41	0.57	2.10	0.00	0.00	0.01	0.26	0.00	0.02	0.59	0.09
15	69	0.71	0.42	0.67	2.25	0.00	0.00	0.02	0.31	0.01	0.01	0.65	0.10
15	70	0.69	0.40	0.79	2.42	0.00	0.00	0.02	0.30	0.01	0.14	0.58	0.17
15	71	0.83	0.38	0.91	2.56	0.00	0.00	0.02	0.25	0.00	0.16	0.55	0.22
15	72	0.81	0.36	1.05	2.71	0.00	0.00	0.02	0.20	0.00	0.22	0.51	0.26
15	73	0.91	0.33	1.19	2.81	0.00	0.00	0.02	0.13	0.01	0.32	0.42	0.34
15	74	0.97	0.31	1.31	2.94	0.00	0.00	0.02	0.13	0.00	0.35	0.40	0.41
15	75	0.88	0.31	1.43	3.02	0.00	0.00	0.02	0.08	0.01	0.42	0.36	0.48
15	76	1.02	0.23	1.49	3.08	0.00	0.00	0.02	0.06	0.00	0.49	0.35	0.55
15	77	1.04	0.26	1.59	3.15	0.00	0.00	0.02	0.01	0.01	0.57	0.27	0.62
15	78	1.06	0.31	1.50	3.29	0.00	0.00	0.02	0.02	0.01	0.61	0.32	0.66
15	79	1.09	0.31	1.71	3.43	0.00	0.00	0.03	0.00	0.01	0.67	0.30	0.72
15	80	1.05	0.24	1.58	3.30	0.00	0.00	0.03	0.00	0.00	0.72	0.22	0.74
15	81	0.94	0.00	1.71	3.13	0.00	0.00	0.03	0.00	0.00	0.73	0.17	0.74
15	82	0.53	0.09	1.72	2.90	0.00	0.00	0.03	0.02	0.00	0.74	0.13	0.79
15	83	0.66	0.00	1.71	2.66	0.00	0.00	0.03	0.00	0.00	0.80	0.05	0.82
15	84	0.46	0.00	1.70	2.40	0.00	0.00	0.03	0.00	0.01	0.83	0.03	0.85
15	85	0.42	0.00	1.65	2.14	0.00	0.00	0.04	0.00	0.00	0.81	0.00	0.83
15	86	0.33	0.00	1.55	1.87	0.00	0.00	0.03	0.00	0.00	0.79	0.00	0.76
15	87	0.33	0.00	1.55	1.78	0.00	0.00	0.04	0.00	0.00	0.77	0.00	0.78
15	88	0.04	0.00	1.50	1.69	0.00	0.00	0.04	0.00	0.01	0.74	0.00	0.75
15	89	0.00	0.00	1.40	1.57	0.00	0.00	0.05	0.00	0.00	0.70	0.00	0.72
15	90	0.00	0.00	1.48	1.52	0.00	0.00	0.05	0.00	0.00	0.67	0.00	0.67
15	91	0.21	0.00	1.55	1.40	0.00	0.00	0.06	0.00	0.00	0.64	0.00	0.65
15	92	0.10	0.02	1.57	1.25	0.00	0.00	0.06	0.02	0.01	0.59	0.00	0.61

15	93	0.07	0.00	1.44	1.06	0.00	0.00	0.06	0.03	0.00	0.47	0.00	0.50
15	94	0.07	0.02	1.19	0.87	0.00	0.00	0.06	0.00	0.00	0.33	0.00	0.35
15	95	0.00	0.00	0.81	0.65	0.00	0.00	0.05	0.01	0.00	0.18	0.00	0.22
15	96	0.03	0.00	0.77	0.66	0.00	0.00	0.05	0.07	0.01	0.18	0.00	0.20
15	97	0.08	0.03	0.47	0.65	0.00	0.00	0.04	0.08	0.00	0.14	0.00	0.18
15	98	0.02	0.03	0.30	0.56	0.00	0.00	0.04	0.15	0.01	0.11	0.01	0.17
15	99	0.00	0.02	0.25	0.53	0.00	0.00	0.03	0.17	0.00	0.15	0.00	0.17
15	100	0.00	0.00	0.19	0.44	0.00	0.00	0.02	0.23	0.00	0.13	0.01	0.16
15	101	0.00	0.00	0.09	0.45	0.00	0.00	0.02	0.30	0.01	0.11	0.01	0.15
15	102	0.02	0.00	0.05	0.45	0.00	0.00	0.02	0.37	0.01	0.11	0.01	0.14
15	103	0.00	0.00	0.08	0.43	0.00	0.00	0.02	0.45	0.00	0.07	0.01	0.12
15	104	0.00	0.00	0.05	0.40	0.00	0.00	0.02	0.47	0.01	0.09	0.01	0.12
15	105	0.00	0.00	0.11	0.36	0.00	0.00	0.02	0.59	0.00	0.03	0.00	0.09
15	106	0.00	0.02	0.04	0.36	0.00	0.00	0.02	0.63	0.01	0.00	0.01	0.09
15	107	0.00	0.00	0.00	0.31	0.00	0.00	0.02	0.67	0.01	0.00	0.01	0.08
15	108	0.00	0.02	0.09	0.27	0.00	0.00	0.02	0.70	0.00	0.00	0.00	0.06
15	109	0.00	0.00	0.20	0.20	0.00	0.00	0.02	0.70	0.01	0.00	0.01	0.05
15	110	0.00	0.00	0.19	0.19	0.00	0.00	0.01	0.75	0.01	0.00	0.01	0.04
15	111	0.00	0.03	0.01	0.17	0.00	0.00	0.02	0.76	0.00	0.00	0.01	0.03
15	112	0.00	0.00	0.02	0.20	0.00	0.00	0.02	0.76	0.01	0.00	0.00	0.01
15	113	0.00	0.02	0.04	0.25	0.00	0.00	0.02	0.68	0.01	0.00	0.01	0.00
15	114	0.00	0.00	0.03	0.31	0.00	0.00	0.02	0.50	0.01	0.00	0.01	0.02
15	115	0.00	0.02	0.02	0.36	0.00	0.00	0.02	0.47	0.01	0.00	0.01	0.02
15	116	0.00	0.00	0.18	0.39	0.00	0.00	0.01	0.34	0.01	0.00	0.01	0.03
15	117	0.00	0.00	0.19	0.39	0.00	0.00	0.02	0.25	0.01	0.00	0.01	0.02
15	118	0.00	0.00	0.18	0.36	0.00	0.00	0.02	0.11	0.01	0.00	0.01	0.02
15	119	0.00	0.00	0.19	0.31	0.00	0.00	0.02	0.06	0.01	0.00	0.01	0.02
15	120	0.00	0.00	0.06	0.28	0.00	0.00	0.02	0.03	0.01	0.00	0.01	0.02
15	121	0.00	0.00	0.18	0.24	0.00	0.00	0.02	0.00	0.00	0.00	0.01	0.01
15	122	0.00	0.00	0.18	0.23	0.00	0.00	0.01	0.04	0.00	0.00	0.01	0.02
15	123	0.00	0.00	0.06	0.21	0.00	0.01	0.02	0.04	0.01	0.00	0.00	0.02
15	124	0.00	0.01	0.08	0.24	0.00	0.01	0.02	0.04	0.01	0.00	0.01	0.02
15	125	0.01	0.00	0.15	0.25	0.00	0.01	0.02	0.03	0.01	0.00	0.01	0.02
15	126	0.01	0.02	0.19	0.25	0.00	0.02	0.02	0.03	0.01	0.00	0.01	0.02
15	127	0.01	0.02	0.19	0.17	0.00	0.02	0.02	0.03	0.01	0.00	0.01	0.02
15	128	0.00	0.00	0.19	0.15	0.00	0.00	0.02	0.00	0.00	0.00	0.01	0.02

15	129	0.00	0.00	0.18	0.16	0.00	0.00	0.02	0.01	0.00	0.00	0.01	0.02
15	130	0.02	0.02	0.20	0.14	0.00	0.00	0.02	0.03	0.00	0.00	0.01	0.02
15	131	0.00	0.00	0.19	0.11	0.00	0.00	0.02	0.01	0.00	0.00	0.01	0.02
15	132	0.00	0.00	0.18	0.09	0.00	0.00	0.02	0.03	0.00	0.00	0.01	0.02
15	133	0.00	0.02	0.18	0.07	0.00	0.00	0.02	0.04	0.00	0.00	0.01	0.02
15	134	0.00	0.01	0.20	0.07	0.00	0.00	0.02	0.02	0.00	0.00	0.01	0.02
16	1	0.00	0.00	0.23	0.00	0.00	0.00	0.02	0.19	0.00	0.00	0.01	0.15
16	2	0.00	0.02	0.26	0.00	0.00	0.00	0.02	0.27	0.00	0.25	0.01	0.21
16	3	0.00	0.00	0.24	0.00	0.00	0.00	0.02	0.30	0.00	0.25	0.01	0.21
16	4	0.00	0.00	0.27	0.00	0.00	0.00	0.01	0.29	0.00	0.24	0.01	0.21
16	5	0.00	0.00	0.25	0.00	0.00	0.00	0.02	0.32	0.00	0.21	0.01	0.20
16	6	0.00	0.03	0.28	0.00	0.00	0.00	0.02	0.36	0.00	0.25	0.01	0.20
16	7	0.00	0.01	0.35	0.00	0.00	0.00	0.02	0.37	0.00	0.17	0.01	0.21
16	8	0.00	0.02	0.38	0.00	0.00	0.00	0.02	0.39	0.00	0.18	0.01	0.21
16	9	0.00	0.00	0.26	0.00	0.00	0.00	0.02	0.39	0.00	0.15	0.00	0.23
16	10	0.00	0.02	0.40	0.00	0.00	0.00	0.02	0.37	0.00	0.19	0.00	0.24
16	11	0.00	0.00	0.56	0.00	0.00	0.00	0.02	0.33	0.00	0.19	0.01	0.25
16	12	0.00	0.00	0.49	0.00	0.00	0.00	0.02	0.32	0.00	0.12	0.01	0.26
16	13	0.00	0.00	0.63	0.00	0.00	0.00	0.02	0.29	0.00	0.10	0.01	0.26
16	14	0.00	0.00	0.69	0.00	0.38	0.47	0.02	0.24	0.01	0.12	0.01	0.26
16	15	0.00	0.00	0.47	0.00	0.38	0.48	0.02	0.16	0.01	0.05	0.01	0.21
16	16	0.00	0.00	0.44	0.00	0.38	0.50	0.02	0.16	0.01	0.04	0.00	0.17
16	17	0.00	0.00	0.62	0.00	0.37	0.52	0.02	0.11	0.01	0.00	0.00	0.16
16	18	0.00	0.00	0.63	0.00	0.36	0.52	0.02	0.08	0.00	0.00	0.01	0.14
16	19	0.00	0.00	0.62	0.00	0.35	0.51	0.01	0.02	0.01	0.00	0.01	0.12
16	20	0.00	0.00	0.63	0.00	0.33	0.51	0.02	0.03	0.01	0.00	0.01	0.10
16	21	0.00	0.00	0.61	0.00	0.31	0.51	0.02	0.03	0.01	0.00	0.01	0.08
16	22	0.00	0.00	0.58	0.00	0.29	0.50	0.02	0.03	0.01	0.01	0.00	0.06
16	23	0.00	0.01	0.56	0.00	0.27	0.48	0.01	0.03	0.01	0.00	0.01	0.06
16	24	0.00	0.00	0.54	0.00	0.25	0.47	0.02	0.00	0.00	0.00	0.01	0.06
16	25	0.00	0.00	0.56	0.00	0.24	0.47	0.02	0.00	0.01	0.00	0.01	0.05
16	26	0.00	0.00	0.59	0.00	0.23	0.50	0.02	0.04	0.01	0.01	0.00	0.05
16	27	0.00	0.00	0.62	0.00	0.22	0.54	0.01	0.00	0.01	0.02	0.00	0.05
16	28	0.01	0.00	0.63	0.00	0.20	0.57	0.02	0.00	0.01	0.02	0.00	0.04
16	29	0.00	0.00	0.53	0.00	0.19	0.59	0.01	0.04	0.00	0.03	0.00	0.05
16	30	0.00	0.00	0.60	0.00	0.19	0.58	0.02	0.04	0.01	0.04	0.00	0.05

16	31	0.00	0.00	0.39	0.00	0.20	0.53	0.02	0.04	0.01	0.00	0.00	0.05
16	32	0.00	0.02	0.45	0.00	0.20	0.48	0.02	0.01	0.01	0.00	0.00	0.05
16	33	0.02	0.00	0.40	0.00	0.21	0.43	0.02	0.04	0.01	0.00	0.00	0.05
16	34	0.00	0.00	0.29	0.00	0.21	0.38	0.02	0.04	0.01	0.00	0.00	0.05
16	35	0.00	0.00	0.14	0.00	0.21	0.32	0.02	0.00	0.01	0.00	0.00	0.05
16	36	0.00	0.00	0.27	0.00	0.21	0.27	0.02	0.00	0.01	0.00	0.00	0.05
16	37	0.00	0.01	0.18	0.00	0.21	0.22	0.01	0.04	0.01	0.00	0.01	0.01
16	38	0.00	0.00	0.20	0.00	0.21	0.17	0.02	0.04	0.01	0.00	0.02	0.05
16	39	0.00	0.00	0.20	0.00	0.19	0.14	0.02	0.02	0.00	0.00	0.00	0.05
16	40	0.00	0.00	0.20	0.00	0.17	0.11	0.02	0.00	0.00	0.00	0.02	0.05
16	41	0.00	0.00	0.12	0.00	0.15	0.09	0.02	0.00	0.01	0.00	0.00	0.01
16	42	0.00	0.00	0.17	0.00	0.13	0.07	0.02	0.00	0.00	0.00	0.00	0.04
16	43	0.00	0.00	0.19	0.00	0.11	0.06	0.02	0.00	0.01	0.00	0.00	0.05
16	44	0.00	0.00	0.19	0.00	0.10	0.05	0.02	0.00	0.01	0.00	0.02	0.05
16	45	0.01	0.00	0.06	0.00	0.08	0.05	0.02	0.04	0.00	0.00	0.00	0.05
16	46	0.00	0.00	0.20	0.00	0.07	0.05	0.02	0.00	0.00	0.00	0.00	0.03
16	47	0.00	0.00	0.19	0.00	0.05	0.05	0.02	0.04	0.00	0.00	0.01	0.05
16	48	0.00	0.00	0.20	0.00	0.04	0.04	0.02	0.00	0.01	0.00	0.01	0.02
16	49	0.00	0.00	0.19	0.00	0.03	0.04	0.02	0.03	0.01	0.00	0.00	0.06
16	50	0.00	0.00	0.20	0.00	0.02	0.03	0.02	0.03	0.00	0.00	0.00	0.06
16	51	0.02	0.00	0.20	0.00	0.01	0.01	0.02	0.04	0.01	0.00	0.01	0.05
16	52	0.00	0.03	0.16	0.00	0.00	0.00	0.02	0.00	0.00	0.00	0.01	0.06
16	53	0.01	0.01	0.16	0.00	0.00	0.00	0.02	0.00	0.00	0.00	0.01	0.06
16	54	0.00	0.00	0.11	0.00	0.00	0.00	0.02	0.00	0.00	0.00	0.01	0.06
16	55	0.00	0.00	0.06	0.00	0.00	0.00	0.02	0.00	0.00	0.00	0.01	0.06
16	56	0.00	0.02	0.19	0.00	0.00	0.00	0.02	0.00	0.00	0.00	0.01	0.06
16	57	0.00	0.00	0.16	0.00	0.00	0.00	0.02	0.04	0.00	0.00	0.01	0.06
16	58	0.00	0.00	0.18	0.00	0.00	0.00	0.02	0.01	0.00	0.00	0.01	0.06
16	59	0.00	0.01	0.06	0.00	0.00	0.00	0.02	0.04	0.01	0.00	0.01	0.06
16	60	0.00	0.00	0.18	0.00	0.00	0.00	0.02	0.04	0.01	0.00	0.01	0.06
16	61	0.00	0.00	0.18	0.00	0.00	0.00	0.02	0.00	0.00	0.00	0.01	0.06
16	62	0.00	0.00	0.19	0.00	0.00	0.00	0.02	0.00	0.01	0.00	0.01	0.05
16	63	0.00	0.02	0.19	0.00	0.00	0.00	0.02	0.04	0.01	0.00	0.00	0.06
16	64	0.01	0.01	0.18	0.00	0.00	0.00	0.03	0.04	0.00	0.00	0.01	0.06
16	65	0.00	0.01	0.19	0.00	0.00	0.00	0.05	0.04	0.01	0.00	0.01	0.06
16	66	0.01	0.00	0.19	0.01	0.00	0.00	0.07	0.04	0.01	0.00	0.01	0.05

16	67	0.00	0.00	0.19	0.00	0.00	0.00	0.09	0.00	0.01	0.00	0.01	0.05
16	68	0.00	0.00	0.13	0.01	0.00	0.00	0.13	0.04	0.01	0.00	0.00	0.05
16	69	0.00	0.00	0.07	0.01	0.00	0.00	0.15	0.04	0.01	0.00	0.01	0.05
16	70	0.00	0.02	0.00	0.03	0.00	0.00	0.16	0.04	0.01	0.00	0.01	0.06
16	71	0.00	0.03	0.08	0.04	0.00	0.00	0.16	0.00	0.01	0.00	0.01	0.04
16	72	0.00	0.00	0.16	0.07	0.00	0.00	0.18	0.01	0.01	0.00	0.00	0.06
16	73	0.00	0.00	0.07	0.08	0.00	0.00	0.19	0.00	0.01	0.00	0.01	0.03
16	74	0.00	0.02	0.01	0.10	0.00	0.00	0.20	0.00	0.01	0.00	0.01	0.06
16	75	0.01	0.03	0.03	0.07	0.00	0.00	0.22	0.01	0.01	0.00	0.01	0.06
16	76	0.00	0.00	0.00	0.04	0.00	0.00	0.23	0.00	0.01	0.00	0.00	0.05
16	77	0.00	0.00	0.09	0.05	0.00	0.00	0.25	0.03	0.00	0.00	0.01	0.06
16	78	0.00	0.02	0.03	0.03	0.00	0.00	0.27	0.03	0.01	0.00	0.01	0.06
16	79	0.01	0.01	0.13	0.03	0.00	0.00	0.29	0.04	0.01	0.00	0.01	0.05
16	80	0.00	0.02	0.03	0.01	0.00	0.00	0.31	0.00	0.01	0.00	0.01	0.06
16	81	0.02	0.00	0.02	0.04	0.00	0.00	0.30	0.02	0.00	0.00	0.01	0.06
16	82	0.00	0.01	0.01	0.06	0.00	0.00	0.28	0.04	0.01	0.00	0.01	0.06
16	83	0.00	0.02	0.00	0.06	0.00	0.00	0.26	0.05	0.01	0.01	0.01	0.06
16	84	0.00	0.00	0.09	0.07	0.00	0.00	0.24	0.03	0.01	0.00	0.01	0.06
16	85	0.00	0.00	0.08	0.08	0.00	0.00	0.21	0.02	0.00	0.00	0.01	0.05
16	86	0.00	0.00	0.03	0.06	0.00	0.00	0.18	0.00	0.00	0.00	0.01	0.06
16	87	0.00	0.00	0.06	0.05	0.00	0.00	0.16	0.02	0.00	0.00	0.01	0.06
16	88	0.00	0.01	0.04	0.06	0.00	0.00	0.17	0.00	0.00	0.00	0.01	0.05
16	89	0.00	0.00	0.07	0.04	0.00	0.00	0.19	0.00	0.01	0.00	0.01	0.06
16	90	0.00	0.02	0.18	0.05	0.00	0.01	0.21	0.00	0.00	0.00	0.01	0.06
16	91	0.02	0.03	0.07	0.06	0.00	0.02	0.23	0.04	0.01	0.00	0.01	0.06
16	92	0.00	0.00	0.20	0.07	0.00	0.03	0.24	0.00	0.01	0.00	0.01	0.06
16	93	0.00	0.00	0.00	0.07	0.00	0.04	0.23	0.00	0.01	0.00	0.01	0.06
16	94	0.02	0.00	0.03	0.09	0.00	0.05	0.21	0.03	0.01	0.00	0.01	0.00
16	95	0.01	0.00	0.20	0.07	0.00	0.06	0.19	0.04	0.00	0.00	0.01	0.05
16	96	0.02	0.01	0.21	0.10	0.00	0.07	0.16	0.04	0.00	0.00	0.01	0.06
16	97	0.00	0.00	0.01	0.10	0.00	0.08	0.15	0.04	0.00	0.00	0.00	0.06
16	98	0.00	0.04	0.20	0.09	0.00	0.10	0.13	0.04	0.00	0.00	0.01	0.06
16	99	0.00	0.05	0.03	0.15	0.00	0.10	0.13	0.03	0.00	0.00	0.01	0.06
16	100	0.00	0.04	0.18	0.18	0.00	0.10	0.14	0.00	0.01	0.00	0.01	0.06
16	101	0.00	0.00	0.04	0.16	0.00	0.10	0.15	0.00	0.01	0.00	0.01	0.05
16	102	0.00	0.08	0.02	0.13	0.00	0.10	0.15	0.00	0.01	0.00	0.00	0.06

16	103	0.00	0.00	0.20	0.13	0.00	0.09	0.16	0.00	0.01	0.01	0.01	0.05
16	104	0.00	0.00	0.00	0.13	0.00	0.09	0.16	0.00	0.01	0.00	0.01	0.06
16	105	0.00	0.00	0.00	0.14	0.00	0.08	0.17	0.00	0.01	0.00	0.00	0.05
16	106	0.00	0.04	0.01	0.14	0.00	0.07	0.17	0.00	0.01	0.00	0.00	0.05
16	107	0.00	0.05	0.08	0.16	0.00	0.06	0.17	0.00	0.00	0.00	0.00	0.05
16	108	0.00	0.08	0.18	0.20	0.00	0.05	0.17	0.00	0.01	0.00	0.00	0.05
16	109	0.00	0.13	0.11	0.23	0.00	0.04	0.17	0.00	0.00	0.00	0.01	0.05
16	110	0.00	0.12	0.19	0.27	0.00	0.04	0.14	0.00	0.00	0.00	0.00	0.06
16	111	0.03	0.00	0.11	0.30	0.00	0.04	0.11	0.00	0.01	0.00	0.00	0.05
16	112	0.01	0.15	0.18	0.34	0.00	0.04	0.09	0.00	0.00	0.00	0.00	0.06
16	113	0.03	0.16	0.19	0.37	0.00	0.04	0.08	0.00	0.01	0.00	0.00	0.05
16	114	0.02	0.20	0.19	0.36	0.00	0.04	0.06	0.00	0.01	0.00	0.00	0.06
16	115	0.03	0.16	0.14	0.35	0.00	0.04	0.04	0.01	0.01	0.00	0.00	0.06
16	116	0.09	0.11	0.05	0.33	0.00	0.04	0.03	0.04	0.01	0.00	0.02	0.06
16	117	0.00	0.17	0.14	0.30	0.00	0.03	0.02	0.00	0.01	0.00	0.04	0.06
16	118	0.05	0.15	0.13	0.22	0.00	0.03	0.02	0.03	0.01	0.00	0.02	0.06
16	119	0.04	0.03	0.14	0.15	0.00	0.03	0.02	0.03	0.01	0.00	0.03	0.06
16	120	0.00	0.18	0.19	0.09	0.00	0.03	0.02	0.04	0.00	0.00	0.03	0.06
16	121	0.00	0.14	0.18	0.05	0.00	0.03	0.02	0.00	0.00	0.00	0.04	0.06
16	122	0.00	0.06	0.05	0.02	0.00	0.02	0.02	0.00	0.01	0.00	0.05	0.05
16	123	0.00	0.10	0.20	0.00	0.00	0.02	0.02	0.00	0.01	0.00	0.05	0.06
16	124	0.00	0.12	0.19	0.00	0.00	0.00	0.02	0.00	0.01	0.00	0.06	0.05
16	125	0.00	0.17	0.19	0.00	0.00	0.00	0.02	0.00	0.02	0.00	0.06	0.06
16	126	0.02	0.00	0.19	0.00	0.00	0.00	0.02	0.00	0.02	0.00	0.08	0.06
16	127	0.00	0.00	0.19	0.00	0.00	0.00	0.02	0.02	0.00	0.00	0.08	0.06
16	128	0.00	0.10	0.20	0.00	0.00	0.00	0.01	0.00	0.00	0.00	0.08	0.05
17	1	0.00	0.00	0.19	0.00	0.00	0.00	0.02	0.00	0.01	0.00	0.01	0.05
17	2	0.00	0.00	0.19	0.00	0.00	0.00	0.02	0.00	0.00	0.00	0.01	0.06
17	3	0.01	0.00	0.17	0.00	0.00	0.00	0.02	0.04	0.01	0.00	0.01	0.05
17	4	0.00	0.02	0.13	0.00	0.00	0.00	0.02	0.00	0.01	0.00	0.00	0.06
17	5	0.00	0.00	0.19	0.00	0.00	0.00	0.02	0.00	0.00	0.00	0.01	0.06
17	6	0.00	0.00	0.18	0.00	0.00	0.00	0.02	0.00	0.01	0.00	0.01	0.06
17	7	0.00	0.00	0.17	0.00	0.00	0.00	0.02	0.00	0.01	0.00	0.00	0.06
17	8	0.00	0.00	0.18	0.00	0.00	0.00	0.02	0.04	0.01	0.00	0.01	0.06
17	9	0.00	0.00	0.17	0.00	0.00	0.00	0.02	0.00	0.01	0.00	0.00	0.06
17	10	0.00	0.00	0.19	0.00	0.00	0.00	0.02	0.00	0.01	0.00	0.01	0.06

17	11	0.00	0.00	0.18	0.00	0.00	0.00	0.02	0.00	0.00	0.00	0.01	0.06
17	12	0.00	0.03	0.04	0.00	0.00	0.00	0.02	0.01	0.00	0.00	0.01	0.06
17	13	0.00	0.01	0.19	0.00	0.00	0.00	0.02	0.03	0.01	0.00	0.01	0.06
17	14	0.00	0.00	0.16	0.00	0.00	0.00	0.02	0.00	0.01	0.00	0.01	0.06
17	15	0.00	0.00	0.11	0.00	0.00	0.00	0.02	0.04	0.01	0.00	0.01	0.06
17	16	0.01	0.00	0.19	0.00	0.00	0.01	0.02	0.03	0.01	0.00	0.01	0.06
17	17	0.00	0.00	0.19	0.00	0.00	0.02	0.02	0.05	0.01	0.00	0.01	0.06
17	18	0.00	0.00	0.17	0.00	0.00	0.02	0.02	0.01	0.01	0.00	0.01	0.06
17	19	0.00	0.00	0.12	0.00	0.00	0.02	0.02	0.11	0.00	0.00	0.01	0.06
17	20	0.00	0.00	0.10	0.00	0.00	0.02	0.02	0.12	0.01	0.00	0.01	0.06
17	21	0.00	0.00	0.18	0.00	0.00	0.03	0.02	0.19	0.01	0.00	0.01	0.06
17	22	0.00	0.02	0.19	0.00	0.00	0.02	0.02	0.19	0.01	0.00	0.01	0.06
17	23	0.00	0.00	0.19	0.00	0.00	0.03	0.02	0.17	0.01	0.00	0.00	0.06
17	24	0.00	0.00	0.19	0.00	0.00	0.03	0.02	0.17	0.01	0.00	0.01	0.06
17	25	0.00	0.00	0.18	0.00	0.00	0.03	0.02	0.17	0.00	0.00	0.00	0.06
17	26	0.00	0.02	0.18	0.00	0.00	0.02	0.02	0.22	0.00	0.00	0.00	0.06
17	27	0.00	0.01	0.20	0.00	0.00	0.02	0.02	0.30	0.00	0.00	0.00	0.05
17	28	0.00	0.00	0.11	0.00	0.00	0.02	0.02	0.38	0.01	0.00	0.00	0.06
17	29	0.00	0.00	0.19	0.00	0.00	0.02	0.02	0.44	0.01	0.00	0.00	0.06
17	30	0.00	0.00	0.18	0.00	0.00	0.02	0.02	0.37	0.01	0.00	0.00	0.06
17	31	0.00	0.01	0.15	0.00	0.00	0.02	0.02	0.33	0.01	0.00	0.00	0.06
17	32	0.00	0.00	0.20	0.00	0.00	0.01	0.02	0.31	0.01	0.00	0.00	0.06
17	33	0.00	0.00	0.19	0.00	0.00	0.00	0.02	0.28	0.01	0.00	0.01	0.06
17	34	0.00	0.00	0.17	0.00	0.00	0.00	0.02	0.26	0.00	0.00	0.01	0.06
17	35	0.00	0.03	0.20	0.00	0.00	0.00	0.02	0.23	0.00	0.00	0.01	0.06
17	36	0.00	0.00	0.17	0.00	0.00	0.00	0.02	0.21	0.00	0.00	0.00	0.06
17	37	0.00	0.01	0.08	0.00	0.00	0.00	0.02	0.16	0.01	0.00	0.00	0.06
17	38	0.00	0.08	0.18	0.00	0.00	0.00	0.02	0.14	0.01	0.00	0.00	0.06
17	39	0.00	0.10	0.19	0.00	0.00	0.00	0.02	0.09	0.00	0.00	0.01	0.05
17	40	0.00	0.12	0.18	0.00	0.00	0.00	0.02	0.05	0.00	0.00	0.00	0.06
17	41	0.01	0.14	0.18	0.00	0.00	0.00	0.02	0.04	0.00	0.00	0.00	0.06
17	42	0.00	0.12	0.11	0.00	0.00	0.00	0.02	0.04	0.00	0.00	0.01	0.06
17	43	0.00	0.14	0.11	0.00	0.00	0.00	0.01	0.00	0.00	0.00	0.00	0.06
17	44	0.00	0.03	0.07	0.00	0.00	0.00	0.02	0.03	0.00	0.00	0.01	0.05
17	45	0.00	0.04	0.18	0.00	0.00	0.00	0.02	0.04	0.00	0.01	0.00	0.06
17	46	0.00	0.17	0.19	0.00	0.00	0.00	0.01	0.04	0.00	0.00	0.01	0.06

17	47	0.00	0.23	0.19	0.00	0.00	0.00	0.02	0.00	0.00	0.00	0.00	0.06
17	48	0.00	0.35	0.19	0.00	0.00	0.00	0.02	0.00	0.00	0.00	0.01	0.06
17	49	0.00	0.48	0.20	0.00	0.00	0.00	0.01	0.00	0.01	0.00	0.03	0.06
17	50	0.00	0.56	0.19	0.00	0.00	0.00	0.02	0.04	0.00	0.00	0.00	0.06
17	51	0.00	0.50	0.17	0.00	0.00	0.00	0.02	0.04	0.01	0.00	0.06	0.05
17	52	0.00	0.47	0.19	0.00	0.00	0.00	0.01	0.01	0.00	0.00	0.01	0.05
17	53	0.00	0.39	0.19	0.01	0.00	0.00	0.02	0.04	0.01	0.00	0.00	0.06
17	54	0.00	0.33	0.19	0.02	0.00	0.00	0.02	0.04	0.01	0.00	0.05	0.05
17	55	0.00	0.43	0.04	0.05	0.00	0.00	0.02	0.00	0.01	0.00	0.00	0.06
17	56	0.00	0.41	0.20	0.08	0.00	0.00	0.02	0.00	0.01	0.00	0.00	0.06
17	57	0.00	0.42	0.20	0.09	0.00	0.00	0.01	0.04	0.01	0.00	0.03	0.05
17	58	0.00	0.41	0.12	0.11	0.00	0.00	0.01	0.03	0.01	0.00	0.00	0.05
17	59	0.00	0.36	0.12	0.11	0.00	0.00	0.01	0.04	0.01	0.00	0.03	0.06
17	60	0.00	0.44	0.18	0.09	0.00	0.00	0.02	0.05	0.01	0.00	0.03	0.05
17	61	0.00	0.35	0.18	0.07	0.00	0.00	0.02	0.06	0.01	0.00	0.01	0.06
17	62	0.00	0.50	0.19	0.07	0.00	0.00	0.02	0.10	0.00	0.00	0.00	0.06
17	63	0.00	0.51	0.18	0.06	0.00	0.00	0.02	0.07	0.01	0.00	0.03	0.06
17	64	0.01	0.39	0.13	0.06	0.00	0.00	0.02	0.09	0.00	0.00	0.00	0.06
17	65	0.00	0.46	0.20	0.07	0.00	0.00	0.02	0.00	0.00	0.00	0.01	0.05
17	66	0.00	0.40	0.19	0.06	0.00	0.00	0.02	0.08	0.00	0.00	0.02	0.06
17	67	0.00	0.40	0.16	0.08	0.00	0.00	0.02	0.10	0.01	0.00	0.00	0.06
17	68	0.00	0.49	0.19	0.08	0.00	0.00	0.02	0.13	0.01	0.00	0.00	0.05
17	69	0.00	0.39	0.20	0.07	0.00	0.00	0.02	0.14	0.01	0.00	0.00	0.06
17	70	0.00	0.48	0.05	0.08	0.00	0.00	0.02	0.07	0.00	0.00	0.01	0.06
17	71	0.00	0.40	0.08	0.07	0.00	0.00	0.02	0.14	0.00	0.00	0.00	0.05
17	72	0.00	0.27	0.14	0.05	0.00	0.00	0.01	0.13	0.01	0.00	0.01	0.06
17	73	0.00	0.30	0.20	0.06	0.00	0.00	0.02	0.12	0.01	0.00	0.00	0.05
17	74	0.00	0.21	0.19	0.06	0.00	0.00	0.01	0.06	0.01	0.00	0.00	0.06
17	75	0.00	0.12	0.20	0.09	0.00	0.00	0.01	0.11	0.01	0.00	0.00	0.05
17	76	0.00	0.00	0.19	0.10	0.00	0.00	0.02	0.10	0.01	0.00	0.00	0.05
17	77	0.01	0.15	0.06	0.12	0.00	0.00	0.02	0.09	0.00	0.00	0.01	0.06
17	78	0.00	0.00	0.17	0.15	0.00	0.00	0.02	0.10	0.01	0.00	0.01	0.06
17	79	0.00	0.00	0.19	0.12	0.00	0.00	0.02	0.07	0.00	0.00	0.01	0.06
17	80	0.00	0.07	0.19	0.19	0.00	0.00	0.02	0.08	0.01	0.00	0.01	0.06
17	81	0.01	0.04	0.19	0.21	0.00	0.00	0.01	0.08	0.01	0.00	0.01	0.06
17	82	0.01	0.08	0.19	0.24	0.00	0.00	0.02	0.07	0.00	0.00	0.01	0.06

17	83	0.00	0.10	0.18	0.28	0.00	0.00	0.02	0.04	0.01	0.00	0.01	0.06
17	84	0.00	0.00	0.18	0.31	0.00	0.00	0.01	0.04	0.00	0.00	0.01	0.06
17	85	0.00	0.19	0.04	0.35	0.00	0.00	0.02	0.04	0.01	0.00	0.00	0.05
17	86	0.00	0.21	0.17	0.39	0.00	0.00	0.02	0.04	0.00	0.00	0.00	0.06
17	87	0.00	0.22	0.18	0.42	0.00	0.00	0.02	0.00	0.01	0.00	0.00	0.06
17	88	0.01	0.28	0.00	0.47	0.00	0.01	0.02	0.00	0.00	0.00	0.01	0.06
17	89	0.00	0.16	0.18	0.53	0.00	0.02	0.02	0.00	0.00	0.00	0.02	0.06
17	90	0.00	0.44	0.05	0.61	0.00	0.03	0.02	0.00	0.01	0.00	0.00	0.06
17	91	0.00	0.54	0.05	0.64	0.00	0.04	0.02	0.00	0.02	0.00	0.06	0.04
17	92	0.00	0.59	0.19	0.73	0.00	0.05	0.02	0.04	0.04	0.00	0.10	0.06
17	93	0.00	0.66	0.18	0.78	0.00	0.06	0.02	0.00	0.07	0.00	0.13	0.06
17	94	0.00	0.70	0.19	0.81	0.00	0.05	0.02	0.01	0.07	0.00	0.17	0.05
17	95	0.00	0.70	0.19	0.90	0.00	0.04	0.02	0.04	0.07	0.01	0.20	0.06
17	96	0.00	0.71	0.20	0.94	0.00	0.03	0.02	0.03	0.06	0.00	0.22	0.06
17	97	0.00	0.75	0.19	0.95	0.00	0.03	0.02	0.03	0.05	0.00	0.29	0.06
17	98	0.03	0.75	0.18	0.97	0.00	0.02	0.02	0.00	0.05	0.02	0.32	0.06
17	99	0.05	0.72	0.19	0.96	0.00	0.01	0.02	0.00	0.05	0.08	0.38	0.06
17	100	0.00	0.67	0.19	0.97	0.00	0.01	0.02	0.04	0.05	0.02	0.39	0.06
17	101	0.00	0.55	0.18	0.97	0.00	0.01	0.02	0.03	0.05	0.00	0.37	0.06
17	102	0.00	0.16	0.18	0.92	0.00	0.00	0.02	0.03	0.04	0.02	0.33	0.05
17	103	0.00	0.22	0.06	0.89	0.00	0.00	0.02	0.00	0.04	0.02	0.29	0.06
17	104	0.00	0.00	0.03	0.78	0.00	0.00	0.02	0.00	0.04	0.00	0.31	0.06
17	105	0.03	0.13	0.13	0.64	0.00	0.01	0.02	0.02	0.05	0.00	0.35	0.06
17	106	0.00	0.33	0.19	0.67	0.00	0.01	0.02	0.00	0.07	0.00	0.41	0.06
17	107	0.00	0.34	0.10	0.73	0.00	0.02	0.02	0.00	0.08	0.13	0.39	0.06
17	108	0.09	0.53	0.07	0.81	0.00	0.04	0.02	0.02	0.10	0.15	0.43	0.06
17	109	0.11	0.67	0.19	0.90	0.00	0.05	0.02	0.00	0.13	0.00	0.55	0.06
17	110	0.00	0.66	0.08	0.98	0.00	0.07	0.02	0.00	0.18	0.23	0.62	0.06
17	111	0.17	0.86	0.09	1.04	0.00	0.08	0.02	0.02	0.23	0.26	0.60	0.06
17	112	0.22	1.01	0.05	1.10	0.00	0.09	0.02	0.04	0.29	0.18	0.63	0.06
17	113	0.27	1.06	0.18	1.15	0.00	0.10	0.02	0.04	0.35	0.23	0.85	0.06
17	114	0.30	1.26	0.17	1.18	0.00	0.12	0.02	0.04	0.41	0.44	0.89	0.06
17	115	0.32	1.37	0.08	1.07	0.00	0.14	0.02	0.03	0.47	0.46	0.96	0.06
17	116	0.30	1.39	0.18	0.97	0.00	0.14	0.02	0.04	0.50	0.44	0.90	0.06
17	117	0.22	1.23	0.17	0.90	0.00	0.12	0.02	0.04	0.45	0.51	0.89	0.06
17	118	0.21	1.17	0.18	0.82	0.00	0.11	0.02	0.04	0.40	0.28	0.71	0.06

17	119	0.05	0.97	0.19	0.75	0.00	0.10	0.02	0.03	0.36	0.41	0.62	0.06
17	120	0.01	0.92	0.18	0.63	0.00	0.09	0.02	0.04	0.31	0.22	0.61	0.06
17	121	0.00	0.83	0.03	0.50	0.00	0.07	0.02	0.00	0.26	0.22	0.51	0.06
17	122	0.00	0.69	0.04	0.49	0.00	0.06	0.02	0.00	0.22	0.17	0.32	0.06
17	123	0.00	0.63	0.19	0.48	0.00	0.05	0.02	0.04	0.18	0.16	0.38	0.06
17	124	0.00	0.58	0.19	0.48	0.00	0.04	0.02	0.04	0.14	0.03	0.34	0.06
17	125	0.00	0.43	0.18	0.48	0.00	0.03	0.02	0.00	0.10	0.06	0.26	0.06
17	126	0.00	0.39	0.17	0.47	0.00	0.03	0.02	0.02	0.07	0.03	0.24	0.06
17	127	0.00	0.09	0.18	0.46	0.00	0.02	0.02	0.04	0.04	0.09	0.19	0.06
17	128	0.00	0.22	0.04	0.46	0.00	0.02	0.01	0.04	0.03	0.03	0.14	0.06
17	129	0.00	0.21	0.07	0.42	0.00	0.01	0.02	0.04	0.00	0.01	0.11	0.06
17	130	0.00	0.01	0.19	0.42	0.00	0.01	0.02	0.04	0.00	0.00	0.09	0.06
17	131	0.02	0.13	0.18	0.31	0.00	0.01	0.02	0.03	0.02	0.05	0.07	0.06
17	132	0.00	0.00	0.07	0.20	0.00	0.02	0.02	0.04	0.02	0.02	0.05	0.06
17	133	0.00	0.09	0.19	0.20	0.00	0.02	0.01	0.04	0.03	0.05	0.00	0.06
17	134	0.00	0.09	0.07	0.17	0.00	0.03	0.02	0.04	0.04	0.02	0.06	0.06
17	135	0.00	0.11	0.16	0.16	0.00	0.03	0.02	0.04	0.05	0.00	0.05	0.06
17	136	0.00	0.00	0.19	0.14	0.00	0.04	0.02	0.00	0.06	0.00	0.08	0.06
17	137	0.00	0.07	0.19	0.13	0.00	0.03	0.00	0.04	0.07	0.00	0.07	0.06
18	1	0.02	0.00	0.06	0.00	0.03	0.00	0.02	0.07	0.01	0.00	0.01	0.06
18	2	0.00	0.00	0.06	0.00	0.02	0.00	0.02	0.14	0.01	0.00	0.01	0.05
18	3	0.00	0.02	0.19	0.00	0.02	0.00	0.02	0.14	0.01	0.00	0.01	0.06
18	4	0.00	0.00	0.18	0.00	0.02	0.00	0.02	0.04	0.01	0.00	0.01	0.06
18	5	0.00	0.02	0.16	0.00	0.02	0.00	0.02	0.04	0.01	0.00	0.01	0.05
18	6	0.00	0.00	0.19	0.00	0.01	0.00	0.02	0.08	0.01	0.00	0.01	0.05
18	7	0.00	0.03	0.20	0.00	0.01	0.00	0.02	0.15	0.01	0.00	0.01	0.06
18	8	0.00	0.00	0.19	0.00	0.01	0.00	0.03	0.17	0.01	0.00	0.01	0.06
18	9	0.00	0.02	0.19	0.00	0.01	0.00	0.02	0.16	0.01	0.00	0.01	0.05
18	10	0.00	0.02	0.20	0.00	0.01	0.00	0.02	0.18	0.01	0.00	0.01	0.06
18	11	0.06	0.00	0.15	0.05	0.02	0.00	0.02	0.22	0.01	0.00	0.00	0.06
18	12	0.04	0.02	0.19	0.09	0.04	0.00	0.02	0.24	0.01	0.00	0.01	0.04
18	13	0.00	0.00	0.19	0.14	0.04	0.00	0.02	0.31	0.01	0.00	0.01	0.04
18	14	0.00	0.00	0.20	0.19	0.06	0.00	0.02	0.27	0.01	0.00	0.01	0.07
18	15	0.00	0.00	0.19	0.25	0.08	0.00	0.02	0.24	0.01	0.00	0.00	0.07
18	16	0.00	0.00	0.18	0.33	0.09	0.00	0.02	0.24	0.01	0.00	0.01	0.08
18	17	0.00	0.00	0.12	0.40	0.11	0.00	0.02	0.13	0.01	0.00	0.01	0.09

18	18	0.00	0.00	0.18	0.53	0.11	0.00	0.02	0.13	0.00	0.00	0.01	0.10
18	19	0.00	0.00	0.19	0.63	0.11	0.00	0.02	0.06	0.00	0.00	0.01	0.12
18	20	0.05	0.00	0.15	0.73	0.11	0.00	0.00	0.02	0.01	0.00	0.01	0.14
18	21	0.00	0.02	0.23	0.86	0.11	0.00	0.02	0.11	0.01	0.00	0.01	0.18
18	22	0.18	0.01	0.31	0.95	0.11	0.00	0.02	0.05	0.01	0.00	0.01	0.22
18	23	0.25	0.01	0.42	1.02	0.11	0.00	0.02	0.13	0.01	0.00	0.01	0.27
18	24	0.18	0.00	0.67	1.02	0.11	0.00	0.02	0.12	0.01	0.00	0.01	0.33
18	25	0.09	0.00	0.72	1.04	0.11	0.00	0.02	0.11	0.01	0.00	0.01	0.37
18	26	0.11	0.00	0.58	1.03	0.10	0.00	0.02	0.13	0.01	0.00	0.01	0.43
18	27	0.26	0.01	0.79	1.02	0.10	0.00	0.02	0.14	0.01	0.00	0.01	0.47
18	28	0.00	0.00	0.81	0.96	0.10	0.00	0.02	0.11	0.01	0.01	0.01	0.52
18	29	0.10	0.00	0.76	0.96	0.10	0.00	0.02	0.11	0.00	0.00	0.01	0.54
18	30	0.00	0.00	0.75	0.90	0.09	0.00	0.02	0.01	0.01	0.00	0.00	0.54
18	31	0.06	0.00	0.73	0.83	0.09	0.00	0.02	0.06	0.01	0.00	0.00	0.54
18	32	0.00	0.00	0.70	0.76	0.08	0.00	0.02	0.00	0.01	0.00	0.00	0.53
18	33	0.08	0.00	0.70	0.70	0.07	0.00	0.02	0.04	0.00	0.00	0.00	0.53
18	34	0.05	0.00	0.65	0.65	0.07	0.00	0.02	0.04	0.00	0.00	0.01	0.54
18	35	0.02	0.02	0.67	0.58	0.06	0.00	0.02	0.00	0.00	0.00	0.01	0.55
18	36	0.05	0.00	0.72	0.51	0.05	0.00	0.02	0.05	0.01	0.00	0.01	0.55
18	37	0.01	0.01	0.67	0.45	0.04	0.00	0.02	0.00	0.01	0.00	0.01	0.54
18	38	0.00	0.00	0.70	0.44	0.04	0.00	0.02	0.00	0.00	0.00	0.01	0.54
18	39	0.01	0.00	0.64	0.49	0.05	0.00	0.02	0.00	0.01	0.00	0.01	0.58
18	40	0.00	0.00	0.63	0.53	0.05	0.00	0.02	0.04	0.00	0.00	0.01	0.60
18	41	0.01	0.00	0.60	0.58	0.06	0.00	0.02	0.02	0.01	0.00	0.01	0.62
18	42	0.00	0.00	0.57	0.61	0.07	0.00	0.02	0.05	0.01	0.00	0.01	0.64
18	43	0.01	0.00	0.58	0.64	0.07	0.00	0.02	0.04	0.01	0.00	0.01	0.64
18	44	0.02	0.03	0.44	0.62	0.07	0.00	0.01	0.05	0.01	0.00	0.01	0.65
18	45	0.04	0.00	0.45	0.61	0.08	0.00	0.02	0.08	0.01	0.00	0.01	0.66
18	46	0.04	0.02	0.30	0.60	0.08	0.00	0.00	0.10	0.00	0.00	0.01	0.65
18	47	0.01	0.03	0.51	0.57	0.09	0.00	0.02	0.09	0.00	0.00	0.01	0.65
18	48	0.00	0.02	0.48	0.51	0.10	0.00	0.02	0.10	0.01	0.00	0.00	0.66
18	49	0.00	0.00	0.63	0.47	0.10	0.00	0.00	0.10	0.00	0.00	0.01	0.67
18	50	0.00	0.00	0.63	0.40	0.10	0.00	0.02	0.12	0.00	0.00	0.00	0.67
18	51	0.00	0.00	0.67	0.36	0.11	0.00	0.02	0.10	0.00	0.01	0.01	0.67
18	52	0.00	0.02	0.73	0.35	0.12	0.00	0.02	0.13	0.00	0.00	0.00	0.68
18	53	0.00	0.00	0.77	0.34	0.13	0.00	0.02	0.11	0.01	0.00	0.01	0.65

18	54	0.00	0.03	0.62	0.30	0.14	0.03	0.02	0.13	0.00	0.00	0.01	0.59
18	55	0.00	0.00	0.66	0.29	0.16	0.05	0.02	0.16	0.01	0.00	0.01	0.51
18	56	0.00	0.03	0.57	0.26	0.16	0.07	0.02	0.17	0.01	0.00	0.01	0.41
18	57	0.00	0.00	0.53	0.22	0.18	0.09	0.02	0.19	0.01	0.00	0.00	0.33
18	58	0.00	0.00	0.44	0.26	0.18	0.11	0.01	0.22	0.01	0.00	0.01	0.23
18	59	0.00	0.00	0.41	0.31	0.17	0.09	0.02	0.25	0.01	0.00	0.01	0.19
18	60	0.00	0.00	0.37	0.36	0.16	0.09	0.02	0.24	0.01	0.00	0.01	0.18
18	61	0.00	0.03	0.33	0.41	0.15	0.08	0.02	0.26	0.00	0.00	0.00	0.17
18	62	0.03	0.00	0.34	0.45	0.13	0.07	0.01	0.22	0.01	0.01	0.01	0.19
18	63	0.02	0.13	0.34	0.46	0.12	0.07	0.01	0.21	0.01	0.00	0.01	0.22
18	64	0.07	0.11	0.35	0.49	0.11	0.05	0.02	0.19	0.01	0.04	0.00	0.25
18	65	0.09	0.00	0.34	0.53	0.09	0.04	0.02	0.16	0.01	0.01	0.00	0.28
18	66	0.06	0.00	0.35	0.55	0.08	0.03	0.02	0.09	0.01	0.00	0.01	0.31
18	67	0.06	0.09	0.16	0.56	0.07	0.03	0.02	0.02	0.01	0.02	0.02	0.35
18	68	0.00	0.29	0.19	0.57	0.06	0.01	0.01	0.03	0.00	0.02	0.00	0.37
18	69	0.08	0.26	0.23	0.57	0.04	0.01	0.01	0.02	0.00	0.00	0.00	0.42
18	70	0.05	0.23	0.36	0.62	0.03	0.01	0.02	0.05	0.01	0.00	0.04	0.45
18	71	0.07	0.48	0.36	0.64	0.03	0.00	0.02	0.06	0.00	0.04	0.04	0.47
18	72	0.12	0.43	0.36	0.65	0.02	0.00	0.02	0.00	0.01	0.06	0.07	0.49
18	73	0.04	0.51	0.36	0.65	0.01	0.00	0.02	0.00	0.01	0.00	0.06	0.49
18	74	0.13	0.41	0.38	0.71	0.01	0.00	0.02	0.00	0.01	0.00	0.02	0.46
18	75	0.12	0.44	0.26	0.71	0.00	0.00	0.02	0.00	0.04	0.01	0.09	0.47
18	76	0.06	0.41	0.30	0.57	0.00	0.00	0.02	0.00	0.05	0.00	0.07	0.48
18	77	0.05	0.56	0.40	0.44	0.00	0.00	0.02	0.02	0.05	0.05	0.04	0.49
18	78	0.04	0.45	0.57	0.31	0.00	0.00	0.02	0.01	0.06	0.04	0.06	0.48
18	79	0.00	0.27	0.59	0.32	0.00	0.00	0.02	0.04	0.07	0.02	0.04	0.47
18	80	0.04	0.40	0.64	0.37	0.00	0.00	0.02	0.04	0.06	0.00	0.03	0.44
18	81	0.07	0.08	0.67	0.39	0.00	0.00	0.02	0.03	0.05	0.00	0.01	0.44
18	82	0.03	0.22	0.63	0.39	0.00	0.00	0.04	0.02	0.03	0.00	0.00	0.42
18	83	0.08	0.21	0.71	0.41	0.00	0.00	0.06	0.04	0.03	0.00	0.00	0.42
18	84	0.00	0.19	0.73	0.39	0.00	0.00	0.10	0.04	0.03	0.00	0.00	0.43
18	85	0.17	0.19	0.81	0.34	0.00	0.00	0.16	0.04	0.03	0.00	0.00	0.43
18	86	0.17	0.00	0.87	0.36	0.00	0.00	0.25	0.04	0.03	0.00	0.01	0.45
18	87	0.04	0.10	0.88	0.33	0.00	0.00	0.37	0.00	0.02	0.00	0.01	0.48
18	88	0.15	0.07	0.96	0.31	0.00	0.01	0.50	0.01	0.01	0.00	0.01	0.50
18	89	0.19	0.00	1.03	0.30	0.00	0.01	0.61	0.01	0.01	0.00	0.01	0.50

18	90	0.19	0.20	1.11	0.30	0.00	0.02	0.71	0.01	0.00	0.00	0.01	0.52
18	91	0.30	0.24	1.18	0.31	0.00	0.02	0.82	0.04	0.00	0.03	0.01	0.54
18	92	0.31	0.09	1.19	0.29	0.00	0.03	0.90	0.04	0.01	0.00	0.01	0.55
18	93	0.04	0.09	1.07	0.19	0.00	0.03	0.81	0.00	0.00	0.00	0.00	0.50
18	94	0.18	0.11	0.95	0.15	0.00	0.04	0.70	0.01	0.01	0.02	0.01	0.43
18	95	0.20	0.00	0.84	0.12	0.00	0.05	0.59	0.00	0.01	0.03	0.01	0.40
18	96	0.23	0.00	0.77	0.16	0.00	0.06	0.49	0.03	0.01	0.03	0.01	0.42
18	97	0.22	0.06	0.79	0.20	0.00	0.07	0.38	0.04	0.01	0.04	0.01	0.44
18	98	0.19	0.09	0.83	0.23	0.00	0.06	0.31	0.05	0.00	0.04	0.01	0.47
18	99	0.26	0.08	0.81	0.25	0.00	0.05	0.28	0.09	0.00	0.05	0.01	0.52
18	100	0.09	0.00	1.16	0.34	0.00	0.05	0.28	0.10	0.00	0.13	0.01	0.64
18	101	0.31	0.07	1.29	0.46	0.00	0.03	0.28	0.12	0.00	0.18	0.00	0.74
18	102	0.26	0.04	1.31	0.49	0.00	0.04	0.29	0.08	0.00	0.21	0.01	0.84
18	103	0.26	0.00	1.33	0.47	0.00	0.03	0.29	0.11	0.01	0.20	0.01	0.92
18	104	0.10	0.01	1.21	0.49	0.00	0.03	0.29	0.12	0.00	0.33	0.01	0.93
18	105	0.23	0.00	1.16	0.48	0.00	0.02	0.28	0.10	0.01	0.26	0.01	0.94
18	106	0.21	0.01	1.19	0.46	0.00	0.01	0.28	0.19	0.00	0.30	0.01	0.94
18	107	0.18	0.00	1.17	0.44	0.00	0.01	0.27	0.18	0.01	0.28	0.01	0.94
18	108	0.14	0.00	1.09	0.42	0.00	0.01	0.26	0.13	0.01	0.28	0.01	0.93
18	109	0.10	0.00	1.06	0.36	0.00	0.01	0.26	0.11	0.01	0.17	0.01	0.93
18	110	0.16	0.02	0.94	0.36	0.00	0.02	0.26	0.15	0.00	0.37	0.01	0.91
18	111	0.08	0.00	0.77	0.33	0.00	0.02	0.26	0.14	0.01	0.40	0.00	0.92
18	112	0.00	0.00	0.70	0.29	0.00	0.03	0.25	0.11	0.01	0.45	0.01	0.94
18	113	0.10	0.00	0.58	0.27	0.00	0.04	0.25	0.08	0.01	0.43	0.01	0.98
18	114	0.13	0.01	0.54	0.19	0.00	0.05	0.23	0.09	0.00	0.53	0.00	1.06
18	115	0.10	0.00	0.42	0.17	0.00	0.06	0.22	0.11	0.01	0.54	0.01	1.08
18	116	0.00	0.00	0.27	0.15	0.00	0.08	0.21	0.13	0.00	0.59	0.01	1.14
18	117	0.04	0.00	0.22	0.17	0.00	0.07	0.18	0.16	0.01	0.49	0.01	1.18
18	118	0.17	0.00	0.31	0.19	0.00	0.06	0.16	0.14	0.01	0.59	0.00	1.28
18	119	0.15	0.00	0.29	0.21	0.00	0.08	0.14	0.13	0.00	0.59	0.00	1.37
18	120	0.00	0.00	0.28	0.22	0.00	0.09	0.12	0.13	0.01	0.62	0.00	1.43
18	121	0.22	0.00	0.25	0.24	0.00	0.11	0.10	0.24	0.00	0.72	0.00	1.45
18	122	0.26	0.00	0.17	0.25	0.00	0.12	0.09	0.26	0.00	0.73	0.00	1.45
19	1	0.19	0.00	0.19	0.00	0.00	0.00	0.00	0.45	0.07	0.00	0.31	0.06
19	2	0.00	0.13	0.08	0.00	0.00	0.00	0.00	0.52	0.07	0.00	0.26	0.06
19	3	0.17	0.04	0.19	0.00	0.00	0.00	0.00	0.53	0.08	0.00	0.30	0.06

19	4	0.20	0.14	0.10	0.00	0.00	0.00	0.00	0.57	0.06	0.00	0.34	0.06
19	5	0.13	0.15	0.19	0.00	0.00	0.00	0.00	0.65	0.07	0.00	0.36	0.06
19	6	0.20	0.13	0.17	0.00	0.00	0.00	0.00	0.71	0.07	0.00	0.31	0.06
19	7	0.18	0.14	0.10	0.00	0.00	0.00	0.00	0.71	0.07	0.00	0.37	0.06
19	8	0.19	0.15	0.18	0.00	0.00	0.00	0.00	0.75	0.07	0.01	0.37	0.06
19	9	0.01	0.00	0.08	0.00	0.00	0.00	0.00	0.87	0.08	0.00	0.36	0.06
19	10	0.08	0.11	0.19	0.00	0.00	0.00	0.02	0.93	0.07	0.00	0.34	0.06
19	11	0.00	0.09	0.19	0.00	0.20	0.00	0.02	0.95	0.08	0.00	0.35	0.06
19	12	0.00	0.14	0.19	0.00	0.20	0.01	0.02	1.06	0.08	0.00	0.32	0.06
19	13	0.00	0.12	0.08	0.00	0.19	0.02	0.02	1.07	0.09	0.00	0.30	0.06
19	14	0.11	0.13	0.08	0.00	0.19	0.04	0.02	1.09	0.10	0.00	0.35	0.06
19	15	0.00	0.12	0.05	0.00	0.17	0.05	0.02	1.11	0.11	0.00	0.35	0.06
19	16	0.00	0.10	0.20	0.00	0.16	0.07	0.02	1.21	0.11	0.00	0.32	0.05
19	17	0.00	0.02	0.19	0.00	0.14	0.05	0.02	1.25	0.12	0.00	0.31	0.06
19	18	0.00	0.09	0.03	0.00	0.12	0.02	0.02	1.28	0.15	0.00	0.34	0.06
19	19	0.00	0.12	0.08	0.00	0.09	0.02	0.02	1.26	0.18	0.00	0.37	0.06
19	20	0.00	0.02	0.19	0.00	0.08	0.01	0.02	1.23	0.20	0.00	0.39	0.06
19	21	0.03	0.17	0.18	0.00	0.05	0.00	0.02	1.18	0.23	0.00	0.43	0.06
19	22	0.01	0.29	0.13	0.00	0.04	0.00	0.02	1.11	0.26	0.00	0.46	0.05
19	23	0.00	0.22	0.17	0.00	0.02	0.00	0.02	0.99	0.29	0.00	0.45	0.06
19	24	0.00	0.29	0.16	0.00	0.01	0.00	0.02	0.96	0.31	0.00	0.40	0.06
19	25	0.00	0.34	0.19	0.00	0.01	0.00	0.02	0.85	0.34	0.00	0.53	0.06
19	26	0.00	0.52	0.19	0.00	0.01	0.00	0.02	0.75	0.36	0.00	0.52	0.06
19	27	0.00	0.47	0.19	0.00	0.01	0.00	0.02	0.72	0.38	0.00	0.48	0.06
19	28	0.00	0.05	0.08	0.00	0.01	0.00	0.02	0.73	0.40	0.00	0.45	0.05
19	29	0.02	0.08	0.18	0.00	0.01	0.00	0.02	0.66	0.41	0.00	0.37	0.06
19	30	0.00	0.23	0.19	0.00	0.01	0.00	0.02	0.56	0.41	0.00	0.31	0.06
19	31	0.00	0.26	0.19	0.00	0.01	0.00	0.02	0.43	0.41	0.00	0.27	0.06
19	32	0.00	0.30	0.19	0.00	0.01	0.00	0.02	0.32	0.39	0.00	0.25	0.05
19	33	0.00	0.30	0.05	0.00	0.00	0.00	0.02	0.36	0.40	0.00	0.14	0.06
19	34	0.00	0.28	0.19	0.00	0.00	0.00	0.02	0.28	0.42	0.02	0.15	0.06
19	35	0.01	0.18	0.16	0.00	0.00	0.00	0.02	0.29	0.44	0.00	0.18	0.06
19	36	0.00	0.23	0.20	0.00	0.00	0.00	0.02	0.33	0.46	0.00	0.17	0.06
19	37	0.00	0.20	0.20	0.00	0.00	0.00	0.02	0.25	0.44	0.01	0.02	0.06
19	38	0.00	0.09	0.19	0.00	0.00	0.00	0.03	0.24	0.42	0.00	0.08	0.06
19	39	0.00	0.00	0.17	0.00	0.00	0.00	0.03	0.33	0.40	0.00	0.04	0.06

19	40	0.00	0.05	0.18	0.00	0.00	0.00	0.02	0.22	0.37	0.00	0.03	0.05
19	41	0.00	0.01	0.19	0.00	0.00	0.00	0.02	0.34	0.34	0.00	0.00	0.06
19	42	0.00	0.00	0.14	0.00	0.00	0.00	0.02	0.27	0.31	0.00	0.00	0.06
19	43	0.00	0.00	0.17	0.00	0.00	0.00	0.01	0.26	0.28	0.00	0.01	0.06
19	44	0.00	0.01	0.19	0.00	0.00	0.00	0.02	0.29	0.25	0.00	0.01	0.06
19	45	0.00	0.00	0.20	0.00	0.00	0.00	0.02	0.26	0.22	0.00	0.01	0.06
19	46	0.01	0.00	0.11	0.00	0.00	0.00	0.02	0.24	0.19	0.00	0.01	0.06
19	47	0.00	0.00	0.07	0.00	0.00	0.00	0.02	0.19	0.16	0.00	0.01	0.05
19	48	0.00	0.02	0.18	0.00	0.00	0.00	0.02	0.15	0.14	0.00	0.01	0.06
19	49	0.00	0.00	0.00	0.00	0.00	0.00	0.02	0.11	0.11	0.01	0.01	0.06
19	50	0.00	0.00	0.17	0.00	0.00	0.00	0.02	0.08	0.09	0.00	0.00	0.06
19	51	0.01	0.00	0.19	0.00	0.00	0.00	0.02	0.03	0.07	0.00	0.01	0.06
19	52	0.00	0.00	0.15	0.00	0.00	0.00	0.02	0.03	0.06	0.00	0.00	0.06
19	53	0.00	0.02	0.06	0.00	0.00	0.00	0.02	0.00	0.04	0.00	0.01	0.06
19	54	0.00	0.02	0.02	0.00	0.00	0.00	0.02	0.06	0.03	0.00	0.01	0.06
19	55	0.00	0.02	0.16	0.00	0.00	0.00	0.02	0.04	0.02	0.00	0.01	0.06
19	56	0.00	0.00	0.17	0.00	0.00	0.00	0.02	0.05	0.01	0.00	0.01	0.06
19	57	0.00	0.00	0.15	0.00	0.00	0.00	0.02	0.00	0.01	0.00	0.01	0.06
19	58	0.00	0.00	0.17	0.00	0.00	0.00	0.01	0.08	0.01	0.00	0.01	0.06
19	59	0.00	0.00	0.03	0.00	0.00	0.00	0.02	0.00	0.01	0.00	0.01	0.06
19	60	0.00	0.00	0.18	0.00	0.00	0.00	0.01	0.09	0.00	0.00	0.01	0.06
19	61	0.02	0.02	0.15	0.00	0.00	0.00	0.02	0.09	0.00	0.00	0.01	0.05
19	62	0.00	0.03	0.19	0.00	0.00	0.00	0.02	0.00	0.01	0.00	0.01	0.06
19	63	0.00	0.02	0.20	0.00	0.00	0.00	0.02	0.00	0.01	0.00	0.01	0.06
19	64	0.00	0.01	0.19	0.00	0.00	0.00	0.02	0.02	0.00	0.00	0.01	0.06
19	65	0.00	0.01	0.19	0.00	0.00	0.00	0.02	0.02	0.01	0.00	0.01	0.06
19	66	0.00	0.00	0.12	0.00	0.00	0.00	0.02	0.03	0.01	0.00	0.01	0.06
19	67	0.00	0.00	0.19	0.01	0.00	0.00	0.02	0.08	0.00	0.00	0.01	0.05
19	68	0.00	0.00	0.03	0.02	0.00	0.00	0.03	0.09	0.01	0.00	0.00	0.06
19	69	0.00	0.02	0.19	0.03	0.00	0.00	0.05	0.10	0.01	0.00	0.01	0.06
19	70	0.00	0.02	0.00	0.03	0.00	0.00	0.05	0.07	0.01	0.00	0.01	0.06
19	71	0.00	0.00	0.19	0.02	0.00	0.00	0.05	0.07	0.01	0.00	0.01	0.06
19	72	0.00	0.02	0.18	0.00	0.00	0.00	0.04	0.05	0.01	0.00	0.01	0.06
19	73	0.00	0.00	0.06	0.00	0.00	0.00	0.04	0.15	0.00	0.00	0.00	0.06
19	74	0.00	0.01	0.00	0.00	0.00	0.00	0.04	0.17	0.00	0.00	0.01	0.06
19	75	0.00	0.00	0.19	0.00	0.00	0.00	0.03	0.22	0.00	0.01	0.01	0.06

19	76	0.00	0.00	0.20	0.00	0.00	0.00	0.04	0.33	0.01	0.00	0.01	0.05
19	77	0.01	0.03	0.19	0.00	0.00	0.00	0.04	0.27	0.01	0.00	0.01	0.05
19	78	0.00	0.01	0.15	0.00	0.00	0.00	0.04	0.21	0.00	0.00	0.01	0.06
19	79	0.00	0.00	0.19	0.00	0.00	0.00	0.04	0.17	0.00	0.00	0.01	0.05
19	80	0.02	0.03	0.20	0.00	0.00	0.00	0.04	0.14	0.01	0.00	0.01	0.06
19	81	0.00	0.00	0.19	0.00	0.00	0.00	0.03	0.05	0.01	0.00	0.00	0.06
19	82	0.00	0.00	0.15	0.00	0.00	0.00	0.04	0.07	0.00	0.00	0.01	0.06
19	83	0.00	0.00	0.19	0.00	0.00	0.00	0.04	0.04	0.01	0.00	0.01	0.06
19	84	0.00	0.00	0.19	0.00	0.00	0.00	0.04	0.09	0.00	0.00	0.01	0.06
19	85	0.00	0.00	0.05	0.00	0.00	0.00	0.04	0.08	0.01	0.01	0.00	0.06
19	86	0.00	0.00	0.20	0.00	0.00	0.00	0.04	0.09	0.00	0.00	0.00	0.06
19	87	0.00	0.00	0.18	0.00	0.00	0.00	0.04	0.10	0.00	0.00	0.00	0.06
19	88	0.00	0.03	0.19	0.00	0.00	0.00	0.02	0.10	0.01	0.10	0.00	0.06
19	89	0.00	0.00	0.18	0.00	0.00	0.00	0.02	0.08	0.01	0.05	0.00	0.06
19	90	0.00	0.00	0.19	0.00	0.00	0.00	0.02	0.08	0.01	0.15	0.00	0.05
19	91	0.00	0.00	0.19	0.00	0.00	0.00	0.02	0.08	0.00	0.16	0.00	0.04
19	92	0.01	0.00	0.19	0.00	0.00	0.00	0.01	0.00	0.00	0.11	0.00	0.05
19	93	0.00	0.00	0.09	0.00	0.00	0.00	0.03	0.00	0.00	0.21	0.00	0.06
19	94	0.02	0.00	0.17	0.00	0.00	0.00	0.02	0.02	0.01	0.27	0.00	0.07
19	95	0.00	0.00	0.19	0.00	0.00	0.00	0.02	0.00	0.00	0.30	0.01	0.07
19	96	0.00	0.01	0.09	0.00	0.00	0.00	0.01	0.03	0.01	0.22	0.00	0.08
19	97	0.00	0.00	0.17	0.00	0.00	0.00	0.02	0.04	0.00	0.21	0.00	0.08
19	98	0.00	0.00	0.20	0.00	0.00	0.00	0.02	0.00	0.00	0.16	0.00	0.07
19	99	0.00	0.00	0.19	0.00	0.00	0.00	0.02	0.00	0.00	0.14	0.01	0.07
19	100	0.00	0.02	0.17	0.00	0.00	0.00	0.02	0.04	0.01	0.11	0.01	0.07
19	101	0.00	0.00	0.17	0.00	0.00	0.00	0.02	0.04	0.01	0.08	0.01	0.05
19	102	0.00	0.02	0.19	0.00	0.00	0.00	0.02	0.03	0.01	0.05	0.01	0.05
19	103	0.00	0.00	0.19	0.00	0.00	0.00	0.02	0.01	0.01	0.04	0.01	0.06
19	104	0.00	0.01	0.19	0.00	0.00	0.00	0.02	0.03	0.01	0.00	0.01	0.05
19	105	0.00	0.00	0.20	0.00	0.00	0.00	0.02	0.04	0.01	0.00	0.01	0.05
19	106	0.00	0.00	0.16	0.00	0.00	0.00	0.02	0.02	0.01	0.00	0.01	0.06
19	107	0.00	0.00	0.20	0.00	0.00	0.00	0.02	0.02	0.01	0.00	0.01	0.05
19	108	0.00	0.00	0.19	0.00	0.00	0.00	0.02	0.01	0.01	0.00	0.01	0.06
19	109	0.00	0.01	0.05	0.00	0.00	0.00	0.02	0.04	0.00	0.00	0.01	0.06
19	110	0.00	0.00	0.18	0.00	0.00	0.00	0.02	0.00	0.01	0.00	0.01	0.06
19	111	0.00	0.02	0.15	0.00	0.00	0.00	0.00	0.00	0.01	0.00	0.00	0.06

19	112	0.00	0.00	0.15	0.00	0.00	0.00	0.00	0.00	0.00	0.00	0.01	0.05
20	0	0.06	0.00	0.87	0.00	0.00	0.00	0.00	0.00	0.04	0.00	0.00	0.94
20	1	0.06	0.00	0.87	0.00	0.00	0.00	0.00	0.00	0.04	0.00	0.00	0.94
20	2	0.08	0.00	0.87	0.00	0.00	0.00	0.00	0.00	0.04	0.89	0.01	0.94
20	3	0.13	0.14	0.87	0.85	0.00	0.13	0.02	0.16	0.05	0.97	0.01	0.99
20	4	0.24	0.46	0.95	0.90	0.00	0.16	0.02	0.11	0.09	1.22	0.02	1.07
20	5	0.26	0.41	0.85	0.94	0.00	0.18	0.02	0.28	0.14	1.37	0.07	1.13
20	6	0.39	0.67	0.94	0.96	0.00	0.21	0.02	0.33	0.20	1.56	0.12	1.18
20	7	0.45	0.77	0.83	0.96	0.00	0.24	0.02	0.31	0.27	1.78	0.20	1.22
20	8	0.60	0.88	0.75	0.94	0.00	0.26	0.02	0.38	0.35	2.11	0.28	1.24
20	9	0.67	1.01	0.81	0.89	0.00	0.29	0.02	0.42	0.43	2.23	0.36	1.25
20	10	0.57	1.10	0.67	0.84	0.00	0.31	0.02	0.57	0.48	2.53	0.49	1.25
20	11	0.88	1.17	0.77	0.77	0.00	0.32	0.02	0.67	0.58	2.64	0.57	1.23
20	12	1.03	1.16	0.95	0.80	0.00	0.34	0.02	0.75	0.63	2.72	0.60	1.23
20	13	1.16	1.17	1.14	0.85	0.00	0.39	0.02	0.80	0.62	2.85	0.52	1.30
20	14	1.27	1.20	1.42	0.85	0.00	0.44	0.02	0.95	0.60	2.86	0.57	1.33
20	15	1.40	1.12	1.70	0.93	0.00	0.50	0.02	1.03	0.58	2.90	0.55	1.35
20	16	1.51	1.13	1.79	0.97	0.00	0.56	0.02	1.11	0.55	2.82	0.50	1.35
20	17	1.56	1.16	2.26	1.00	0.00	0.61	0.02	1.19	0.52	2.84	0.49	1.35
20	18	1.68	1.04	2.49	1.03	0.00	0.67	0.02	1.24	0.49	2.81	0.48	1.32
20	19	1.68	1.07	2.67	1.03	0.00	0.73	0.02	1.18	0.46	2.54	0.45	1.28
20	20	1.76	1.04	2.67	1.00	0.00	0.78	0.02	1.07	0.43	2.45	0.48	1.27
20	21	1.69	1.14	2.70	0.98	0.00	0.82	0.02	1.08	0.39	2.32	0.53	1.32
20	22	1.67	1.16	2.69	0.93	0.00	0.86	0.02	1.11	0.35	2.34	0.59	1.34
20	23	1.73	1.15	2.63	0.86	0.00	0.91	0.02	1.10	0.32	2.21	0.62	1.36
20	24	1.66	1.17	2.55	0.79	0.00	0.93	0.01	0.99	0.29	2.00	0.61	1.36
20	25	1.64	1.19	2.46	0.72	0.01	0.96	0.02	0.98	0.29	2.09	0.71	1.43
20	26	1.50	1.20	2.46	0.68	0.02	0.97	0.02	0.93	0.32	1.90	0.74	1.50
20	27	1.44	1.19	2.38	0.60	0.03	0.97	0.02	0.86	0.33	1.94	0.80	1.52
20	28	1.18	1.19	2.25	0.55	0.06	0.97	0.02	0.77	0.36	1.94	0.85	1.61
20	29	1.38	1.25	2.23	0.48	0.08	0.95	0.02	0.76	0.38	2.13	0.92	1.67
20	30	1.38	1.34	2.21	0.47	0.11	0.93	0.02	0.73	0.40	2.04	1.00	1.73
20	31	1.42	1.40	2.23	0.50	0.15	0.89	0.02	0.74	0.41	2.41	1.11	1.79
20	32	1.44	1.43	2.19	0.51	0.18	0.85	0.02	0.69	0.42	2.30	1.09	1.81
20	33	1.33	1.48	2.04	0.52	0.22	0.80	0.02	0.70	0.43	2.56	1.27	1.81
20	34	1.16	1.48	2.00	0.53	0.26	0.75	0.02	0.66	0.44	2.66	1.30	1.78

20	35	1.20	1.42	1.94	0.52	0.29	0.69	0.02	0.67	0.46	2.68	1.31	1.73
20	36	1.13	1.39	1.75	0.51	0.29	0.61	0.02	0.71	0.46	2.65	1.21	1.63
20	37	1.01	1.36	1.42	0.53	0.29	0.54	0.02	0.69	0.48	2.57	1.27	1.51
20	38	1.00	1.30	0.98	0.54	0.29	0.49	0.02	0.69	0.48	2.67	1.19	1.36
20	39	1.03	1.24	0.84	0.53	0.28	0.42	0.02	0.74	0.48	2.61	1.11	1.21
20	40	1.02	1.19	0.70	0.55	0.26	0.35	0.02	0.62	0.47	2.52	1.16	1.06
20	41	1.01	1.07	0.64	0.61	0.25	0.29	0.02	0.64	0.47	2.50	1.17	0.93
20	42	0.78	1.01	0.53	0.59	0.23	0.20	0.02	0.50	0.46	2.22	1.20	0.75
20	43	0.55	0.93	0.33	0.56	0.21	0.17	0.02	0.24	0.44	1.80	1.21	0.56
20	44	0.43	0.93	0.36	0.54	0.19	0.13	0.02	0.10	0.37	1.55	1.17	0.37
20	45	0.28	0.85	0.29	0.51	0.18	0.09	0.02	0.03	0.34	1.42	0.99	0.31
20	46	0.12	0.67	0.10	0.47	0.17	0.07	0.02	0.03	0.25	1.13	0.77	0.27
20	47	0.19	0.53	0.11	0.42	0.16	0.05	0.02	0.00	0.17	0.79	0.59	0.22
20	48	0.07	0.28	0.16	0.37	0.16	0.04	0.02	0.04	0.10	0.75	0.43	0.17
20	49	0.00	0.22	0.15	0.31	0.15	0.02	0.02	0.02	0.05	0.42	0.27	0.16
20	50	0.00	0.13	0.20	0.25	0.13	0.01	0.02	0.00	0.02	0.35	0.11	0.13
20	51	0.00	0.00	0.10	0.20	0.12	0.00	0.02	0.01	0.01	0.21	0.01	0.11
20	52	0.00	0.04	0.10	0.16	0.11	0.00	0.02	0.02	0.00	0.11	0.01	0.11
20	53	0.00	0.04	0.17	0.13	0.10	0.00	0.02	0.01	0.01	0.00	0.00	0.09
20	54	0.00	0.00	0.19	0.11	0.09	0.00	0.02	0.02	0.01	0.02	0.01	0.08
20	55	0.00	0.00	0.11	0.09	0.08	0.00	0.02	0.02	0.01	0.00	0.00	0.05
20	56	0.00	0.00	0.19	0.07	0.07	0.00	0.02	0.02	0.01	0.00	0.00	0.06
20	57	0.01	0.00	0.19	0.05	0.05	0.00	0.02	0.01	0.01	0.00	0.01	0.06
20	58	0.00	0.00	0.15	0.04	0.03	0.00	0.02	0.00	0.01	0.00	0.01	0.04
20	59	0.00	0.02	0.13	0.02	0.01	0.00	0.02	0.02	0.01	0.00	0.01	0.05
20	60	0.00	0.00	0.20	0.01	0.00	0.00	0.02	0.04	0.00	0.00	0.01	0.06
20	61	0.00	0.00	0.18	0.00	0.00	0.00	0.02	0.00	0.00	0.00	0.01	0.06
20	62	0.00	0.00	0.19	0.00	0.00	0.00	0.02	0.00	0.01	0.00	0.00	0.06
20	63	0.00	0.00	0.13	0.00	0.00	0.00	0.02	0.00	0.00	0.00	0.01	0.07
20	64	0.00	0.00	0.19	0.00	0.00	0.00	0.02	0.04	0.01	0.00	0.01	0.08
20	65	0.00	0.00	0.15	0.00	0.00	0.00	0.02	0.02	0.00	0.00	0.01	0.07
20	66	0.00	0.00	0.18	0.00	0.00	0.00	0.02	0.05	0.00	0.00	0.01	0.07
20	67	0.00	0.00	0.14	0.00	0.00	0.00	0.02	0.03	0.01	0.00	0.01	0.06
20	68	0.00	0.00	0.19	0.00	0.00	0.00	0.02	0.05	0.00	0.00	0.01	0.06
20	69	0.00	0.00	0.20	0.00	0.00	0.00	0.02	0.00	0.00	0.00	0.01	0.05
20	70	0.00	0.00	0.18	0.00	0.00	0.00	0.02	0.00	0.00	0.00	0.01	0.05

20	71	0.00	0.00	0.18	0.00	0.00	0.00	0.02	0.00	0.01	0.00	0.01	0.05
20	72	0.00	0.00	0.19	0.00	0.00	0.00	0.02	0.00	0.00	0.00	0.01	0.05
20	73	0.00	0.00	0.18	0.00	0.00	0.00	0.02	0.04	0.01	0.00	0.01	0.05
20	74	0.00	0.00	0.19	0.05	0.00	0.00	0.03	0.04	0.01	0.00	0.00	0.05
20	75	0.00	0.00	0.13	0.11	0.00	0.00	0.06	0.02	0.00	0.00	0.01	0.06
20	76	0.00	0.00	0.17	0.19	0.00	0.00	0.09	0.04	0.01	0.00	0.01	0.06
20	77	0.00	0.00	0.08	0.21	0.00	0.00	0.14	0.04	0.01	0.00	0.00	0.06
20	78	0.00	0.01	0.13	0.20	0.00	0.00	0.19	0.04	0.01	0.00	0.01	0.06
20	79	0.00	0.00	0.19	0.19	0.00	0.00	0.26	0.02	0.01	0.00	0.00	0.05
20	80	0.00	0.00	0.18	0.16	0.00	0.00	0.31	0.02	0.01	0.00	0.00	0.06
20	81	0.00	0.00	0.19	0.12	0.00	0.00	0.36	0.05	0.00	0.00	0.01	0.06
20	82	0.00	0.00	0.19	0.07	0.00	0.00	0.41	0.12	0.01	0.00	0.01	0.06
20	83	0.02	0.00	0.18	0.07	0.00	0.00	0.46	0.16	0.01	0.00	0.01	0.06
20	84	0.00	0.00	0.11	0.06	0.00	0.00	0.46	0.20	0.01	0.00	0.01	0.06
20	85	0.09	0.00	0.18	0.05	0.00	0.00	0.38	0.24	0.01	0.00	0.01	0.05
20	86	0.00	0.01	0.16	0.04	0.00	0.00	0.31	0.23	0.01	0.00	0.01	0.05
20	87	0.00	0.00	0.18	0.02	0.00	0.00	0.24	0.25	0.00	0.00	0.01	0.05
20	88	0.00	0.00	0.18	0.02	0.00	0.00	0.18	0.22	0.00	0.00	0.01	0.06
20	89	0.00	0.00	0.19	0.00	0.00	0.00	0.13	0.20	0.01	0.00	0.00	0.06
20	90	0.01	0.00	0.13	0.00	0.00	0.00	0.09	0.20	0.00	0.00	0.00	0.06
20	91	0.00	0.00	0.19	0.00	0.00	0.00	0.05	0.31	0.01	0.00	0.00	0.06
20	92	0.02	0.00	0.19	0.00	0.00	0.00	0.04	0.26	0.01	0.00	0.00	0.06
20	93	0.00	0.00	0.15	0.00	0.00	0.00	0.02	0.42	0.01	0.00	0.01	0.06
20	94	0.00	0.02	0.19	0.00	0.00	0.00	0.02	0.45	0.00	0.00	0.00	0.06
20	95	0.00	0.03	0.20	0.00	0.00	0.00	0.02	0.58	0.01	0.01	0.00	0.06
20	96	0.00	0.02	0.18	0.00	0.00	0.00	0.02	0.63	0.01	0.00	0.00	0.06
20	97	0.00	0.00	0.19	0.00	0.00	0.00	0.02	0.57	0.01	0.05	0.04	0.06
20	98	0.07	0.00	0.19	0.00	0.00	0.00	0.02	0.71	0.00	0.02	0.02	0.06
20	99	0.00	0.00	0.17	0.01	0.00	0.00	0.02	0.69	0.01	0.00	0.07	0.06
20	100	0.00	0.03	0.19	0.00	0.00	0.00	0.02	0.74	0.00	0.00	0.05	0.06
20	101	0.00	0.00	0.19	0.00	0.00	0.00	0.02	0.78	0.00	0.00	0.02	0.06
21	7	0.00	0.00	1.15	0.00	0.00	0.00	0.00	0.06	0.28	0.00	0.00	1.96
21	8	0.01	0.00	1.05	0.00	0.00	0.00	0.00	0.07	0.28	0.00	0.00	2.01
21	9	0.00	0.00	1.14	0.00	0.00	0.00	0.02	0.11	0.28	0.01	0.00	2.09
21	10	0.06	0.02	1.15	0.00	0.00	0.00	0.01	0.11	0.28	0.02	0.06	2.14
21	11	0.03	0.00	1.22	0.00	0.00	0.00	0.02	0.19	0.26	0.30	0.10	2.17

21	12	0.02	0.00	1.06	0.01	0.00	0.00	0.02	0.17	0.28	0.44	0.13	2.17
21	13	0.11	0.00	1.18	0.02	0.05	0.00	0.00	0.29	0.28	0.59	0.18	2.15
21	14	0.22	0.00	1.25	0.01	0.04	0.00	0.02	0.33	0.27	0.74	0.23	2.16
21	15	0.19	0.00	1.26	0.03	0.04	0.00	0.01	0.27	0.28	1.01	0.24	2.14
21	16	0.13	0.00	1.28	0.03	0.04	0.00	0.02	0.33	0.27	0.89	0.32	2.11
21	17	0.05	0.00	1.28	0.02	0.04	0.00	0.01	0.30	0.28	1.05	0.39	2.10
21	18	0.25	0.04	1.29	0.03	0.03	0.00	0.02	0.30	0.27	1.23	0.39	2.05
21	19	0.38	0.13	1.34	0.04	0.03	0.00	0.02	0.25	0.25	1.28	0.36	2.08
21	20	0.48	0.16	1.34	0.08	0.03	0.00	0.02	0.18	0.23	1.39	0.42	2.13
21	21	0.53	0.09	1.33	0.14	0.02	0.00	0.01	0.12	0.22	1.30	0.40	2.16
21	22	0.57	0.11	1.38	0.19	0.02	0.00	0.02	0.06	0.21	1.31	0.39	2.22
21	23	0.56	0.28	1.40	0.25	0.02	0.00	0.02	0.00	0.20	1.36	0.46	2.25
21	24	0.59	0.11	1.32	0.26	0.02	0.53	0.02	0.04	0.18	1.15	0.42	2.21
21	25	0.57	0.27	1.20	0.27	0.02	0.45	0.02	0.00	0.22	1.18	0.49	2.20
21	26	0.52	0.04	1.13	0.27	0.02	0.36	0.02	0.00	0.29	1.28	0.49	2.14
21	27	0.52	0.15	0.99	0.26	0.02	0.28	0.02	0.04	0.33	1.28	0.46	2.06
21	28	0.41	0.00	0.84	0.25	0.02	0.22	0.02	0.00	0.37	1.24	0.40	1.93
21	29	0.36	0.25	0.59	0.24	0.02	0.15	0.03	0.04	0.42	1.02	0.37	1.76
21	30	0.29	0.20	0.63	0.20	0.02	0.11	0.04	0.04	0.46	0.99	0.35	1.67
21	31	0.41	0.24	0.56	0.16	0.03	0.07	0.03	0.00	0.50	0.87	0.34	1.63
21	32	0.38	0.22	0.53	0.12	0.03	0.05	0.03	0.00	0.54	1.14	0.35	1.61
21	33	0.12	0.21	0.55	0.09	0.03	0.05	0.02	0.00	0.54	1.02	0.33	1.57
21	34	0.11	0.17	0.45	0.08	0.02	0.04	0.02	0.02	0.52	1.01	0.31	1.44
21	35	0.11	0.18	0.40	0.07	0.01	0.04	0.02	0.00	0.50	0.83	0.29	1.30
21	36	0.12	0.01	0.35	0.05	0.00	0.04	0.02	0.00	0.47	0.79	0.25	1.15
21	37	0.00	0.21	0.28	0.04	0.00	0.04	0.02	0.00	0.44	0.63	0.22	1.00
21	38	0.00	0.00	0.21	0.03	0.00	0.04	0.01	0.00	0.38	0.60	0.19	0.85
21	39	0.00	0.00	0.13	0.03	0.00	0.04	0.02	0.00	0.37	0.33	0.16	0.69
21	40	0.00	0.01	0.20	0.02	0.00	0.03	0.02	0.08	0.34	0.36	0.13	0.55
21	41	0.00	0.13	0.19	0.00	0.00	0.03	0.02	0.06	0.31	0.25	0.09	0.42
21	42	0.00	0.12	0.19	0.00	0.00	0.03	0.02	0.00	0.28	0.19	0.08	0.31
21	43	0.00	0.08	0.19	0.00	0.00	0.03	0.02	0.03	0.25	0.12	0.06	0.24
21	44	0.00	0.08	0.08	0.00	0.00	0.03	0.02	0.00	0.24	0.05	0.00	0.23
21	45	0.00	0.07	0.19	0.00	0.00	0.04	0.02	0.02	0.23	0.04	0.00	0.21
21	46	0.00	0.05	0.06	0.00	0.00	0.04	0.02	0.08	0.21	0.09	0.02	0.24
21	47	0.00	0.00	0.16	0.00	0.00	0.04	0.02	0.00	0.20	0.00	0.00	0.29

21	48	0.00	0.02	0.19	0.00	0.00	0.03	0.02	0.00	0.18	0.10	0.01	0.34
21	49	0.00	0.02	0.19	0.00	0.00	0.05	0.02	0.03	0.16	0.14	0.01	0.40
21	50	0.00	0.02	0.19	0.03	0.00	0.03	0.02	0.04	0.15	0.03	0.00	0.45
21	51	0.00	0.00	0.19	0.07	0.00	0.05	0.02	0.04	0.12	0.03	0.01	0.51
21	52	0.00	0.00	0.17	0.06	0.00	0.06	0.02	0.01	0.10	0.04	0.01	0.55
21	53	0.00	0.00	0.09	0.07	0.00	0.05	0.02	0.04	0.08	0.11	0.00	0.61
21	54	0.01	0.00	0.19	0.08	0.00	0.06	0.02	0.00	0.07	0.00	0.00	0.68
21	55	0.00	0.00	0.07	0.08	0.00	0.06	0.02	0.02	0.05	0.18	0.00	0.71
21	56	0.00	0.00	0.19	0.08	0.00	0.06	0.02	0.00	0.04	0.20	0.01	0.75
21	57	0.00	0.01	0.07	0.08	0.00	0.00	0.02	0.00	0.03	0.14	0.01	0.84
21	58	0.00	0.02	0.05	0.07	0.00	0.00	0.02	0.00	0.02	0.22	0.00	0.89
21	59	0.00	0.01	0.19	0.06	0.00	0.00	0.02	0.00	0.01	0.33	0.00	0.94
21	60	0.00	0.00	0.19	0.05	0.00	0.00	0.02	0.00	0.02	0.21	0.00	0.99
21	61	0.00	0.02	0.18	0.04	0.00	0.00	0.02	0.00	0.01	0.25	0.00	1.04
21	62	0.00	0.00	0.18	0.03	0.00	0.00	0.02	0.00	0.00	0.39	0.01	1.08
21	63	0.01	0.00	0.10	0.07	0.00	0.00	0.02	0.00	0.00	0.44	0.00	1.11
21	64	0.00	0.00	0.20	0.14	0.00	0.00	0.02	0.00	0.00	0.56	0.03	1.11
21	65	0.00	0.00	0.19	0.15	0.00	0.00	0.02	0.00	0.00	0.57	0.07	1.10
21	66	0.00	0.02	0.19	0.18	0.00	0.00	0.02	0.00	0.00	0.71	0.12	1.04
21	67	0.00	0.06	0.19	0.17	0.00	0.00	0.02	0.00	0.00	0.56	0.17	0.95
21	68	0.02	0.07	0.18	0.15	0.00	0.00	0.02	0.00	0.00	0.69	0.20	0.84
21	69	0.00	0.11	0.19	0.16	0.00	0.00	0.02	0.00	0.00	0.50	0.19	0.74
21	70	0.00	0.15	0.19	0.18	0.00	0.00	0.02	0.00	0.00	0.64	0.27	0.72
21	71	0.00	0.00	0.00	0.00	0.00	0.00	0.00	0.00	0.00	0.00	0.00	0.00
21	72	0.00	0.00	0.00	0.00	0.00	0.00	0.00	0.00	0.00	0.00	0.00	0.00
21	73	0.00	0.00	0.00	0.00	0.00	0.00	0.00	0.00	0.00	0.00	0.00	0.00
21	74	0.00	0.00	0.00	0.00	0.00	0.00	0.00	0.00	0.00	0.00	0.00	0.00
22	3	0.03	0.00	0.22	0.00	0.00	0.37	0.06	0.08	0.00	0.00	0.00	0.06
22	4	0.00	0.00	0.21	0.00	0.00	0.42	0.05	0.10	0.00	0.07	0.00	0.06
22	5	0.00	0.00	0.20	0.00	0.00	0.43	0.05	0.05	0.01	0.00	0.01	0.06
22	6	0.00	0.00	0.20	0.00	0.00	0.44	0.04	0.14	0.01	0.12	0.04	0.06
22	7	0.00	0.08	0.19	0.00	0.00	0.43	0.04	0.09	0.00	0.09	0.08	0.05
22	8	0.00	0.05	0.19	0.00	0.00	0.42	0.03	0.06	0.00	0.13	0.09	0.06
22	9	0.08	0.17	0.07	0.00	0.00	0.41	0.03	0.16	0.01	0.23	0.16	0.06
22	10	0.00	0.05	0.19	0.00	0.00	0.39	0.03	0.17	0.01	0.13	0.13	0.06
22	11	0.00	0.09	0.17	0.00	0.00	0.39	0.03	0.17	0.02	0.21	0.27	0.06

22	12	0.07	0.37	0.00	0.00	0.00	0.36	0.02	0.16	0.04	0.32	0.38	0.06
22	13	0.07	0.42	0.18	0.00	0.00	0.34	0.02	0.16	0.06	0.23	0.35	0.05
22	14	0.01	0.05	0.20	0.00	0.00	0.32	0.02	0.16	0.08	0.33	0.45	0.06
22	15	0.00	0.17	0.13	0.00	0.01	0.29	0.02	0.08	0.10	0.41	0.55	0.05
22	16	0.03	0.32	0.15	0.00	0.01	0.25	0.02	0.04	0.16	0.40	0.65	0.06
22	17	0.18	0.36	0.09	0.00	0.01	0.20	0.02	0.00	0.21	0.40	0.80	0.06
22	18	0.28	0.42	0.20	0.00	0.01	0.18	0.04	0.04	0.28	0.41	0.90	0.06
22	19	0.13	0.42	0.18	0.00	0.01	0.15	0.05	0.04	0.34	0.43	0.96	0.06
22	20	0.17	0.78	0.19	0.00	0.00	0.11	0.06	0.04	0.36	0.34	0.95	0.06
22	21	0.15	0.79	0.18	0.00	0.01	0.09	0.06	0.00	0.37	0.38	0.91	0.06
22	22	0.08	0.77	0.20	0.00	0.00	0.06	0.06	0.04	0.38	0.42	0.93	0.06
22	23	0.00	0.56	0.20	0.00	0.00	0.06	0.05	0.00	0.40	0.38	0.88	0.06
22	24	0.06	0.33	0.19	0.00	0.01	0.09	0.07	0.00	0.40	0.24	0.82	0.06
22	25	0.00	0.58	0.20	0.00	0.01	0.11	0.08	0.01	0.40	0.29	0.71	0.06
22	26	0.00	0.53	0.15	0.00	0.01	0.15	0.08	0.04	0.39	0.17	0.57	0.06
22	27	0.00	0.30	0.20	0.00	0.01	0.18	0.09	0.00	0.37	0.04	0.58	0.06
22	28	0.02	0.39	0.20	0.00	0.01	0.23	0.09	0.00	0.36	0.19	0.49	0.06
22	29	0.00	0.23	0.19	0.00	0.02	0.28	0.09	0.03	0.34	0.16	0.43	0.06
22	30	0.00	0.21	0.01	0.00	0.02	0.34	0.10	0.04	0.34	0.06	0.45	0.06
22	31	0.01	0.38	0.19	0.00	0.03	0.38	0.10	0.00	0.34	0.14	0.47	0.06
22	32	0.00	0.25	0.20	0.00	0.03	0.46	0.10	0.00	0.34	0.04	0.52	0.06
22	33	0.00	0.23	0.14	0.00	0.03	0.52	0.10	0.00	0.34	0.00	0.52	0.06
22	34	0.00	0.35	0.19	0.00	0.03	0.58	0.11	0.00	0.26	0.15	0.47	0.06
22	35	0.00	0.30	0.17	0.00	0.03	0.65	0.12	0.00	0.18	0.11	0.38	0.06
22	36	0.02	0.16	0.14	0.00	0.03	0.71	0.12	0.04	0.13	0.07	0.31	0.06
22	37	0.00	0.09	0.20	0.00	0.04	0.78	0.12	0.00	0.06	0.01	0.24	0.06
22	38	0.00	0.15	0.20	0.00	0.02	0.77	0.11	0.00	0.04	0.00	0.19	0.05
22	39	0.00	0.04	0.20	0.00	0.01	0.73	0.09	0.04	0.03	0.08	0.14	0.06
22	40	0.00	0.00	0.11	0.00	0.01	0.68	0.08	0.00	0.02	0.10	0.08	0.06
22	41	0.00	0.00	0.18	0.00	0.00	0.61	0.08	0.04	0.01	0.02	0.02	0.06
22	42	0.00	0.00	0.19	0.00	0.00	0.54	0.09	0.01	0.01	0.11	0.00	0.06
22	43	0.00	0.00	0.20	0.00	0.00	0.58	0.13	0.00	0.01	0.08	0.04	0.06
22	44	0.00	0.00	0.20	0.00	0.00	0.62	0.16	0.00	0.01	0.08	0.01	0.05
22	45	0.00	0.00	0.18	0.00	0.01	0.67	0.18	0.04	0.01	0.01	0.00	0.06
22	46	0.04	0.00	0.18	0.00	0.01	0.70	0.18	0.01	0.01	0.06	0.01	0.06
22	47	0.00	0.00	0.17	0.00	0.02	0.72	0.20	0.00	0.01	0.06	0.01	0.06

22	48	0.01	0.00	0.07	0.00	0.03	0.70	0.21	0.02	0.01	0.00	0.01	0.06
22	49	0.05	0.00	0.01	0.00	0.05	0.72	0.22	0.00	0.01	0.10	0.01	0.05
22	50	0.00	0.00	0.08	0.00	0.06	0.69	0.22	0.02	0.01	0.02	0.01	0.06
22	51	0.12	0.02	0.05	0.00	0.07	0.65	0.22	0.04	0.00	0.00	0.01	0.05
22	52	0.01	0.02	0.11	0.00	0.09	0.59	0.22	0.00	0.01	0.05	0.01	0.05
22	53	0.00	0.00	0.07	0.00	0.10	0.55	0.22	0.00	0.01	0.07	0.00	0.05
22	54	0.00	0.00	0.16	0.00	0.12	0.48	0.21	0.00	0.01	0.07	0.01	0.05
22	55	0.20	0.01	0.13	0.00	0.15	0.42	0.19	0.00	0.00	0.08	0.01	0.05
22	56	0.21	0.00	0.11	0.00	0.17	0.36	0.19	0.04	0.00	0.06	0.00	0.03
22	57	0.00	0.02	0.20	0.00	0.18	0.32	0.16	0.00	0.00	0.00	0.01	0.06
22	58	0.00	0.01	0.09	0.00	0.00	0.00	0.16	0.02	0.01	0.08	0.03	0.05
22	59	0.25	0.05	0.13	0.00	0.00	0.00	0.14	0.00	0.01	0.00	0.01	0.05
22	60	0.26	0.00	0.14	0.00	0.00	0.00	0.13	0.00	0.00	0.09	0.02	0.05
22	61	0.27	0.09	0.16	0.00	0.00	0.00	0.11	0.03	0.04	0.11	0.03	0.02
22	62	0.16	0.04	0.19	0.00	0.00	0.00	0.08	0.00	0.05	0.13	0.03	0.06
22	63	0.09	0.08	0.18	0.00	0.00	0.00	0.06	0.00	0.09	0.06	0.02	0.06
22	64	0.00	0.00	0.18	0.00	0.00	0.00	0.04	0.03	0.11	0.03	0.04	0.06
22	65	0.00	0.10	0.10	0.00	0.00	0.00	0.03	0.03	0.13	0.02	0.02	0.06
22	66	0.00	0.10	0.09	0.00	0.00	0.00	0.02	0.00	0.15	0.00	0.00	0.06
22	67	0.00	0.07	0.04	0.00	0.00	0.00	0.02	0.00	0.16	0.03	0.00	0.06
22	68	0.00	0.00	0.08	0.00	0.00	0.00	0.02	0.00	0.17	0.03	0.05	0.06
22	69	0.00	0.06	0.19	0.00	0.00	0.00	0.02	0.00	0.17	0.08	0.05	0.06
22	70	0.00	0.08	0.19	0.00	0.00	0.00	0.00	0.00	0.00	0.00	0.00	0.05
23	0	0.00	0.00	0.00	0.00	0.00	0.00	0.00	0.00	0.00	0.00	0.00	0.00
23	1	0.00	0.00	0.00	0.00	0.00	0.00	0.00	0.00	0.00	0.00	0.00	0.00
X	2	0.00	0.00	0.00	0.00	0.00	0.00	0.00	0.00	0.00	0.00	0.00	0.00
X	3	0.00	0.00	0.00	0.00	0.00	0.00	0.00	0.00	0.00	0.00	0.00	0.00
X	4	0.00	0.00	0.00	0.00	0.00	0.00	0.00	0.00	0.00	0.00	0.00	0.00
X	5	0.00	0.00	0.00	0.00	0.00	0.00	0.00	0.00	0.00	0.00	0.00	0.00
X	6	0.00	0.00	0.00	0.00	0.00	0.00	0.00	0.00	0.00	0.00	0.00	0.00
X	7	0.00	0.00	0.00	0.00	0.00	0.00	0.00	0.00	0.00	0.00	0.00	0.00
X	8	0.00	0.31	0.00	0.00	0.00	0.00	0.00	0.00	0.00	0.09	0.00	0.00
X	9	0.00	0.00	0.27	0.00	0.00	0.00	0.00	0.09	0.00	0.12	0.00	0.07
X	10	0.03	0.00	0.12	0.00	0.00	0.00	0.00	0.00	0.00	0.14	0.00	0.08
X	11	0.00	0.00	0.27	0.00	0.00	0.00	0.00	0.09	0.00	0.17	0.00	0.08
X	12	0.06	0.00	0.26	0.00	0.00	0.00	0.00	0.05	0.00	0.09	0.00	0.08

X	13	0.09	0.00	0.28	0.00	0.00	0.37	0.22	0.07	0.53	0.00	0.00	0.07
X	14	0.00	0.00	0.08	0.00	0.00	0.26	0.21	0.09	0.51	0.08	0.00	0.05
X	15	0.00	0.00	0.17	0.00	0.00	0.16	0.19	0.09	0.50	0.00	0.00	0.00
X	16	0.00	0.00	0.11	0.00	0.00	0.08	0.14	0.09	0.47	0.13	0.00	0.02
X	17	0.00	0.00	0.07	0.00	0.00	0.02	0.10	0.07	0.43	0.12	0.00	0.01
X	18	0.00	0.00	0.13	0.00	0.00	0.00	0.07	0.08	0.37	0.05	0.00	0.00
X	19	0.00	0.00	0.12	0.00	0.00	0.00	0.04	0.09	0.34	0.00	0.00	0.01
X	20	0.00	0.00	0.17	0.00	0.00	0.00	0.01	0.04	0.27	0.04	0.00	0.00
X	21	0.01	0.00	0.04	0.00	0.00	0.00	0.01	0.05	0.21	0.11	0.00	0.00
X	22	0.00	0.00	0.19	0.00	0.00	0.00	0.00	0.09	0.14	0.04	0.00	0.00
X	23	0.00	0.00	0.22	0.00	0.00	0.00	0.00	0.09	0.09	0.13	0.00	0.00
X	24	0.00	0.00	0.20	0.00	0.00	0.00	0.00	0.05	0.04	0.11	0.00	0.00
X	25	0.00	0.00	0.23	0.00	0.00	0.00	0.00	0.08	0.02	0.06	0.00	0.00
X	26	0.00	0.00	0.23	0.00	0.00	0.00	0.00	0.00	0.01	0.08	0.00	0.00
X	27	0.00	0.00	0.25	0.00	0.00	0.00	0.00	0.04	0.01	0.11	0.00	0.00
X	28	0.00	0.00	0.25	0.00	0.00	0.00	0.00	0.03	0.01	0.06	0.00	0.00
X	29	0.00	0.00	0.29	0.00	0.00	0.00	0.00	0.00	0.01	0.09	0.00	0.00
X	30	0.00	0.00	0.31	0.00	0.00	0.00	0.00	0.10	0.01	0.13	0.00	0.00
X	31	0.00	0.00	0.31	0.00	0.00	0.00	0.00	0.07	0.01	0.00	0.00	0.00
X	32	0.00	0.00	0.30	0.00	0.00	0.00	0.00	0.00	0.01	0.06	0.00	0.00
X	33	0.00	0.00	0.31	0.00	0.00	0.00	0.00	0.00	0.01	0.13	0.00	0.02
X	34	0.00	0.00	0.28	0.01	0.00	0.00	0.00	0.03	0.01	0.12	0.00	0.04
X	35	0.01	0.00	0.26	0.01	0.00	0.00	0.00	0.00	0.01	0.09	0.00	0.04
X	36	0.00	0.00	0.28	0.02	0.00	0.00	0.00	0.00	0.01	0.03	0.00	0.05
X	37	0.00	0.00	0.06	0.03	0.00	0.00	0.00	0.05	0.01	0.00	0.00	0.07
X	38	0.00	0.00	0.08	0.04	0.00	0.00	0.00	0.10	0.01	0.11	0.00	0.09
X	39	0.00	0.00	0.25	0.05	0.00	0.00	0.00	0.06	0.01	0.09	0.00	0.12
X	40	0.04	0.00	0.24	0.07	0.00	0.00	0.00	0.00	0.01	0.10	0.00	0.15
X	41	0.03	0.00	0.31	0.08	0.00	0.01	0.00	0.00	0.01	0.01	0.00	0.21
X	42	0.05	0.00	0.39	0.13	0.00	0.03	0.00	0.04	0.01	0.05	0.00	0.27
X	43	0.15	0.00	0.28	0.19	0.00	0.06	0.00	0.03	0.01	0.27	0.00	0.34
X	44	0.11	0.00	0.56	0.24	0.00	0.09	0.00	0.03	0.01	0.34	0.00	0.41
X	45	0.07	0.00	0.64	0.25	0.00	0.13	0.00	0.09	0.01	0.34	0.00	0.45
X	46	0.20	0.00	0.70	0.25	0.00	0.18	0.00	0.05	0.01	0.29	0.00	0.48
X	47	0.14	0.00	0.60	0.24	0.00	0.19	0.00	0.05	0.01	0.12	0.00	0.51
X	48	0.33	0.00	0.80	0.23	0.00	0.18	0.00	0.09	0.01	0.07	0.00	0.55

X	49	0.35	0.00	0.85	0.23	0.00	0.17	0.00	0.02	0.01	0.39	0.00	0.59
X	50	0.16	0.00	0.95	0.24	0.00	0.16	0.00	0.08	0.01	0.43	0.01	0.63
X	51	0.00	0.00	1.00	0.25	0.00	0.14	0.00	0.09	0.01	0.33	0.00	0.66
X	52	0.37	0.00	1.00	0.24	0.00	0.13	0.00	0.00	0.02	0.44	0.00	0.68
X	53	0.43	0.00	1.13	0.24	0.00	0.12	0.01	0.00	0.01	0.36	0.00	0.71
X	54	0.40	0.00	1.18	0.24	0.00	0.10	0.03	0.00	0.01	0.42	0.00	0.73
X	55	0.21	0.00	1.20	0.23	0.00	0.09	0.04	0.03	0.01	0.55	0.00	0.74
X	56	0.35	0.00	1.15	0.20	0.00	0.08	0.06	0.00	0.01	0.53	0.00	0.72
X	57	0.30	0.00	1.11	0.16	0.00	0.06	0.08	0.05	0.01	0.51	0.00	0.71
X	58	0.08	0.00	0.97	0.13	0.00	0.05	0.10	0.06	0.01	0.46	0.00	0.68
X	59	0.09	0.00	0.80	0.10	0.00	0.04	0.11	0.00	0.01	0.38	0.00	0.65
X	60	0.11	0.00	0.94	0.06	0.00	0.03	0.13	0.08	0.01	0.37	0.00	0.62
X	61	0.00	0.00	0.79	0.05	0.00	0.04	0.14	0.02	0.01	0.35	0.00	0.65
X	62	0.19	0.00	0.78	0.07	0.00	0.05	0.13	0.09	0.01	0.62	0.00	0.69
X	63	0.08	0.00	0.66	0.08	0.00	0.06	0.11	0.09	0.01	0.75	0.00	0.70
X	64	0.25	0.00	0.54	0.08	0.00	0.09	0.09	0.04	0.00	0.83	0.00	0.68
X	65	0.11	0.00	0.58	0.09	0.00	0.11	0.08	0.00	0.01	1.13	0.00	0.90
X	66	0.24	0.00	0.45	0.10	0.00	0.14	0.07	0.08	0.01	1.38	0.00	1.13
X	67	0.28	0.00	0.47	0.09	0.00	0.17	0.05	0.01	0.01	1.71	0.00	1.30
X	68	0.35	0.00	0.62	0.08	0.00	0.21	0.05	0.06	0.01	1.84	0.00	1.37
X	69	0.29	0.03	0.58	0.06	0.00	0.26	0.05	0.08	0.01	1.94	0.00	1.40
X	70	0.21	0.07	0.43	0.05	0.00	0.30	0.03	0.04	0.01	1.95	0.00	1.37
X	71	0.30	0.09	0.53	0.03	0.00	0.35	0.02	0.00	0.01	1.92	0.00	1.36
X	72	0.03	0.06	0.50	0.02	0.00	0.40	0.01	0.08	0.01	1.77	0.00	1.35
X	73	0.21	0.08	0.49	0.01	0.00	0.45	0.00	0.05	0.01	1.82	0.00	1.33
X	74	0.00	0.07	0.43	0.01	0.00	0.50	0.00	0.00	0.01	1.82	0.00	1.30
X	75	0.04	0.02	0.34	0.00	0.00	0.55	0.00	0.09	0.01	1.75	0.00	1.25
X	76	0.09	0.06	0.37	0.00	0.00	0.59	0.00	0.09	0.01	1.61	0.00	1.21
X	77	0.00	0.06	0.33	0.00	0.00	0.64	0.00	0.09	0.00	1.62	0.00	1.19
X	78	0.00	0.07	0.28	0.00	0.00	0.68	0.00	0.09	0.01	1.53	0.00	1.16
X	79	0.02	0.05	0.28	0.00	0.00	0.71	0.00	0.09	0.01	1.40	0.00	1.12
X	80	0.00	0.07	0.07	0.00	0.00	0.72	0.00	0.03	0.01	1.25	0.00	1.09
X	81	0.00	0.23	0.19	0.00	0.00	0.69	0.00	0.07	0.01	1.45	0.00	1.11
X	82	0.00	0.00	0.16	0.00	0.00	0.66	0.00	0.09	0.01	1.44	0.00	1.12
X	83	0.00	0.00	0.12	0.00	0.00	0.60	0.00	0.00	0.01	1.45	0.00	1.13
X	84	0.00	0.00	0.09	0.00	0.00	0.58	0.00	0.02	0.01	1.45	0.00	1.13

X	85	0.00	0.00	0.00	0.00	0.00	0.53	0.00	0.09	0.01	1.39	0.00	1.13
X	86	0.00	0.00	0.00	0.00	0.00	0.48	0.00	0.09	0.01	1.34	0.00	1.08
X	87	0.00	0.00	0.00	0.00	0.00	0.43	0.01	0.00	0.01	1.23	0.00	0.99
X	88	0.00	0.00	0.00	0.00	0.00	0.48	0.01	0.06	0.01	1.18	0.00	0.88
X	89	0.00	0.00	0.00	0.00	0.00	0.55	0.01	0.09	0.01	1.04	0.00	0.76
X	90	0.00	0.00	0.00	0.00	0.00	0.62	0.01	0.09	0.01	0.71	0.02	0.62
X	91	0.00	0.00	0.00	0.00	0.00	0.69	0.01	0.04	0.01	0.76	0.00	0.53
X	92	0.00	0.00	0.01	0.00	0.00	0.76	0.01	0.05	0.01	0.58	0.00	0.45
X	93	0.00	0.00	0.01	0.00	0.00	0.83	0.01	0.09	0.01	0.40	0.02	0.36
X	94	0.00	0.01	0.00	0.00	0.00	0.89	0.01	0.07	0.01	0.42	0.00	0.28
X	95	0.02	0.01	0.00	0.00	0.00	0.95	0.01	0.09	0.01	0.32	0.00	0.21
X	96	0.00	0.00	0.00	0.00	0.00	1.01	0.00	0.08	0.01	0.29	0.00	0.17
X	97	0.00	0.00	0.00	0.00	0.00	1.07	0.00	0.06	0.02	0.24	0.01	0.14
X	98	0.00	0.00	0.00	0.00	0.00	1.12	0.00	0.03	0.01	0.03	0.00	0.19
X	99	0.00	0.00	0.00	0.00	0.00	1.16	0.01	0.07	0.01	0.24	0.00	0.23
X	100	0.00	0.00	0.01	0.00	0.00	1.18	0.01	0.00	0.01	0.20	0.00	0.24
X	101	0.00	0.00	0.00	0.00	0.00	1.21	0.02	0.09	0.01	0.28	0.00	0.28
X	102	0.00	0.00	0.00	0.00	0.00	1.20	0.03	0.09	0.01	0.30	0.00	0.30
X	103	0.00	0.00	0.00	0.00	0.00	1.20	0.04	0.00	0.01	0.27	0.00	0.32
X	104	0.00	0.00	0.00	0.00	0.00	1.22	0.05	0.09	0.01	0.35	0.00	0.37
X	105	0.00	0.00	0.00	0.00	0.00	1.31	0.06	0.09	0.01	0.36	0.00	0.47
X	106	0.00	0.00	0.00	0.00	0.00	1.38	0.03	0.09	0.01	0.49	0.00	0.61
X	107	0.00	0.00	0.07	0.00	0.00	1.44	0.01	0.00	0.01	0.64	0.00	0.75
X	108	0.02	0.00	0.13	0.00	0.00	1.50	0.00	0.09	0.01	0.65	0.00	0.91
X	109	0.00	0.00	0.18	0.00	0.00	1.55	0.00	0.08	0.00	0.85	0.05	1.05
X	110	0.01	0.00	0.14	0.00	0.00	1.58	0.00	0.05	0.01	0.99	0.09	1.19
X	111	0.00	0.00	0.29	0.00	0.00	1.61	0.00	0.08	0.01	1.13	0.11	1.24
X	112	0.00	0.00	0.30	0.00	0.00	1.62	0.00	0.01	0.01	1.19	0.08	1.26
X	113	0.00	0.00	0.33	0.00	0.00	1.62	0.00	0.00	0.01	1.30	0.11	1.26
X	114	0.00	0.00	0.18	0.00	0.00	1.62	0.00	0.07	0.01	1.28	0.11	1.20
X	115	0.07	0.00	0.30	0.00	0.00	1.60	0.00	0.07	0.01	1.42	0.12	1.22
X	116	0.22	0.00	0.29	0.00	0.00	1.56	0.00	0.01	0.01	1.47	0.15	1.17
X	117	0.18	0.00	0.10	0.00	0.00	1.56	0.00	0.02	0.01	1.47	0.14	1.13
X	118	0.21	0.00	0.25	0.01	0.00	1.54	0.00	0.08	0.04	1.50	0.21	1.08
X	119	0.26	0.00	0.25	0.01	0.00	1.50	0.00	0.06	0.06	1.46	0.25	1.02
X	120	0.33	0.00	0.11	0.02	0.00	1.47	0.01	0.08	0.11	1.41	0.28	0.97

X	121	0.46	0.00	0.05	0.04	0.00	1.46	0.01	0.09	0.17	1.50	0.34	0.91
X	122	0.36	0.00	0.19	0.06	0.00	1.48	0.01	0.03	0.22	1.59	0.42	0.83
X	123	0.44	0.00	0.17	0.07	0.00	1.50	0.01	0.05	0.28	1.63	0.47	0.76
X	124	0.35	0.00	0.08	0.15	0.00	1.51	0.01	0.09	0.35	1.66	0.58	0.76
X	125	0.67	0.00	0.38	0.26	0.00	1.51	0.01	0.10	0.43	1.96	0.66	0.80
X	126	0.97	0.00	0.51	0.39	0.00	1.52	0.02	0.05	0.51	2.10	0.74	0.82
X	127	1.18	0.00	0.65	0.51	0.00	1.51	0.02	0.00	0.59	2.21	0.79	0.82
X	128	1.51	0.00	0.86	0.62	0.00	1.49	0.02	0.00	0.66	2.29	0.87	0.80
X	129	1.38	0.00	0.99	0.72	0.00	1.47	0.01	0.00	0.73	2.07	0.91	0.76
X	130	1.87	0.00	1.17	0.84	0.00	1.44	0.01	0.02	0.78	2.40	0.91	0.80
X	131	2.03	0.00	1.48	1.02	0.00	1.40	0.01	0.00	0.82	2.69	1.00	1.12
X	132	1.86	0.00	1.39	0.99	0.00	1.36	0.01	0.00	0.87	2.54	1.00	1.08
X	133	1.98	0.00	1.34	0.90	0.00	1.28	0.01	0.11	0.89	2.06	0.92	0.94
X	134	1.74	0.00	1.22	0.80	0.00	1.20	0.01	0.00	0.90	1.95	0.95	0.80
X	135	1.67	0.00	1.07	0.70	0.00	1.12	0.02	0.00	0.92	1.69	0.91	0.66
X	136	1.24	0.00	0.95	0.59	0.00	1.04	0.02	0.00	0.88	1.23	0.86	0.51
X	137	1.15	0.00	0.80	0.47	0.00	0.95	0.03	0.00	0.95	1.04	0.81	0.38
X	138	0.68	0.00	0.68	0.37	0.00	0.88	0.03	0.02	0.95	0.85	0.78	0.28
X	139	0.88	0.00	0.58	0.30	0.00	0.79	0.02	0.00	0.96	0.74	0.74	0.21
X	140	0.66	0.00	0.51	0.28	0.00	0.71	0.04	0.00	0.92	0.59	0.69	0.19
X	141	0.59	0.00	0.47	0.25	0.00	0.63	0.05	0.09	0.91	0.57	0.69	0.18
X	142	0.68	0.00	0.41	0.21	0.00	0.55	0.05	0.09	0.91	0.41	0.67	0.15
X	143	0.63	0.00	0.23	0.18	0.00	0.48	0.06	0.09	0.89	0.52	0.65	0.13
X	144	0.54	0.00	0.26	0.15	0.00	0.40	0.06	0.08	0.85	0.40	0.62	0.11
X	145	0.47	0.00	0.23	0.12	0.00	0.34	0.07	0.02	0.81	0.42	0.58	0.09
X	146	0.40	0.00	0.15	0.08	0.00	0.27	0.07	0.06	0.77	0.30	0.56	0.08
X	147	0.13	0.00	0.00	0.07	0.00	0.22	0.08	0.00	0.73	0.22	0.54	0.06
X	148	0.24	0.00	0.10	0.05	0.00	0.17	0.08	0.00	0.68	0.28	0.54	0.04
X	149	0.28	0.00	0.01	0.05	0.00	0.13	0.07	0.00	0.64	0.22	0.54	0.02
X	150	0.18	0.00	0.00	0.04	0.00	0.09	0.06	0.07	0.59	0.21	0.51	0.02
X	151	0.23	0.00	0.00	0.04	0.00	0.07	0.05	0.03	0.55	0.16	0.51	0.01
X	152	0.18	0.00	0.00	0.03	0.00	0.05	0.04	0.00	0.52	0.15	0.44	0.01
X	153	0.00	0.00	0.00	0.02	0.00	0.04	0.03	0.09	0.50	0.16	0.46	0.01
X	154	0.01	0.00	0.00	0.00	0.00	0.03	0.03	0.09	0.45	0.14	0.43	0.00
X	155	0.11	0.00	0.00	0.00	0.00	0.02	0.02	0.09	0.42	0.08	0.42	0.00
X	156	0.00	0.00	0.00	0.00	0.00	0.00	0.02	0.07	0.39	0.11	0.40	0.00

X	157	0.00	0.00	0.00	0.00	0.00	0.01	0.01	0.00	0.34	0.13	0.37	0.00
X	158	0.06	0.00	0.01	0.00	0.00	0.00	0.04	0.09	0.31	0.12	0.37	0.00
X	159	0.00	0.00	0.00	0.00	0.00	0.00	0.06	0.06	0.28	0.14	0.35	0.00
X	160	0.00	0.00	0.00	0.00	0.00	0.00	0.09	0.07	0.22	0.10	0.37	0.00
X	161	0.00	0.00	0.00	0.00	0.00	0.00	0.13	0.08	0.21	0.03	0.33	0.00
X	162	0.00	0.00	0.00	0.00	0.00	0.00	0.16	0.00	0.20	0.13	0.33	0.00
X	163	0.00	0.00	0.00	0.00	0.00	0.00	0.20	0.00	0.17	0.05	0.32	0.00
X	164	0.00	0.00	0.00	0.00	0.00	0.00	0.24	0.04	0.17	0.12	0.31	0.00
X	165	0.00	0.00	0.00	0.00	0.00	0.00	0.28	0.09	0.16	0.05	0.29	0.00
X	166	0.00	0.00	0.00	0.00	0.00	0.00	0.31	0.08	0.15	0.05	0.21	0.00
X	167	0.00	0.00	0.00	0.00	0.00	0.00	0.31	0.08	0.12	0.13	0.19	0.00
X	168	0.00	0.00	0.00	0.00	0.00	0.00	0.00	0.09	0.12	0.12	0.15	0.00
X	169	0.00	0.00	0.00	0.00	0.00	0.00	0.00	0.04	0.11	0.09	0.14	0.00
X	170	0.02	0.00	0.00	0.00	0.00	0.00	0.00	0.08	0.12	0.06	0.09	0.00
X	171	0.00	0.00	0.00	0.00	0.00	0.00	0.00	0.06	0.12	0.10	0.14	0.00
X	172	0.00	0.00	0.00	0.00	0.00	0.00	0.00	0.06	0.12	0.07	0.08	0.00
X	173	0.00	0.00	0.00	0.00	0.00	0.00	0.00	0.04	0.12	0.04	0.11	0.00
X	174	0.02	0.00	0.00	0.00	0.00	0.00	0.00	0.08	0.12	0.13	0.10	0.00
X	175	0.02	0.00	0.00	0.00	0.00	0.00	0.00	0.00	0.13	0.13	0.09	0.00
X	176	0.00	0.00	0.00	0.00	0.00	0.00	0.00	0.03	0.12	0.12	0.07	0.00
X	177	0.00	0.00	0.00	0.00	0.00	0.00	0.00	0.00	0.12	0.12	0.08	0.00
X	178	0.02	0.00	0.00	0.00	0.00	0.00	0.00	0.00	0.11	0.07	0.07	0.00
X	179	0.00	0.00	0.00	0.00	0.00	0.00	0.00	0.00	0.12	0.14	0.03	0.00
X	180	0.00	0.00	0.00	0.00	0.00	0.00	0.00	0.03	0.11	0.06	0.11	0.00
X	181	0.03	0.00	0.00	0.00	0.00	0.00	0.00	0.07	0.11	0.13	0.10	0.00
X	182	0.00	0.00	0.00	0.00	0.00	0.00	0.00	0.09	0.10	0.12	0.12	0.00
X	183	0.00	0.00	0.00	0.00	0.00	0.00	0.00	0.02	0.11	0.09	0.13	0.00
X	184	0.00	0.00	0.00	0.00	0.00	0.00	0.00	0.06	0.11	0.13	0.14	0.00
X	185	0.00	0.00	0.00	0.00	0.00	0.00	0.00	0.00	0.10	0.08	0.09	0.00
X	186	0.00	0.00	0.00	0.00	0.00	0.00	0.00	0.07	0.10	0.10	0.13	0.00
X	187	0.00	0.00	0.00	0.00	0.00	0.00	0.00	0.03	0.08	0.09	0.10	0.00
X	188	0.02	0.00	0.00	0.00	0.00	0.01	0.00	0.00	0.10	0.10	0.06	0.00
X	189	0.00	0.00	0.12	0.00	0.00	0.01	0.00	0.00	0.09	0.00	0.10	0.00

* Results for females and males
